# Strain‐Release Driven Spirocyclization of Azabicyclo[1.1.0]butyl Ketones

**DOI:** 10.1002/anie.202102754

**Published:** 2021-04-16

**Authors:** Jasper L. Tyler, Adam Noble, Varinder K. Aggarwal

**Affiliations:** ^1^ School of Chemistry University of Bristol Cantock's Close Bristol BS8 1TS UK

**Keywords:** Azabicylo[1.1.0]butanes, Azetidines, Heterocycles, Spiro compounds, Strained molecules

## Abstract

Due to their intrinsic rigidity, three‐dimensionality and structural novelty, spirocyclic molecules have become increasingly sought‐after moieties in drug discovery. Herein, we report a strain‐release driven synthesis of azetidine‐containing spirocycles by harnessing the inherent ring strain of the azabicyclo[1.1.0]butane (ABB) fragment. Novel ABB‐ketone precursors bearing silyl‐protected alcohols were synthesized in a single step and shown to engage in electrophile‐induced spirocyclization‐desilylation reactions. Primary, secondary and tertiary silyl ethers were effectively transformed into a library of new spiro‐azetidines, with a range of substituents and ring sizes. In addition, the products are generated with synthetically useful ketone and protected‐amine functional groups, which provides the potential for further elaboration and for this chemistry to be utilized in the rapid assembly of medicinally relevant compounds.

## Graphical abstract

Strain‐release driven spirocyclization: The expedient and modular construction of azetidine‐containing spirocycles harnessing the strain energy of the azabicyclo[1.1.0]butane (ABB) fragment is described. From easily accessible ABB‐ketones, electrophile‐induced spirocyclization gives access to a range of spiro‐azetidines with varying functionalities and ring sizes.

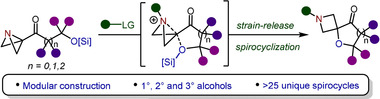

Spirocyclic molecules are gaining considerable interest within medicinal chemistry discovery programs, emanating from the constant drive to develop new and unusual scaffolds that populate regions of unexplored chemical space.^[1]^ In contrast to well‐studied aromatic ring‐based structures, which are inherently two‐dimensional, the sp^3^‐rich nature of a spirocycle is such that the exit vectors of a single unit can populate the third dimension; a feature that has been shown to improve clinical success in drug candidates.[^2^, ^3^] The rigidity imparted from the inclusion of four‐membered rings, such as an azetidine, also provides a well‐defined spatial disposition of the substituents for highly predictable vectorization.^[4]^ The identification of such advantageous properties has fuelled a surge in reports of biologically active spirocycle‐based molecules, with a number of azetidine‐containing spirocyclic lead compounds entering clinical trials or gaining approval as pharmaceuticals (Figure 1 a).^[5]^

**Figure 1:**
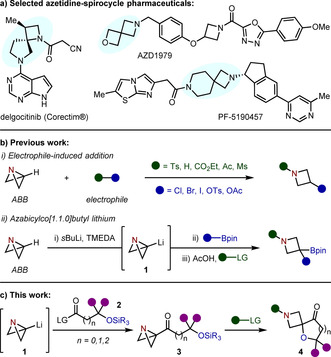
a) Selected azetidine‐containing spirocyclic pharmaceuticals. b) Previous work: (i) electrophile‐induced ring opening of ABB and (ii) ABB‐Li in the synthesis of azetidinyl boronic esters. c) This work: strain‐release driven spirocyclization of azabicyclo[1.1.0]butyl ketones. LG=leaving group.

Despite these attractive features, there is a limited range of approaches available for the synthesis of spiro‐azetidines.^[6]^ Current strategies often involve lengthy syntheses of tethered cyclic molecules before invoking a further ring‐closing step, typically through alkylation,^[7]^ cycloaddition,^[8]^ metal‐catalyzed cyclization onto alkenes or alkynes,^[9]^ or ring‐closing metathesis reactions.^[10]^ Another limitation is the difficulty in forming highly functionalized spiro‐azetidines that would be amenable to additional structural diversification. Such fragments would represent highly valuable building blocks that could facilitate the application of spiro‐azetidines in medicinal chemistry programs.^[11]^

An emerging strategy for the construction of azetidines is the use of azabicyclo[1.1.0]butane (ABB).[^12^, ^13^] Upon electrophilic activation of the nitrogen of ABB, this “spring‐loaded” fragment shows strong electrophilicity at the bridgehead carbon, where nucleophilic addition is driven by cleavage of the highly strained central bond (Figure 1 b,i).^[14]^ We recently reported that lithiation of ABB to give azabicyclobutyl‐lithium (ABB‐Li, **1**) effectively converts the bridgehead carbon into a strong nucleophile, thus providing a highly strained carbenoid species that was applied to the synthesis of 1,3‐substituted azetidine boronic esters (Figure 1 b,ii).^[15]^

We were eager to further investigate the carbenoid‐like reactivity of ABB‐Li and considered its application in the expedient and modular construction of azetidine‐containing spirocycles. We postulated that the coupling of ABB‐Li to an electrophile containing a nucleophilic atom would provide an intermediate predisposed to strain‐release driven spirocyclization reactions, thus providing a novel approach to access spiro‐azetidines from two ambiphilic coupling partners. Herein, we report the addition of ABB‐Li to silyl‐protected alcohol‐containing carboxylate esters and Weinreb amides to generate a novel class of stable azabicyclo[1.1.0]butyl ketones (ABB‐ketones). Upon electrophilic activation, these species engaged in an intramolecular spirocyclization event with concomitant oxygen desilylation to provide a diverse range of spiro‐azetidine products (Figure 1 c).

We began by investigating the synthesis of ABB‐ketones **3** by the reaction of carboxylic acid derivatives **2** with ABB‐Li (**1**), which was formed in situ from commercially available ammonium salt **5**.[^15^, ^16^, ^17^] Pleasingly, this allowed the single‐step construction of a wide variety of ABB‐ketones bearing α (**3 a**–**c**), β (**3 d**–**q**), γ (**3 r**), and δ (**3 s**) silyl‐protected alcohols (Scheme 1). In general, higher yields were obtained when using Weinreb amides (**2**, LG=N(Me)OMe) as the electrophile; however, in the case of α‐oxy ketone products **3 a**–**c** and aryl ketone products **3 t**–**v**, moderate to good yields could also be obtained when readily available carboxylate esters (**2**, LG=OMe, OEt) were used. The specific choice of silyl group was principally dictated by its proficiency in the following spirocyclization step (vide infra), which was found to be facilitated by the use of the readily cleavable TMS group. However, in the synthesis of several α‐ and β‐oxy ketones (**3 a**–**f**), and aryl ketones **3 t**–**v**, TMS ethers were too labile under the reaction conditions, necessitating the installation of more robust protecting groups (TES/TBS). Surprisingly, ABB‐ketones **3 a**–**v** were found to be stable to chromatographic purification, in contrast to related azabicyclo[1.1.0]butyl carbinols.^[16]^

**Scheme 1:**
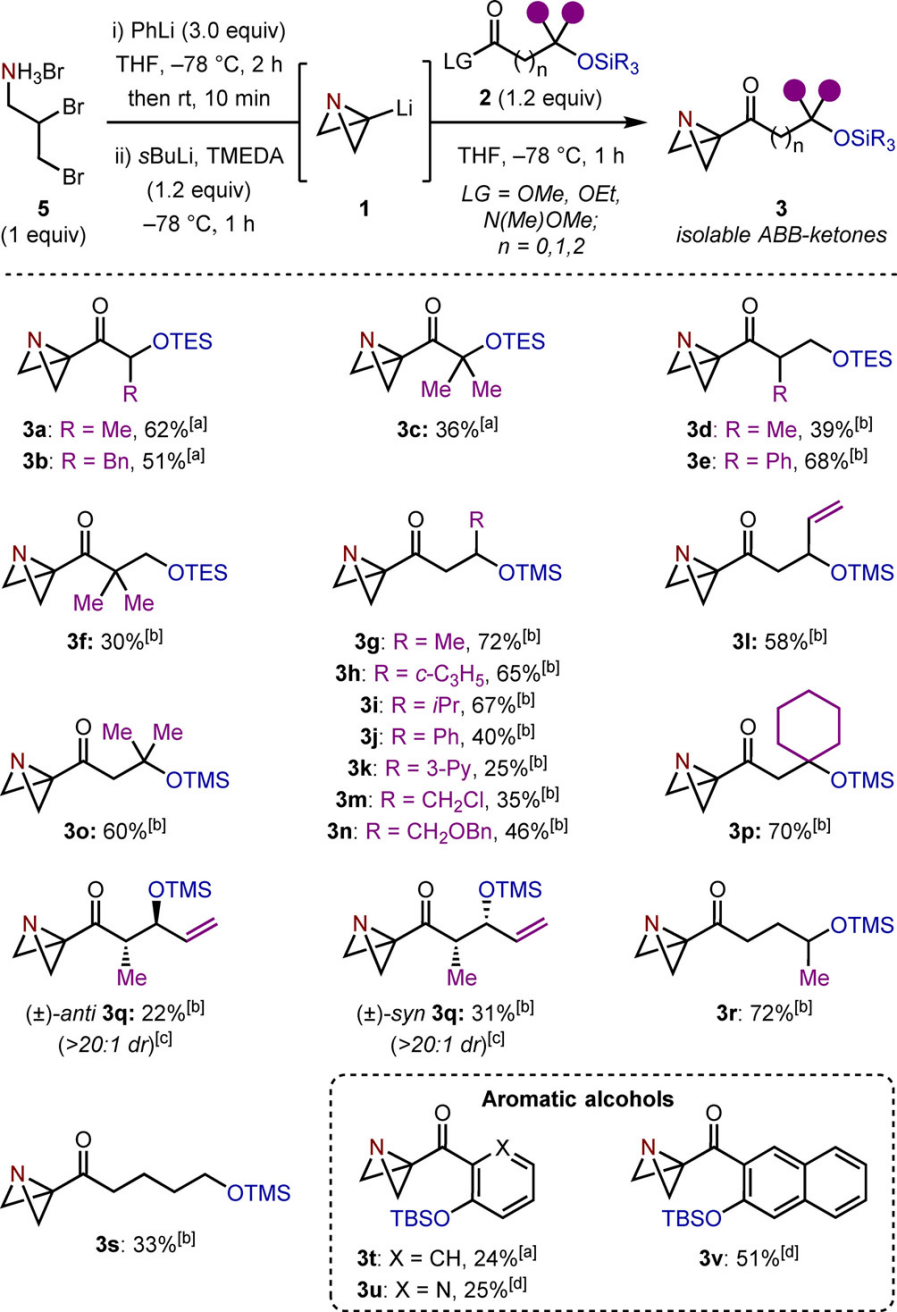
Preparation of ABB‐ketones from **1**. All reactions were carried out using ammonium salt **5** (1.0 mmol) in THF (3.6 mL), trimethylsilyl (TMS), triethylsilyl (TES) and *tert*‐butyldimethylsilyl (TBS) ethers, **2**, were added as 1.0 M solutions in THF. Isolated yields given. [a] From the corresponding ethyl ester. [b] From the corresponding Weinreb amide. [c] The diastereomeric ratio (*dr*) was determined by ^1^H NMR analysis of the crude reaction mixture. The *dr* of Weinreb amides was >20:1 [d] From the corresponding methyl ester.

With a range of silyl ether‐containing ABB‐ketones in hand, we subsequently investigated the proposed spirocyclization reaction. ABB‐ketone **3 a** was selected as the model substrate, as azaspiro[3.3]heptanes are highly sought‐after in drug discovery programs due to their favorable pharmacokinetic properties compared to commonly employed heterocyclic isosteres.^[18]^ We also anticipated that spirocyclization to generate a four‐membered ring would be more challenging than for larger ring analogues and so the solution here would be more broadly applicable. We hypothesized that, upon activation of the ABB nitrogen atom, interaction of the oxygen lone pair of the tethered silyl ether with the strongly electrophilic bridgehead carbon would facilitate concomitant desilylation, allowing for a single step spirocyclization procedure from the easily accessible ABB‐ketones.

Immediately, it became apparent that careful selection of the electrophile used for N‐activation was required (Table 1. see Supporting Information for full optimization study). We found that employing either 2,2,2‐trichloroethoxycarbonyl chloride (TrocCl) or HBr gave exclusively 3‐halo‐azetidines **6** (entries 1 and 2).^[14]^ Unfortunately, all attempts to convert **6** to the desired spirocycle upon silyl deprotection were unsuccessful, therefore, we sought alternative activators in an attempt to suppress this undesired reactivity. Lewis acids, such as boron trifluoride, failed to induce spirocyclization, instead forming uncharacterized oligomeric species (entry 3).^[19]^ Gratifyingly, treatment of **3 a** with triflic anhydride (Tf_2_O) at −78 °C provided spirocycle **4** in 21 % yield, alongside the corresponding triflate **6** (entry 4). The yield of **4** was increased to 36 % upon switching to trifluoroacetic anhydride (TFAA), which also prevented the formation of side‐product **6** (entry 5).^[20]^ Further improvements were accomplished by extending the reaction time, with a maximum yield (65 %) achieved after 3 hours at −78 °C (entries 6 and 7). Altering the equivalents of TFAA or the addition of base were both found to be deleterious to the reaction (entries 8–10). The discovery that TFAA was optimum was particularly fortuitous since the trifluoroacetamide product can be easily deprotected to unveil the free azetidine.^[21]^

**Table 1:** Optimization of the spirocyclization–desilylation reaction.^[a]^ 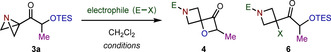

Entry	Electrophile (equiv)	Conditions	**4**: % Yield	**6**: % Yield
1	TrocCl (1.2)	0 °C, 0.5 h	0	87
2	HBr (1.2)	rt, 16 h	0	76^[b]^
3	BF_3_⋅OEt_2_ (1.2)	−78 °C−0 °C, 2 h	0^[c]^	0
4	Tf_2_O (2.0)	−78 °C, 0.5 h	21	53
5	TFAA (2.0)	−78 °C, 0.5 h	36	0
**6**	**TFAA (2.0)**	**−78 °C, 3 h**	**65 (61)^[d]^**	**0**
7	TFAA (2.0)	−78 °C, 24 h	61	0
8^[e]^	TFAA (2.0)	−78 °C, 3 h	24	0
9	TFAA (1.5)	−78 °C, 3 h	43	0
10	TFAA (3.0)	−78 °C, 3 h	50	0

[a] All reactions were carried out using **3 a** (0.10 mmol) in CH_2_Cl_2_ (1.0 mL). Yields were determined by ^1^H NMR analysis using dibromomethane as an internal standard. [b] Yield determined after protection of the azetidine with di‐*tert*‐butyl dicarbonate (Boc_2_O). [c] Only uncharacterized oligomers were observed. [d] Isolated yield. [e] With 2,6‐lutidine (2.0 equiv).

Having established optimal conditions, we then investigated the scope of the reaction (Scheme 2). In addition to secondary alcohol‐derived substrates, which provided methyl (**4 a**) and benzyl (**4 b**) monosubstituted products, tertiary alcohols also underwent high yielding spirocyclization to form disubstituted spirocycle **4 c**. The reaction was also amenable to scale‐up, with **4 a** isolated in comparable yield when prepared on a gram‐scale. Extending the carbon chain to access oxa‐azaspiro[3.4]octanes was highly favorable under the same single‐step spirocyclization–desilylation procedure, highlighting the modularity of the approach. Primary alcohol substrates bearing methyl (**4 d**), aromatic (**4 e**) and dimethyl (**4 f**) substitution patterns all performed well in the reaction from the corresponding TES ethers. However, secondary alcohol substrates were more effectively transformed into the spirocyclic products when employing TMS as the protecting group (**4 g**–**n**). A variety of functionalities were tolerated at the carbonyl β‐position including aromatic (**4 j**), heteroaromatic (**4 k**), alkenyl (**4 l**) and heteroatom (**4 m**, **4 n**) substituents. It should be noted that in the case of **4 n**, the yield was diminished by competing cyclization and debenzylation of the benzyl ether moiety, forming the corresponding oxa‐azaspiro[3.5]nonane in 15 % yield. Tertiary alcohols could also be employed, providing **4 o** and dispirocyclic compound **4 p** in excellent yields.

**Scheme 2:**
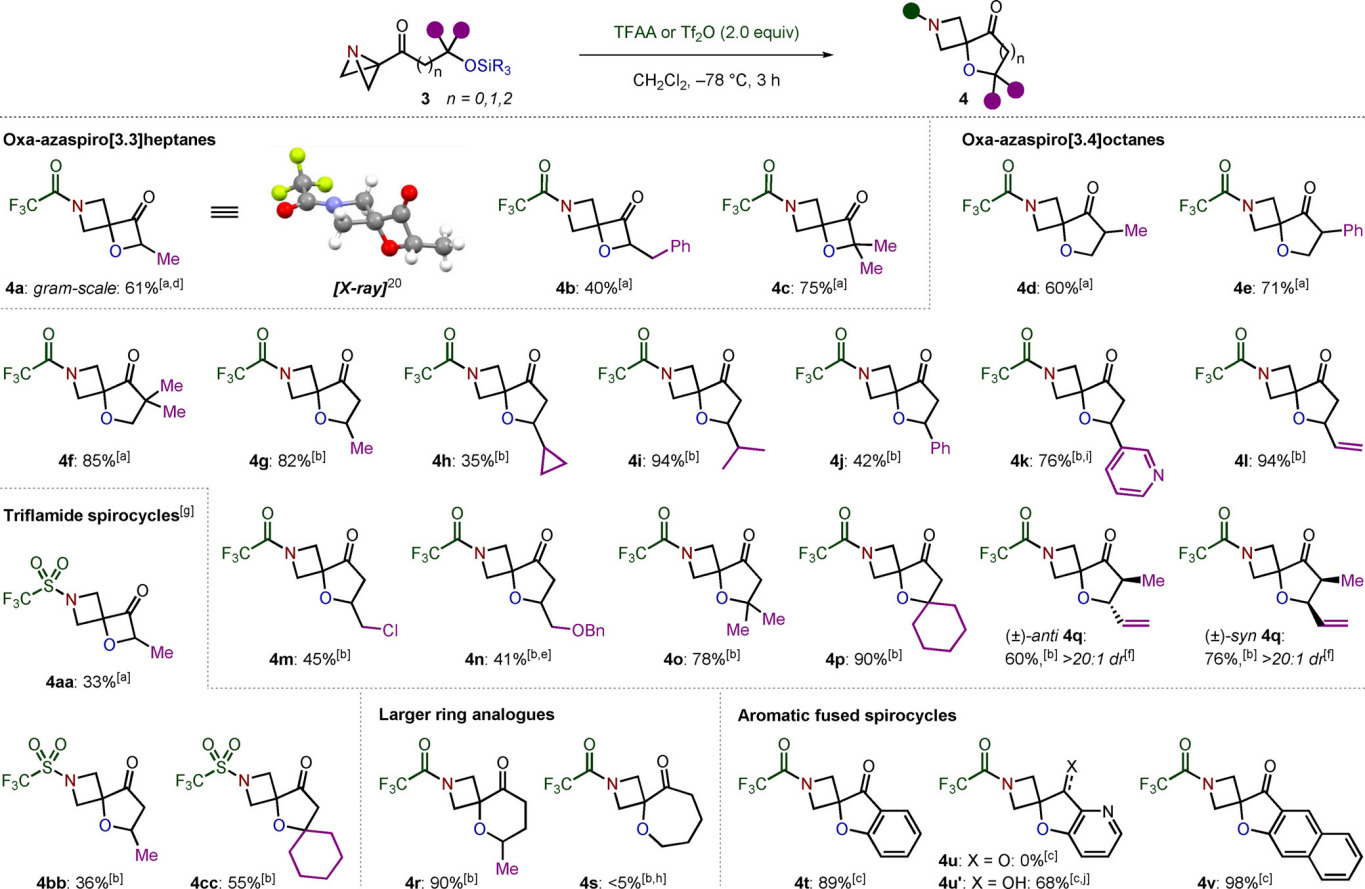
Substrate scope for the spirocyclization–desilylation reaction of ABB‐ketones. All reactions were carried out using **3** (0.10 mmol) in CH_2_Cl_2_ (1.0 mL). Isolated yields given. [a] From the corresponding TES ether. [b] From the corresponding TMS ether. [c] From the corresponding TBS ether. [d] Gram‐scale reaction was carried out with **3 a** (4.0 mmol) in CH_2_Cl_2_ (20 mL). [e] Also observed 15 % NMR yield of oxa‐azaspiro[3.5]nonane. [f] The *dr* was determined by ^1^H NMR analysis of the crude reaction mixture. The *dr* of ABB‐ketones was >20:1. [g] With 2,6‐lutidine (2.0 equiv). [h] Trace product was detected by high resolution mass spectrometry. Reaction performed at 0.02 M concentration. [i] Isolated as the trifluoroacetic acid salt. [j] From the TMS protected ABB‐carbinol. The TMS group was cleaved upon work‐up.

One of the key benefits of spirocyclic scaffolds over aromatic‐based molecules in drug discovery is that the sp^3^‐rich nature provides the opportunity to exploit point chirality.^[22]^ We therefore wanted to explore whether the spirocyclization–desilylation process is stereoretentive. We rationalized that the stereochemical integrity of both the α‐ and β‐positions could be simultaneously probed by monitoring the diastereomeric ratio (*dr*) of an α,β‐disubstituted substrate throughout the synthesis. To this end, *syn*‐**3 q** and *anti*‐**3 q** were submitted to the spirocyclization reaction to provide *syn*‐**4 q** and *anti*‐**4 q**, respectively. Pleasingly, no change in *dr* was observed,^[23]^ showing that the stereochemical integrity of the substrate is fully maintained during the spirocyclization–desilylation process.

In addition to the formation of trifluoroacetyl‐protected azetidines, the synthesis of sulfonamide products **4 aa**, **4 bb** and **4 cc** was achieved by employing triflic anhydride as the activator, albeit with decreased yields due to competing nucleophilic substitution at the bridgehead carbon of the ABB by the triflate anion (see Table 1, entry 4). Despite the weak nucleophilic character of the triflate anion, addition is nevertheless observed, presumably due to the increased electrophilicity of the ABB bridgehead carbon upon activation with triflic anhydride, resulting in a less selective reaction.^[16]^ Further extension of the carbon chain gave access to oxa‐azaspiro[3.5]nonane **4 r** in 90 % yield. However, cyclization to form the corresponding 7‐membered ring product **4 s** occurred in only trace amounts. Finally, the scope was broadened to include aryl alcohol‐based nucleophiles. TBS‐protected phenol (**4 t**), 3‐pyridinol (**4 u′**) and naphthol (**4 v**) substrates were all transformed into the spirocyclic products in good to excellent yields. Notably, in the case of **4 u**, the ketone adjacent to the pyridine was prone to nucleophilic addition and subsequent side reactions (see Supporting Information for details). However, this decomposition could be prevented by employing the analogous TMS‐protected ABB‐carbinol, which was readily deprotected upon work‐up to give alcohol **4 u′**.

During our investigations into the substrate scope, we were intrigued by the significant disparity in yields between seemingly similar substrates. For example, cyclopropyl product **4 h** was formed in only 35 % yield, compared to a 94 % yield for isopropyl product **4 i**. A comparably low yield of 42 % was also obtained for phenyl substituted product **4 j**. Further investigation into the diminished yields for the spirocyclization of both cyclopropyl ABB‐ketone **3 h** and phenyl ABB‐ketone **3 j** revealed that trifluoroacetates **7 h** and **7 j** were the major side products formed in these reactions (Table 2, entries 1 and 2). Interestingly, repeating these reactions with a bulkier silyl group (TES) almost entirely suppressed product formation and further increased the amount of side product detected (entries 3 and 4). This drastic effect, and formation of the corresponding side products, was not observed in other secondary silyl ether substrates, where the more hindered silyl protecting group simply resulted in a slight decrease in yield (see Supporting Information for details).

**Table 2:** Investigation of suboptimal substrates and comparison of protecting groups. 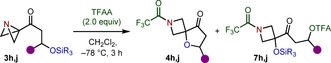

Entry	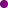	SiR_3_	**4**: % Yield^[a]^	**7**: % Yield^[b]^
1	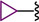	TMS: **3 h**	35	44
2	Ph	TMS: **3 j**	42	17
3	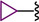	TES: **3 h′**	5^[b]^	80
4	Ph	TES: **3 j′**	trace	44

[a] Isolated yield. [b] Yields were determined by ^1^H NMR analysis using dibromomethane as an internal standard.

To rationalize these observations, we propose the following mechanism (Scheme 3). Firstly, trifluoroacyl ammonium intermediate **I** is formed upon acylation of the ABB nitrogen of **3**, which activates the bridgehead carbon towards nucleophilic substitution. Intramolecular attack of the silyl ether then ensues to construct the challenging spirocenter and give oxonium intermediate **II**, which is driven by the relief of the highly strained central bond of the ABB. Alternatively, intermolecular substitution at the bridgehead carbon by a trifluoroacetate anion to generate **6** (Pathway **a**) is also possible and can be detected for substrates where a tethered nucleophile is absent. We initially considered the possibility of **6** as an intermediate in the formation of **4**, however, subsequent attempts to promote this pathway by increasing the relative concentration of the trifluoroacetate anion with the addition of tetrabutylammonium trifluoroacetate resulted in the generation of **6** whilst simultaneously inhibiting formation of **4**, therefore suggesting this species is not an intermediate on the reaction pathway (see Supporting Information for details). After spirocyclization, cationic intermediate **II** can then undergo a trifluoroacetate‐promoted desilylation to access spirocyclic product **4** (Pathway **b**). This proposed cation‐induced intramolecular cyclization–desilylation mechanism of silyl ethers has been previously established for related cyclization reactions.^[24]^

**Scheme 3:**
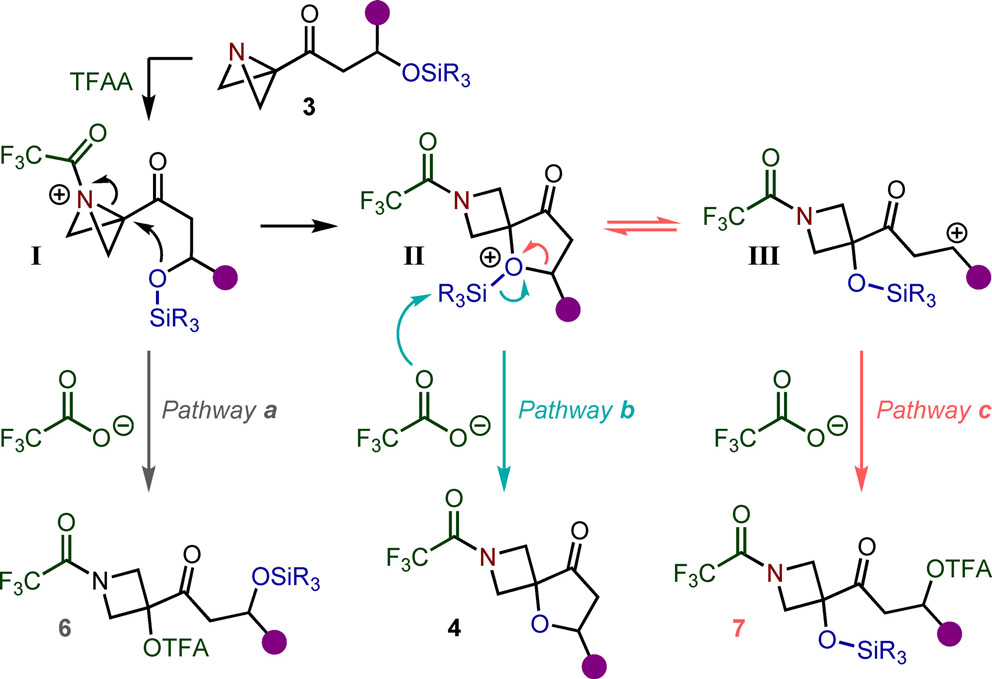
Proposed mechanism for the spirocyclization–desilylation reaction.

If a cation‐stabilizing substituent, such as phenyl or cyclopropyl, is present α to the oxonium in **II**, then competing C−O bond cleavage occurs to form carbocation **III**,^[25]^ which is trapped by a trifluoroacetate anion to give **7** (Pathway **c**). In this case, when the larger TES group is employed, the rate of desilylation of **II** is reduced, therefore favoring Pathway **c** over the desired Pathway **b**. Evidence for the undesired Pathway **c** was obtained by the identification and isolation of **7 j′**. Formation of **7 j′** also supports the intermediacy of oxonium **II**, required to facilitate this C−O bond cleavage. Additionally, the retention of the silyl ether moiety demonstrates that desilylation does not occur prior to cyclisation but must be induced by coordination to the activated ABB system.

Finally, we have demonstrated that the trifluoroacetamide group can be rapidly cleaved from the azetidine under exceptionally mild conditions (room temperature, K_2_CO_3_, MeOH:H_2_O).^[21]^ Using three different classes of spirocyclic compounds, hydrolysis and *tert*‐butyloxycarbonyl (Boc) protection was achieved in 67–83 % yield (Scheme 4).

**Scheme 4:**
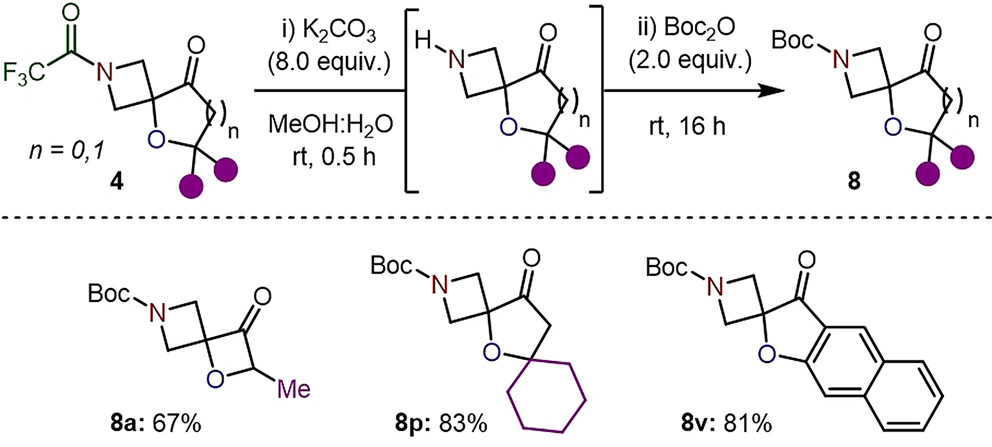
Hydrolysis and functionalization of trifluoroacetyl‐protected azetidine spirocycles **4 a**, **4 p** and **4 v**. All reactions were carried out using **4** (0.20 mmol) in MeOH:H_2_O (3:1, 2.2 mL). Isolated yields given.

In summary, we have developed a protocol for the modular construction of a diverse family of azetidine‐containing spirocycles. Novel ABB‐ketones were synthesized in a single step from ABB‐Li and found to engage directly in electrophile‐induced spirocyclization reactions. Primary, secondary and tertiary silyl‐protected alcohols were effectively transformed into a library of substituted spiro‐azetidines that incorporated 4‐, 5‐ or 6‐membered rings, as well as benzo‐fused ring systems. The ability to preserve stereogenic centers was also demonstrated by the complete retention of relative stereochemistry throughout the synthesis. The multitude of possible points for diversification of the ABB‐ketone spirocyclization precursors (substitution pattern, stereochemistry, tether length), as well as the synthetic utility of the ketone and azetidine nitrogen in the spirocyclic products, provides the potential for this chemistry to be utilized in the rapid assembly of medicinally relevant compounds.

## Conflict of interest

The authors declare no conflict of interest.

## Supporting information

As a service to our authors and readers, this journal provides supporting information supplied by the authors. Such materials are peer reviewed and may be re‐organized for online delivery, but are not copy‐edited or typeset. Technical support issues arising from supporting information (other than missing files) should be addressed to the authors.

Supplementary
