# Supplementary material for: Strain‐Release Driven Spirocyclization of Azabicyclo[1.1.0]butyl Ketones
Source: Angew Chem Int Ed Engl. 2021 Apr 16;60(21):11824–9. doi: 10.1002/anie.202102754 (PMC8251566; doi:10.1002/anie.202102754)

Angewandte Chemie
Eine Zeitschrift der Gesellschaft Deutscher Chemiker

Supporting Information

Strain-Release Driven Spirocyclization of Azabicyclo[1.1.0]butyl Ketones

*Jasper L. Tyler, Adam Noble, and Varinder K. Aggarwal**

anie_202102754_sm_miscellaneous_information.pdf

Contents

1 **General experimental** 2

1.1 Solvents, reagents, and glassware 2

1.2 Chromatography, spectroscopy, and crystallography 2

2 **Spirocyclization optimization study** 3

2.1 Electrophile screen 3

2.2 Reaction time 4

2.3 Reaction temperature 5

2.4 Electrophile stoichiometry 5

2.5 Solvent 6

2.6 Additives, addition rate and concentration 7

3 **Synthetic procedures and characterization data** 8

3.1 General Procedures 8

3.2 Synthesis of protected alcohol carboxylate esters: **2a-2c** 12

3.3 Synthesis of protected alcohol Weinreb amides: **2d-2s** 14

3.4 Synthesis of protected aromatic alcohol carboxylate esters: **2t-2v** 28

3.5 Synthesis of ABB-ketones: **3a-3v** 30

3.6 Synthesis of oxa-azaspirocycles: **4a-4v** 44

3.7 Synthesis of triflamide spirocycles: **4aa-4cc** 59

3.8 Functionalization of oxa-azaspirocycles 61

4 **Further studies of the spirocyclization-desilylation reaction** 64

4.1 Unsuccessful substrates 64

4.2 Comparison of secondary alcohol TES and TMS protecting groups 65

4.3 Effect of trifluoroacetate concentration 65

4.4 Investigation of suboptimal substrates: Representative example **3j′** 66

5 **X-ray crystal structure of 4a** 69

6 **References** 71

7 **NMR spectra** 73

1 General experimental

1.1 Solvents, reagents, and glassware

Anhydrous solvents were commercially supplied or provided by the School of Chemistry, University of Bristol, and were dried using a purification column composed of activated alumina and stored over 3 Å mol sieves. All reactions were conducted under an inert atmosphere of nitrogen using standard Schlenk manifold techniques unless stated otherwise. All glassware was flame-dried prior to use. Reagents were purchased from commercial sources and used as received. Exceptions: trifluoroacetic anhydride (TFAA) was distilled over P_2O_5 under an inert atmosphere at standard pressure, tetramethylethylenediamine (TMEDA) and trimethylsilyl chloride (TMS-Cl) were distilled over CaH_2 under an inert atmosphere at standard pressure. All organolithium and Grignard reagents were regularly titrated.

1.2 Chromatography, spectroscopy, and crystallography

Flash column chromatography was carried out using Fisher Scientific silica gel 60 (40 – 63 µm) or Sigma Aldrich Florisil® 60-100 mesh. When practical, all reactions were followed by thin-layer chromatography (TLC), using Merck Kieselgel 60 F254 fluorescent treated silica, which was visualised under UV light, or by staining an aqueous solution of $KMnO_4$ followed by heating. **^{1}H, ^{19}F and ^{13}C NMR** spectra were recorded using Jeol ECS 400 MHz, Jeol ECS 400 MHz Varian VNMR 400 MHz and Bruker Avance III HD 500 Cryo spectrometers. Chemical shifts (δ) are given in parts per million (ppm) and coupling constants (*J*) are given in Hertz (Hz), rounded to the nearest 0.1 Hz. NMR assignments were made according to spin systems, using two-dimensional NMR spectroscopy (COSY, HSQC, HMBC) to assist the characterization. Where an assignment could not be made unambiguously, no assignments are given. NMR yields were determined by ^{1}H NMR analysis using dibromomethane as an internal standard. Diastereomeric ratios (dr) were determined by ^{1}H NMR analysis of the crude reaction mixture. **High resolution mass spectra (HRMS)** were recorded on a Bruker Daltonics MicrOTOF II by Electrospray Ionisation (ESI) or a Thermo Scientific Orbitrap by Atmospheric Pressure Chemical Ionisation (APCI). **IR spectra** were recorded as a thin film facilitated with CH_2Cl_2 on a Perkin Elmer Spectrum One FT-IR. Selected absorption maxima (ν_{max}) are reported in wavenumbers (cm^{-1}). **Compound names** are those generated by ChemDraw 20.0 software (PerkinElmer), following the IUPAC nomenclature.

2 Spirocyclization optimization study

Reactions were performed using **3a** (0.10 mmol) according to General Procedure **G** (*see below*). Modifications to standard conditions and key observatios from each study are stated.

2.1 Electrophile screen

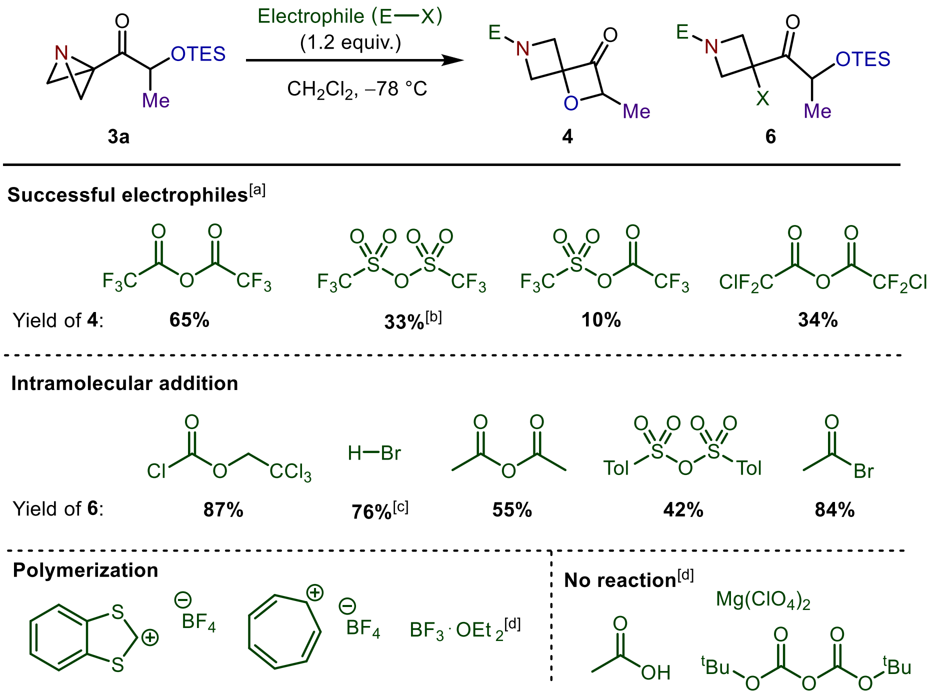

Yields were determined by ^{1}H NMR analysis using dibromomethane as an internal standard. [a] 2.0 equiv. of electrophile. [b] With 2,6-lutidine (2.0 equiv.). Isolated yield. [c] Isolated yield after protection of azetidine with Boc₂O. [d] −78 °C for 1 h, then slowly warmed to rt over 16 h.

Scheme S1. Electrophiles employed in the spirocyclization-desilylation reaction.

2.2 Reaction time

Table S1. Optimization of the spirocyclization reaction: Effect of reaction time.

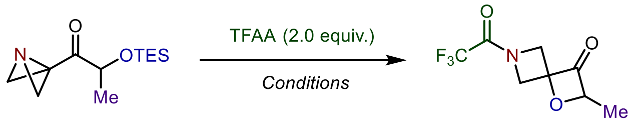

Entry	Conditions	% Yield[a]
1	CH_2Cl_2, −78 °C, 0.5 h	36
2[b]	CH_2Cl_2, −78 °C 0.5 h	12
3	CH_2Cl_2, −78 °C 3 h, rt 1 h	53
4	CH_2Cl_2, −78 °C 15 h, rt 1 h	54
5	CH_2Cl_2, −78 °C 3 h, rt over 48 h	49
6	CH_2Cl_2, −78 °C 3 h, rt over 62 h	41
**7**	**CH_2Cl_2, −78 °C, 3 h**	**65**
8	CH_2Cl_2, −78 °C, 6 h	64
9	CH_2Cl_2, −78 °C, 24 h	61

[a] Yields were determined by ^{1}H NMR analysis using dibromomethane as an internal standard. [b] From TBS protected substrate.

Key observations: The reaction was found to reach maximum conversion after 3 h at −78 °C (entry 7). The slight decrease in yield detected when warming to rt as opposed to quenching the reaction at low temperatures indicates product decomposition during this warming process (entries 3 and 4). Leaving the reaction mixture at rt for an extended period of time also results in a decrease in yield suggesting product instability under the reaction conditions at rt (entries 5 and 6).

2.3 Reaction temperature

Table S2. Optimization of the spirocyclization reaction: Effect of reaction temperature.

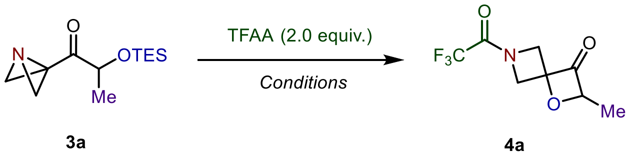

Entry	Conditions	% Yield[a]
1	CH_2Cl_2, −40 °C, 3 h	60
2	CH_2Cl_2, −20 °C, 3 h	44
3	CH_2Cl_2, 0 °C, 3 h	26
4	CH_2Cl_2, rt, 3 h	24
**5**	**CH_2Cl_2, −78 °C, 3 h**	**65**

[a] Yields were determined by ^{1}H NMR analysis using dibromomethane as an internal standard.

Key observations: Performing the reaction at −40 °C has little effect on yield (entry 1), however further increasing the temperature is deleterious to product formation (entries 2-4). Optimal temperature found to be to −78 °C (entry 5).

2.4 Electrophile stoichiometry

Table S3. Optimization of the spirocyclization reaction: Effect of electrophile stoichiometry.

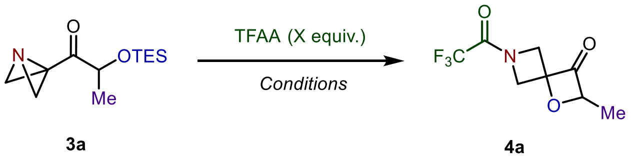

Entry	X	Conditions	% Yield[a]
1	1.2	CH_2Cl_2, −78 °C, 3 h	40
2	1.5	CH_2Cl_2, −78 °C, 3 h	43
**3**	**2.0**	**CH_2Cl_2, −78 °C, 3 h**	**65**
4	2.25	CH_2Cl_2, −78 °C, 3 h	57
5	3.0	CH_2Cl_2, −78 °C, 3 h	50

[a] Yields were determined by ^{1}H NMR analysis using dibromomethane as an internal standard.

Key observations: Optimal yield achieved using 2.0 equivalents of TFAA (entry 3).

2.5 Solvent

Table S4. Optimization of the spirocyclization reaction: Effect of solvent.

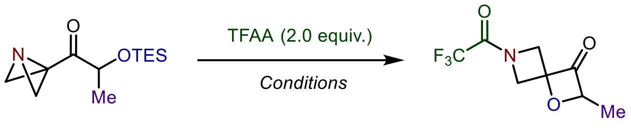

Entry	Conditions	% Yield[a]
1	THF, −78 °C, 3 h	0
2	Et_2O, −78 °C, 3 h	2
3	toluene, −78 °C, 3 h	46
4	MeCN, −40 °C, 3 h	36
5	DMF, −40 °C, 3 h	28
6	acetone, −78 °C, 3 h	11
7	EtOH, −78 °C, 3 h	0
8	nitroethane, −78 °C, 3 h	18
9	CPME, −78 °C, 3 h	0
10	hexane, −78 °C, 3 h	0
11	$CHCl_3$, −50 °C, 3 h	10

[a] Yields were determined by ^1H NMR analysis using dibromomethane as an internal standard.

Key observations: CH_2Cl_2 is the optimal solvent. In some cases, temperature was limited by freezing point of solvent.

2.6 Additives, addition rate and concentration

Table S5. Optimization of the spirocyclization reaction: Effect of additives, addition rate and concentration.

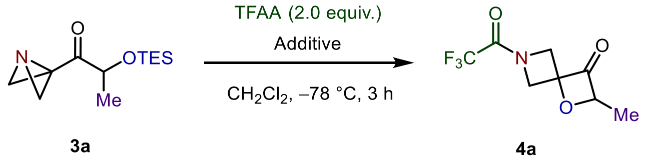

Entry	Additive	% Yield[a]
1	None (batch addition of TFAA)	55
2	None (0.17 mL/h TFAA addition rate)	18
3	2,6-lutidine (2.0 equiv.)	24
4	Et_3N (2.0 equiv.)	20
5	None (0.25 M)[b]	46
6	None (0.05 M)[b]	59

[a] Yields were determined by ^{1}H NMR analysis using dibromomethane as an internal standard. [b] Standard concentration = 0.1 M.

Key observations: Unable to further improve on yield by slowing rate of electrophile addition (entries 1-2). Optimal concentration was determined to be 0.1 M and addition of base supressed product formation (entries 3-6).

3 Synthetic procedures and characterization data

3.1 General Procedures

General Procedure A: Silyl protection of alcohols

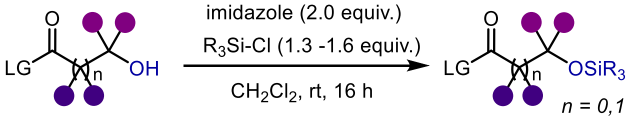

Following a modified literature procedure.[1]

Hydroxy carbonyl compounds (1.00 equiv.) were dissolved in CH_2Cl_2 (0.4 M) before the addition of imidazole (2.00 equiv.) and silyl chloride (1.30 or 1.60 equiv.). The resulting mixture was stirred at rt for 16 h then poured into H_2O. The solution was diluted with CH_2Cl_2, the organic layer separated, and the aqueous phase was extracted with CH_2Cl_2 (2×). The combined organic phases were dried ($MgSO_4$) and concentrated under reduced pressure. The residue was either purified by flash column chromatography on silica gel to yield the corresponding protected alcohol or used directly in the following reaction without further purification.

General Procedure B: Weinreb amide synthesis from esters

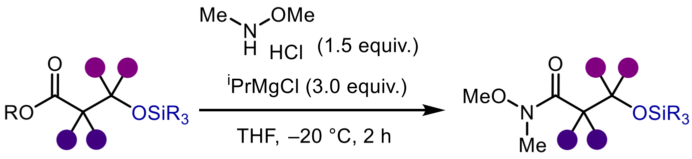

Following a modified literature procedure.[2]

Isopropylmagnesium chloride (in THF, 3.00 equiv.) was added dropwise at −20 °C to a solution of ester (1.00 equiv.) and N,O-dimethylhydroxylamine hydrochloride (1.50 equiv.) in THF (0.4 M). After stirring at −20 °C for 2 h, the reaction was quenched with sat. aq. NH_4Cl. The organic phase was separated, and the aqueous phase extracted with Et_2O (2×). The combined organic phases were then washed with H_2O, dried ($MgSO_4$) and concentrated under reduced pressure. The resulting residue was purified by flash column chromatography on silica gel to yield the corresponding Weinreb amide.

General Procedure C: Weinreb amide synthesis from carboxylic acids

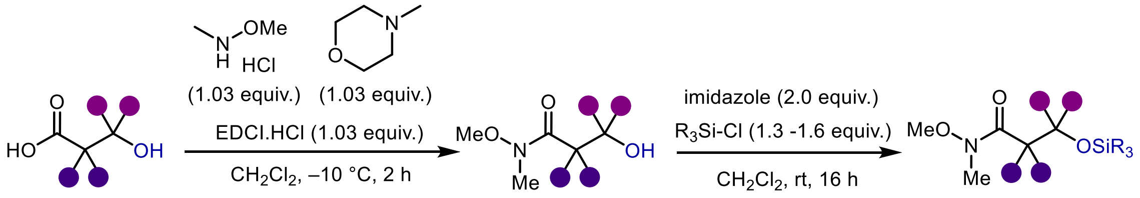

Following a modified literature procedure.[3]

N,O-Dimethylhydroxylamine hydrochloride (1.03 equiv.) and N-methylmorpholine (1.03 equiv.) were added to a solution of carboxylic acid (1.00 equiv.) in CH_2Cl_2 (0.5 M) at −10 °C, followed by the portion-wise addition of 1-(3-dimethylaminopropyl)-3-ethylcarbodiimide hydrochloride (EDCI·HCl) (1.03 equiv.) over 15 minutes. After stirring at −10 °C for 2 h, H_2O was added to the reaction. The organic phase was separated, and the aqueous phase extracted with CH_2Cl_2 (3×). The combined organic phases were dried ($MgSO_4$) and concentrated under reduced pressure. The resulting residue was then dissolved in CH_2Cl_2 (0.4 M) followed by the addition of imidazole (2.00 equiv.) and silyl chloride (1.30-1.60 equiv.). The mixture was stirred at rt for 16 h then poured into H_2O. The solution was diluted with CH_2Cl_2, the organic layer separated, and the aqueous phase was extracted with CH_2Cl_2 (2×). The combined organic phases were dried ($MgSO_4$) and concentrated under reduced pressure. The residue was purified by flash column chromatography on silica gel to yield the corresponding Weinreb amide.

General Procedure D: Weinreb amide synthesis *via* aldol reaction

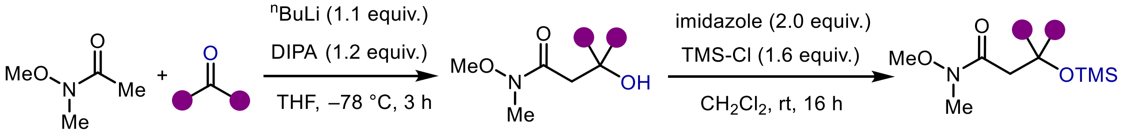

Following a modified literature procedure.[4]

^{n}BuLi (1.10 equiv.) was added dropwise[A] to a solution of diisopropylamine (DIPA) (1.20 equiv.) in THF (0.33 M) at −78 °C and stirred for 10 minutes. N-Methoxy-N-methylacetamide (1.00 equiv.) in THF (1.0 M) was then added slowly at −78 °C and the reaction stirred for a further 1 h. After this time, a solution of aldehyde/ketone (1.20 equiv.) in THF (1.0 M) was added before allowing the reaction to stir for a final 1 h at −78 °C. The reaction was then quenched at −78 °C with sat. aq. NH_4Cl and the organic phase was separated.

The aqueous phase was extracted with EtOAc (3×) and the combined organic phases were dried ($MgSO_4$) and concentrated under reduced pressure.[B] The resulting residue was then dissolved in CH_2Cl_2 (0.4 M) followed by the addition of imidazole (2.00 equiv. w.r.t alcohol) and silyl chloride (1.30 – 1.60 equiv. w.r.t alcohol). The mixture was stirred at rt for 16 h then poured into H_2O. The solution was diluted with CH_2Cl_2, the organic layer separated, and the aqueous phase was extracted with CH_2Cl_2 (2×). The combined organic phases were dried ($MgSO_4$) and concentrated under reduced pressure. The residue was purified by flash column chromatography on silica gel to yield the corresponding Weinreb amide.

Notes: (**A**) At 10 mmol scale all reagents were added *via* syringe pump at a rate of 0.5 mL/min. 0.25 mL/min at 5 mmol scale. (**B**) At this point, the yield of the corresponding alcohol product was determined by ^{1}H NMR analysis using dibromomethane as an internal standard

General Procedure E: Modified Weinreb amide synthesis from lactones

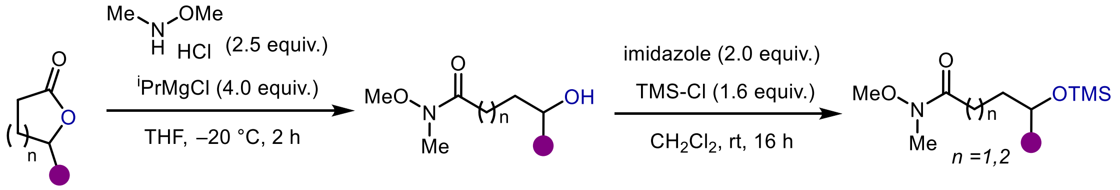

Weinreb amide formation was performed according to General Procedure **B** from: Lactone (10.0 mmol, 1.00 equiv.), N,O-dimethylhydroxylamine hydrochloride (2.50 equiv.) and isopropylmagnesium chloride (2.0 M, 4.00 equiv.). The crude alcohol was then dissolved in CH_2Cl_2 (0.4 M) followed by the addition of imidazole (2.00 equiv.) and trimethylsilyl chloride (1.60 equiv.). The resulting mixture was stirred at rt for 16 h then poured into H_2O. The solution was diluted with CH_2Cl_2, the organic layer separated, and the aqueous phase was extracted with CH_2Cl_2 (2×). The combined organic phases were dried ($MgSO_4$) and concentrated under reduced pressure. The residue was purified by flash column chromatography to afford the desired product.

General Procedure F: Synthesis of ABB-ketones

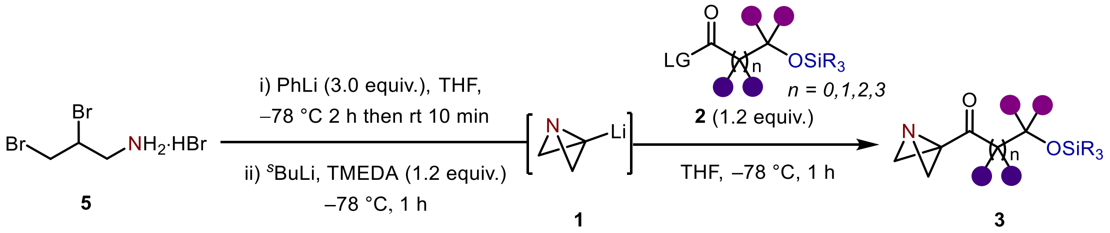

Following a modified literature procedure.[5]

Phenyl lithium (in Bu_2O, 3.00 equiv.)**[A]** was added at a rate of 0.05 mL/min *via* syringe pump to a suspension of **5** (298 mg, 1.00 mmol, 1.00 equiv.) in anhydrous THF (0.27 M) at −78 °C. After addition, the resulting solution was stirred for 2 h at −78 °C then warmed to rt for 10 minutes before cooling down again to −78 °C. **[B]** TMEDA**[C]** (0.180 mL, 1.20 equiv.) and ^{s}BuLi (in cyclohexane/hexane (95/5), 1.20 equiv.)**[A]** were then added at a rate of 0.05 mL/min *via* syringe pump, and the resulting solution was stirred for a further 1 h at −78 °C. To this solution of ABB-Li was added **2** (1.2 equiv.) as a 1.0 M solution in anhydrous THF at a rate of 0.10 mL/min *via* syringe pump and stirred at −78 °C for a final 1 h. H_2O was then added to quench the reaction, and the mixture was extracted with EtOAc (3×). The combined organic phases were dried ($MgSO_4$), filtered, and concentrated under reduced pressure. The crude residue was purified by flash column chromatography to yield the corresponding ABB-ketone.**[D,E]**

Notes: (**A**) Organolithiums should be carefully titrated prior to use. (**B**) Warming to rt at this stage is vital for reproducibility as it ensures the complete consumption of unreacted **5**. (**C**) TMEDA should be distilled over CaH_2 prior to use. (**D**) ABB-ketones bearing TMS ethers show signs of decomposition in $CDCl_3$ and so the NMR solvent was stored over K_2CO_3 to basify. (**E**) ABB compounds can be directly visualised on silica gel thin-layer chromatography (TLC) plates as a dark yellow spot that appears after prolonged heating.

General Procedure G: Spirocyclization of ABB-ketones

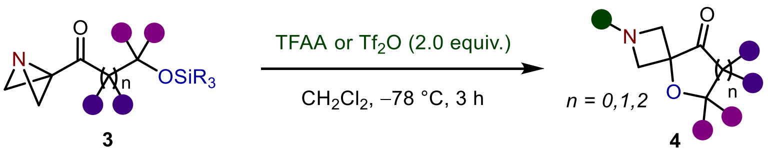

To a stirred solution of ABB-ketone (1.00 equiv.) in anhydrous CH_2Cl_2 (0.1 M) at −78 °C was added either trifluoroacetic anhydride (TFAA) (2.00 equiv.) or both triflic anhydride (Tf_2O) (2.00 equiv.) and 2,6-lutidine (2.00 equiv.) dropwise. The solution was then stirred at −78 °C for 3 h. After this time, the reaction mixture was quenched with 1:1 MeOH:CH_2Cl_2 (0.5 mL) before warming to rt. The resulting solution was concentrated under reduced pressure then directly purified by flash column chromatography to yield the corresponding spirocyclic product.

General Procedure H: Functionalization of oxa-azaspirocycles

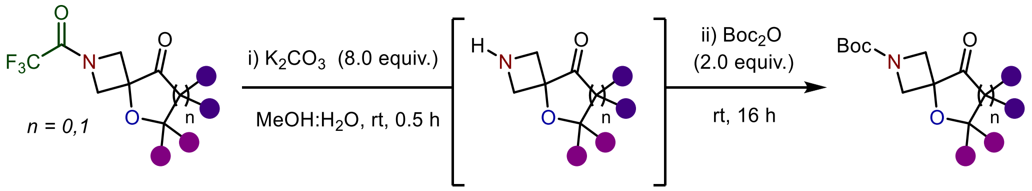

To a stirred solution of oxa-azaspirocycle (1.00 equiv.) in MeOH (0.13 M) at rt was added K_2CO_3 (8.00 equiv.) as a 2.7 M solution in H_2O. The reaction was left to stir for 30 minutes before the addition of di-*tert*-butyl dicarbonate (2.00 equiv.). The resulting mixture was stirred at rt for 16 h then poured into H_2O. The solution was diluted with EtOAc, the organic layer separated, and the organic phase was washed with H_2O and brine. The organic phase was dried ($MgSO_4$) and concentrated under reduced pressure. The residue was purified by flash column chromatography on silica gel to yield the desired product.

3.2 Synthesis of protected alcohol carboxylate esters: 2a-2c

Ethyl 2-((triethylsilyl)oxy)propanoate: 2a

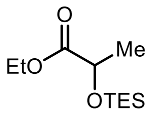

Synthesised according to General Procedure **A** from: Ethyl L-lactate (6.88 mL, 60.0 mmol, 1.00 equiv.), imidazole (8.17 g, 2.00 equiv.) and chlorotriethylsilane (13.1 mL, 1.30 equiv.). Purified by flash column chromatography (SiO_2; 19:1 hexane:EtOAc) to afford **2a** (11.8 g, 51.0 mmol, 85%) as a colourless oil.

TLC: R_f = 0.25 (19:1 hexane:EtOAc).

^{1}H NMR (400 MHz, $CDCl_3$) δ 4.29 (q, *J* = 6.8 Hz, 1H, C*H*(C=O)), 4.16 (app-qd, *J* = 7.2, 3.9 Hz, 2H, OC*H*$_2$CH$_3$), 1.38 (d, *J* = 6.8 Hz, 3H, CHC*H*$_3$), 1.26 (t, *J* = 7.2 Hz, 3H, OCH$_2$C*H*$_3$), 0.94 (t, *J* = 7.9 Hz, 9H, Si(CH$_2$C*H*$_3$)$_3$), 0.61 (q, *J* = 7.9 Hz, 6H, Si(C*H*$_2$CH$_3$)$_3$) ppm. **^{13}C NMR** (101 MHz, $CDCl_3$) δ 174.1 (*C*=O), 68.2 (*C*H), 60.8 (O*C*H$_2$CH$_3$), 21.5 (CH*C*H$_3$), 14.2 (OCH$_2$*C*H$_3$), 6.7 (Si(CH$_2$*C*H$_3$)$_3$), 4.6 (Si(*C*H$_2$CH$_3$)$_3$) ppm. (*see spectra*)

All characterization data are consistent with that reported in the literature.[6]

Ethyl 3-phenyl-2-((triethylsilyl)oxy)propanoate: 2b

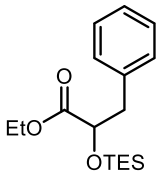

Synthesised according to General Procedure **A** from: Ethyl 2-hydroxy-3-phenylpropanoate (971 mg, 5.00 mmol, 1.00 equiv.), imidazole (681 mg, 2.00 equiv.) and chlorotriethylsilane (1.09 mL, 1.30 equiv.). Purified by flash column chromatography (SiO_2; 19:1 hexane:EtOAc) to afford **2b** (1.07 g, 3.45 mmol, 69%) as a colourless oil.

TLC: R_f = 0.22 (19:1 hexane:EtOAc).

^{1}H NMR (400 MHz, $CDCl_3$) δ 7.30 – 7.18 (m, 5H, ArC*H*), 4.33 (dd, *J* = 8.6, 4.4 Hz, 1H, C*H*(C=O)), 4.16 (q, *J* = 7.1 Hz, 2H, OCH_2CH$_3$), 3.06 (dd, *J* = 13.4, 4.4 Hz, 1H, PhCH_2), 2.90 (dd, *J* = 13.4, 8.6 Hz, 1H, PhCH_2), 1.24 (t, *J* = 7.1 Hz, 3H, OCH$_2$CH_3), 0.82 (t, *J* = 7.9 Hz, 9H, Si(CH$_2$CH_3)$_3$), 0.57 – 0.35 (m, 6H, Si(CH_2CH$_3$)$_3$) ppm. **^{13}C NMR** (101 MHz, $CDCl_3$) δ 173.2 (*C*=O), 137.5 (Ar*C*), 129.8 (Ar*C*H), 128.2 (Ar*C*H), 126.6 (Ar*C*H), 73.7 (*C*H(C=O)), 60.9 (O*C*H$_2$CH$_3$), 41.8 (Ph*C*H$_2$), 14.2 (OCH$_2$*C*H$_3$), 6.6 (Si(CH$_2$*C*H$_3$)$_3$), 4.4 (Si(*C*H$_2$CH$_3$)$_3$) ppm. (*see spectra*)

HRMS (m/z): (ESI) calc'd for $C_{17}H_{28}O_3NaSi$ $[M+Na]^+$: 331.169992, found: 331.170453.

IR (thin film) ν_{max}: 3031, 2955, 2877, 1754 (C=O), 1455, 1129 cm^{-1}.

Ethyl 2-methyl-2-((triethylsilyl)oxy)propanoate: 2c

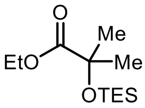

Synthesised according to General Procedure **A** from: Ethyl 2-hydroxyisobutyrate (661 mg, 5.00 mmol, 1.00 equiv.), imidazole (681 mg, 2.00 equiv.) and chlorotriethylsilane (1.09 mL, 1.30 equiv.). Purified by flash column chromatography (SiO_2; 39:1 hexane:EtOAc) to afford **2c** (870 mg, 3.55 mmol, 71%) as a colourless oil.

TLC: R_f = 0.46 (19:1 hexane:EtOAc).

^{1}H NMR (400 MHz, $CDCl_3$) δ 4.15 (q, *J* = 7.1 Hz, 2H, OCH_2CH$_3$), 1.43 (s, 6H, C(CH_3)$_2$), 1.28 (t, *J* = 7.1 Hz, 3H, OCH$_2$CH_3), 0.95 (t, *J* = 7.9 Hz, 9H, Si(CH$_2$CH_3)$_3$), 0.60 (q, *J* = 7.9 Hz, 6H, Si(CH_2CH$_3$)$_3$) ppm. **^{13}C NMR** (126 MHz, $CDCl_3$) δ 176.1 (*C*=O), 74.8 (*C*(C=O)), 61.0 (O*C*H$_2$CH$_3$), 28.8 (C(*C*H$_3$)$_3$), 14.3 (OCH$_2$*C*H$_3$), 7.1 (Si(CH$_2$*C*H$_3$)$_3$), 6.5 (Si(*C*H$_2$CH$_3$)$_3$) ppm. (*see spectra*)

HRMS (m/z): (ESI) calc'd for $C_{12}H_{26}O_3NaSi$ $[M+Na]^+$: 269.1543, found: 269.1549.

IR (thin film) ν_{max}: 2952, 2918, 1732 (C=O), 1431, 1241, 1100, 1033 cm^{-1}.

3.3 Synthesis of protected alcohol Weinreb amides: 2d-2s

Methyl 2-methyl-3-((triethylsilyl)oxy)propanoate

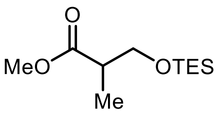

Synthesised according to General Procedure **A** from: Methyl 3-hydroxy-2-methylpropanoate (3.32 mL, 30.0 mmol, 1.00 equiv.), imidazole (4.08 g, 2.00 equiv.) and chlorotriethylsilane (6.55 mL, 1.30 equiv.). Purified by flash column chromatography (SiO_2; 19:1 hexane:EtOAc) to afford the desired product (6.02 g, 25.9 mmol, 86%) as a colourless oil.

TLC: R_f = 0.20 (49:1 hexane:EtOAc).

^{1}H NMR (500 MHz, $CDCl_3$) δ 3.79 (dd, *J* = 9.7, 7.0 Hz, 1H, C*H*$_2$), 3.67 (s, 3H, OC*H*$_3$), 3.63 (dd, *J* = 9.7, 6.2 Hz, 1H, C*H*$_2$), 2.69 – 2.61 (m, 1H, C*H*(C=O)), 1.14 (d, *J* = 7.0 Hz, 3H, CH(C*H*$_3$)), 0.94 (t, *J* = 8.0 Hz, 9H, Si(CH$_2$C*H*$_3$)$_3$), 0.58 (q, *J* = 8.0 Hz, 6H, Si(C*H*$_2$CH$_3$)$_3$) ppm. **^{13}C NMR** (126 MHz, $CDCl_3$) δ 175.6 (*C*=O), 65.1 (*C*H$_2$), 51.7 (O*C*H$_3$), 42.7 (*C*H(C=O)), 13.7 (CH(*C*H$_3$)), 6.8 (Si(CH$_2$*C*H$_3$)$_3$), 4.5 (Si(*C*H$_2$CH$_3$)$_3$) ppm. (*see spectra*)

All characterization data are consistent with that reported in the literature.[7]

N-Methoxy-N,2-dimethyl-3-((triethylsilyl)oxy)propanamide: 2d

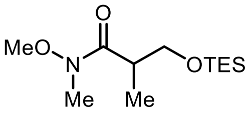

Synthesised according to General Procedure **B** from: **Methyl 2-methyl-3-((triethylsilyl)oxy)propanoate** (581 mg, 2.50 mmol, 1.00 equiv.), N,O-dimethylhydroxylamine hydrochloride (366 mg, 1.50 equiv.) and isopropylmagnesium chloride (1.3 M, 5.77 mL, 3.00 equiv.). Purified by flash column chromatography (SiO_2; 5.5:1 hexane:EtOAc) to afford **2d** (495 mg, 1.89 mmol, 76%) as a colourless oil.

TLC: R_f = 0.10 (9:1 hexane:EtOAc).

^{1}H NMR (400 MHz, $CDCl_3$) δ 3.84 (dd, *J* = 9.5, 8.1 Hz, 1H, C*H*$_2$OSi), 3.71 (s, 3H, OC*H*$_3$), 3.52 (dd, *J* = 9.5, 6.2 Hz, 1H, C*H*$_2$OSi), 3.19 (s, 3H, NC*H*$_3$), 3.17 (br. s, 1H, C*H*CH$_3$), 1.08 (d, *J* = 6.9 Hz, 3H, CHC*H*$_3$), 0.94 (t, *J* = 7.9 Hz, 9H, Si(CH$_2$C*H*$_3$)$_3$), 0.58 (q, *J* = 7.9 Hz, 6H,

$Si(CH_2CH_3)_3$) ppm. **^{13}C NMR** (101 MHz, $CDCl_3$) δ 176.1 (*C*=O), 65.4 (*C*H_2OSi), 61.4 (O*C*H_3), 38.1 (*C*HCH_3), 31.9 (N*C*H_3), 13.8 (CH*C*H_3), 6.7 (Si(CH_2*C*H_3)$_3$), 4.3 (Si(*C*H_2CH_3)$_3$) ppm. (*see spectra*)

All characterization data are consistent with that reported in the literature.[8]

N-Methoxy-N-methyl-2-phenyl-3-((triethylsilyl)oxy)propanamide: 2e

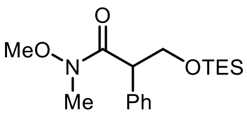

Synthesised according to General Procedure **C** from: Tropic acid (2.91 g, 17.5 mmol, 1.00 equiv.), N,O-dimethylhydroxylamine hydrochloride (1.76 g, 1.03 equiv.), N-methylmorpholine (1.98 mL, 1.03 equiv.) 1-(3-dimethylaminopropyl)-3-ethylcarbodiimide hydrochloride (EDCI·HCl) (3.46 g, 1.03 equiv.), imidazole (2.38 g, 2.00 equiv.) and triethylsilyl chloride (3.82 mL, 1.30 equiv.). Purified by flash column chromatography (SiO_2; 7.3:1 hexane:EtOAc) to afford **2e** (4.89 g, 15.1 mmol, 86% (over 2 steps)) as a colourless oil.

TLC: R_f = 0.28 (7.3:1 hexane:EtOAc).

^{1}H NMR (400 MHz, $CDCl_3$) δ 7.37 – 7.33 (m, 2H, ArC*H*), 7.31 – 7.28 (m, 2H, ArC*H*), 7.26 – 7.22 (m, 1H, ArC*H*), 4.32 – 4.24 (m, 2H, C*H*$_2$), 3.74 (app-q, *J* = 4.4 Hz, 1H, C*H*), 3.56 (s, 3H, OC*H*$_3$), 3.17 (s, 3H, NC*H*$_3$), 0.90 (t, *J* = 7.9 Hz, 9H, Si(CH_2C*H*$_3$)$_3$), 0.55 (qd, *J* = 7.9, 2.4 Hz, 6H, Si(C*H*$_2CH_3$)$_3$) ppm. **^{13}C NMR** (126 MHz, $CDCl_3$) δ 173.3 (*C*=O), 137.2 (Ar*C*), 128.6 (Ar*C*H), 127.4 (Ar*C*H), 65.6 (*C*H_2), 61.5 (O*C*H_3), 50.4 (C*H*), 32.2 (N*C*H_3), 6.8 (Si(CH_2*C*H_3)$_3$), 4.4 (Si(*C*H_2CH_3)$_3$) ppm. (*see spectra*)

All characterization data are consistent with that reported in the literature.[9]

Methyl 2,2-dimethyl-3-((triethylsilyl)oxy)propanoate

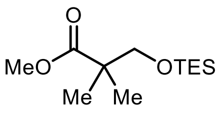

Synthesised according to General Procedure **A** from: Methyl 3-hydroxy-2,2-dimethylpropanoate (3.83 mL, 30.0 mmol, 1.00 equiv.), imidazole (4.08 g, 2.00 equiv.) and chlorotriethylsilane (6.55 mL, 1.30 equiv.). Purified by flash column chromatography (SiO_2; 32:1 hexane:EtOAc) to afford the desired product (6.11 g, 24.8 mmol, 83%) as a yellow oil.

TLC: R_f = 0.30 (32:1 hexane:EtOAc).

^{1}H NMR (400 MHz, $CDCl_3$) δ 3.66 (s, 3H, C*H*$_3$), 3.58 (s, 2H, C*H*$_2$), 1.15 (s, 6H, C(C*H*$_3$)$_2$), 0.93 (t, *J* = 7.9 Hz, 9H, Si(CH$_2$C*H*$_3$)$_3$), 0.56 (q, *J* = 7.9 Hz, 6H, Si(C*H*$_2$CH$_3$)$_3$) ppm. **^{13}C NMR** (101 MHz, $CDCl_3$) δ 177.4 (*C*=O), 70.0 (*C*H$_2$), 51.8 (O*C*H$_3$), 45.0 (*C*(CH$_3$)$_3$), 22.1 (C(*C*H$_3$)$_3$), 6.8 (Si(CH$_2$*C*H$_3$)$_3$), 4.5 (Si(*C*H$_2$CH$_3$)$_3$) ppm. (*see spectra*)

HRMS (m/z): (ESI) calc'd for $C_{12}H_{26}O_3NaSi$ [M+Na]$^+$: 269.1543, found: 269.1551.

IR (thin film) ν_{max}: 2954, 2914, 2877, 1738 (C=O), 1460, 1240, 1155, 1033, 1009 cm^{-1}.

N-Methoxy-N,2-dimethyl-3-((triethylsilyl)oxy)propanamide: 2f

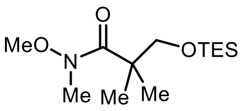

Synthesised according to General Procedure **B** from: **Methyl 2,2-dimethyl-3-((triethylsilyl)oxy)propanoate** (2.46 g, 10.0 mmol, 1.00 equiv.), N,O-dimethylhydroxylamine hydrochloride (1.46 g, 1.50 equiv.) and isopropylmagnesium chloride (2.0 M, 15.0 mL, 3.00 equiv.). Purified by flash column chromatography (SiO_2; 9:1 hexane:EtOAc) to afford **2f** (1.65 g, 5.99 mmol, 60%) as a colourless oil.

TLC: R_f = 0.20 (9:1 hexane:EtOAc).

^{1}H NMR (400 MHz, $CDCl_3$) δ 3.68 (s, 2H, C*H*$_2$OSi), 3.67 (s, 3H, OC*H*$_3$), 3.16 (s, 3H, NC*H*$_3$), 1.21 (s, 6H, C(C*H*$_3$)$_2$), 0.93 (t, *J* = 7.9 Hz, 9H, Si(CH$_2$C*H*$_3$)$_3$), 0.57 (q, *J* = 7.9 Hz, 6H, Si(C*H*$_2$CH$_3$)$_3$) ppm. **^{13}C NMR** (101 MHz, $CDCl_3$) δ 177.3 (*C*=O), 69.1 (*C*H$_2$OSi), 60.7 (O*C*H$_3$), 45.4 (*C*(CH$_3$)$_2$), 33.7 (N*C*H$_3$), 22.3 (C(*C*H$_3$)$_2$), 6.9, (Si(CH$_2$*C*H$_3$)$_3$), 4.4 (Si(*C*H$_2$CH$_3$)$_3$) ppm. (*see spectra*)

All characterization data are consistent with that reported in the literature.[9]

Ethyl 3-((trimethylsilyl)oxy)butanoate

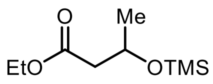

Synthesised according to General Procedure **A** from: Ethyl 3-hydroxybutanoate (1.30 mL, 10.0 mmol, 1.00 equiv.), imidazole (1.36 g, 2.00 equiv.) and trimethylsilyl chloride (2.03 mL, 1.60 equiv.) to afford the desired crude product as a colourless oil which was used without further purification.

N-Methoxy-N-methyl-3-((trimethylsilyl)oxy)butanamide: 2g

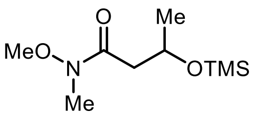

Synthesised according to General Procedure **B** from: Crude **ethyl 3-((trimethylsilyl)oxy)butanoate** (2.04 g, 10.0 mmol, 1.00 equiv.), N,O-dimethylhydroxylamine hydrochloride (1.46 g, 1.50 equiv.) and isopropylmagnesium chloride lithium chloride (2.0 M, 15.0 mL, 3.00 equiv.). Purified by flash column chromatography (SiO_2; 4:1 hexane:EtOAc) to afford **2g** (1.54 g, 7.02 mmol, 70% (over 2 steps)) as a colourless oil.

TLC: R_f = 0.22 (4:1 hexane:EtOAc).

^{1}H NMR (400 MHz, $CDCl_3$) δ 4.34 (app-dp, *J* = 7.3, 6.1 Hz, 1H, C*H*CH_3), 3.69 (s, 3H, OC*H*$_3$), 3.18 (s, 3H, NC*H*$_3$), 2.77 (dd, *J* = 14.8, 7.3 Hz, 1H, C*H*$_2$), 2.38 (dd, *J* = 14.8, 5.7 Hz, 1H, C*H*$_2$), 1.22 (d, *J* = 6.1 Hz, 3H, CHC*H*$_3$), 0.11 (s, 9H, Si(C*H*$_3$)$_3$) ppm. **^{13}C NMR** (126 MHz, $CDCl_3$) δ 172.5 (*C*=O), 65.9 (*C*HCH_3), 61.5 (O*C*H_3), 41.9 (*C*H_2), 32.1 (N*C*H_3), 24.3 (CH*C*H_3), 0.2 (Si(*C*H_3)$_3$) ppm. (*see spectra*)

HRMS (m/z): (ESI) calc'd for $C_9H_{21}NNaO_3Si$ $[M+Na]^+$: 242.1183, found: 242.1184.

IR (thin film) ν_{max}: 2959, 2901, 1660 (C=O), 1379, 1249, 1091, 998 cm^{-1}.

Ethyl 3-cyclopropyl-3-((trimethylsilyl)oxy)propanoate

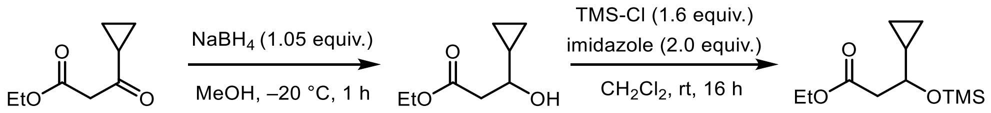

Following a modified literature procedure.[10]

Ethyl 3-cyclopropyl-3-oxopropanoate (4.43 mL, 30.0 mmol, 1.00 equiv.) was dissolved in anhydrous MeOH (30 mL) and cooled to −20 °C. $NaBH_4$ (1.19 g, 1.05 equiv.) was added portion wise and the solution was stirred at this temperature for 1 h. After this time, H_2O was added, and the solution was warmed to rt. The solution was then diluted with CH_2Cl_2, the organic layer separated, and the aqueous phase was extracted with CH_2Cl_2 (2×). The combined organic phases were dried ($MgSO_4$) and concentrated under reduced pressure. The crude alcohol was then dissolved in CH_2Cl_2 (75 mL) and protected according to General Procedure **A** from: imidazole (4.08 g, 2.00 equiv.) and trimethylsilyl chloride (6.09 mL, 1.60 equiv.) to afford the desired crude product as a colourless oil which was used without further purification.

3-Cyclopropyl-N-methoxy-N-methyl-3-((trimethylsilyl)oxy)propanamide: 2h

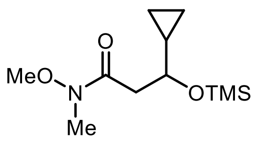

Synthesised according to General Procedure **B** from: Crude **ethyl 3-cyclopropyl-3-((trimethylsilyl)oxy)propanoate** (2.30 g, 10.0 mmol, 1.00 equiv.), N,O-dimethylhydroxylamine hydrochloride (1.46 g, 1.50 equiv.) and isopropylmagnesium chloride (2.0 M, 15.0 mL, 3.00 equiv.). Purified by flash column chromatography (SiO_2; 4:1 hexane:EtOAc) to afford **2h** (1.36 g, 5.54 mmol, 55% (over 3 steps)) as a colourless oil.

TLC: R_f = 0.28 (4:1 hexane:EtOAc).

^{1}H NMR (400 MHz, $CDCl_3$) δ 3.70 (s, 3H, OC*H*$_3$), 3.61 (app-td, *J* = 8.0, 4.6 Hz, 1H, C*H*OSi), 3.18 (s, 3H, NC*H*$_3$), 2.87 (dd, *J* = 14.3, 8.0 Hz, 1H, C*H*$_2$), 2.53 (dd, *J* = 14.3, 4.6 Hz, 1H, C*H*$_2$), 1.04 – 0.89 (m, 1H, cyclopropane C*H*), 0.54 – 0.41 (m, 2H, cyclopropane C*H*$_2$), 0.34 – 0.27 (m, 1H, cyclopropane C*H*$_2$), 0.27 – 0.21 (m, 1H, cyclopropane C*H*$_2$), 0.09 (s, 9H, Si(C*H*$_3$)$_3$) ppm. **^{13}C NMR** (126 MHz, $CDCl_3$) δ 172.5 (*C*=O), 73.7 (*C*HOSi), 61.5 (O*C*H$_3$), 40.7 (*C*H$_2$), 32.1 (N*C*H$_3$), 18.0 (cyclopropane *C*H), 3.8 (cyclopropane *C*H$_2$), 2.6 (cyclopropane *C*H$_2$), 0.5 (Si(*C*H$_3$)$_3$) ppm. (*see spectra*)

HRMS (m/z): (ESI) calc'd for $C_{11}H_{23}NNaO_3Si$ $[M+Na]^+$: 268.1339, found: 268.1346.

IR (thin film) ν_{max}: 3081, 2957, 2900, 1660 (C=O), 1384, 1249, 1049 cm^{-1}.

N-Methoxy-N,4-dimethyl-3-((trimethylsilyl)oxy)pentanamide: 2i

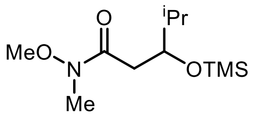

Synthesised according to General Procedure **D** from: Diisopropylamine (1.69 mL, 1.20 equiv.), ^{n}BuLi (1.60 M, 1.10 equiv.), N-methoxy-N-methylacetamide (1.02 mL, 10.0 mmol, 1.00 equiv.), isobutyraldehyde (1.10 mL, 1.20 equiv.), imidazole (885 mg, 2.00 equiv. w.r.t alcohol) and trimethylsilyl chloride (1.32 mL, 1.60 equiv. w.r.t alcohol). Purified by flash column chromatography (SiO_2; 5.7:1 hexane:EtOAc) to afford **2i** (1.28 g, 5.17 mmol, 52% (65% for 1st step)) as a colourless oil.

TLC: R_f = 0.21 (5.7:1 hexane:EtOAc).

^{1}H NMR (400 MHz, $CDCl_3$) δ 4.06 (ddd, *J* = 8.6, 4.6, 3.8 Hz, 1H, C*H*OSi), 3.69 (s, 3H, OC*H*$_3$), 3.18 (s, 3H, NC*H*$_3$), 2.73 (dd, *J* = 14.8, 8.6 Hz, 1H, C*H*$_2$), 2.33 (dd, *J* = 14.8, 3.8 Hz, 1H, C*H*$_2$),

1.70 (app-heptd, J = 6.8, 4.6 Hz, 1H, CH(CH$_3$)$_2$), 0.90 (d, J = 6.8 Hz, 3H, CH(CH_3)$_2$), 0.89 (d, J = 6.8 Hz, 3H, CH(CH_3)$_2$), 0.08 (s, 9H, Si(CH_3)$_3$) ppm. **^{13}C NMR** (126 MHz, CDCl$_3$) δ 173.1 (C=O), 74.2 (CHOSi), 61.4 (OCH$_3$), 36.8 (CH$_2$), 34.1 (CH(CH$_3$)$_2$), 32.1 (NCH$_3$), 18.7 (CH(CH$_3$)$_2$), 17.8 (CH(CH$_3$)$_2$), 0.5 (Si(CH$_3$)$_3$) ppm. (*see spectra*)

HRMS (m/z): (ESI) calc'd for $C_{11}H_{25}NNaO_3Si$ $[M+Na]^+$: 270.149591, found: 270.150116.

IR (thin film) ν_{max}: 2959, 2898, 1662 (C=O), 1385, 1248, 1079, 839 cm^{-1}.

N-Methoxy-N-methyl-3-phenyl-3-((trimethylsilyl)oxy)propanamide: 2j

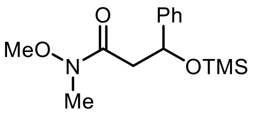

Synthesised according to General Procedure **D** from: Diisopropylamine (1.69 mL, 1.20 equiv.), ^{n}BuLi (1.60 M, 1.10 equiv.), N-methoxy-N-methylacetamide (1.02 mL, 10.0 mmol, 1.00 equiv.), benzaldehyde (1.22 mL, 1.20 equiv.), imidazole (953 mg, 2.00 equiv. w.r.t alcohol) and trimethylsilyl chloride (1.42 mL, 1.60 equiv. w.r.t alcohol). Purified by flash column chromatography (SiO$_2$; 4:1 hexane:EtOAc) to afford **2j** (1.69 g, 6.01 mmol, 60% (70% for 1st step)) as a colourless oil.

TLC: R_f = 0.27 (4:1 hexane:EtOAc).

^{1}H NMR (400 MHz, CDCl$_3$) δ 7.41 – 7.36 (m, 2H, ArCH), 7.36 – 7.27 (m, 2H, ArCH), 7.26 – 7.21 (m, 1H, ArCH), 5.26 (dd, J = 9.0, 4.3 Hz, 1H, CHOSi), 3.64 (s, 3H, OCH_3), 3.18 (s, 3H, NCH_3), 3.04 (dd, J = 14.7, 9.0 Hz, 1H, CH_2), 2.56 (dd, J = 14.7, 4.3 Hz, 1H, CH_2), 0.03 (s, 9H, Si(CH_3)$_3$) ppm. **^{13}C NMR** (126 MHz, CDCl$_3$) δ 171.9 (C=O), 144.8 (ArC), 128.4 (ArCH), 127.4 (ArCH), 125.9 (ArCH), 71.8 (CHOSi), 61.4 (OCH$_3$), 43.2 (CH$_2$), 32.1 (NCH$_3$), 0.2 (Si(CH$_3$)$_3$) ppm. (*see spectra*)

All characterization data are consistent with that reported in the literature.[11]

N-Methoxy-N-methyl-3-(pyridin-3-yl)-3-((trimethylsilyl)oxy)propanamide: 2k

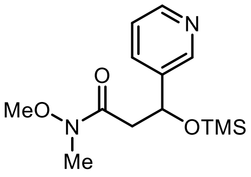

Synthesised according to General Procedure **D** from: Diisopropylamine (0.847 mL, 1.20 equiv.), ^{n}BuLi (1.60 M, 1.10 equiv.), N-methoxy-N-methylacetamide (0.511 mL, 5.00 mmol, 1.00 equiv.), nicotinaldehyde (0.563 mL, 1.20 equiv.), imidazole (451 mg, 2.00 equiv. w.r.t alcohol) and trimethylsilyl chloride (0.672 mL, 1.60 equiv. w.r.t alcohol). Purified by flash column

chromatography (SiO_2; 0.43:1 hexane:EtOAc) to afford **2k** (0.846 g, 3.00 mmol, 60% (66% for 1st step)) as a yellow oil.

TLC: R_f = 0.20 (0.43:1 hexane:EtOAc).

^{1}H NMR (400 MHz, $CDCl_3$) δ 8.64 (d, *J* = 2.2 Hz, 1H, ArC*H*), 8.51 (dd, *J* = 4.8, 1.6 Hz, 1H, ArC*H*), 7.70 (ddd, *J* = 7.8, 2.2, 1.6 Hz, 1H, ArC*H*), 7.25 (dd, *J* = 7.8, 4.8 Hz, 1H, ArC*H*), 5.30 (dd, *J* = 8.6, 4.7 Hz, 1H, C*H*OSi), 3.65 (s, 3H, OCH_3), 3.17 (s, 3H, NCH_3), 3.05 (dd, *J* = 14.9, 8.6 Hz, 1H, CH_2), 2.59 (dd, *J* = 14.9, 4.7 Hz, 1H, CH_2), 0.05 (s, 9H, Si(CH_3)$_3$) ppm. **^{13}C NMR** (126 MHz, $CDCl_3$) δ 171.3 (*C*=O), 149.0 (Ar*C*H), 148.0 (Ar*C*H), 140.0 (Ar*C*), 133.7 (Ar*C*H), 123.4 (Ar*C*H), 69.7 (*C*HOSi), 61.5 (O*C*H_3), 43.0 (*C*H_2), 32.1 (N*C*H_3), 0.1 (Si(*C*H_3)$_3$) ppm. (*see spectra*)

HRMS (m/z): (ESI) calc'd for $C_{13}H_{22}N_2NaO_3Si$ $[M+Na]^+$: 305.129190, found: 305.129873.

IR (thin film) ν_{max}: 2958, 2898, 1656 (C=O), 1425, 1250, 1083 cm^{-1}.

N-Methoxy-N-methyl-3-((trimethylsilyl)oxy)pent-4-enamide: 2l

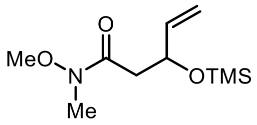

Synthesised according to General Procedure **D** from: Diisopropylamine (1.69 mL, 1.20 equiv.), ^{n}BuLi (1.60 M, 1.10 equiv.), N-methoxy-N-methylacetamide (1.02 mL, 10.0 mmol, 1.00 equiv.), acrolein (0.802 mL, 1.20 equiv.), imidazole (1.06 g, 2.00 equiv. w.r.t alcohol) and trimethylsilyl chloride (1.58 mL, 1.60 equiv. w.r.t alcohol). Purified by flash column chromatography (SiO_2; 4:1 hexane:EtOAc) to afford **2l** (1.58 g, 6.83 mmol, 68% (78% for 1st step)) as a colourless oil.

TLC: R_f = 0.28 (4:1 hexane:EtOAc).

^{1}H NMR (400 MHz, $CDCl_3$) δ 5.89 (ddd, *J* = 17.2, 10.4, 5.5 Hz, 1H, *H*C=CH_2), 5.25 (app-dt, *J* = 17.2, 1.6 Hz, 1H, HC=CH_2), 5.07 (app-dt, *J* = 10.4, 1.6 Hz, 1H, HC=CH_2), 4.79 – 4.61 (m, 1H, C*H*OSi), 3.69 (s, 3H, OCH_3), 3.18 (s, 3H, NCH_3), 2.81 (dd, *J* = 14.6, 8.1 Hz, 1H, CH_2), 2.45 (dd, *J* = 14.6, 5.1 Hz, 1H, CH_2), 0.11 (s, 9H, Si(CH_3)$_3$) ppm. **^{13}C NMR** (126 MHz, $CDCl_3$) δ 171.9 (*C*=O), 140.7 (H*C*=CH_2), 114.3 (HC=*C*H_2), 70.6 (*C*HOSi), 61.5 (O*C*H_3), 40.5 (*C*H_2), 32.1 (N*C*H_3), 0.2 (Si(*C*H_3)$_3$) ppm. (*see spectra*)

HRMS (m/z): (ESI) calc'd for $C_{10}H_{21}NNaO_3Si$ $[M+Na]^+$: 254.1183, found: 254.1188.

IR (thin film) ν_{max}: 2958, 2900, 1660 (C=O), 1417, 1384, 1250 cm^{-1}.

Ethyl 4-chloro-3-((trimethylsilyl)oxy)butanoate

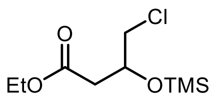

Synthesised according to General Procedure **A** from: Ethyl 4-chloro-3-hydroxybutanoate (1.67 mL, 10.0 mmol, 1.00 equiv.), imidazole (1.36 g, 2.00 equiv.) and trimethylsilyl chloride (2.03 mL, 1.60 equiv.) to afford the desired crude product as a colourless oil which was used without further purification.

4-Chloro-N-methoxy-N-methyl-3-((trimethylsilyl)oxy)butanamide: 2m

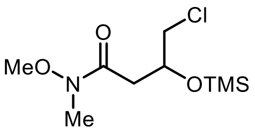

Synthesised according to General Procedure **B** from: Crude **ethyl 4-chloro-3-((trimethylsilyl)oxy)butanoate** (2.30 g, 10.0 mmol, 1.00 equiv.), N,O-dimethylhydroxylamine hydrochloride (1.46 g, 1.50 equiv.) and isopropylmagnesium chloride (2.0 M, 15.0 mL, 3.00 equiv.). Purified by flash column chromatography (SiO_2; 4:1 hexane:EtOAc) to afford **2m** (1.13 g, 4.45 mmol, 45% (over 2 steps)) as a pale-yellow oil.

TLC: R_f = 0.22 (4:1 hexane:EtOAc).

^{1}H NMR (400 MHz, $CDCl_3$) δ 4.42 (app-dq, *J* = 7.2, 5.2 Hz, 1H, C*H*OSi), 3.70 (s, 3H, OCH_3), 3.56 (d, *J* = 5.2 Hz, 2H, CH_2Cl), 3.19 (s, 3H, NCH_3), 2.76 (dd, *J* = 15.5, 7.2 Hz, 1H, CH_2), 2.67 (dd, *J* = 15.5, 5.2 Hz, 1H, CH_2), 0.14 (s, 9H, Si(CH_3)$_3$) ppm. **^{13}C NMR** (126 MHz, $CDCl_3$) δ 171.5 (*C*=O), 69.4 (*C*HOSi), 61.5 (O*C*H_3), 49.2 (*C*H_2Cl), 37.6 (*C*H_2), 32.2 (N*C*H_3), 0.3 (Si(*C*H_3)$_3$) ppm. (*see spectra*)

HRMS (m/z): (ESI) calc'd for $C_{19}H_{20}ClNNaO_3Si$ $[M+Na]^+$: 276.0793, found: 276.0801.

IR (thin film) ν_{max}: 2957, 1661 (C=O), 1419, 1387, 1252, 1093 cm^{-1}.

4-(Benzyloxy)-N-methoxy-N-methyl-3-((trimethylsilyl)oxy)butanamide: 2n

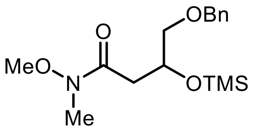

Synthesised according to General Procedure **D** from: Diisopropylamine (1.69 mL, 1.20 equiv.), ^{n}BuLi (1.60 M, 1.10 equiv.), N-methoxy-N-methylacetamide (1.02 mL, 10.0 mmol, 1.00 equiv.), 2-(benzyloxy)acetaldehyde (1.69 mL, 1.20 equiv.), imidazole (708 mg, 2.00 equiv. w.r.t alcohol) and trimethylsilyl chloride (1.06 mL, 1.60 equiv. w.r.t alcohol). Purified by flash column

chromatography (SiO_2; 4:1 hexane:EtOAc) to afford **2n** (1.47 g, 4.52 mmol, 45% (52% for 1st step)) as a colourless oil.

TLC: R_f = 0.27 (4:1 hexane:EtOAc).

^{1}H NMR (400 MHz, $CDCl_3$) δ 7.37 – 7.27 (m, 5H, ArC*H*), 4.55 (s, 2H, OCH_2Ph), 4.41 (app-dq, *J* = 7.6, 5.2 Hz, 1H, C*H*OSi), 3.68 (s, 3H, OCH_3), 3.50 – 3.40 (m, 2H, CHCH_2O), 3.17 (s, 3H, NCH_3), 2.72 (dd, *J* = 14.8, 7.6 Hz, 1H, (C=O)CH_2), 2.59 (dd, *J* = 14.8, 5.2 Hz, 1H, (C=O)CH_2), 0.11 (s, 9H, Si(CH_3)$_3$) ppm. **^{13}C NMR** (126 MHz, $CDCl_3$) δ 172.3 (*C*=O), 138.5 (Ar*C*), 128.4 (Ar*C*H), 127.7 (Ar*C*H), 127.7 (Ar*C*H), 74.4 (CH*C*H_2O), 73.4 (O*C*H_2Ph), 68.7 (*C*HOSi), 61.5 (O*C*H_3), 37.2 ((C=O)*C*H_2), 32.2 (N*C*H_3), 0.3 (Si(*C*H_3)$_3$) ppm. (*see spectra*)

HRMS (m/z): (ESI) calc'd for $C_{16}H_{27}NNaO_4Si$ $[M+Na]^+$: 348.160156, found: 348.160830.

IR (thin film) ν_{max}: 2954, 2858, 1658 (C=O), 1385, 1248, 1096 cm^{-1}.

N-Methoxy-N,3-dimethyl-3-((trimethylsilyl)oxy)butanamide: 2o

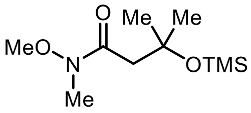

Synthesised according to General Procedure **C** from: 3-Hydroxy-3-methylbutanoic acid (2.20 mL, 17.5 mmol, 1.00 equiv.), N,O-dimethylhydroxylamine hydrochloride (1.76 g, 1.03 equiv.), N-methylmorpholine (1.98 mL, 1.03 equiv.) 1-(3-dimethylaminopropyl)-3-ethylcarbodiimide hydrochloride (EDCI·HCl) (3.46 g, 1.03 equiv.), imidazole (2.38 g, 2.00 equiv.) and trimethylsilyl chloride (3.55 mL, 1.60 equiv.). Purified by flash column chromatography (SiO_2; 4.7:1 hexane:EtOAc) to afford **2o** (2.77 g, 11.9 mmol, 68% (over 2 steps)) as a colourless oil.

TLC: R_f = 0.31 (4.7:1 hexane:EtOAc).

^{1}H NMR (400 MHz, $CDCl_3$) δ 3.69 (s, 3H, OCH_3), 3.18 (s, 3H, NCH_3), 2.61 (s, 2H, CH_2), 1.40 (s, 6H, C(CH_3)$_2$), 0.11 (s, 9H, Si(CH_3)$_3$) ppm. **^{13}C NMR** (126 MHz, $CDCl_3$) δ 172.2 (*C*=O), 73.9 (*C*(CH$_3$)$_2$), 61.2 (O*C*H$_3$), 45.4 ((C=O)*C*H$_2$), 32.1 (N*C*H$_3$), 30.4 (C(*C*H$_3$)$_2$), 2.7 (Si(*C*H$_3$)$_3$) ppm. (*see spectra*)

HRMS (m/z): (ESI) calc'd for $C_{10}H_{23}NNaO_3Si$ $[M+Na]^+$: 256.1339, found: 256.1349.

IR (thin film) ν_{max}: 2964, 2901, 1663 (C=O), 1366, 1249, 1156, 1039 cm^{-1}.

N-Methoxy-N-methyl-2-(1-((trimethylsilyl)oxy)cyclohexyl)acetamide: 2p

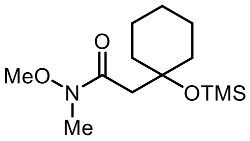

Synthesised according to General Procedure **D** from: Diisopropylamine (1.69 mL, 1.20 equiv.), ^{n}BuLi (1.60 M, 1.10 equiv.), N-methoxy-N-methylacetamide (1.02 mL, 10.0 mmol, 1.00 equiv.), cyclohexanone (1.24 mL, 1.20 equiv.), imidazole (1.27 g, 2.00 equiv. w.r.t alcohol) and trimethylsilyl chloride (1.89 mL, 1.60 equiv. w.r.t alcohol). Purified by flash column chromatography (SiO_2; 5.7:1 hexane:EtOAc) to afford **2p** (2.29 g, 8.37 mmol, 84% (93% for 1st step)) as a colourless oil.

TLC: R_f = 0.20 (5.7:1 hexane:EtOAc).

^{1}H NMR (400 MHz, $CDCl_3$) δ 3.70 (s, 3H, OC*H*$_3$), 3.19 (s, 3H, NC*H*$_3$), 2.66 (s, 2H, *C*H$_2$), 1.87 – 1.74 (m, 2H, cy-C*H*$_2$), 1.71 – 1.51 (m, 4H, cy-C*H*$_2$), 1.51 – 1.26 (m, 4H, cy-C*H*$_2$), 0.14 (s, 9H, Si(C*H*$_3$)$_3$) ppm. **^{13}C NMR** (126 MHz, $CDCl_3$) δ 172.2 (*C*=O), 75.8 (cy-*C*OSi), 61.1 (O*C*H$_3$), 42.6 (*C*H$_2$), 38.5 (cy-*C*H$_2$), 32.1 (N*C*H$_3$), 25.7 (cy-*C*H$_2$), 22.9 (cy-*C*H$_2$), 2.8 (Si(*C*H$_3$)$_3$) ppm. (*see spectra*)

All characterization data are consistent with that reported in the literature.[11]

***syn*-Ethyl 2-methyl-3-((trimethylsilyl)oxy)pent-4-enoate**

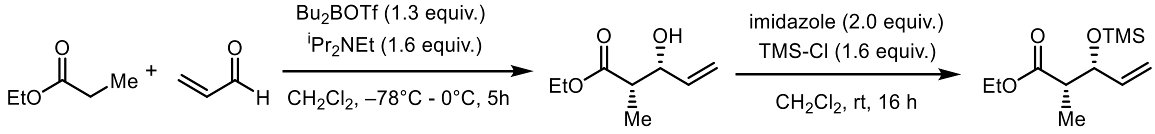

Following a modified literature procedure.[12]

A 1.0 M solution of Bu_2BOTf in CH_2Cl_2 (13.0 mL, 1.30 equiv.) was added at a rate of 1.0 mL/min[**A**] to a solution of ethyl propionate (1.15 mL, 10.0 mmol, 1.00 equiv.) in CH_2Cl_2 (30 mL, 0.33 M) at −78 °C. $^{i}Pr_2NEt$ (2.79 mL, 1.60 equiv.) was then added at a rate of 0.25 mL/min and the reaction stirred at −78 °C for 2 h. After this time, acrolein (0.802 mL, 1.20 equiv.) was added at a rate of 0.10 mL/min. The reaction was stirred for 1 h at −78 °C, then warmed to 0 °C over 2 h. After this time, the reaction was quenched at −78 °C with pH 7.4 phosphate buffer solution (20 mL) and MeOH (40 mL). To this was slowly added a 2:1 mixture of MeOH/30% aq. H_2O_2 (60 mL) at 0 °C. After warming to rt, the solution was diluted with H_2O and the organic phase was separated. The aqueous phase was extracted with Et_2O (3×) and the combined organic phases were washed with sat. aq. $NaHCO_3$, dried ($MgSO_4$) and concentrated under reduced pressure.[**B**] The resulting residue was then dissolved in CH_2Cl_2 (19 mL, 0.4 M) followed by the addition of imidazole (1.03 g, 2.00 equiv. w.r.t alcohol) and trimethylsilyl

chloride (1.54 mL, 1.60 equiv. w.r.t alcohol). The mixture was stirred at rt for 16 h then poured into H_2O. The solution was diluted with CH_2Cl_2, the organic layer separated, and the aqueous phase was extracted with CH_2Cl_2 (2×). The combined organic phases were dried ($MgSO_4$) and concentrated under reduced pressure. The residue was purified by flash column chromatography (SiO_2; 24:1 hexane:EtOAc) to afford the desired product as the *syn*-diastereomer 94:6 dr (1.04 g, 4.51 mmol, 59% (76% for 1st step)) as a colourless oil.

Notes: (**A**) All reagents were added *via* syringe pump at the given rate. (**B**) At this point, the yield of the alcohol product was determined by ^{1}H NMR analysis using dibromomethane as an internal standard **TLC**: R_f = 0.31 (24:1 hexane:EtOAc).

^{1}H NMR (400 MHz, $CDCl_3$) *syn*-diastereomer δ 5.81 (ddd, *J* = 17.2, 10.4, 6.2 Hz, 1H, *H*C=CH_2), 5.19 (app-dt, *J* = 17.2, 1.6 Hz, 1H, HC=C*H*$_2$), 5.09 (ddd, *J* = 10.4, 1.6, 1.3 Hz, 1H, HC=C*H*$_2$), 4.35 (app-tt, *J* = 6.2, 1.3 Hz, 1H, C*H*OSi), 4.11 (app-qd, *J* = 7.3, 3.7 Hz, 2H, OC*H*$_2$$CH_3$), 2.54 – 2.41 (m, 1H, C*H*CH_3), 1.25 (t, *J* = 7.3 Hz, 3H, OCH_2C*H*$_3$), 1.14 (d, *J* = 7.1 Hz, 3H, CHC*H*$_3$), 0.09 (s, 9H, Si(C*H*$_3$)$_3$) ppm. **^{13}C NMR** (126 MHz, $CDCl_3$) *syn*-diastereomer δ 174.6 (*C*=O), 139.5 (H*C*=CH_2), 115.5 (HC=*C*H_2), 75.1 (*C*HOSi), 60.4 (O*C*$H_2$$CH_3$), 46.8 (*C*H$CH_3$), 14.4 (O$CH_2$*C*$H_3$), 12.3 (CHC*H*$_3$), 0.3 (Si(*C*$H_3$)$_3$) ppm. (*see spectra*)

HRMS (m/z): (ESI) calc'd for $C_{11}H_{22}NNaO_3Si$ $[M+Na]^+$: 253.123042, found: 253.122078.

IR (thin film) ν_{max}: 2965, 1729 (C=O), 1253, 1083, 907 cm^{-1}.

syn-N-Methoxy-N,2-dimethyl-3-((trimethylsilyl)oxy)pent-4-enamide: _syn_-2q

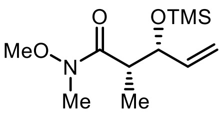

Synthesised according to General Procedure **B** from: *syn*-**ethyl 2-methyl-3-((trimethylsilyl)oxy)pent-4-enoate** (dr 94:6, 606 mg, 2.63 mmol, 1.00 equiv.), N,O-dimethylhydroxylamine hydrochloride (385 mg, 1.50 equiv.) and isopropylmagnesium chloride (2.0 M, 3.94 mL, 3.00 equiv.). Purified by flash column chromatography (SiO_2; 4:1 hexane:EtOAc) to afford **2q** as the *syn*-diastereomer 97:3 dr[**A**] (490 mg, 2.00 mmol, 76%) as a colourless oil.

Notes: (**A**) Analysis of the crude reaction mixture determined the dr to be 94:6. *anti*-diastereomer was partially removed during column chromatography.

TLC: R_f = 0.30 (4:1 hexane:EtOAc).

^{1}**H NMR** (400 MHz, $CDCl_3$) *syn*-diastereomer δ 5.84 (ddd, *J* = 17.2, 10.4, 6.1 Hz, 1H, *H*C=CH_2), 5.20 (ddd, *J* = 17.2, 1.8, 1.2 Hz, 1H, HC=CH_2), 5.06 (ddd, *J* = 10.4, 1.8, 1.2 Hz, 1H, HC=CH_2), 4.23 (app-ddt, *J* = 8.8, 6.1, 1.2 Hz, 1H, C*H*OSi), 3.65 (s, 3H, OCH_3), 3.15 (s, 3H, NCH_3), 2.98 (br. s, 1H, C*H*CH_3), 1.19 (d, *J* = 6.9 Hz, 3H, CHCH_3), 0.13 (s, 9H, Si(CH_3)$_3$) ppm. ^{13}**C NMR** (126 MHz, $CDCl_3$) *syn*-diastereomer δ 175.9 (*C*=O), 139.7 (H*C*=CH_2), 115.3 (HC=*C*H_2), 75.7 (*C*HOSi), 61.7 (O*C*H_3), 42.6 (*C*HCH_3), 32.2 (N*C*H_3), 14.9 (CH*C*H_3), 0.4 (Si(*C*H_3)$_3$) ppm. (see spectra)

HRMS (m/z): (ESI) calc'd for $C_{11}H_{23}NNaO_3Si$ $[M+Na]^+$: 268.133941, found: 268.133818.

IR (thin film) ν_{max}: 2963, 1662 (C=O), 1461, 1419, 1385, 1252, 1074 cm^{-1}.

anti-Ethyl 2-methyl-3-((trimethylsilyl)oxy)pent-4-enoate

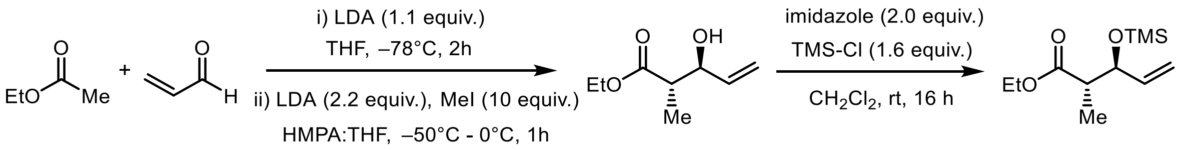

Following a modified literature procedure.[13]

^{n}BuLi (1.6 M, 10.3 mL, 1.10 equiv.) was added dropwise to a solution of diisopropylamine (2.64 mL, 1.20 equiv.) in THF (45 mL, 0.33 M) at −78 °C and stirred for 10 minutes. Ethyl acetate (1.47 mL, 15.0 mmol, 1.00 equiv.) in THF (15 mL, 1.0 M) was added slowly and the solution was stirred at −78 °C for 1 h. Acrolein (1.20 mL, 1.20 equiv.) in THF (18 mL, 1.0 M) was then added dropwise before the solution was stirred at −78 °C for a further 1 h. After this time, the reaction was quenched with sat. aq. NH_4Cl, diluted with H_2O and the organic phase was separated. The aqueous phase was extracted with Et_2O (3×) and the combined organic phases were washed with sat. aq. $NaHCO_3$, dried ($MgSO_4$) and concentrated under reduced pressure. The residue was then filtered through a short plug of silica gel eluting with EtOAc and the solvent removed under reduced pressure.[A]

The crude alcohol (11.4 mmol, 1.00 equiv.) was redissolved in THF (2.28 mL, 5.0 M) then added dropwise to a solution of LDA (2.20 equiv.) as prepared previously at −50 °C. The reaction was warmed to −20 °C and stirred for 30 min before the dropwise addition of a solution of MeI (7.10 mL, 10.0 equiv.) in HMPA (2.28 mL, 5.0 M). The reaction was allowed to warm to 0 °C and immediately quenched with sat. aq. NH_4Cl. The solution was then diluted with H_2O, and the organic phase was separated. The aqueous phase was extracted with Et_2O (3×)

and the combined organic phases were washed with sat. aq. $NaHCO_3$, dried ($MgSO_4$) and concentrated under reduced pressure.[A]

The resulting residue was then dissolved in CH_2Cl_2 (22.8 mL, 0.4 M) followed by the addition of imidazole (1.24 g, 2.00 equiv. w.r.t alcohol) and trimethylsilyl chloride (1.85 mL, 1.60 equiv. w.r.t alcohol). The mixture was stirred at rt for 16 h then poured into H_2O. The solution was diluted with CH_2Cl_2, the organic layer separated, and the aqueous phase was then extracted with CH_2Cl_2 (2×). The combined organic phases were dried ($MgSO_4$) and concentrated under reduced pressure. The residue was purified by flash column chromatography (SiO_2; 32:1 hexane:EtOAc) to afford the desired product as the *anti*-diastereomer 94:6 dr (1.81 g, 7.86 mmol, 52% (over 3 steps)) as a colourless oil.

Notes: (**A**) At this point, the yield of the alcohol product was determined by ^{1}H NMR analysis using dibromomethane as an internal standard.

TLC: R_f = 0.32 (32:1 hexane:EtOAc).

^{1}H NMR (400 MHz, $CDCl_3$) *anti*-diastereomer δ 5.74 (ddd, *J* = 17.2, 10.3, 7.2 Hz, 1H, *H*C=CH$_2$), 5.20 (ddd, *J* = 17.2, 1.8, 1.0 Hz, 1H, HC=C*H*$_2$), 5.16 (ddd, *J* = 10.3, 1.8, 1.0 Hz, 1H, HC=C*H*$_2$), 4.31 – 4.04 (m, 3H, C*H*OSi, OC*H*$_2$CH$_3$), 2.51 (dq, *J* = 8.6, 7.0 Hz, 1H, C*H*CH$_3$), 1.27 (t, *J* = 7.1 Hz, 3H, OCH$_2$C*H*$_3$), 1.04 (d, *J* = 7.0 Hz, 3H, CHC*H*$_3$), 0.08 (s, 9H, Si(C*H*$_3$)$_3$) ppm. **^{13}C NMR** (126 MHz, $CDCl_3$) *anti*-diastereomer δ 175.2 (*C*=O), 138.7 (H*C*=CH$_2$), 116.9 (HC=*C*H$_2$), 76.6 (*C*HOSi), 60.4 (O*C*H$_2$CH$_3$), 47.1 (*C*HCH$_3$), 14.5 (OCH$_2$*C*H$_3$), 13.3 (CHC*H*$_3$), 0.3 (Si(*C*H$_3$)$_3$) ppm. (see spectra)

***anti*-N-Methoxy-N,2-dimethyl-3-((trimethylsilyl)oxy)pent-4-enamide: *anti*-2q**

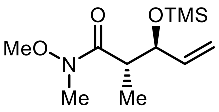

Synthesised according to General Procedure **B** from: *anti*-**ethyl 2-methyl-3-((trimethylsilyl)oxy)pent-4-enoate** (dr 94:6, 979 mg, 4.25 mmol, 1.00 equiv.), N,O-dimethylhydroxylamine hydrochloride (622 mg, 1.50 equiv.) and isopropylmagnesium chloride (2.0 M, 6.28 mL, 3.00 equiv.). Purified by flash column chromatography (SiO_2; 4:1 hexane:EtOAc) to afford **2q** as the *anti*-diastereomer >98:2 dr[A] (649 mg, 2.64 mmol, 62%) as a colourless oil.

Notes: (**A**) Analysis of the crude reaction mixture determined the dr to be 94:6. *syn*-diastereomer was partially removed during column chromatography.

TLC: R_f = 0.21 (5.7:1 hexane:EtOAc).

^{1}H NMR (400 MHz, $CDCl_3$) *anti*-diastereomer δ 5.78 (ddd, *J* = 17.2, 10.2, 7.3 Hz, 1H, *H*C=CH_2), 5.22 (ddd, *J* = 17.2, 1.8, 1.0 Hz, 1H, HC=CH_2), 5.15 (ddd, *J* = 10.2, 1.8, 1.0 Hz, 1H, HC=CH_2), 4.22 – 4.26 (m, 1H, C*H*OSi), 3.72 (s, 3H, OCH_3), 3.20 (s, 3H, NCH_3), 3.03 (br. s, 1H, C*H*CH_3), 0.99 (d, *J* = 6.9 Hz, 3H, CHCH_3), 0.06 (s, 9H, Si(CH_3)$_3$) ppm. **^{13}C NMR** (126 MHz, $CDCl_3$) *anti*-diastereomer δ 176.2 (*C*=O), 139.3 (H*C*=CH_2), 116.6 (HC=*C*H_2), 76.7 (*C*HOSi), 61.6 (O*C*H_3), 41.8 (*C*HCH_3), 32.1 (N*C*H_3), 13.9 (CHC*H*$_3$), 0.4 (Si(*C*H_3)$_3$) ppm. (*see spectra*)

N-Methoxy-N-methyl-4-((trimethylsilyl)oxy)pentanamide: 2r

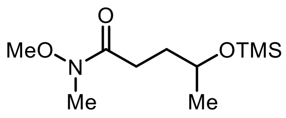

Synthesised according to General Procedure **E** from: γ-Valerolactone (0.954 mL, 10.0 mmol, 1.00 equiv.), N,O-dimethylhydroxylamine hydrochloride (2.44 g, 2.50 equiv.), isopropylmagnesium chloride (2.0 M, 20.0 mL, 4.00 equiv.), imidazole (1.36 g, 2.00 equiv.) and trimethylsilyl chloride (2.03 mL, 1.60 equiv.). Purified by flash column chromatography (SiO_2; 4:1 hexane:EtOAc) to afford **2r** (1.67 g, 7.16 mmol, 72% (over 2 steps)) as a colourless oil.

TLC: R_f = 0.18 (4:1 hexane:EtOAc).

^{1}H NMR (400 MHz, $CDCl_3$) δ 3.87 (dqd, *J* = 7.7, 6.1, 4.3 Hz, 1H, C*H*CH_3), 3.69 (s, 3H, OCH_3), 3.17 (s, 3H, NCH_3), 2.60 – 2.36 (m, 2H, (C=O)CH_2), 1.88 – 1.72 (m, 1H, CH_2CH), 1.74 – 1.61 (m, 1H, CH_2CH), 1.17 (d, *J* = 6.1 Hz, 3H, CHCH_3), 0.11 (s, 9H, Si(CH_3)$_3$) ppm. **^{13}C NMR** (126 MHz, $CDCl_3$) δ 174.8 (*C*=O), 67.9 (*C*HCH_3), 61.3 (O*C*H_3), 34.0 (*C*H_2CH), 32.4 (N*C*H_3), 28.2 ((C=O)*C*H_2), 24.0 (CH*C*H_3), 0.4 (Si(*C*H_3)$_3$) ppm. (*see spectra*)

HRMS (m/z): (ESI) calc'd for $C_{10}H_{23}NNaO_3Si$ $[M+Na]^+$: 256.133941, found: 256.134678.

IR (thin film) ν_{max}: 2955, 1669 (C=O), 1380, 1249, 1139, 1079, 993 cm^{-1}.

N-Methoxy-N-methyl-5-((trimethylsilyl)oxy)pentanamide: 2s

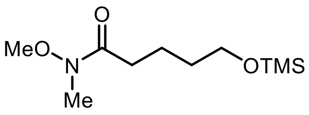

Synthesised according to General Procedure **E** from: δ-Valerolactone (0.928 mL, 10.0 mmol, 1.00 equiv.), N,O-dimethylhydroxylamine hydrochloride (2.44 g, 2.50 equiv.), isopropylmagnesium chloride (2.0 M, 20.0 mL, 4.00 equiv.), imidazole (1.36 g, 2.00 equiv.)

and trimethylsilyl chloride (2.03 mL, 1.60 equiv.). Purified by flash column chromatography (SiO_2; 4:1 hexane:EtOAc) to afford **2s** (1.29 g, 5.53 mmol, 55% (over 2 steps)) as a colourless oil.

TLC: R_f = 0.23 (4:1 hexane:EtOAc).

^{1}H NMR (400 MHz, $CDCl_3$) δ 3.68 (s, 3H, OC*H*$_3$), 3.60 (t, *J* = 6.4 Hz, 2H, C*H*$_2$OSi), 3.18 (s, 3H, NC*H*$_3$), 2.45 (t, *J* = 7.5 Hz, 2H, (C=O)C*H*$_2$), 1.73 – 1.55 (m, 4H, C*H*$_2$C*H*$_2$CH$_2$OSi), 0.11 (s, 9H, Si(C*H*$_3$)$_3$) ppm. **^{13}C NMR** (126 MHz, $CDCl_3$) δ 174.7 (*C*=O), 62.5 (*C*H$_2$OSi), 61.4 (O*C*H$_3$), 32.5 (CH$_2$*C*H$_2$CH$_2$OSi), 32.3 (N*C*H$_3$), 31.8 ((C=O)*C*H$_2$), 21.3 (*C*H$_2$CH$_2$CH$_2$OSi), −0.3 (Si(*C*H$_3$)$_3$) ppm. (*see spectra*)

HRMS (m/z): (ESI) calc'd for $C_{10}H_{23}NNaO_3Si$ $[M+Na]^+$: 256.133941, found: 256.134313.

IR (thin film) ν_{max}: 2953, 1664 (C=O), 1417, 1384, 1250, 1098, 999 cm^{-1}.

3.4 Synthesis of protected aromatic alcohol carboxylate esters: 2t-2v

Ethyl 2-((*tert*-butyldimethylsilyl)oxy)benzoate: 2t

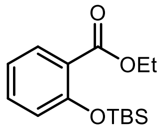

Synthesised according to General Procedure **A** from: Ethyl salicylate (1.47 mL, 10.0 mmol, 1.00 equiv.), imidazole (1.36 g, 2.00 equiv.) and *tert*-butyldimethylsilyl chloride (1.96 g, 1.30 equiv.). Purified by flash column chromatography (SiO_2; 9:1 pentane:CH_2Cl_2) to afford **2t** (2.16 g, 7.70 mmol, 77%) as a colourless oil.

TLC: R_f = 0.33 (9:1 pentane:CH_2Cl_2).

^{1}H NMR (400 MHz, $CDCl_3$) δ 7.72 (dd, *J* = 7.6, 1.9 Hz, 1H, ArC*H*), 7.34 (ddd, *J* = 8.2, 7.6, 1.9 Hz, 1H, ArC*H*), 6.97 (app-td, *J* = 7.6, 1.1 Hz, 1H, ArC*H*), 6.87 (dd, *J* = 8.2, 1.1 Hz, 1H, ArC*H*), 4.34 (q, *J* = 7.1 Hz, 2H, OC*H*$_2$CH$_3$), 1.37 (t, *J* = 7.1 Hz, 3H, OCH$_2$C*H*$_3$), 1.01 (s, 9H, SiC(C*H*$_3$)$_3$), 0.22 (s, 6H, Si(C*H*$_3$)$_2$) ppm. **^{13}C NMR** (126 MHz, $CDCl_3$) δ 167.1 (*C*=O), 155.2 (Ar*C*), 133.0 (Ar*C*H), 131.6 (Ar*C*H), 123.7 (Ar*C*), 121.3 (Ar*C*H), 121.1 (Ar*C*H), 61.0 (O*C*H$_2$CH$_3$), 26.0 (SiC(*C*H$_3$)$_3$), 18.6 (Si*C*(CH$_3$)$_3$), 14.5 (OCH$_2$*C*H$_3$), −4.0 (Si(*C*H$_3$)$_2$) ppm. (*see spectra*)

HRMS (m/z): (ESI) calc'd for $C_{15}H_{24}NaO_3Si$ $[M+Na]^+$: 303.1387, found: 303.1394.

IR (thin film) ν$_{max}$: 2956, 2931, 2859, 1729 (C=O), 1486, 1238, 1078 cm^{-1}.

Methyl 3-((*tert*-butyldimethylsilyl)oxy)picolinate: 2u

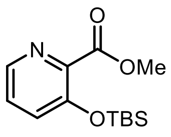

Synthesised according to General Procedure **A** from: Methyl 3-hydroxypicolinate (2.30 g, 15.0 mmol, 1.00 equiv.), imidazole (2.04 g, 2.00 equiv.) and *tert*-butyldimethylsilyl chloride (3.62 g, 1.60 equiv.). Purified by flash column chromatography (SiO_2; 2.3:1 hexane:EtOAc) to afford **2u** (3.18 g, 11.9 mmol, 79%) as a colourless oil.

TLC: R_f = 0.31 (19:1 CH_2Cl_2:EtOAc).

^{1}H NMR (400 MHz, $CDCl_3$) δ 8.30 (dd, *J* = 4.4, 1.4 Hz, 1H, ArC*H*), 7.32 (dd, *J* = 8.4, 4.4 Hz, 1H, ArC*H*), 7.24 (dd, *J* = 8.4, 1.4 Hz, 1H, ArC*H*), 3.94 (s, 3H, OCH_3), 1.01 (s, 9H, SiC(CH_3)$_3$), 0.23 (s, 6H, Si(CH_3)) ppm. **^{13}C NMR** (126 MHz, $CDCl_3$) δ 165.6 (*C*=O), 152.0 (Ar*C*), 142.2 (Ar*C*H), 141.5 (Ar*C*), 128.9 (Ar*C*H), 126.9 (Ar*C*H), 52.6 (O*C*H_3), 25.7 (SiC(*C*H_3)$_3$), 18.4 (Si*C*(CH_3)$_3$), −4.3 (Si(*C*H_3)$_2$) ppm. (*see spectra*)

HRMS (m/z): (ESI) calc'd for $C_{13}H_{21}NNaO_3Si$ $[M+Na]^+$: 290.1183, found: 290.1193.

IR (thin film) ν$_{max}$: 2952, 2932, 2860, 1736 (C=O), 1578, 1450, 1305, 1254, 1096 cm^{-1}.

3-((*tert*-Butyldimethylsilyl)oxy)picolinaldehyde: 2u′

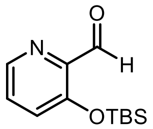

Synthesised according to General Procedure **A** from: 3-Hydroxypicolinaldehyde (616 mg, 5.00 mmol, 1.00 equiv.), imidazole (681 mg, 2.00 equiv.) and *tert*-butyldimethylsilyl chloride (1.21 g, 1.60 equiv.). Purified by flash column chromatography (SiO_2; 19:1 CH_2Cl_2:EtOAc) to afford **2u′** (812 mg, 3.42 mmol, 68%) as a pale-yellow oil.

TLC: R_f = 0.21 (19:1 CH_2Cl_2:EtOAc).

^{1}H NMR (400 MHz, $CDCl_3$) δ 10.36 (s, 1H, (C=O)*H*), 8.43 (dd, *J* = 4.4, 1.3 Hz, 1H, ArC*H*), 7.39 (dd, *J* = 8.4, 4.4 Hz, 1H, ArC*H*), 7.29 (dd, *J* = 8.4, 1.3 Hz, 1H, ArC*H*), 1.04 (s, 9H, SiC(CH_3)$_3$), 0.28 (s, 6H, Si(CH_3)$_2$) ppm. **^{13}C NMR** (126 MHz, $CDCl_3$) δ 190.2 (*C*=O), 155.1 (Ar*C*), 143.6 (Ar*C*), 143.4 (Ar*C*H), 129.2 (Ar*C*H), 128.6 (Ar*C*H), 25.7 (SiC(*C*H_3)$_3$), 18.5 (Si*C*(CH_3)$_3$), −4.2 (Si(*C*H_3)$_2$) ppm. (*see spectra*)

HRMS (m/z): (ESI) calc'd for $C_{12}H_{19}NNaO_2Si$ $[M+Na]^+$: 260.107726, found: 260.108318.

IR (thin film) ν_{max}: 2955, 2932, 2860, 2808, 1716 (C=O), 1578, 1457, 1300, 922 cm^{-1}.

Methyl 3-((*tert*-butyldimethylsilyl)oxy)-2-naphthoate: 2v

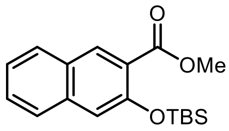

Synthesised according to General Procedure **A** from: Methyl 3-hydroxy-2-naphthoate (2.02 g, 10.0 mmol, 1.00 equiv.), imidazole (1.36 g, 2.00 equiv.) and *tert*-butyldimethylsilyl chloride (2.41 g, 1.60 equiv.). Purified by flash column chromatography (SiO_2; 2.3:1 pentane:CH_2Cl_2) to afford **2v** (2.74 g, 8.66 mmol, 87%) as a colourless oil.

TLC: R_f = 0.22 (2.3:1 pentane:CH_2Cl_2).

^{1}H NMR (400 MHz, $CDCl_3$) δ 8.28 (s, 1H, ArC*H*), 7.81 (d, *J* = 8.2 Hz, 1H, ArC*H*), 7.68 (d, *J* = 8.2 Hz, 1H, ArC*H*), 7.49 (ddd, *J* = 8.2, 6.9, 1.3 Hz, 1H, ArC*H*), 7.37 (ddd, *J* = 8.2, 6.9, 1.3 Hz, 1H, ArC*H*), 7.20 (s, 1H, ArC*H*), 3.93 (s, 3H, OCH_3), 1.05 (s, 9H, SiC(CH_3)$_3$), 0.27 (s, 6H, Si(CH_3)$_2$) ppm. **^{13}C NMR** (126 MHz, $CDCl_3$) δ 167.5 (*C*=O), 151.5 (Ar*C*), 136.1 (Ar*C*), 132.7 (Ar*C*H), 128.8 (Ar*C*H), 128.2 (Ar*C*H), 128.2 (Ar*C*), 126.4 (Ar*C*H), 124.6 (Ar*C*H), 124.6 (Ar*C*), 116.1 (Ar*C*H), 52.3 (OCH_3), 25.9 (SiC(CH_3)$_3$), 18.5 (Si*C*(CH_3)$_3$), −4.2 (Si(CH_3)$_2$) ppm. (*see spectra*)

All characterization data are consistent with that reported in the literature.[14]

3.5 Synthesis of ABB-ketones: 3a-3v

2,3-Dibromopropan-1-amine hydrobromide: 5

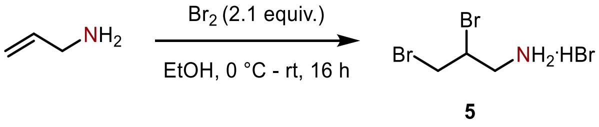

Following literature procedure.[5] **5** is also commercially available (ALD00562, CAS 6963-32-2).

Br_2 (10.0 mL, 0.196 mol, 2.10 equiv.) was added dropwise at a rate of 0.25 mL/min *via* syringe pump to a rapidly stirring flask containing EtOH (25 mL) at 0 °C. After the addition was complete, allylamine (7.00 mL, 0.09 mol, 1.00 equiv.) was added dropwise at a rate of 0.25 mL/min *via* syringe pump under vigorous stirring at 0 °C. The mixture was warmed to rt and stirred for 16 h. The white precipitate formed was collected *via* vacuum filtration and washed with Et_2O at 0 °C. The crude material was then dissolved in the minimum amount of MeOH (~40 mL) and Et_2O was added dropwise until a white precipitate formed. After complete precipitation, ammonium salt **5** (18.0 g, 0.129 mol, 66%) was collected as white crystals *via* vacuum filtration.

^{1}H NMR (400 MHz, CD_3OD) δ 4.54 (dddd, J = 9.5, 8.5, 4.7, 3.2 Hz, 1H, C*H*Br), 4.02 (dd, J = 11.0, 4.7 Hz, 1H, CH_2Br), 3.87 (dd, J = 11.0, 8.5 Hz, 1H, CH_2Br), 3.71 (dd, J = 14.0, 3.2 Hz, 1H, C$H_2$$NH_2$), 3.39 (dd, J = 14.0, 9.5 Hz, 1H, C$H_2$$NH_2$) ppm. **^{13}C NMR** (101 MHz, CD_3OD) δ 48.0 (*C*HBr), 45.6 (*C*$H_2$$NH_2$), 34.1 (*C*$H_2$Br) ppm. (*see spectra*)

All characterization data are consistent with that reported in the literature.[5]

1-(1-Azabicyclo[1.1.0]butan-3-yl)-2-((triethylsilyl)oxy)propan-1-one: 3a

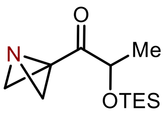

ABB-Li was synthesised according to General Procedure **F** followed by the addition of **2a** (279 mg, 1.20 equiv.) as a solution in anhydrous THF (1 mL). Purified by flash column chromatography (SiO_2; 12:1 hexane:EtOAc) to afford **3a** (150 mg, 0.624 mmol, 62%) as a colourless oil.

TLC: R_f = 0.30 (12:1 hexane:EtOAc).

^{1}H NMR (400 MHz, $CDCl_3$) δ 4.42 (q, *J* = 6.9 Hz, 1H, C*H*(C=O)), 3.12 (dd, *J* = 6.5, 2.8 Hz, 1H, NCH_2), 3.04 (dd, *J* = 6.5, 2.5 Hz, 1H, NCH_2), 1.55 (d, *J* = 2.5 Hz, 1H, NCH_2), 1.51 (d, *J* = 2.8 Hz, 1H, NCH_2), 1.40 (d, *J* = 6.9 Hz, 3H, CHCH_3), 0.95 (t, *J* = 7.9 Hz, 9H, Si(CH_2CH_3)$_3$), 0.61 (q, *J* = 7.9 Hz, 6H, Si(C$H_2$$CH_3$)$_3$) ppm. **^{13}C NMR** (126 MHz, $CDCl_3$) δ 208.2 (*C*=O), 74.2 (*C*HCH_3), 56.0 (N*C*H_2), 55.8 (N*C*H_2), 27.5 (*C*N(CH_2)$_2$), 21.6 (CH*C*H_3), 6.82 (Si(CH_2*C*H_3)$_3$), 4.81 (Si(*C*$H_2$$CH_3$)$_3$) ppm. (*see spectra*)

HRMS (m/z): (ESI) calc'd for $C_{12}H_{23}NNaO_2Si$ $[M+Na]^+$: 264.139026, found: 264.138276.

IR (thin film) ν_{max}: 2955, 2878, 1698 (C=O), 1402, 1133, 1006 cm^{-1}.

1-(1-Azabicyclo[1.1.0]butan-3-yl)-3-phenyl-2-((triethylsilyl)oxy)propan-1-one: 3b

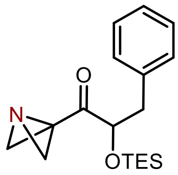

ABB-Li was synthesised according to General Procedure **F** followed by the addition of **2b** (370 mg, 1.20 equiv.) as a solution in anhydrous THF (1 mL). Purified by flash column chromatography (SiO_2; 16:1 hexane:EtOAc) to afford **3b** (160 mg, 0.509 mmol, 51%) as a colourless oil.

TLC: R_f = 0.21 (16:1 hexane:EtOAc).

^{1}H NMR (400 MHz, $CDCl_3$) δ 7.32 – 7.25 (m, 2H, ArC*H*), 7.25 – 7.17 (m, 3H, ArC*H*), 4.43 (dd, *J* = 8.4, 4.6 Hz, 1H, C*H*(C=O)), 3.10 (dd, *J* = 6.5, 2.8 Hz, 1H, NCH_2), 3.04 – 2.96 (m, 2H, NCH_2, CH_2Ph), 2.88 (dd, *J* = 13.6, 8.4 Hz, 1H, CH_2Ph), 1.52 (d, *J* = 2.8 Hz, 1H, NCH_2), 1.47 (d, *J* = 2.8 Hz, 1H, NCH_2), 0.82 (t, *J* = 7.9 Hz, 9H, Si(CH_2CH_3)$_3$), 0.54 – 0.33 (m, 6H, Si(CH_2CH_3)$_3$) ppm. **^{13}C NMR** (101 MHz, $CDCl_3$) δ 207.8 (*C*=O), 136.9 (Ar*C*), 129.9 (Ar*C*H), 128.4 (Ar*C*H), 126.9 (Ar*C*H), 79.6 (*C*H(C=O)), 56.0 (N*C*H_2), 55.8 (N*C*H_2), 42.0 (*C*H_2Ph), 27.8 (*C*N(CH_2)$_2$), 6.68 (Si(CH_2CH_3)$_3$), 4.50 (Si(CH_2CH_3)$_3$) ppm. (*see spectra*)

HRMS (m/z): (ESI) calc'd for $C_{18}H_{27}NNaO_2Si$ $[M+Na]^+$: 340.1703, found: 340.1699.

IR (thin film) ν_{max}: 2953, 2877, 1697 (C=O), 1455, 1097, 1005, 742 cm^{-1}.

1-(1-Azabicyclo[1.1.0]butan-3-yl)-2-methyl-2-((triethylsilyl)oxy)propan-1-one: 3c

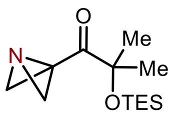

ABB-Li was synthesised according to General Procedure **F** followed by the addition of **2c** (296 mg, 1.20 equiv.) as a solution in anhydrous THF (1 mL). Purified by flash column chromatography (SiO_2; 19:1 hexane:EtOAc) to afford **3c** (93.1 mg, 0.364 mmol, 36%) as a colourless oil.

TLC: R_f = 0.27 (19:1 hexane:EtOAc).

^{1}H NMR (400 MHz, $CDCl_3$) δ 3.07 (dd, *J* = 1.8, 1.1 Hz, 2H, NCH_2), 1.48 (dd, *J* = 1.8, 1.1 Hz, 2H, NCH_2), 1.42 (s, 6H, C(CH_3)$_2$), 0.95 (t, *J* = 7.9 Hz, 9H, Si(CH_2CH_3)$_3$), 0.63 (q, *J* = 7.9 Hz, 6H, Si(CH_2CH_3)$_3$) ppm. **^{13}C NMR** (101 MHz, $CDCl_3$) δ 209.4 (*C*=O), 80.3 (*C*(C=O)), 56.3 (N*C*H_2), 27.7 (C(*C*H_3)$_2$), 27.4 (*C*N(CH_2)$_2$), 7.0 (Si(CH_2CH_3)$_3$), 6.6 (Si(CH_2CH_3)$_3$) ppm. (*see spectra*)

HRMS (m/z): (ESI) calc'd for $C_{13}H_{25}NNaO_2Si$ $[M+Na]^+$: 278.154676, found: 278.155275.

IR (thin film) ν_{max}: 2955, 2878, 1698 (C=O), 1395, 1172, 1009, 728 cm^{-1}.

1-(1-Azabicyclo[1.1.0]butan-3-yl)-2-methyl-3-((triethylsilyl)oxy)propan-1-one: 3d

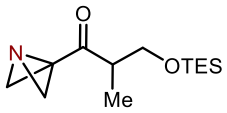

ABB-Li was synthesised according to General Procedure **F** followed by the addition of **2d** (314 mg, 1.20 equiv.) as a solution in anhydrous THF (1 mL). Purified by flash column chromatography (SiO_2; 12:1 hexane:EtOAc) to afford **3d** (99.6 mg, 0.390 mmol, 39%) as a yellow oil.

TLC: R_f = 0.25 (12:1 hexane:EtOAc).

^{1}H NMR (400 MHz, $CDCl_3$) δ 3.81 (dd, *J* = 9.8, 7.2 Hz, 1H, C*H*$_2$OSi), 3.60 (dd, *J* = 9.8, 6.2 Hz, 1H, C*H*$_2$OSi), 3.05 – 2.87 (m, 3H, NC*H*$_2$, C*H*CH$_3$), 1.55 (d, *J* = 2.3 Hz, 1H, NC*H*$_2$), 1.51 (d, *J* = 2.3 Hz, 1H, NC*H*$_2$), 1.08 (d, *J* = 6.9 Hz, 3H, CHC*H*$_3$), 0.92 (t, *J* = 7.9 Hz, 9H, Si(CH$_2$C*H*$_3$)$_3$), 0.57 (q, *J* = 7.9 Hz, 6H, Si(C*H*$_2$CH$_3$)$_3$) ppm. **^{13}C NMR** (101 MHz, $CDCl_3$) δ 209.4 (*C*=O), 65.2 (C*H*$_2$OSi), 55.6 (N*C*H$_2$), 55.3 (N*C*H$_2$), 45.4 (*C*HCH$_3$), 30.2 (*C*N(CH$_2$)$_2$), 13.5 (CH*C*H$_3$), 6.8 (Si(CH$_2$*C*H$_3$)$_3$), 4.4 (Si(*C*H$_2$CH$_3$)$_3$) ppm. (*see spectra*)

HRMS (m/z): (ESI) calc'd for $C_{13}H_{26}NO_2SiNa$ $[M+Na]^+$: 278.154676, found: 278.155555.

IR (thin film) ν_{max}: 2954, 2912, 2877, 1697 (C=O), 1458, 1407, 1240, 1126, 1014 cm^{-1}.

1-(1-Azabicyclo[1.1.0]butan-3-yl)-2-phenyl-3-((triethylsilyl)oxy)propan-1-one: 3e

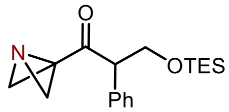

ABB-Li was synthesised according to General Procedure **F** followed by the addition of **2e** (388 mg, 1.20 equiv.) as a solution in anhydrous THF (1 mL). Purified by flash column chromatography (SiO_2; 19:1 hexane:EtOAc) to afford **3e** (215 mg, 0.677 mmol, 68%) as a colourless oil.

TLC: R_f = 0.27 (12.3:1 hexane:EtOAc).

^{1}H NMR (400 MHz, $CDCl_3$) δ 7.36 – 7.25 (m, 3H, ArC*H*), 7.24 – 7.20 (m, 2H, ArC*H*), 4.24 (dd, *J* = 9.8, 7.8 Hz, 1H, C*H*$_2$OSi), 4.11 (dd, *J* = 7.8, 6.0 Hz, 1H, C*H*Ph), 3.74 (dd, *J* = 9.8, 6.0 Hz, 1H, C*H*$_2$OSi), 2.92 – 2.83 (m, 2H, NC*H*$_2$), 1.46 (d, *J* = 2.0 Hz, 1H, NC*H*$_2$), 1.44 (d, *J* = 2.0 Hz, 1H, NC*H*$_2$), 0.87 (t, *J* = 7.9 Hz, 9H, Si(CH$_2$C*H*$_3$)$_3$), 0.59 – 0.43 (m, 6H, Si(C*H*$_2$CH$_3$)$_3$) ppm. **^{13}C NMR** (126 MHz, $CDCl_3$) δ 205.3 (*C*=O), 135.5 (Ar*C*), 129.0 (ArC*H*), 128.8 (ArC*H*), 127.9 (ArC*H*), 64.6 (C*H*$_2$OSi), 57.9 (*C*HPh), 55.8 (N*C*H$_2$), 55.6 (N*C*H$_2$), 30.5 (*C*N(CH$_2$)$_2$), 6.8 (Si(CH$_2$*C*H$_3$)$_3$), 4.4 (Si(*C*H$_2$CH$_3$)$_3$) ppm. (*see spectra*)

HRMS (m/z): (ESI) calc'd for $C_{18}H_{27}NNaO_2Si$ $[M+Na]^+$: 340.1703, found: 340.1705.

IR (thin film) ν_{max}: 2952, 2875, 2078, 1701 (C=O), 1455, 1403, 1055, 1033, 1008 cm^{-1}.

1-(1-Azabicyclo[1.1.0]butan-3-yl)-2,2-dimethyl-3-((triethylsilyl)oxy)propan-1-one: 3f

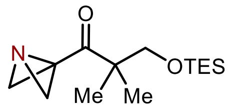

ABB-Li was synthesised according to General Procedure **F** followed by the addition of **2f** (331 mg, 1.20 equiv.) as a solution in anhydrous THF (1 mL). Purified by flash column chromatography (SiO_2; 12.3:1 hexane:EtOAc) to afford **3f** (80.7 mg, 0.299 mmol, 30%) as a colourless oil.

TLC: R_f = 0.28 (12.3:1 hexane:EtOAc).

^{1}H NMR (400 MHz, $CDCl_3$) δ 3.70 (s, 2H, C*H*$_2$OSi), 2.90 (dd, *J* = 1.8, 1.0 Hz, 2H, NC*H*$_2$), 1.45 (dd, *J* = 1.8, 1.0 Hz, 2H, NC*H*$_2$), 1.20 (s, 6H, C(C*H*$_3$)$_2$), 0.93 (t, *J* = 7.9 Hz, 9H, Si(CH$_2$C*H*$_3$)$_3$), 0.58 (q, *J* = 7.9 Hz, 6H, Si(C*H*$_2$CH$_3$)$_3$) ppm. **^{13}C NMR** (126 MHz, $CDCl_3$) δ 209.1 (*C*=O), 69.7 (*C*H$_2$OSi), 56.0 (N*C*H$_2$), 50.9 (*C*(CH$_3$)$_2$), 28.6 (*C*N(CH$_2$)$_2$), 21.6 (C(*C*H$_3$)$_2$), 6.9 (Si(CH$_2$*C*H$_3$)$_3$), 4.4 (Si(*C*H$_2$CH$_3$)$_3$) ppm. (*see spectra*)

HRMS (m/z): (ESI) calc'd for $C_{14}H_{27}NNaO_2Si$ $[M+Na]^+$: 292.1703, found: 292.1708.

IR (thin film) ν_{max}: 2954, 2876, 1692 (C=O), 1471, 1398, 1239, 1098, 1033, 1007 cm^{-1}.

1-(1-Azabicyclo[1.1.0]butan-3-yl)-3-((trimethylsilyl)oxy)butan-1-one: 3g

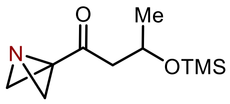

ABB-Li was synthesised according to General Procedure **F** followed by the addition of **2g** (263 mg, 1.20 equiv.) as a solution in anhydrous THF (1 mL). Purified by flash column chromatography (SiO_2; 5.7:1 hexane:EtOAc) to afford **3g** (154 mg, 0.722 mmol, 72%) as a colourless oil.

TLC: R_f = 0.24 (5.7:1 hexane:EtOAc).

^{1}H NMR (400 MHz, $CDCl_3$) δ 4.37 – 4.27 (m, 1H, C*H*CH$_3$), 2.96 (dd, *J* = 6.5, 2.1 Hz, 1H, NC*H*$_2$), 2.93 (dd, *J* = 6.5, 2.2 Hz, 1H, NC*H*$_2$), 2.77 (dd, *J* = 15.7, 6.9 Hz, 1H, (C=O)C*H*$_2$), 2.54 (dd, *J* = 15.7, 5.6 Hz, 1H, (C=O)C*H*$_2$), 1.56 (d, *J* = 2.1 Hz, 1H, NC*H*$_2$), 1.54 (d, *J* = 2.2 Hz, 1H, NC*H*$_2$), 1.20 (d, *J* = 6.1 Hz, 3H, CHC*H*$_3$), 0.10 (s, 9H, Si(C*H*$_3$)$_3$) ppm. **^{13}C NMR** (126 MHz, $CDCl_3$) δ 205.4 (*C*=O), 65.2 (*C*HCH$_3$), 55.8 (N*C*H$_2$), 55.6 (N*C*H$_2$), 48.9 ((C=O)*C*H$_2$), 30.7 (*C*N(CH$_2$)$_2$), 24.3 (CH*C*H$_3$), 0.3 (Si(*C*H$_3$)$_3$) ppm. (*see spectra*)

HRMS (m/z): (ESI) calc'd for $C_{10}H_{19}NNaO_2Si$ $[M+Na]^+$: 236.1077, found: 236.1083.

IR (thin film) ν_{max}: 2954, 2912, 2877, 1698 (C=O), 1458, 1408, 1238, 1131, 1002 cm^{-1}.

1-(1-Azabicyclo[1.1.0]butan-3-yl)-3-cyclopropyl-3-((trimethylsilyl)oxy)propan-1-one: 3h

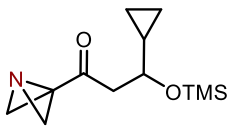

ABB-Li was synthesised according to General Procedure **F** followed by the addition of **2h** (294 mg, 1.20 equiv.) as a solution in anhydrous THF (1 mL). Purified by flash column chromatography (SiO_2; 5.7:1 hexane:EtOAc) to afford **3h** (156 mg, 0.652 mmol, 65%) as a colourless oil.

TLC: R_f = 0.26 (5.7:1 hexane:EtOAc).

^{1}H NMR (400 MHz, $CDCl_3$) δ 3.59 (app-td, *J* = 7.8, 4.4 Hz, 1H, C*H*OSi), 2.97 (dd, *J* = 6.5, 2.2 Hz, 1H, NC*H*$_2$), 2.93 (dd, *J* = 6.5, 2.3 Hz, 1H, NC*H*$_2$), 2.86 (dd, *J* = 15.2, 7.8 Hz, 1H, (C=O)C*H*$_2$), 2.69 (dd, *J* = 15.2, 4.4 Hz, 1H, (C=O)C*H*$_2$), 1.56 (d, *J* = 2.2 Hz, 1H, NC*H*$_2$), 1.54 (d, *J* = 2.3 Hz, 1H, NC*H*$_2$), 1.01 – 0.86 (m, 1H, cyclopropane C*H*), 0.55 – 0.42 (m, 2H, cyclopropane C*H*$_2$), 0.37 – 0.25 (m, 1H, cyclopropane C*H*$_2$), 0.23 – 0.16 (m, 1H, cyclopropane C*H*$_2$), 0.10 (s, 9H, Si(C*H*$_3$)$_3$) ppm. **^{13}C NMR** (126 MHz, $CDCl_3$) δ 205.4 (*C*=O), 73.1 (*C*HOSi), 55.8 (N*C*H$_2$), 55.6 (N*C*H$_2$), 47.8 ((C=O)*C*H$_2$), 30.8 (*C*N(CH$_2$)$_2$), 18.0 (cyclopropane *C*H), 4.1 (cyclopropane *C*H$_2$), 2.7 (cyclopropane *C*H$_2$), 0.5 (Si(*C*H$_3$)$_3$) ppm. (*see spectra*)

HRMS (m/z): (ESI) calc'd for $C_{12}H_{21}NNaO_2Si$ $[M+Na]^+$: 262.123376, found: 262.123555.

IR (thin film) ν_{max}: 3081, 2956, 2899, 1698 (C=O), 1404, 1250, 1064 cm^{-1}.

1-(1-Azabicyclo[1.1.0]butan-3-yl)-4-methyl-3-((trimethylsilyl)oxy)pentan-1-one: 3i

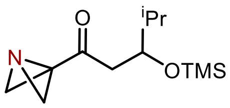

ABB-Li was synthesised according to General Procedure **F** followed by the addition of **2i** (297 mg, 1.20 equiv.) as a solution in anhydrous THF (1 mL). Purified by flash column chromatography (SiO_2; 9:1 hexane:EtOAc) to afford **3i** (161 mg, 0.667 mmol, 67%) as a colourless oil.

TLC: R_f = 0.28 (9:1 hexane:EtOAc).

^{1}H NMR (500 MHz, $CDCl_3$) δ 4.06 (ddd, *J* = 8.3, 4.7, 3.8 Hz, 1H, C*H*OSi), 2.97 (dd, *J* = 6.5, 2.2 Hz, 1H, NC*H*$_2$), 2.91 (dd, *J* = 6.5, 2.3 Hz, 1H, NC*H*$_2$), 2.69 (dd, *J* = 15.7, 8.3 Hz, 1H, (C=O)C*H*$_2$), 2.51 (dd, *J* = 15.7, 3.8 Hz, 1H, (C=O)C*H*$_2$), 1.69 (app-heptd, *J* = 6.8, 4.7 Hz, 1H, C*H*(CH$_3$)$_2$), 1.56 (d, *J* = 2.2 Hz, 1H, NC*H*$_2$), 1.53 (d, *J* = 2.3 Hz, 1H, NC*H*$_2$), 0.88 (d, *J* = 6.8 Hz, 3H, CH(C*H*$_3$)$_2$), 0.86 (d, *J* = 6.8 Hz, 3H, CH(C*H*$_3$)$_2$), 0.09 (s, 9H, Si(C*H*$_3$)$_3$) ppm.

^{13}C NMR (126 MHz, $CDCl_3$) δ 205.9 (*C*=O), 73.4 (*C*HOSi), 55.8 (N*C*H_2), 55.5 (N*C*H_2), 43.7 ((C=O)*C*H_2), 34.2 (*C*H(CH_3)$_2$), 30.8 (*C*N(CH_2)$_2$), 18.2 (CH(*C*H_3)$_2$), 17.8 (CH(*C*H_3)$_2$), 0.5 (Si(*C*H_3)$_3$) ppm. (*see spectra*)

HRMS (m/z): (ESI) calc'd for $C_{12}H_{23}NNaO_2Si$ $[M+Na]^+$: 264.1390, found: 264.1399.

IR (thin film) ν_{max}: 2958, 2898, 1699 (C=O), 1406, 1249, 1056 cm^{-1}.

1-(1-Azabicyclo[1.1.0]butan-3-yl)-3-phenyl-3-((trimethylsilyl)oxy)propan-1-one: 3j

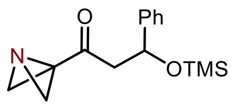

ABB-Li was synthesised according to General Procedure **F** followed by the addition of **2j** (338 mg, 1.20 equiv.) as a solution in anhydrous THF (1 mL). Purified by flash column chromatography (SiO_2; 9:1 hexane:EtOAc) to afford **3j** (111 mg, 0.404 mmol, 40%) as a colourless oil.

TLC: R_f = 0.23 (9:1 hexane:EtOAc).

^{1}H NMR (500 MHz, $CDCl_3$) δ 7.35 – 7.29 (m, 4H, ArC*H*), 7.27 – 7.23 (m, 1H, ArC*H*), 5.22 (dd, *J* = 8.7, 3.9 Hz, 1H, C*H*OSi), 3.04 (dd, *J* = 15.9, 8.7 Hz, 1H, (C=O)C*H*$_2$), 2.94 (dd, *J* = 6.5, 2.2 Hz, 1H, NC*H*$_2$), 2.91 (dd, *J* = 6.5, 2.3 Hz, 1H, NC*H*$_2$), 2.69 (dd, *J* = 15.9, 3.9 Hz, 1H, (C=O)C*H*$_2$), 1.54 (d, *J* = 2.2 Hz, 1H, NC*H*$_2$), 1.51 (d, *J* = 2.3 Hz, 1H, NC*H*$_2$), 0.02 (s, 9H, Si(C*H*$_3$)$_3$) ppm. **^{13}C NMR** (126 MHz, $CDCl_3$) δ 204.6 (*C*=O), 144.3 (Ar*C*), 128.5 (Ar*C*H), 127.6 (Ar*C*H), 125.8 (Ar*C*H), 71.1 (*C*HOSi), 55.8 (N*C*H_2), 55.5 (N*C*H_2), 50.2 ((C=O)*C*H_2), 30.7 (*C*N(CH_2)$_2$), 0.1 (Si(*C*H_3)$_3$) ppm. (*see spectra*)

HRMS (m/z): (ESI) calc'd for $C_{15}H_{21}NNaO_2Si$ $[M+Na]^+$: 298.1234, found: 298.1233.

IR (thin film) ν_{max}: 2956, 2895, 1702 (C=O), 1405, 1251, 1070 cm^{-1}.

1-(1-Azabicyclo[1.1.0]butan-3-yl)-3-(pyridin-3-yl)-3-((trimethylsilyl)oxy)propan-1-one: 3k

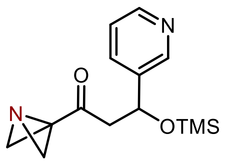

ABB-Li was synthesised according to General Procedure **F** followed by the addition of **2k** (339 mg, 1.20 equiv.) as a solution in anhydrous THF (1 mL). Purified by flash column chromatography (SiO_2; 0.7:1 hexane:EtOAc) to afford **3k** (69.5 mg, 0.251 mmol, 25%) as a yellow solid.

TLC: R_f = 0.25 (0.7:1 hexane:EtOAc).

^{1}**H NMR** (400 MHz, $CDCl_3$) δ 8.60 (d, *J* = 1.6 Hz, 1H, ArC*H*), 8.52 (dd, *J* = 4.8, 1.6 Hz, 1H, ArC*H*), 7.69 – 7.61 (m, 1H, ArC*H*), 7.31 – 7.21 (m, 1H, ArC*H*), 5.27 (dd, *J* = 8.3, 4.3 Hz, 1H, C*H*OSi), 3.07 (dd, *J* = 16.2, 8.3 Hz, 1H, (C=O)CH_2), 2.95 (dd, *J* = 6.5, 2.1 Hz, 1H, NCH_2), 2.91 (dd, *J* = 6.5, 2.1 Hz, 1H, NCH_2), 2.72 (dd, *J* = 16.2, 4.3 Hz, 1H, (C=O)CH_2), 1.57 (d, *J* = 2.1 Hz, 1H, NCH_2), 1.54 (d, *J* = 2.1 Hz, 1H, NCH_2), 0.04 (s, 9H, Si(CH_3)$_3$) ppm. ^{13}**C NMR** (126 MHz, $CDCl_3$) δ 204.0 (*C*=O), 149.2 (Ar*C*H), 147.9 (Ar*C*H), 139.6 (Ar*C*), 133.6 (Ar*C*H), 123.5 (Ar*C*H), 68.8 (*C*HOSi), 55.8 (N*C*H_2), 55.6 (N*C*H_2), 49.8 ((C=O)*C*H_2), 30.6 (*C*N(CH_2)$_2$), 0.1 (Si(*C*H_3)$_3$) ppm. (*see spectra*)

HRMS (m/z): (ESI) calc'd for $C_{14}H_{20}N_2NaO_2Si$ $[M+Na]^+$: 299.118625, found: 299.118227.

IR (thin film) ν_{max}: 2954, 1701 (C=O), 1407, 1252, 1092 cm^{-1}.

1-(1-Azabicyclo[1.1.0]butan-3-yl)-3-((trimethylsilyl)oxy)pent-4-en-1-one: 3l

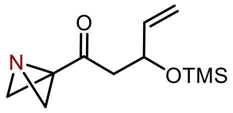

ABB-Li was synthesised according to General Procedure **F** followed by the addition of **2l** (278 mg, 1.20 equiv.) as a solution in anhydrous THF (1 mL). Purified by flash column chromatography (SiO_2; 5.7:1 hexane:EtOAc) to afford **3l** (131 mg, 0.581 mmol, 58%) as a colourless oil.

TLC: R_f = 0.26 (5.7:1 hexane:EtOAc).

^{1}**H NMR** (400 MHz, $CDCl_3$) δ 5.84 (ddd, *J* = 17.1, 10.4, 5.6 Hz, 1H, *H*C=CH_2), 5.24 (app-dt, *J* = 17.1, 1.5 Hz, 1H, HC=CH_2), 5.08 (app-dt, *J* = 10.4, 1.5 Hz, 1H, HC=CH_2), 4.73 – 4.61 (m, 1H, C*H*OSi), 2.96 (dd, *J* = 6.5, 2.1 Hz, 1H, NCH_2), 2.93 (dd, *J* = 6.5, 2.2 Hz, 1H, NCH_2), 2.81 (dd, *J* = 15.7, 7.8 Hz, 1H, (C=O)CH_2), 2.59 (dd, *J* = 15.7, 4.8 Hz, 1H, (C=O)CH_2), 1.56 (d, *J* = 2.1 Hz, 1H, NCH_2), 1.54 (d, *J* = 2.2 Hz, 1H, NCH_2), 0.11 (s, 9H, Si(CH_3)$_3$) ppm. ^{13}**C NMR** (126 MHz, $CDCl_3$) δ 204.7 (*C*=O), 140.1 (H*C*=CH_2), 114.8 (HC=*C*H_2), 69.9 (*C*HOSi), 55.8 (N*C*H_2), 55.6 (N*C*H_2), 47.4 ((C=O)*C*H_2), 30.7 (*C*N(CH_2)$_2$), 0.3 (Si(*C*H_3)$_3$) ppm. (*see spectra*)

HRMS (m/z): (ESI) calc'd for $C_{11}H_{19}NNaO_2Si$ $[M+Na]^+$: 248.107726, found: 248.108947.

IR (thin film) ν_{max}: 2956, 1699 (C=O), 1404, 1251, 1132, 1071, 1024 cm^{-1}.

1-(1-Azabicyclo[1.1.0]butan-3-yl)-4-chloro-3-((trimethylsilyl)oxy)butan-1-one: 3m

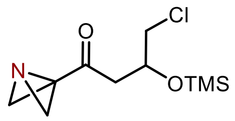

ABB-Li was synthesised according to General Procedure **F** followed by the addition of **2m** (305 mg, 1.20 equiv.) as a solution in anhydrous THF (1 mL). Purified by flash column chromatography (SiO_2; 5.7:1 hexane:EtOAc) to afford **3m** (86.8 mg, 0.350 mmol, 35%) as a colourless oil.

TLC: R_f = 0.23 (5.7:1 hexane:EtOAc).

^{1}H NMR (400 MHz, $CDCl_3$) δ 4.40 (app-dq, *J* = 7.1, 5.0 Hz, 1H, C*H*OSi), 3.53 – 3.43 (m, 2H, C*H*$_2$Cl), 2.98 (dd, *J* = 6.5, 2.0 Hz, 1H, NC*H*$_2$), 2.95 (dd, *J* = 6.5, 2.0 Hz, 1H, NC*H*$_2$), 2.86 (dd, *J* = 16.6, 5.0 Hz, 1H, (C=O)C*H*$_2$), 2.77 (dd, *J* = 16.6, 7.1 Hz, 1H, (C=O)C*H*$_2$), 1.58 (d, *J* = 2.0 Hz, 1H, NC*H*$_2$), 1.57 (d, *J* = 2.0 Hz, 1H, NC*H*$_2$), 0.14 (s, 9H, Si(C*H*$_3$)$_3$) ppm. **^{13}C NMR** (126 MHz, $CDCl_3$) δ 204.3 (*C*=O), 68.5 (*C*HOSi), 55.8 (N*C*H$_2$), 55.7 (N*C*H$_2$), 48.6 (*C*H$_2$Cl), 44.4 ((C=O)*C*H$_2$), 30.6 (*C*N(CH$_2$)$_2$), 0.3 (Si(*C*H$_3$)$_3$) ppm. (*see spectra*)

HRMS (m/z): (ESI) calc'd for $C_{10}H_{18}ClNNaO_2Si$ $[M+Na]^+$: 270.0688, found: 270.0683.

IR (thin film) ν_{max}: 2955, 1701 (C=O), 1409, 1260, 1075 cm^{-1}.

4-(Benzyloxy)-1-(1-azabicyclo[1.1.0]butan-3-yl)-3-((trimethylsilyl)oxy)butan-1-one: 3n

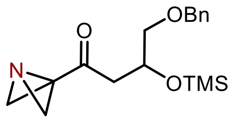

ABB-Li was synthesised according to General Procedure **F** followed by the addition of **2n** (391 mg, 1.20 equiv.) as a solution in anhydrous THF (1 mL). Purified by flash column chromatography (SiO_2; 5.7:1 hexane:EtOAc) to afford **3n** (147 mg, 0.460 mmol, 46%) as a colourless oil.

TLC: R_f = 0.28 (5.7:1 hexane:EtOAc).

^{1}H NMR (400 MHz, $CDCl_3$) δ 7.39 – 7.27 (m, 5H, ArC*H*), 4.53 (s, 2H, OC*H*$_2$Ph), 4.42 – 4.32 (m, 1H, C*H*OSi), 3.44 (dd, *J* = 9.7, 5.1 Hz, 1H, CHC*H*$_2$O), 3.37 (dd, *J* = 9.7, 5.5 Hz, 1H, CHC*H*$_2$O), 2.97 – 2.89 (m, 2H, NC*H*$_2$), 2.78 (dd, *J* = 16.0, 4.9 Hz, 1H, (C=O)C*H*$_2$), 2.71 (dd, *J* = 16.0, 7.3 Hz, 1H, (C=O)C*H*$_2$), 1.54 (d, *J* = 2.0 Hz, 1H, NC*H*$_2$), 1.52 (d, *J* = 2.1 Hz, 1H, NC*H*$_2$), 0.10 (s, 9H, Si(C*H*$_3$)$_3$) ppm. **^{13}C NMR** (126 MHz, $CDCl_3$) δ 205.0 (*C*=O), 138.2 (Ar*C*), 128.5 (Ar*C*H), 127.8 (Ar*C*H), 127.8 (Ar*C*H), 74.0 (CH*C*H$_2$O), 73.5 (O*C*H$_2$Ph), 67.9 (*C*HOSi), 55.8 (N*C*H$_2$), 55.6 (N*C*H$_2$), 44.3 ((C=O)*C*H$_2$), 30.7 (*C*N(CH$_2$)$_2$), 0.3 (Si(*C*H$_3$)$_3$) ppm. (*see spectra*)

HRMS (m/z): (ESI) calc'd for $C_{17}H_{25}NNaO_3Si$ $[M+Na]^+$: 342.149591, found: 342.150364.

IR (thin film) ν_{max}: 2954, 2902, 1700 (C=O), 1408, 1249, 1092 cm^{-1}.

1-(1-Azabicyclo[1.1.0]butan-3-yl)-3-methyl-3-((trimethylsilyl)oxy)butan-1-one: 3o

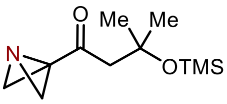

ABB-Li was synthesised according to General Procedure **F** followed by the addition of **2o** (280 mg, 1.20 equiv.) as a solution in anhydrous THF (1 mL). Purified by flash column chromatography (SiO_2; 15.7:1 hexane:EtOAc) to afford **3o** (136 mg, 0.598 mmol, 60%) as a colourless oil.

TLC: R_f = 0.29 (15.7:1 hexane:EtOAc).

^{1}H NMR (400 MHz, $CDCl_3$) δ 2.96 (s, 2H, NCH_2), 2.63 (s, 2H, (C=O)CH_2), 1.53 (s, 2H, NCH_2), 1.36 (s, 6H, C(CH_3)$_2$), 0.11 (s, 9H, Si(CH_3)$_3$) ppm. **^{13}C NMR** (126 MHz, $CDCl_3$) δ 205.9 (*C*=O), 73.9 (*C*(CH$_3$)$_2$), 56.0 (N*C*H$_2$), 53.2 ((C=O)*C*H$_2$), 31.4 (*C*N(CH$_2$)$_2$), 30.3 (C(*C*H$_3$)$_2$), 2.6 (Si(*C*H$_3$)$_3$) ppm. (see spectra)

HRMS (m/z): (ESI) calc'd for $C_{11}H_{21}NNaO_2Si$ [M+Na]$^+$: 250.1234, found: 250.1239.

IR (thin film) ν_{max}: 2956, 1693 (C=O), 1404, 1250, 1035, 838 cm^{-1}.

1-(1-Azabicyclo[1.1.0]butan-3-yl)-2-(1-((trimethylsilyl)oxy)cyclohexyl)ethan-1-one: 3p

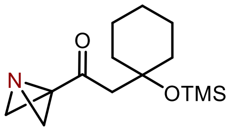

ABB-Li was synthesised according to General Procedure **F** followed by the addition of **2p** (328 mg, 1.20 equiv.) as a solution in anhydrous THF (1 mL). Purified by flash column chromatography (SiO_2; 9:1 hexane:EtOAc) to afford **3p** (188 mg, 0.703 mmol, 70%) as a colourless oil.

TLC: R_f = 0.20 (9:1 hexane:EtOAc).

^{1}H NMR (400 MHz, $CDCl_3$) δ 2.97 – 2.93 (s, 2H, NCH_2), 2.69 (s, 2H, (C=O)*C*H$_2$), 1.84 – 1.74 (m, 2H cy-CH_2), 1.73 – 1.62 (m, 2H, cy-CH_2), 1.60 – 1.54 (m, 2H, cy-CH_2), 1.53 (s, 2H, NCH_2), 1.49 – 1.22 (m, 4H, cy-CH_2), 0.12 (s, 9H, Si(CH_3)$_3$) ppm. **^{13}C NMR** (126 MHz, $CDCl_3$) δ 206.1 (*C*=O), 76.3 (cy-*C*OSi), 56.0 (N*C*H$_2$), 48.7 ((C=O)*C*H$_2$), 39.0 (cy-*C*H$_2$), 31.7 (*C*N(CH$_2$)$_2$), 25.6 (cy-*C*H$_2$), 23.3 (cy-*C*H$_2$), 2.9 (Si(*C*H$_3$)$_3$) ppm. (see spectra)

HRMS (m/z): (ESI) calc'd for $C_{14}H_{25}NNaO_2Si$ [M+Na]$^+$: 290.1547, found: 290.1551.

IR (thin film) ν_{max}: 2936, 2863, 1691 (C=O), 1402, 1250, 1057, 1033 cm^{-1}.

***syn*-1-(1-Azabicyclo[1.1.0]butan-3-yl)-2-methyl-3-((trimethylsilyl)oxy)pent-4-en-1-one: *syn*-3q**

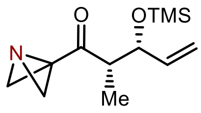

ABB-Li was synthesised according to General Procedure **F** followed by the addition of *syn*-**2q** (97:3 dr, 294 mg, 1.20 equiv.) as a solution in anhydrous THF (1 mL). Purified by flash column chromatography (SiO_2; 5.7:1 hexane:EtOAc) to afford **3q** as the *syn*-diastereomer 97:3 dr (75.1 mg, 0.314 mmol, 31%) as a colourless oil.

TLC: R_f = 0.24 (7:1 hexane:EtOAc).

^{1}H NMR (400 MHz, $CDCl_3$) *syn*-diastereomer δ 5.78 (ddd, *J* = 17.1, 10.4, 6.1 Hz, 1H, *H*C=CH_2), 5.19 (app-dt, *J* = 17.1, 1.5 Hz, 1H, HC=CH_2), 5.12 (app-dt, *J* = 10.4, 1.5 Hz, 1H, HC=CH_2), 4.35 – 4.29 (m, 1H, C*H*OSi), 2.97 – 2.84 (m, 3H, C*H*CH_3, NCH_2), 1.57 (d, *J* = 2.2 Hz, 1H, NCH_2), 1.51 (d, *J* = 2.3 Hz, 1H, NCH_2), 1.13 (d, *J* = 6.9 Hz, 3H, CHCH_3), 0.11 (s, 9H, Si(CH_3)$_3$) ppm. **^{13}C NMR** (126 MHz, $CDCl_3$) *syn*-diastereomer δ 208.7 (*C*=O), 138.8 (H*C*=CH_2), 116.1 (HC=*C*H_2), 75.1 (*C*HOSi), 55.8 (N*C*H_2), 55.4 (N*C*H_2), 49.3 (*C*HCH_3), 30.4 (*C*N(CH_2)$_2$), 13.2 (CH*C*H_3), 0.3 (Si(*C*H_3)$_3$) ppm. (*see spectra*)

HRMS (m/z): (ESI) calc'd for $C_{12}H_{21}NNaO_2Si$ $[M+Na]^+$: 262.1234, found: 262.1239.

IR (thin film) ν_{max}: 2958, 1695 (C=O), 1405, 1253, 1137, 1012 cm^{-1}.

***anti*-1-(1-Azabicyclo[1.1.0]butan-3-yl)-2-methyl-3-((trimethylsilyl)oxy)pent-4-en-1-one: *anti*-3q**

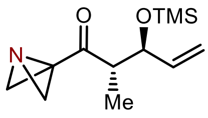

ABB-Li was synthesised according to General Procedure **F** followed by the addition of *anti*-**2q** (>98:2 dr, 294 mg, 1.20 equiv.) as a solution in anhydrous THF (1 mL). Purified by flash column chromatography (SiO_2; 7:1 hexane:EtOAc) to afford **3q** as the *anti*-diastereomer >98:2 dr (52.4 mg, 0.219 mmol, 22%) as a colourless oil.

TLC: R_f = 0.32 (5.7:1 hexane:EtOAc).

^{1}H NMR (400 MHz, $CDCl_3$) *anti*-diastereomer δ 5.74 (ddd, *J* = 17.2, 10.2, 7.3 Hz, 1H, *H*C=CH_2), 5.25 – 5.13 (m, 2H, HC=CH_2), 4.21 (app-ddt, *J* = 8.6, 7.3, 1.0 Hz, 1H, C*H*OSi), 3.00 (dd, *J* = 6.5, 2.2 Hz, 1H, NCH_2), 2.92 – 2.82 (m, 2H, C*H*CH_3, NCH_2), 1.57 (dd, *J* = 2.2, 0.5 Hz, 1H, NCH_2), 1.50 (dd, *J* = 2.2, 0.5 Hz, 1H, NCH_2), 0.97 (d, *J* = 6.9 Hz, 3H, CHCH_3), 0.06 (s, 9H, Si(CH_3)$_3$) ppm. **^{13}C NMR** (126 MHz, $CDCl_3$) *anti*-diastereomer δ 210.1 (*C*=O),

138.9 (H*C*=CH$_2$), 117.0 (HC=*C*H$_2$), 77.4 (*C*HOSi), 55.9 (N*C*H$_2$), 55.2 (N*C*H$_2$), 48.6 (*C*HCH$_3$), 31.1 (*C*N(CH$_2$)$_2$), 13.5 (CHC*H*$_3$), 0.3 (Si(*C*H$_3$)$_3$) ppm. (*see spectra*)

1-(1-Azabicyclo[1.1.0]butan-3-yl)-4-((trimethylsilyl)oxy)pentan-1-one: 3r

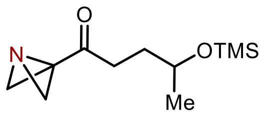

ABB-Li was synthesised according to General Procedure **F** followed by the addition of **2r** (280 mg, 1.20 equiv.) as a solution in anhydrous THF (1 mL). Purified by flash column chromatography (SiO$_2$; 7:1 hexane:EtOAc) to afford **3r** (163 mg, 0.717 mmol, 72%) as a colourless oil.

TLC: R_f = 0.18 (7:1 hexane:EtOAc).

^{1}H NMR (400 MHz, CDCl$_3$) δ 3.83 (dqd, *J* = 7.7, 6.1, 4.1 Hz, 1H, C*H*CH$_3$), 2.94 (s, 2H, NC*H*$_2$), 2.70 – 2.49 (m, 2H, (C=O)C*H*$_2$), 1.84 – 1.74 (m, 1H, C*H*$_2$CH), 1.73 – 1.61 (m, 1H, C*H*$_2$CH), 1.54 (s, 2H, NC*H*$_2$), 1.14 (d, *J* = 6.1 Hz, 3H, CHC*H*$_3$), 0.10 (s, 9H, Si(C*H*$_3$)$_3$) ppm. **^{13}C NMR** (126 MHz, CDCl$_3$) δ 206.5 (*C*=O), 67.4 (*C*HCH$_3$), 55.5 (N*C*H$_2$), 55.5 (N*C*H$_2$), 35.4 ((C=O)*C*H$_2$), 32.8 (*C*H$_2$CH), 30.0 (*C*N(CH$_2$)$_2$), 23.9 (CH*C*H$_3$), 0.4 (Si(*C*H$_3$)$_3$) ppm. (*see spectra*)

HRMS (m/z): (ESI) calc'd for $C_{11}H_{21}NNaO_2Si$ $[M+Na]^+$: 250.1234, found: 250.1249.

IR (thin film) ν_{max}: 2954, 1700 (C=O), 1407, 1251, 1137 cm^{-1}.

1-(1-Azabicyclo[1.1.0]butan-3-yl)-5-((trimethylsilyl)oxy)pentan-1-one: 3s

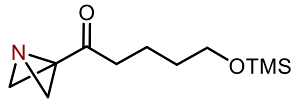

ABB-Li was synthesised according to General Procedure **F** followed by the addition of **2s** (280 mg, 1.20 equiv.) as a solution in anhydrous THF (1 mL). Purified by flash column chromatography (SiO$_2$; 5.7:1 hexane:EtOAc) to afford **3s** (74.9 mg, 0.329 mmol, 33%) as a colourless oil.

TLC: R_f = 0.19 (5.7:1 hexane:EtOAc).

^{1}H NMR (400 MHz, CDCl$_3$) δ 3.58 (t, *J* = 6.3 Hz, 2H, C*H*$_2$OSi), 2.93 (s, 2H, NC*H*$_2$), 2.56 (t, *J* = 7.3 Hz, 2H, (C=O)C*H*$_2$), 1.74 – 1.62 (m, 2H, C*H*$_2$CH$_2$CH$_2$OSi), 1.61 – 1.49 (m, 4H, CH$_2$C*H*$_2$CH$_2$OSi, NC*H*$_2$), 0.10 (s, 9H, Si(C*H*$_3$)$_3$) ppm. **^{13}C NMR** (126 MHz, CDCl$_3$) δ 206.4 (*C*=O), 62.3 (*C*H$_2$OSi), 55.5 (N*C*H$_2$), 39.0 ((C=O)*C*H$_2$), 32.0 (CH$_2$*C*H$_2$CH$_2$OSi), 30.0 (*C*N(CH$_2$)$_2$), 20.0 (*C*H$_2$CH$_2$CH$_2$OSi), −0.4 (Si(*C*H$_3$)$_3$) ppm. (*see spectra*)

HRMS (m/z): (ESI) calc'd for $C_{11}H_{21}NNaO_2Si$ $[M+Na]^+$: 250.1234, found: 250.1242.

IR (thin film) ν_{max}: 2949, 1701 (C=O), 1407, 1275, 1260, 1097 cm^{-1}.

(1-Azabicyclo[1.1.0]butan-3-yl)(2-((*tert*-butyldimethylsilyl)oxy)phenyl)methanone: 3t

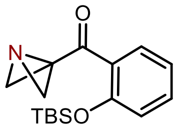

ABB-Li was synthesised according to General Procedure **F** followed by the addition of **2t** (337 mg, 1.20 equiv.) as a solution in anhydrous THF (1 mL). Purified by flash column chromatography (SiO_2; 9:1 hexane:EtOAc) to afford **3t** (68.1 mg, 0.235 mmol, 24%) as a colourless oil.

TLC: R_f = 0.22 (9:1 hexane:EtOAc).

^{1}H NMR (400 MHz, $CDCl_3$) δ 7.41 (dd, *J* = 7.6, 1.9 Hz, 1H, ArC*H*), 7.36 (ddd, *J* = 8.2, 7.6, 1.9 Hz, 1H, ArC*H*), 7.00 (app-td, *J* = 7.6, 1.0 Hz, 1H, ArC*H*), 6.85 (dd, *J* = 8.2, 1.0 Hz, 1H, ArC*H*), 2.97 (dd, *J* = 1.4, 0.9 Hz, 2H, NC*H*$_2$), 1.69 (dd, *J* = 1.4, 0.9 Hz, 1H, NC*H*$_2$), 0.98 (s, 9H, SiC(C*H*$_3$)$_3$), 0.20 (s, 6H, Si(C*H*$_3$)) ppm. **^{13}C NMR** (126 MHz, $CDCl_3$) δ 201.6 (*C*=O), 154.4 (Ar*C*), 133.1 (Ar*C*H), 130.2 (Ar*C*), 129.7 (Ar*C*H), 121.6 (Ar*C*H), 120.4 (Ar*C*H), 57.1 (N*C*H$_2$), 30.8 (*C*N(CH$_2$)$_2$), 25.8 (SiC(*C*H$_3$)$_3$), 18.3 (Si*C*(CH$_3$)$_3$), −4.1 (Si(*C*H$_3$)$_2$) ppm. (*see spectra*)

HRMS (m/z): (ESI) calc'd for $C_{16}H_{23}NNaO_2Si$ $[M+Na]^+$: 312.1390, found: 312.1394.

IR (thin film) ν_{max}: 2932, 2860, 1679 (C=O), 1598, 1481, 1453, 1392, 1279, 1254 cm^{-1}.

(1-Azabicyclo[1.1.0]butan-3-yl)(3-((*tert*-butyldimethylsilyl)oxy)pyridin-2-yl)methanone: 3u

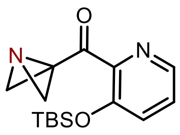

ABB-Li was synthesised according to General Procedure **F** followed by the addition of **2u** (321 mg, 1.20 equiv.) as a solution in anhydrous THF (1 mL). Purified by flash column chromatography (SiO_2; 2.3:1 hexane:EtOAc) to afford **3u** (73.0 mg, 0.251 mmol, 25%) as a yellow oil.

TLC: R_f = 0.28 (2.33:1 hexane:EtOAc).

^{1}H NMR (400 MHz, $CDCl_3$) δ 8.25 (dd, *J* = 4.5, 1.3 Hz, 1H, ArC*H*), 7.31 (dd, *J* = 8.4, 4.5 Hz, 1H, ArC*H*), 7.24 (dd, *J* = 8.4, 1.3 Hz, 1H, ArC*H*), 3.09 (s, 2H, NC*H*$_2$), 1.70 (s, 2H, NC*H*$_2$), 0.99 (s, 9H, SiC(C*H*$_3$)$_3$), 0.22 (s, 6H, Si(C*H*$_3$)) ppm. **^{13}C NMR** (126 MHz, $CDCl_3$) δ 197.3

(*C*=O), 151.4 (Ar*C*), 146.0 (Ar*C*), 141.6 (Ar*C*H), 128.9 (Ar*C*H), 127.0 (Ar*C*H), 57.1 (N*C*H_2), 30.4 (*C*N(CH_2)$_2$), 25.7 (SiC(*C*H_3)$_3$), 18.4 (Si*C*(CH_3)$_3$), −4.2 (Si(*C*H_3)$_2$) ppm. (*see spectra*)

HRMS (m/z): (ESI) calc'd for $C_{15}H_{22}N_2NaO_2Si$ $[M+Na]^+$: 313.1343, found: 313.1353.

IR (thin film) ν_{max}: 3064, 2931, 2858, 1731 (C=O), 1585, 1452, 1292 cm^{-1}.

3-((3-((*tert*-Butyldimethylsilyl)oxy)pyridin-2-yl)((trimethylsilyl)oxy)methyl)-1-azabicyclo[1.1.0]butane: 3u′

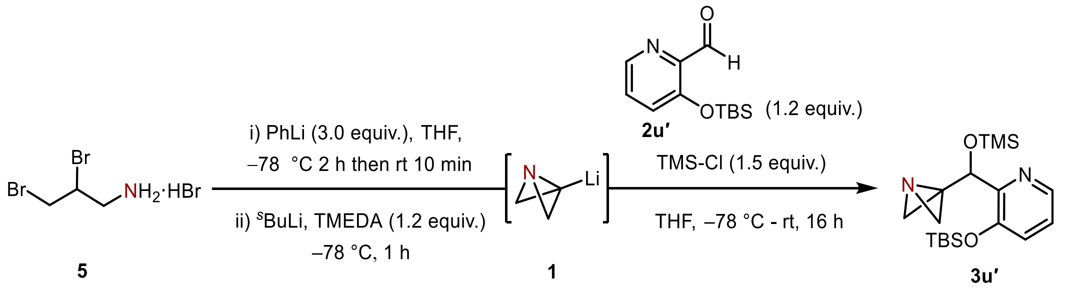

ABB-Li was synthesised according to General Procedure **F** followed by the addition of **2u′** (285 mg, 1.20 equiv.) as a solution in anhydrous THF (1 mL). The reaction was stirred for 1 h before trimethylsilyl chloride (0.190 mL, 1.50 equiv.) was added at a rate of 0.025 mL/min *via* syringe pump and the reaction was left to warm to rt slowly over 16 h. H_2O was then added to quench the reaction, and the mixture was extracted with EtOAc (3×). The combined organic phases were dried ($MgSO_4$), filtered, and concentrated under reduced pressure. The crude residue was purified by flash column chromatography[**A**] (SiO_2; 1.5:1 hexane:EtOAc) to afford **3u′** (98.7 mg, 0.271 mmol, 27%) as a yellow oil.

Notes: (**A**) Product showed signs of silica instability so column chromatography was performed rapidly.

TLC: R_f = 0.24 (SiO_2; 1.5:1 hexane:EtOAc).

^{1}H NMR (400 MHz, $CDCl_3$) δ 8.29 (dd, *J* = 3.4, 2.6 Hz, 1H, ArC*H*), 7.13 – 7.03 (m, 2H, ArC*H*), 5.59 (s, 1H, C*H*OSi), 2.51 (dd, *J* = 6.4, 3.0 Hz, 1H, NC*H*$_2$), 2.29 (dd, *J* = 6.4, 2.8 Hz, 1H, NC*H*$_2$), 1.24 (d, *J* = 3.0 Hz, 1H, NC*H*$_2$), 1.19 (d, *J* = 2.8 Hz, 1H, NC*H*$_2$), 1.02 (s, 9H, SiC(C*H*$_3$)$_3$), 0.28 (s, 3H, Si(C*H*$_3$)$_2$), 0.27 (s, 3H, Si(C*H*$_3$)$_2$), 0.07 (s, 9H, Si(C*H*$_3$)$_3$) ppm. **^{13}C NMR** (126 MHz, $CDCl_3$) δ 151.0 (Ar*C*), 149.1 (Ar*C*), 142.4 (Ar*C*H), 125.3 (Ar*C*H), 123.4 (Ar*C*H), 66.9 (*C*HOSi), 53.4 (N*C*H_2), 53.2 (N*C*H_2), 33.3 (*C*N(CH_2)$_2$), 25.9 (SiC(*C*H_3)$_3$), 18.4 (Si*C*(CH_3)$_3$), 0.3 (Si(*C*H_3)$_3$), −3.9 (Si(*C*H_3)$_2$) ppm. (*see spectra*)

HRMS (m/z): (ESI) calc'd for $C_{18}H_{22}N_2NaO_2Si_2$ $[M+Na]^+$: 387.1895, found: 387.1906.

IR (thin film) ν_{max}: 3052, 2955, 2959, 1695, 1441, 1252, 1087 cm^{-1}.

(1-Azabicyclo[1.1.0]butan-3-yl)(3-((*tert*-butyldimethylsilyl)oxy)naphthalen-2-yl)methanone: 3v

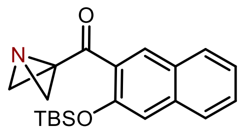

ABB-Li was synthesised according to General Procedure **F** followed by the addition of **2v** (380 mg, 1.20 equiv.) as a solution in anhydrous THF (1 mL). Purified by flash column chromatography (SiO_2; 9:1 hexane:EtOAc) to afford **3v** (174 mg, 0.513 mmol, 51%) as a bright yellow solid.

TLC: R_f = 0.21 (9:1 hexane:EtOAc).

^{1}H NMR (500 MHz, $CDCl_3$) δ 7.91 (s, 1H, ArC*H*), 7.80 (d, *J* = 8.2 Hz, 1H, ArC*H*), 7.69 (d, *J* = 8.2 Hz, 1H, ArC*H*), 7.50 (ddd, *J* = 8.2, 6.9, 1.1 Hz, 1H, ArC*H*), 7.38 (ddd, *J* = 8.2, 6.9, 1.1 Hz, 1H, ArC*H*), 7.18 (s, 1H, ArC*H*), 2.98 (s, 2H, NCH_2), 1.73 (s, 2H, NCH_2), 1.02 (s, 9H, SiC(CH_3)$_3$), 0.27 (s, 6H, Si(CH_3)$_2$) ppm. **^{13}C NMR** (126 MHz, $CDCl_3$) δ 201.5 (*C*=O), 151.0 (Ar*C*), 136.0 (Ar*C*), 131.5 (Ar*C*), 130.1 (Ar*C*H), 129.0 (Ar*C*H), 128.5 (Ar*C*), 128.2 (Ar*C*H), 126.6 (Ar*C*H), 124.9 (Ar*C*H), 115.2 (Ar*C*H), 57.2 (N*C*H_2), 31.0 (*C*N(CH_2)$_2$), 25.9 (SiC(*C*H_3)$_3$), 18.4 (Si*C*(CH_3)$_3$), −4.1 (Si(*C*H_3)$_2$) ppm. (see spectra)

HRMS (m/z): (ESI) calc'd for $C_{20}H_{25}NNaO_2Si$ $[M+Na]^+$: 362.154676, found: 362.156350.

IR (thin film) ν_{max}: 3059, 2931, 2858, 1680 (C=O), 1596, 1461, 1332, 1257, 1175 cm^{-1}.

3.6 Synthesis of oxa-azaspirocycles: 4a-4v

2-Methyl-6-(2,2,2-trifluoroacetyl)-1-oxa-6-azaspiro[3.3]heptan-3-one: 4a

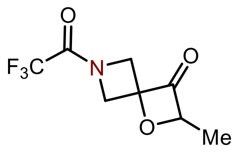

Synthesised according to General Procedure **G** from: **3a** (24.1 mg, 0.100 mmol, 1.00 equiv.), and TFAA (27.8 µL, 2.00 equiv.). Purified by flash column chromatography (Florisil®; 99:1 CH_2Cl_2:Et_2O) to afford **4a** (13.6 mg, 0.061 mmol, 61%) as a white solid.

^{1}H NMR (400 MHz, $CDCl_3$) δ 5.54 (2×q, *J* = 7.0 Hz, 1H, (C=O)C*H*), 4.78 (2×d, *J* = 11.9 Hz, 1H, NCH_2), 4.67 (d, *J* = 11.3 Hz, 1H, NCH_2), 4.57 (2×d, *J* = 11.3 Hz, 1H, NCH_2), 4.43 (2×d,

J = 11.9 Hz, 1H, NC*H*$_2$), 1.51 (2×d, *J* = 7.0 Hz, 3H, CHC*H*$_3$) ppm. **^{13}C NMR** (126 MHz, $CDCl_3$) δ 200.3 (*C*=O), 156.2 (q, *J* = 38.5 Hz, F_3C(*C*=O)), 115.9 (q, *J* = 288 Hz, F_3*C*(C=O)), 98.3 and 98.3 ((C=O)C*H*), 95.5 (*spiro-C*), 61.4 and 61.3 (N*C*H$_2$), 59.0 and 58.9 (N*C*H$_2$), 17.0 and 16.9 (CH*C*H$_3$) ppm. **^{19}F NMR** (377 MHz, $CDCl_3$) δ –72.7, –72.7 ppm. *Doubling of signals due to presence of rotamers*. (*see spectra*)

HRMS (m/z): (APCI) calc'd for $C_8H_9NO_3F_3$ $[M+H]^+$: 224.0529, found: 224.0522.

IR (thin film) ν_{max}: 2931, 2859, 1669 (C=O), 1386, 1090, 730 cm^{-1}.

Scale up reaction of 3a

Scale up reaction was performed according to a modified General Procedure **G** from: **3a** (966 mg, 4.00 mmol, 1.00 equiv.), CH_2Cl_2 (20 mL) and TFAA (1.11 mL, 2.00 equiv.). Purified by flash column chromatography (Florisil®; 99:1 to 49:1 CH_2Cl_2:Et_2O) to afford **4a** (544 mg, 2.44 mmol, 61%) as a white solid.

2-Benzyl-6-(2,2,2-trifluoroacetyl)-1-oxa-6-azaspiro[3.3]heptan-3-one: 4b

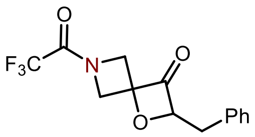

Synthesised according to General Procedure **G** from: **3b** (31.8 mg, 0.100 mmol, 1.00 equiv.), and TFAA (27.8 µL, 2.00 equiv.). Purified by flash column chromatography (Florisil®; 2.3:1 pentane:Et_2O) to afford **4b** (12.0 mg, 0.040 mmol, 40%) as a white solid.

^{1}H NMR (500 MHz, $CDCl_3$) δ 7.41 – 7.30 (m, 3H, ArC*H*), 7.23 – 7.17 (m, 2H, ArC*H*), 5.72 (2×dd, *J* = 8.7, 4.0 Hz, 1H, (C=O)C*H*), 4.64 (d, *J* = 11.5 Hz, 0.5H, NC*H*$_2$), 4.53 (d, *J* = 11.5 Hz, 0.5H, NC*H*$_2$), 4.43 (d, *J* = 12.3 Hz, 0.5H, NC*H*$_2$), 4.30 (d, *J* = 12.3 Hz, 0.5H, NC*H*$_2$), 3.95 (d, *J* = 11.5 Hz, 0.5H, NC*H*$_2$), 3.75 (2×d, *J* = 12.6 Hz, 1H, NC*H*$_2$), 3.57 (d, *J* = 12.6 Hz, 0.5H, NC*H*$_2$), 3.19 – 3.06 (m, 2H, PhC*H*$_2$) ppm. **^{13}C NMR** (126 MHz, $CDCl_3$) δ 200.1 and 200.1 (*C*=O), 155.8 (2×q, *J* = 38.4 Hz, F_3C(*C*=O)), 134.3 and 134.2 (Ar*C*), 130.3 and 130.2 (Ar*C*H), 128.9 and 128.9 (Ar*C*H), 128.0 and 127.8 (Ar*C*H), 115.8 (2×q, *J* = 288 Hz, F_3*C*(C=O)), 102.6 and 102.5 ((C=O)C*H*), 96.2 (*spiro-C*), 60.58 (q, *J* = 2.3 Hz, N*C*H$_2$) and 60.19 (q, *J* = 2.3 Hz, N*C*H$_2$), 58.2 and 57.9 (N*C*H$_2$), 37.4 (Ph*C*H$_2$) ppm. **^{19}F NMR** (377 MHz, $CDCl_3$) δ –72.8, –72.8 ppm. *Doubling of signals due to presence of rotamers*. (*see spectra*)

HRMS (m/z): (APCI) calc'd for $C_{14}H_{13}NO_3F_3$ $[M+H]^+$: 300.0842, found: 300.0840.

IR (thin film) ν_{max}: 2940, 1824 (C=O), 1669 (C=O), 1455, 1143, 960 cm^{-1}.

2,2-Dimethyl-6-(2,2,2-trifluoroacetyl)-1-oxa-6-azaspiro[3.3]heptan-3-one: 4c

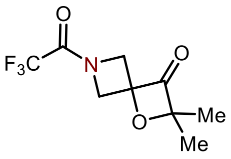

Synthesised according to General Procedure **G** from: **3c** (25.5 mg, 0.100 mmol, 1.00 equiv.), and TFAA (27.8 µL, 2.00 equiv.). Purified by flash column chromatography (Florisil®; 2.3:1 pentane:Et_2O) to afford **4c** (17.7 mg, 0.075 mmol, 75%) as a white solid.

^{1}H NMR (400 MHz, $CDCl_3$) δ 4.78 (d, J = 11.1 Hz, 1H, NCH_2), 4.65 (d, J = 11.1 Hz, 1H, NCH_2), 4.56 (dd, J = 12.0, 2.0 Hz, 1H, NCH_2), 4.41 (dd, J = 12.0, 2.0 Hz, 1H, NCH_2), 1.50 (s, 3H, C(CH_3)$_2$), 1.48 (s, 3H, C(CH_3)$_2$) ppm. **^{13}C NMR** (126 MHz, $CDCl_3$) δ 202.7 (*C*=O), 156.2 (q, J = 38.2 Hz, F_3C(*C*=O)), 115.9 (q, J = 288 Hz, F_3*C*(C=O)), 105.6 (*C*(CH_3)$_2$), 92.3 (*spiro-C*), 61.4 (N*C*H_2), 59.0 (N*C*H_2), 23.0 (C(*C*H_3)$_2$), 23.0 (C(*C*H_3)$_2$) ppm. **^{19}F NMR** (377 MHz, $CDCl_3$) δ –72.7 ppm. (*see spectra*)

HRMS (m/z): (APCI) calc'd for $C_9H_{11}NO_3F_3$ [M+H]$^+$: 238.0686, found: 238.0680.

IR (thin film) ν_{max}: 2924, 2874, 1825 (C=O), 1701 (C=O), 1391, 1201, 1145, 924 cm^{-1}.

7-Methyl-2-(2,2,2-trifluoroacetyl)-5-oxa-2-azaspiro[3.4]octan-8-one: 4d

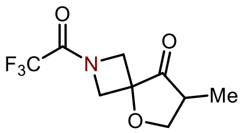

Synthesised according to General Procedure **G** from: **3d** (25.5 mg, 0.100 mmol, 1.00 equiv.), and TFAA (27.8 µL, 2.00 equiv.). Purified by flash column chromatography (SiO_2; 3:1 hexane:EtOAc) to afford **4d** (14.3 mg, 0.060 mmol, 60%) as a white solid.

TLC: R_f = 0.25 (3:1 hexane:EtOAc).

^{1}H NMR (400 MHz, $CDCl_3$) δ 4.58 – 4.41 (m, 2.5H, OCH_2, NCH_2), 4.38 – 4.26 (m, 1.5H, NCH_2), 4.21 (d, J = 11.4 Hz, 0.5H, NCH_2), 4.11 (d, J = 10.9 Hz, 0.5H, NCH_2), 3.69 (2×dd, J = 12.2, 9.2 Hz, 1H, OCH_2), 2.74 – 2.56 (m, 1H, (C=O)C*H*), 1.18 (2×d, J = 7.1 Hz, 3H, CHCH_3) ppm. **^{13}C NMR** (126 MHz, $CDCl_3$) δ 214.2 and 214.1 (*C*=O), 156.2 (q, J = 38.0 Hz, F_3C(*C*=O)), 116.0 (q, J = 288 Hz, F_3*C*(C=O)), 76.3 and 76.2 (*spiro-C*), 71.4 and 71.3 (O*C*H_2), 62.3 and 61.4 (N*C*H_2), 59.7 and 59.0 (N*C*H_2), 41.0 and 40.9 ((C=O)C*H*), 11.9 and 11.9 (CH*C*H_3) ppm. **^{19}F NMR** (377 MHz, $CDCl_3$) δ –72.6, –72.6 ppm. *Doubling of signals due to presence of rotamers*. (*see spectra*)

HRMS (m/z): (APCI) calc'd for $C_9H_{11}NO_3F_3$ $[M+H]^+$: 238.0686, found: 238.0677.

IR (thin film) ν_{max}: 2944, 1760 (C=O), 1695 (C=O), 1477, 1455, 1244, 1204, 1146 cm^{-1}.

7-Phenyl-2-(2,2,2-trifluoroacetyl)-5-oxa-2-azaspiro[3.4]octan-8-one: 4e

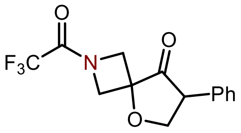

Synthesised according to General Procedure **G** from: **3e** (31.8 mg, 0.100 mmol, 1.00 equiv.), and TFAA (27.8 µL, 2.00 equiv.). Purified by flash column chromatography (SiO_2; 4:1 hexane:EtOAc) to afford **4e** (21.2 mg, 0.071 mmol, 71%) as a white solid.

TLC: R_f = 0.30 (4:1 hexane:EtOAc).

^{1}H NMR (500 MHz, $CDCl_3$) δ 7.40 – 7.30 (m, 3H, ArC*H*), 7.16 (d, *J* = 7.4 Hz, 2H, ArC*H*), 4.71 – 4.62 (m, 1.5H, OCH_2, NCH_2), 4.59 – 4.41 (m, 2H, NCH_2), 4.36 – 4.21 (m, 2.5H, (C=O)C*H*, NCH_2), 3.80 (2×dd, *J* = 12.2, 8.2 Hz, 1H, OCH_2) ppm. **^{13}C NMR** (126 MHz, $CDCl_3$) δ 210.9 and 210.9 (*C*=O), 156.26 (q, *J* = 37.8 Hz, F_3C(*C*=O)), 134.3 and 134.2 (Ar*C*), 129.4 and 129.4 (Ar*C*H), 128.3 and 128.3 (Ar*C*H), 127.9 and 127.9 (Ar*C*H), 116.0 (q, *J* = 288 Hz, F_3*C*(C=O)), 76.8 (*spiro-C*), 71.0 and 71.0 (O*C*H_2), 62.2 and 61.4 (N*C*H_2), 59.7 and 59.1 (N*C*H_2), 52.1 and 52.0 ((C=O)C*H*) ppm. **^{19}F NMR** (377 MHz, $CDCl_3$) δ –72.6, –72.6 ppm. *Doubling of signals due to presence of rotamers*. (*see spectra*)

HRMS (m/z): (APCI) calc'd for $C_{14}H_{12}NO_3F_3$ $[M+H]^+$: 300.0842, found: 300.0830.

IR (thin film) ν_{max}: 2951, 1759 (C=O), 1697 (C=O), 1476, 1452, 1250, 1205, 1148 cm^{-1}.

7,7-Dimethyl-2-(2,2,2-trifluoroacetyl)-5-oxa-2-azaspiro[3.4]octan-8-one: 4f

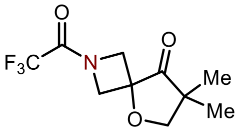

Synthesised according to General Procedure **G** from: **3f** (27.0 mg, 0.100 mmol, 1.00 equiv.), and TFAA (27.8 µL, 2.00 equiv.). Purified by flash column chromatography (SiO_2; 4:1 hexane:EtOAc) to afford **4f** (21.3 mg, 0.085 mmol, 85%) as a white solid.

TLC: R_f = 0.28 (4:1 hexane:EtOAc).

^{1}H NMR (500 MHz, $CDCl_3$) δ 4.54 (d, *J* = 10.1 Hz, 1H, NCH_2), 4.42 (d, *J* = 10.1 Hz, 1H, NCH_2), 4.31 (d, *J* = 11.0 Hz, 1H, NCH_2), 4.18 (d, *J* = 11.0 Hz, 1H, NCH_2), 3.94 (d, *J* = 9.3 Hz, 1H, OCH_2), 3.91 (d, *J* = 9.3 Hz, 1H, OCH_2), 1.16 (s, 3H, C(CH_3)$_2$), 1.14 (s, 3H, C(CH_3)$_2$) ppm.

^{13}C NMR (126 MHz, $CDCl_3$) δ 216.2 (*C*=O), 156.2 (q, *J* = 37.9 Hz, F_3C(*C*=O)), 116.0 (q, *J* = 288 Hz, F_3*C*(C=O)), 77.5 (O*C*H_2), 76.3 (*spiro-C*), 62.0 (q, *J* = 2.2 Hz, N*C*H_2), 59.5 (N*C*H_2), 44.1 (*C*(CH_3)$_2$), 21.7 (C(*C*H_3)$_2$), 21.7 (C(*C*H_3)$_2$) ppm. **^{19}F NMR** (377 MHz, $CDCl_3$) δ –72.6 ppm. (see spectra)

HRMS (m/z): (APCI) calc'd for $C_{10}H_{12}NO_3F_3$ $[M+H]^+$: 222.0842, found: 222.0832.

IR (thin film) ν_{max}: 2924, 2852, 1761 (C=O), 1698 (C=O), 1466, 1257, 1145, 1041, 977 cm^{-1}.

6-Methyl-2-(2,2,2-trifluoroacetyl)-5-oxa-2-azaspiro[3.4]octan-8-one: 4g

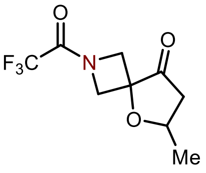

Synthesised according to General Procedure **G** from: **3g** (21.3 mg, 0.100 mmol, 1.00 equiv.), and TFAA (27.8 µL, 2.00 equiv.). Purified by flash column chromatography (SiO_2; 3:1 hexane:EtOAc) to afford **4g** (19.5 mg, 0.082 mmol, 82%) as a colourless oil.

TLC: R_f = 0.20 (3:1 hexane:EtOAc).

^{1}H NMR (400 MHz, $CDCl_3$) δ 4.60 – 4.48 (m, 1H, NC*H*$_2$), 4.46 – 4.32 (m, 2.5H, NC*H*$_2$, C*H*CH_3), 4.28 (d, *J* = 10.9 Hz, 0.5H, NC*H*$_2$), 4.18 (d, *J* = 11.9 Hz, 0.5H, NC*H*$_2$), 4.10 (d, *J* = 10.9 Hz, 0.5H, NC*H*$_2$), 2.68 (2×dd, *J* = 18.3, 6.1 Hz, 1H), 2.23 (2×dd, *J* = 18.3, 8.6 Hz, 1H), 1.42 (2×d, *J* = 6.2 Hz, 3H, CHC*H*$_3$) ppm. **^{13}C NMR** (126 MHz, $CDCl_3$) δ 206.0 and 205.9 (*C*=O), 152.4 (2×q, *J* = 36.1 Hz, F_3C(*C*=O)), 114.2 (q, *J* = 274 Hz, F_3*C*(C=O)), 77.1 and 77.1 (*spiro-C*), 72.7 and 72.5 (O*C*HCH_3), 63.1 (q, *J* = 2.0 Hz, N*C*H_2) and 62.2 (q, *J* = 2.0 Hz, N*C*H_2), 60.7 and 59.9 (N*C*H_2), 44.7 and 44.6 ((C=O)*C*H_2), 24.1 and 24.1 (CH*C*H_3) ppm. **^{19}F NMR** (377 MHz, $CDCl_3$) δ –72.6, –72.7 ppm. *Doubling of signals due to presence of rotamers*. (see spectra)

HRMS (m/z): (APCI) calc'd for $C_9H_{11}NO_3F_3$ $[M+H]^+$: 238.0686, found: 238.0681.

IR (thin film) ν_{max}: 2963, 1762 (C=O), 1695 (C=O), 1455, 1257, 1142, 1013 cm^{-1}.

6-Cyclopropyl-2-(2,2,2-trifluoroacetyl)-5-oxa-2-azaspiro[3.4]octan-8-one: 4h

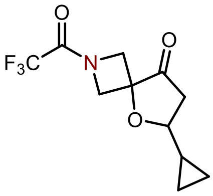

Synthesised according to General Procedure **G** from: **3h** (23.9 mg, 0.100 mmol, 1.00 equiv.), and TFAA (27.8 µL, 2.00 equiv.). Purified by flash column chromatography (SiO_2; 99:1 CH_2Cl_2:Et_2O) to afford **4h** (9.30 mg, 0.035 mmol, 35%) as a colourless oil.

TLC: R_f = 0.29 (2.3:1 hexane:EtOAc).

^{1}H NMR (400 MHz, $CDCl_3$) δ 4.63 – 4.43 (m, 1.5H, NC*H*$_2$), 4.41 – 4.31 (m, 1H, NC*H*$_2$), 4.31 – 4.20 (m, 1H, NC*H*$_2$), 4.1 (d, *J* = 10.9 Hz, 0.5H, NC*H*$_2$), 3.79 – 3.71 (m, 1H, OC*H*), 2.70 (2×dd, *J* = 18.3, 6.5 Hz, 1H, (C=O)C*H*$_2$), 2.43 (2×dd, *J* = 18.3, 7.9 Hz, 1H, (C=O)C*H*$_2$), 1.10 – 0.94 (m, 1H, cyclopropane C*H*), 0.75 – 0.56 (m, 2H, cyclopropane C*H*$_2$), 0.51 – 0.38 (m, 1H, cyclopropane C*H*$_2$), 0.37 – 0.24 (m, 1H, cyclopropane C*H*$_2$) ppm. **^{13}C NMR** (126 MHz, $CDCl_3$) δ 212.2 and 212.1 (*C*=O), 156.2 (2×q, *J* = 37.9 Hz, F_3C(*C*=O)), 116.03 (q, *J* = 288 Hz, F_3*C*(C=O)), 80.4 and 80.3 (O*C*H), 62.2 (q, *J* = 2.1 Hz, N*C*H$_2$) and 61.6 (q, *J* = 2.1 Hz, N*C*H$_2$), 59.7 and 59.3 (N*C*H$_2$), 41.2 ((C=O)*C*H$_2$), 15.5 and 15.5 (cyclopropane *C*H), 3.3 and 3.3 (cyclopropane *C*H$_2$), 2.0 and 1.9 (cyclopropane *C*H$_2$) ppm. *Spiro carbon obscured by solvent residual signal.* **^{19}F NMR** (377 MHz, $CDCl_3$) δ –72.6 ppm. *Doubling of signals due to presence of rotamers*. (*see spectra*)

HRMS (m/z): (APCI) calc'd for $C_{11}H_{12}NO_3F_3$ $[M+H]^+$: 264.0842, found: 264.0832.

IR (thin film) ν_{max}: 2961, 1758 (C=O), 1696 (C=O), 1478, 1242, 1143 cm^{-1}.

6-Isopropyl-2-(2,2,2-trifluoroacetyl)-5-oxa-2-azaspiro[3.4]octan-8-one: 4i

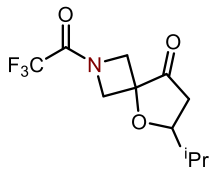

Synthesised according to General Procedure **G** from: **3i** (24.1 mg, 0.100 mmol, 1.00 equiv.), and TFAA (27.8 μL, 2.00 equiv.). Purified by flash column chromatography (SiO_2; 4.7:1 hexane:EtOAc) to afford **4i** (24.9 mg, 0.094 mmol, 94%) as a colourless oil.

TLC: R_f = 0.24 (4.7:1 hexane:EtOAc).

^{1}H NMR (500 MHz, $CDCl_3$) δ 4.60 – 4.48 (m, 1H, NC*H*$_2$), 4.43 (d, *J* = 10.3 Hz, 0.5H, NC*H*$_2$), 4.39 – 4.31 (m, 1H, NC*H*$_2$), 4.28 (d, *J* = 10.8 Hz, 0.5H, NC*H*$_2$), 4.19 (d, *J* = 11.1 Hz, 0.5H, NC*H*$_2$), 4.10 (d, *J* = 10.8 Hz, 0.5H, NC*H*$_2$), 4.02 – 3.86 (m, 1H, OC*H*), 2.59 (2×dd, *J* = 18.4, 6.3 Hz, 1H, (C=O)C*H*$_2$), 2.32 (2×dd, *J* = 18.4, 8.9 Hz, 1H, (C=O)C*H*$_2$), 1.86 – 1.78 (m, 1H, C*H*(CH$_3$)$_2$), 1.03 (2×d, *J* = 6.8 Hz, 3H, CH(C*H*$_3$)$_2$), 0.93 (d, *J* = 6.8 Hz, 3H, CH(C*H*$_3$)$_2$) ppm. **^{13}C NMR** (126 MHz, $CDCl_3$) δ 212.3 and 212.2 (*C*=O), 156.2 (q, *J* = 38.0 Hz, F_3C(*C*=O)), 116.1 (q, *J* = 288 Hz, F_3*C*(C=O)), 81.5 and 81.2 (O*C*H), 62.5 (q, *J* = 2.2 Hz, N*C*H$_2$) and 61.2 (q, *J* = 2.2 Hz, N*C*H$_2$), 60.0 and 58.9 (N*C*H$_2$), 39.4 and 39.3 ((C=O)*C*H$_2$), 33.4 and 33.3 (*C*H(CH$_3$)$_2$), 18.4 and 18.4 (CH(*C*H$_3$)$_2$), 18.1 and 18.0 (CH(*C*H$_3$)$_2$) ppm. *Spiro carbon*

obscured by solvent residual signal. **^{19}F NMR** (377 MHz, $CDCl_3$) δ –72.6 ppm. *Doubling of signals due to presence of rotamers*. (see spectra)

HRMS (m/z): (APCI) calc'd for $C_{11}H_{14}NO_3F_3$ $[M+H]^+$: 266.0999, found: 266.0993.

IR (thin film) ν_{max}: 2951, 2877, 1760 (C=O), 1694 (C=O), 1471, 1248, 1141, 1080 cm^{-1}.

6-Phenyl-2-(2,2,2-trifluoroacetyl)-5-oxa-2-azaspiro[3.4]octan-8-one: 4j

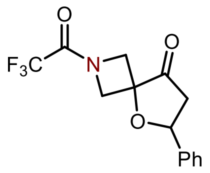

Synthesised according to General Procedure **G** from: **3j** (27.5 mg, 0.100 mmol, 1.00 equiv.), and TFAA (27.8 µL, 2.00 equiv.). Purified by flash column chromatography (SiO_2; 4:1 hexane:EtOAc) to afford **4j** (12.6 mg, 0.042 mmol, 42%) as a colourless oil.

TLC: R_f = 0.20 (4:1 hexane:EtOAc).

^{1}H NMR (400 MHz, $CDCl_3$) δ 7.46 – 7.33 (m, 5H, ArC*H*), 5.31 (2×dd, *J* = 8.9, 6.5 Hz, 1H, OC*H*), 4.63 (d, *J* = 10.2 Hz, 1H, NCH_2), 4.54 – 4.43 (m, 1H, NCH_2), 4.40 (d, *J* = 11.0 Hz, 1H, NCH_2), 4.24 (2×dd, *J* = 10.6, 1.5 Hz, 1H, NCH_2), 3.00 (2×dd, *J* = 18.5, 6.5 Hz, 1H, (C=O)CH_2), 2.69 (2×dd, *J* = 18.5, 8.9 Hz, 1H, (C=O)CH_2) ppm. **^{13}C NMR** (126 MHz, $CDCl_3$) δ 211.4 and 211.3 (*C*=O), 156.3 (2×q, *J* = 38.2 Hz, F_3C(*C*=O)), 138.9 and 138.8 (Ar*C*), 129.2 (Ar*C*H), 129.1 and 129.1 (Ar*C*H), 126.0 and 126.0 (Ar*C*H), 116.0 (q, *J* = 288 Hz, F_3*C*(C=O)), 62.3 (q, *J* = 2.2 Hz, N*C*H_2) and 61.3 (q, *J* = 2.2 Hz, N*C*H_2) 59.9 and 59.0 (N*C*H_2), 43.10 ((C=O)*C*H_2) ppm. *Spiro carbon and benzylic carbon obscured by solvent residual signal.* **^{19}F NMR** (377MHz, $CDCl_3$) δ –72.6, –72.6 ppm. *Doubling of signals due to presence of rotamers*. (see spectra)

HRMS (m/z): (APCI) calc'd for $C_{14}H_{12}NO_3F_3$ $[M+H]^+$: 300.0842, found: 300.0841.

IR (thin film) ν_{max}: 2955, 1761 (C=O), 1696 (C=O), 1454, 1250, 1145, 1092 cm^{-1}.

3-(8-Oxo-2-(2,2,2-trifluoroacetyl)-5-oxa-2-azaspiro[3.4]octan-6-yl)pyridin-1-ium 2,2,2-trifluoroacetate: 4k

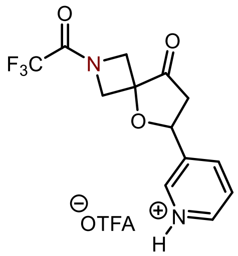

Synthesised according to a modified General Procedure **G** from: **3k** (27.6 mg, 0.100 mmol, 1.00 equiv.), and TFAA (27.8 µL, 2.00 equiv.). The crude trifluoroacetic acid salt was then washed with cold CH_2Cl_2

(4×), carefully removing the solvent with a pasteur pipette to afford **4k** (31.5 mg, 0.076 mmol, 76%) as a pale-brown solid.

TLC: R_f = 0.25 (EtOAc).

^{1}H NMR (400 MHz, CD_3OD) δ 8.88 (s, 1H, ArC*H*), 8.73 (d, *J* = 5.6 Hz, 1H, ArC*H*), 8.58 (d, *J* = 8.2 Hz, 1H, ArC*H*), 8.01 – 7.97 (m, 1H ArC*H*), 5.43 (dd, *J* = 8.2, 4.6 Hz, 1H, OC*H*), 4.72 (d, *J* = 10.4 Hz, 1H, NCH_2), 4.40 (2×dd, *J* = 11.1, 1.3 Hz, 1H, NCH_2), 4.26 (2×d, *J* = 10.4 Hz, 1H, NCH_2), 3.97 (2×d, *J* = 11.1 Hz, 1H, NCH_2), 3.35 – 3.26 (m, 1H, (C=O)CH_2), 3.15 (2×dd, *J* = 17.5, 4.6 Hz, 1H, (C=O)CH_2) ppm. **^{13}C NMR** (126 MHz, CD_3OD) δ 208.1 and 208.0 (*C*=O), 162.4 (q, *J* = 35.4 Hz, *counterion*-F_3C(*C*=O)), 157.6 (q, *J* = 37.3 Hz, F_3C(*C*=O)), 146.4 (Ar*C*), 144.4 (Ar*C*H), 142.4 (Ar*C*H), 141.8 (Ar*C*H), 127.9 (Ar*C*H), 117.9 (q, *J* = 287 Hz, *counterion*-F_3*C*(C=O)), 117.5 (q, *J* = 287 Hz, F_3*C*(C=O)), 75.4 (*spiro-C*), 67.3 (O*C*H), 63.6 (q, *J* = 2.0 Hz, N*C*H_2) and 63.3 (q, *J* = 2.0 Hz, N*C*H_2), 60.8 and 60.60 (N*C*H_2), 46.6 ((C=O)*C*H_2) ppm. **^{19}F NMR** (377 MHz, CD_3OD) δ –74.2, –74.2, –76.9 (*counterion*) ppm. *Doubling of signals due to presence of rotamers*. (*see spectra*)

HRMS (m/z): (APCI) calc'd for $C_{13}H_{11}N_2O_3F_3$ $[M+H]^+$: 301.0795, found: 301.0790.

IR (thin film) ν_{max}: 3301 (N-H), 2944, 2928, 2038, 2030, 1685 (C=O), 1676 (C=O), 1471, 1254, 1201, 1138 cm^{-1}.

2-(2,2,2-Trifluoroacetyl)-6-vinyl-5-oxa-2-azaspiro[3.4]octan-8-one: 4l

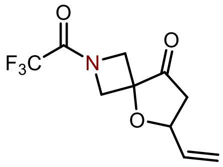

Synthesised according to General Procedure **G** from: **3l** (22.5 mg, 0.100 mmol, 1.00 equiv.), and TFAA (27.8 µL, 2.00 equiv.). Purified by flash column chromatography (SiO_2; 3:1 hexane:EtOAc) to afford **4l** (23.3 mg, 0.094 mmol, 94%) as a colourless oil.

TLC: R_f = 0.31 (3:1 hexane:EtOAc).

^{1}H NMR (400 MHz, $CDCl_3$) δ 5.93 (2×ddd, *J* = 17.2, 10.6, 4.6 Hz, 1H, *H*C=CH_2), 5.40 (d, *J* = 17.2 Hz, 1H, HC=CH_2), 5.35 – 5.32 (m, 1H, HC=CH_2), 4.82 – 4.70 (m, 1H, O*C*H), 4.61 – 4.52 (m, 1H, NCH_2), 4.47 – 4.29 (m, 2H, NCH_2), 4.22 (dd, *J* = 11.0, 1.8 Hz, 0.5H, NCH_2), 4.16 (dd, *J* = 11.0, 1.8 Hz, 0.5H, NCH_2), 2.76 (2×dd, *J* = 18.1, 6.8 Hz, 1H, (C=O)CH_2), 2.46 (2×dd, *J* = 18.5, 8.1 Hz, 1H, (C=O)CH_2) ppm. **^{13}C NMR** (126 MHz, $CDCl_3$) δ 211.4 and 211.3 (*C*=O), 156.22 (2×q, *J* = 38.1 Hz, F_3C(*C*=O)), 135.9 (H*C*=CH_2), 118.9 and 118.9 (HC=*C*H_2), 116.0

(q, J = 288 Hz, F_3C(C=O)), 76.7 (*spiro*-*C*), 76.6 and 76.4 (O*C*H), 62.2 (q, J = 2.1 Hz, NCH_2) and 61.6 (q, J = 2.1 Hz, NCH_2), 59.8 and 59.2 (NCH_2), 41.1 ((C=O)CH_2) ppm. **^{19}F NMR** (377 MHz, $CDCl_3$) δ –72.6, –72.6 ppm. *Doubling of signals due to presence of rotamers*. (see spectra)

HRMS (m/z): (APCI) calc'd for $C_{10}H_{10}NO_3F_3$ $[M+H]^+$: 250.0686, found: 250.0682.

IR (thin film) ν_{max}: 2952, 1760 (C=O), 1694 (C=O), 1476, 1254, 1144, 1013 cm^{-1}.

6-(Chloromethyl)-2-(2,2,2-trifluoroacetyl)-5-oxa-2-azaspiro[3.4]octan-8-one: 4m

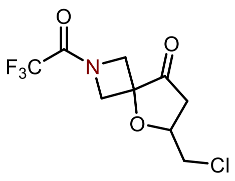

Synthesised according to General Procedure **G** from: **3m** (24.8 mg, 0.100 mmol, 1.00 equiv.), and TFAA (27.8 µL, 2.00 equiv.). Purified by flash column chromatography (SiO_2; 2.3:1 hexane:EtOAc) to afford **4m** (12.2 mg, 0.045 mmol, 45%) as a white solid.

TLC: R_f = 0.13 (3:1 hexane:EtOAc).

^{1}H NMR (400 MHz, $CDCl_3$) δ 4.89 – 4.74 (m, 1H, OC*H*), 4.60 – 4.44 (m, 1.5H, NCH_2), 4.42 – 4.21 (m, 2H, NCH_2), 4.15 (d, J = 11.1 Hz, 0.5H, NCH_2), 3.85 (2×dd, J = 11.8, 3.6 Hz, 1H, CH_2Cl), 3.67 (2×dd, J = 11.8, 3.0 Hz, 1H, CH_2Cl), 2.79 (2×dd, J = 18.8, 8.0 Hz, 1H, (C=O)CH_2), 2.65 (2×dd, J = 18.8, 5.1 Hz, 1H, (C=O)CH_2) ppm. **^{13}C NMR** (126 MHz, $CDCl_3$) δ 210.3 (*C*=O), 156.0 (2×q, J = 38.2 Hz, F_3C(*C*=O)), 116.0 (q, J = 288 Hz, F_3C(C=O)), 77.3 and 77.3 (*spiro*-*C*), 74.9 and 74.7 (O*C*H), 63.0 (q, J = 2.2 Hz, NCH_2) and 61.97 (q, J = 2.2 Hz, NCH_2), 60.6 and 59.6 (NCH_2), 48.0 and 47.7 (CH_2Cl), 38.2 and 38.1 ((C=O)CH_2) ppm. **^{19}F NMR** (377 MHz, $CDCl_3$) δ –72.6, –72.6 ppm. *Doubling of signals due to presence of rotamers*. (see spectra)

HRMS (m/z): (APCI) calc'd for $C_9H_9NO_3ClF_3$ $[M+H]^+$: 272.0296, found: 272.0288.

IR (thin film) ν_{max}: 2964, 1762 (C=O), 1695 (C=O), 1478, 1455, 1249, 1144, 1033 cm^{-1}.

6-((Benzyloxy)methyl)-2-(2,2,2-trifluoroacetyl)-5-oxa-2-azaspiro[3.4]octan-8-one: 4n

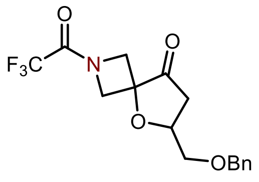

Synthesised according to General Procedure **G** from: **3n** (32.0 mg, 0.100 mmol, 1.00 equiv.), and TFAA (27.8 µL, 2.00 equiv.). Purified by flash column chromatography (SiO_2; 3:1 hexane:EtOAc) to afford **4n** (14.1 mg, 0.041 mmol, 41%) as a colourless oil.[A]

Notes: (**A**) Also observed competing cyclization from the benzyl ether fragment which undergoes spirocyclization-debenzylation to give 2-(2,2,2-trifluoroacetyl)-7-((trimethylsilyl) oxy)-5-oxa-2-azaspiro[3.5]nonan-9-one in 15% NMR yield.

TLC: R_f = 0.31 (3:1 hexane:EtOAc).

^{1}H NMR (500 MHz, $CDCl_3$) δ 7.39 – 7.29 (m, 3H, ArC*H*), 7.26 – 7.20 (m, 2H, ArC*H*), 4.67 – 4.58 (m, 1H, O*C*H), 4.56 – 4.42 (m, 2.5H, OC*H*$_2$Ph, NC*H*$_2$), 4.34 (d, *J* = 10.2 Hz, 0.5H, NC*H*$_2$), 4.27 – 4.17 (m, 2H, NC*H*$_2$), 4.12 – 4.05 (m, 1H, NC*H*$_2$), 3.76 (2×dd, *J* = 10.0, 2.2 Hz, 1H, CHC*H*$_2$O), 3.45 (2×dd, *J* = 10.0, 2.4 Hz, 1H, CHC*H*$_2$O), 2.67 (2×dd, *J* = 18.1, 8.7 Hz, 1H, (C=O)C*H*$_2$), 2.51 (2×dd, *J* = 18.1, 3.0 Hz, 1H, (C=O)C*H*$_2$) ppm. **^{13}C NMR** (126 MHz, $CDCl_3$) δ 211.6 (*C*=O), 156.1 (2×q, *J* = 37.7 Hz, F_3C(*C*=O)), 136.9 and 136.7 (Ar*C*), 128.8 and 128.8 (Ar*C*H), 128.5 and 128.4 (Ar*C*H), 128.3 and 128.0 (Ar*C*H), 116.1 (2×q, *J* = 288 Hz, F_3*C*(C=O)), 76.83 (*spiro-C*), 75.2 and 75.1 (*C*HO), 74.2 and 74.0 (O*C*H$_2$Ph), 72.8 and 72.5 (CH*C*H$_2$O), 64.2 (d, *J* = 2.2 Hz, N*C*H$_2$) and 61.5 (d, *J* = 2.2 Hz, N*C*H$_2$), 61.5 and 58.9 (N*C*H$_2$), 37.9 ((C=O)*C*H$_2$) ppm. **^{19}F NMR** (377 MHz, $CDCl_3$) δ –72.7 ppm. *Doubling of signals due to presence of rotamers*. (*see spectra*)

HRMS (m/z): (APCI) calc'd for $C_{16}H_{16}NO_4F_3$ $[M+H]^+$: 344.1104, found: 344.1104.

IR (thin film) ν_{max}: 2944, 2864, 1761 (C=O), 1697 (C=O), 1453, 1246, 1204, 1145 cm^{-1}.

6,6-Dimethyl-2-(2,2,2-trifluoroacetyl)-5-oxa-2-azaspiro[3.4]octan-8-one: 4o

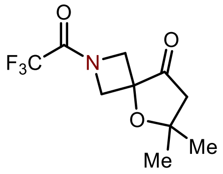

Synthesised according to General Procedure **G** from: **3o** (22.7 mg, 0.100 mmol, 1.00 equiv.), and TFAA (27.8 µL, 2.00 equiv.). Purified by flash column chromatography (SiO_2; 3:1 to 9:1 CH_2Cl_2:hexane) to afford **4o** (19.6 mg, 0.078 mmol, 78%) as a white solid.

TLC: R_f = 0.30 (4:1 hexane:EtOAc).

^{1}H NMR (400 MHz, $CDCl_3$) δ 4.59 – 4.56 (m, 1H, NC*H*$_2$), 4.43 – 4.31 (m, 2H, NC*H*$_2$), 4.14 (dd, *J* = 11.2, 1.7 Hz, 1H, NC*H*$_2$), 2.52 – 2.42 (m, 2H, (C=O)C*H*$_2$), 1.38 (s, 3H, C(C*H*$_3$)$_2$), 1.36 (s, 3H, C(C*H*$_3$)$_2$) ppm. **^{13}C NMR** (126 MHz, $CDCl_3$) δ 213.3 (*C*=O), 156.1 (q, *J* = 37.9 Hz, F_3C(*C*=O)), 116.0 (q, *J* = 288 Hz, F_3*C*(C=O)), 79.8 (O*C*(CH$_3$)$_2$), 76.7 (q, *J* = 1.5 Hz, *spiro-C*), 62.9 (q, *J* = 2.1 Hz, N*C*H$_2$), 60.5 (N*C*H$_2$), 48.8 ((C=O)*C*H$_2$), 28.4 (C(*C*H$_3$)$_2$), 28.4 (C(*C*H$_3$)$_2$) ppm. **^{19}F NMR** (377 MHz, $CDCl_3$) δ –72.6 ppm. (*see spectra*)

HRMS (m/z): (APCI) calc'd for $C_{10}H_{12}NO_3F_3$ $[M+H]^+$: 222.0842, found: 222.0831.

IR (thin film) ν_{max}: 2978, 2931, 2852, 1762 (C=O), 1698 (C=O), 1476, 1456, 1257, 1222, 1147, 972 cm^{-1}.

2-(2,2,2-Trifluoroacetyl)-5-oxa-2-azadispiro[3.1.5^6.2^4]tridecan-13-one: 4p

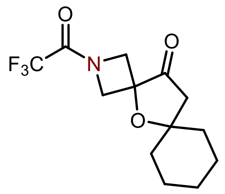

Synthesised according to General Procedure **G** from: **3p** (26.7 mg, 0.100 mmol, 1.00 equiv.), and TFAA (27.8 µL, 2.00 equiv.). Purified by flash column chromatography (SiO_2; 4:1 hexane:EtOAc) to afford **4p** (26.2 mg, 0.090 mmol, 90%) as colourless crystals.

TLC: R_f = 0.30 (4:1 hexane:EtOAc).

^{1}H NMR (400 MHz, $CDCl_3$) δ 4.57 – 4.53 (m, 1H, NCH_2), 4.37 (d, J = 10.1 Hz, 1H, NCH_2), 4.33 (d, J = 10.9 Hz, 1H, NCH_2), 4.15 (d, J = 10.9 Hz, 1H, NCH_2), 2.49 – 2.37 (m, 2H, (C=O)CH_2), 1.83 – 1.71 (m, 2H, cy-CH_2), 1.70 – 1.36 (m, 8H, cy-CH_2) ppm. **^{13}C NMR** (126 MHz, $CDCl_3$) δ 213.3 (*C*=O), 156.1 (q, J = 37.8 Hz, F_3C(*C*=O)), 116.1 (q, J = 288 Hz, F_3*C*(C=O)), 81.9 (cy *spiro*-*C*), 76.2 (azetidine *spiro*-*C*), 62.8 (q, J = 2.0 Hz, N*C*H_2), 60.4 (N*C*H_2), 47.4 ((C=O)*C*H_2), 37.5 (cy-*C*H_2), 37.5 (cy-*C*H_2), 25.0, (cy-*C*H_2), 22.9 (cy-*C*H_2), 22.9 (cy-*C*H_2) ppm. **^{19}F NMR** (377 MHz, $CDCl_3$) δ –72.6 ppm. (*see spectra*)

HRMS (m/z): (APCI) calc'd for $C_{13}H_{16}NO_3F_3$ $[M+H]^+$: 292.1155, found: 292.1152.

IR (thin film) ν_{max}: 2937, 2862, 1760 (C=O), 1700 (C=O), 1454, 1249, 1146, 1033 cm^{-1}.

Stereoretention study

The stereoretentive nature of the electrophile induced spirocyclization-desilylation reaction was determined by analysis of the crude reaction mixtures of substrates **3q**. No change in the diastereomeric ratio (dr) was observed from either *syn*- or *anti*-**3q** (Figure S1, *see procedure*), demonstrating that both α- and β-carbonyl carbons retain their stereochemical integrity. In the case of *syn*-**4q**, minor levels of epimerization could be observed when the reaction was quenched with MeOH, presumably from the generation of TFA and subsequent racemization of the α-carbon. However, this could be easily prevented by avoiding the use of MeOH in this step.

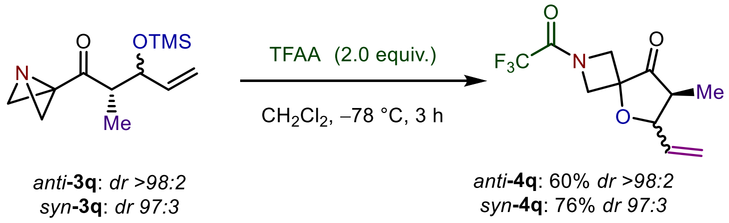

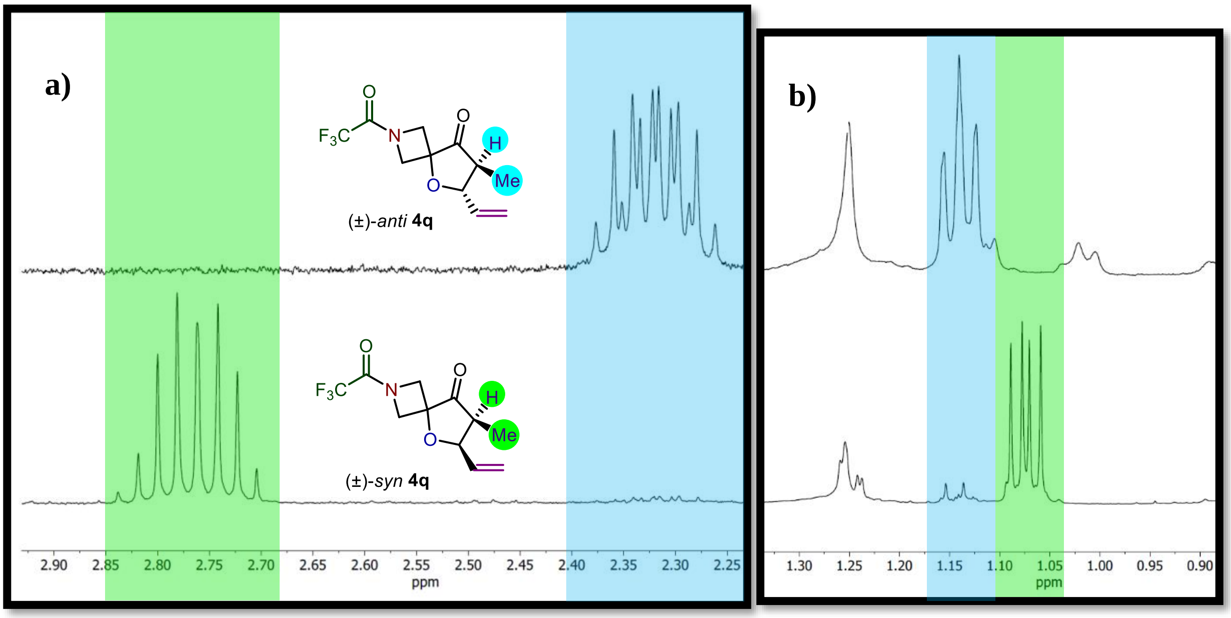

Figure S1. Comparison of the crude ^{1}H NMR spectra of the spirocyclization-desilylation reaction from *anti*-**3q** (top) and *syn*-**3q** (bottom). a) α-H signal. b) α-Methyl signal. No change in the dr from the corresponding ABB-ketones was observed in either crude reactions.

anti-7-Methyl-2-(2,2,2-trifluoroacetyl)-6-vinyl-5-oxa-2-azaspiro[3.4]octan-8-one: *anti*-4q

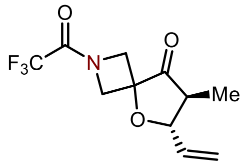

Synthesised according to General Procedure **G** from: *anti*-**3q** (>98:2 dr, 23.9 mg, 0.100 mmol, 1.00 equiv.), and TFAA (27.8 µL, 2.00 equiv.). Crude ^{1}H NMR analysis showed the dr to be >98:2 for the *anti*-diastereomer (*see above*). Small amounts of epimerisation of the spirocyclic product was observed during flash column chromatography (SiO_2; 4:1 hexane:EtOAc) to afford **4q** as a 96:4 *anti*:*syn* ratio of diastereomers (15.8 mg, 0.060 mmol, 60%) as a white solid.

TLC: R_f = 0.30 (4:1 hexane:EtOAc).

^{1}H NMR (400 MHz, $CDCl_3$) *anti*-diastereomer δ 5.93 (2×ddd, *J* = 17.2, 10.3, 5.6 Hz, 1H, *H*C=CH$_2$), 5.46 (2×app-dt, *J* = 17.2, 1.1 Hz, 1H, HC=C*H*$_2$), 5.39 (d, *J* = 10.3 Hz, 1H, HC=C*H*$_2$),

4.61 – 4.47 (m, 1.5H, NC*H*$_2$), 4.39 – 4.25 (m, 2H, NC*H*$_2$), 4.17 – 4.03 (m, 1.5H, NC*H*$_2$, OC*H*), 2.37 – 2.26 (m, 1H, C*H*CH$_3$), 1.14 (2×d, *J* = 7.0 Hz, 3H, CHC*H*$_3$) ppm. **^{13}C NMR** (126 MHz, CDCl$_3$) *anti*-diastereomer δ 213.6 and 213.5 (*C*=O), 156.2 (q, *J* = 37.5 Hz, F$_3$C(*C*=O)), 135.0 and 135.0 (H*C*=CH$_2$), 119.9 (HC=*C*H$_2$), 116.0 (q, *J* = 288 Hz, F$_3$*C*(C=O)), 84.0 and 83.7 (*C*HO), 76.8 (q, *J* = 1.5 Hz, *spiro*-*C*), 62.7 (q, *J* = 2.2 Hz, N*C*H$_2$) and 61.4 (q, *J* = 2.2 Hz, N*C*H$_2$), 60.1 and 59.0 (N*C*H$_2$), 47.0 and 47.0 (*C*HCH$_3$), 10.3 and 10.2 (CHC*H*$_3$) ppm. **^{19}F NMR** (377 MHz, CDCl$_3$) *anti*-diastereomer δ –72.6, –72.6 ppm. *Doubling of signals due to presence of rotamers*. (*see spectra*)

HRMS (m/z): (APCI) calc'd for $C_{11}H_{12}NO_3F_3$ [M+H]$^+$: 264.0842, found: 264.0842.

IR (thin film) ν$_{max}$: 2943, 1761 (C=O), 1698 (C=O), 1455, 1246, 1148, 1009 cm^{-1}.

***syn*-7-Methyl-2-(2,2,2-trifluoroacetyl)-6-vinyl-5-oxa-2-azaspiro[3.4]octan-8-one: *syn*-4q**

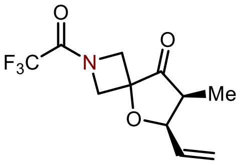

Synthesised according to a modified General Procedure **G** from: *syn*-**3q** (97:3 dr, 23.9 mg, 0.100 mmol, 1.00 equiv.), and TFAA (27.8 µL, 2.00 equiv.). After stirring at –78 °C for 3 h the solvent was removed under vacuum whilst warming to rt. Crude ^{1}H NMR analysis showed the dr to be 97:3 for the *syn*-diastereomer (*see above*). Epimerisation of the spirocyclic product was observed during flash column chromatography (SiO$_2$; 4:1 hexane:EtOAc) to afford **4q** as an 83:17 *anti*:*syn* ratio of diastereomers (20.1 mg, 0.076 mmol, 76%) as a white solid.

TLC: R$_f$ = 0.27 (4:1 hexane:EtOAc).

^{1}H NMR (400 MHz, CDCl$_3$) *syn*-diastereomer δ 5.74 – 5.54 (m, 1H, *H*C=CH$_2$), 5.46 – 5.33 (m, 2H, HC=C*H*$_2$), 4.89 – 4.83 (m, 1H, OC*H*), 4.58 – 4.46 (m, 1H, NC*H*$_2$), 4.47 – 4.40 (m, 1H, NC*H*$_2$), 4.32 – 4.26 (m, 1H, NC*H*$_2$), 4.21 (d, *J* = 11.1 Hz, 1H, NC*H*$_2$), 2.83 – 2.68 (m, 1H, C*H*CH$_3$), 1.07 (2×d, *J* = 7.4 Hz, 3H, CHC*H*$_3$) ppm. **^{13}C NMR** (126 MHz, CDCl$_3$) *syn*-diastereomer δ 214.1 and 214.0 (*C*=O), 156.2 (q, *J* = 37.5 Hz, F$_3$C(*C*=O)), 132.9 and 132.9 (H*C*=CH$_2$), 120.0 (HC=*C*H$_2$), 116.0 (q, *J* = 288 Hz, F$_3$*C*(C=O)), 80.8 and 80.8 (*C*HO), 75.8 and 75.8 (*spiro*-*C*), 62.4 (q, *J* = 2.1 Hz, N*C*H$_2$) and 62.3 (q, *J* = 2.1 Hz, N*C*H$_2$), 60.0 and 60.0 (N*C*H$_2$), 44.5 and 44.4 (*C*HCH$_3$), 10.1 and 10.0 (CHC*H*$_3$) ppm. **^{19}F NMR** (377 MHz, CDCl$_3$) *syn*-diastereomer δ –72.6, –72.6 ppm. *Doubling of signals due to presence of rotamers*. (*see spectra*)

6-Methyl-2-(2,2,2-trifluoroacetyl)-5-oxa-2-azaspiro[3.5]nonan-9-one: 4r

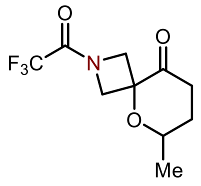

Synthesised according to General Procedure **G** from: **3r** (22.7 mg, 0.100 mmol, 1.00 equiv.), and TFAA (27.8 µL, 2.00 equiv.). Purified by flash column chromatography (SiO_2; 1.9:1 hexane:EtOAc) to afford **4r** (22.7 mg, 0.090 mmol, 90%) as a colourless oil.

TLC: R_f = 0.28 (1.9:1 hexane:EtOAc).

^{1}H NMR (400 MHz, $CDCl_3$) δ 4.98 – 4.95 (m, 0.6H, NC*H*$_2$), 4.69 (d, *J* = 10.9 Hz, 0.4H, NC*H*$_2$), 4.44 – 4.31 (m, 0.8H, NC*H*$_2$), 4.21 – 4.05 (m, 1.8H, NC*H*$_2$), 4.02 – 3.85 (m, 1.4H, C*H*CH_3, NC*H*$_2$), 2.71 – 2.56 (m, 1H, (C=O)C*H*$_2$), 2.59 – 2.41 (m, 1H, (C=O)C*H*$_2$), 2.15 – 2.04 (m, 1H, C*H*$_2$CH), 2.01 – 1.83 (m, 1H, C*H*$_2$CH), 1.33 (2×d, *J* = 6.1 Hz, 3H, CHC*H*$_3$) ppm. **^{13}C NMR** (126 MHz, $CDCl_3$) δ 204.4 and 204.0 (*C*=O), 156.64 (2×q, *J* = 37.7 Hz, F_3C(*C*=O)), 116.06 (2×q, *J* = 288 Hz, F_3*C*(C=O)), 77.7 and 77.6 (*spiro*-*C*), 69.8 and 69.5 (*C*HCH_3), 60.8 (q, *J* = 2.2 Hz, N*C*H$_2$) and 58.7 (q, *J* = 2.2 Hz, N*C*H$_2$), 57.9 and 56.5 (N*C*H$_2$), 35.6 and 35.5 ((C=O)*C*H$_2$), 32.6 and 32.2 (CH*C*H$_2$), 21.3 and 21.2 (CH*C*H$_3$) ppm. **^{19}F NMR** (377 MHz, $CDCl_3$) δ –72.5, –72.6 ppm. *Doubling of signals due to presence of rotamers*. (*see spectra*)

HRMS (m/z): (APCI) calc'd for $C_{10}H_{13}NO_3F_3$ $[M+H]^+$: 252.084204, found: 252.084952.

IR (thin film) ν_{max}: 2961, 1715 (C=O), 1700 (C=O), 1473, 1253, 1202, 1145, 1066 cm^{-1}.

2-(2,2,2-Trifluoroacetyl)-5-oxa-2-azaspiro[3.6]decan-10-one: 4s

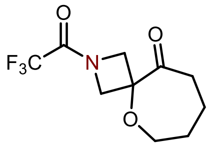

Synthesised according to a modified General Procedure **G** from: **3s** (22.7 mg, 0.100 mmol, 1.00 equiv.), CH_2Cl_2 (5 mL) and TFAA (27.8 µL, 2.00 equiv.). Formation of trace product verified with high resolution mass spectrometry. **HRMS (m/z):** (ESI) calc'd for $C_{10}H_{12}NNaO_3F_3$ $[M+Na]^+$: 274.0661, found: 274.0670. Purification by flash column chromatography failed to isolate the desired product.

1-(2,2,2-Trifluoroacetyl)-3'H-spiro[azetidine-3,2'-benzofuran]-3'-one: 4t

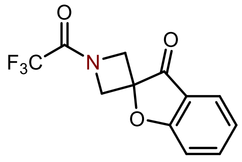

Synthesised according to General Procedure **G** from: **3t** (29.0 mg, 0.100 mmol, 1.00 equiv.), and TFAA (27.8 µL, 2.00 equiv.). Purified by flash column chromatography (SiO_2; 3:1 hexane:EtOAc) to afford **4t** (24.0 mg, 0.089 mmol, 89%) as a white solid.

TLC: R_f = 0.27 (3:1 hexane:EtOAc).

^{1}H NMR (500 MHz, $CDCl_3$) δ 7.75 – 7.67 (m, 2H, ArC*H*), 7.23 – 7.15 (m, 2H, ArC*H*), 4.76 (d, *J* = 10.6 Hz, 1H, NC*H*$_2$), 4.69 (d, *J* = 10.6 Hz, 1H, NC*H*$_2$), 4.53 (dd, *J* = 11.4, 1.7 Hz, 1H, NC*H*$_2$), 4.46 (dd, *J* = 11.4, 1.7 Hz, 1H, NC*H*$_2$) ppm. **^{13}C NMR** (126 MHz, $CDCl_3$) δ 197.6 (*C*=O), 171.5 (Ar*C*), 156.3 (q, *J* = 38.2 Hz, F_3C(*C*=O)), 139.3 (Ar*C*H), 125.0 (Ar*C*H), 123.5 (Ar*C*H), 119.4 (Ar*C*), 116.0 (q, *J* = 288 Hz, F_3*C*(C=O)), 113.6 (Ar*C*H), 80.8 (q, *J* = 1.6 Hz, *spiro*-*C*), 60.5 (q, *J* = 2.5 Hz, N*C*H$_2$) 58.0 (N*C*H$_2$) ppm. **^{19}F NMR** (377 MHz, $CDCl_3$) δ –72.5 ppm. (*see spectra*)

HRMS (m/z): (APCI) calc'd for $C_{12}H_{18}NO_3F_3$ $[M+H]^+$: 272.0259, found: 272.0251.

IR (thin film) ν_{max}: 2951, 1693 (C=O), 1613 (C=O), 1463, 1254, 1201, 1143 cm^{-1}.

2,2,2-Trifluoro-1-(3'-hydroxy-3'H-spiro[azetidine-3,2'-furo[3,2-b]pyridin]-1-yl)ethan-1-one: 4u′

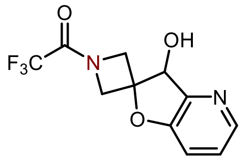

Synthesised according to a modified General Procedure **G** from: **3u′** (36.5 mg, 0.100 mmol, 1.00 equiv.), and TFAA (27.8 µL, 2.00 equiv.). The crude trifluoroacetic acid salt was then washed 4 times with both Et_2O and CH_2Cl_2, carefully removing the solvent with a pasteur pipette. The free pyridine was liberated by dissolving the crude salt in EtOAc solid and stirring with Et_3N (0.139 mL, 10.0 equiv.) for 5 minutes. This mixture was then directly purified by flash column chromatography (SiO_2; EtOAc) to afford **4u′** (18.6 mg, 0.068 mmol, 68%) as a white solid.

TLC: R_f = 0.42 (EtOAc).

^{1}H NMR (500 MHz, $CDCl_3$) δ 8.19 (dd, *J* = 4.3, 1.8 Hz, 1H, ArC*H*), 7.28 – 7.23 (m, 2H, ArC*H*), 6.10 (2×br. s, 1H, O*H*), 5.36 (2×s, 1H, C*H*OH), 5.24 (d, *J* = 11.0 Hz, 0.5H, NC*H*$_2$), 5.01 (d, *J* = 12.4 Hz, 0.5H, NC*H*$_2$), 4.66 (d, *J* = 11.0 Hz, 0.5H, NC*H*$_2$), 4.57 (2×d, *J* = 12.4 Hz, 1H, NC*H*$_2$), 4.46 – 4.30 (m, 1.5H, NC*H*$_2$) ppm. **^{13}C NMR** (126 MHz, $CDCl_3$) δ 156.5 (2×q, *J* = 37.8 Hz, F_3C(*C*=O)), 152.8 and 152.8 (Ar*C*), 149.3 (Ar*C*), 143.3 and 143.3 (Ar*C*H), 125.4 (Ar*C*H), 118.6 (Ar*C*H), 116.1 (q, *J* = 288 Hz, F_3*C*(C=O)), 85.5 (q, *J* = 1.5 Hz, *spiro*-*C*) and 85.4 (q, *J* = 1.5 Hz, *spiro*-*C*), 74.1 and 74.0 (*C*HOH), 64.0 (q, *J* = 2.3 Hz, N*C*H$_2$), 61.2 (N*C*H$_2$), 57.9 (q, *J* = 2.3 Hz, N*C*H$_2$), 55.14 (N*C*H$_2$) ppm. **^{19}F NMR** (377 MHz, $CDCl_3$) δ –72.5, –72.5 ppm. *Doubling of signals due to presence of rotamers*. (*see spectra*)

HRMS (m/z): (APCI) calc'd for $C_{11}H_9N_2O_3F_3$ $[M+H]^+$: 275.0638, found: 275.0636.

IR (thin film) ν_{max}: 3171 (br. OH), 1698 (C=O), 1434, 1261, 1152 cm^{-1}.

1-(2,2,2-Trifluoroacetyl)-3'H-spiro[azetidine-3,2'-naphtho[2,3-b]furan]-3'-one: 4v

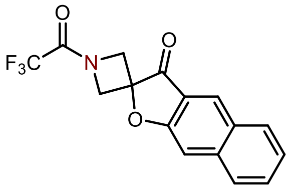

Synthesised according to General Procedure **G** from: **3v** (34.0 mg, 0.100 mmol, 1.00 equiv.), and TFAA (27.8 µL, 2.00 equiv.). Purified by flash column chromatography (SiO_2; 4:1 hexane:EtOAc) to afford **4v** (31.4 mg, 0.098 mmol, 98%) as a bright yellow solid.

TLC: R_f = 0.34 (4:1 hexane:EtOAc).

^{1}H NMR (500 MHz, $CDCl_3$) δ 8.34 (s, 1H, ArC*H*), 7.96 (d, *J* = 8.4 Hz, 1H, ArC*H*), 7.82 (d, *J* = 8.4 Hz, 1H, ArC*H*), 7.62 (app-t, *J* = 7.6 Hz, 1H, ArC*H*), 7.48 – 7.42 (m, 2H, ArC*H*), 4.81 (d, *J* = 10.5 Hz, 1H, NCH_2), 4.73 (d, *J* = 10.5 Hz, 1H, NCH_2), 4.58 (d, *J* = 11.3 Hz, 1H, NCH_2), 4.50 (d, *J* = 11.3 Hz, 1H, NCH_2) ppm. **^{13}C NMR** (126 MHz, $CDCl_3$) δ 198.1 (*C*=O), 164.0 (Ar*C*), 156.3 (q, *J* = 38.2 Hz, F_3C(*C*=O)), 139.8 (Ar*C*), 131.1 (Ar*C*H), 130.6 (Ar*C*H), 129.8 (Ar*C*), 127.7 (Ar*C*H), 127.1 (Ar*C*H), 125.6 (Ar*C*H), 119.7 (Ar*C*), 116.0 (q, *J* = 288 Hz, F_3*C*(C=O)), 107.9 (Ar*C*H), 81.0 (q, *J* = 1.4 Hz, *spiro*-*C*), 60.9 (q, *J* = 2.3 Hz, N*C*H_2), 58.4 (N*C*H_2) ppm. **^{19}F NMR** (377 MHz, $CDCl_3$) δ –72.5 ppm. (*see spectra*)

HRMS (m/z): (APCI) calc'd for $C_{16}H_{10}NO_3F_3$ $[M+H]^+$: 322.0686, found: 322.0684.

IR (thin film) ν_{max}: 3059, 2948, 1700 (C=O), 1634 (C=O), 1452, 1211, 1152 cm^{-1}.

3.7 Synthesis of triflamide spirocycles: 4aa-4cc

2-Methyl-6-((trifluoromethyl)sulfonyl)-1-oxa-6-azaspiro[3.3]heptan-3-one: 4aa

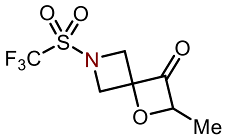

Synthesised according to a modified General Procedure **G** from: **3a** (24.1 mg, 0.100 mmol, 1.00 equiv.), 2,6-lutidine (23.2 µL, 2.00 equiv.) and Tf_2O (33.6 µL, 2.00 equiv.). After 3 h at −78 °C the reaction mixture was warmed to rt and the solvent removed. The residue was purified by flash column chromatography (SiO_2 2.3:1 hexane:EtOAc) to afford **4aa** (8.60 mg, 0.033 mmol, 33%) as a pale-yellow solid.

TLC: R_f = 0.22 (2.3:1 hexane:EtOAc).

^{1}H NMR (400 MHz, CD_2Cl_2) δ 5.55 (q, *J* = 7.0 Hz, 1H, (C=O)C*H*), 4.65 – 4.51 (m, 4H, NC*H*$_2$), 1.48 (d, *J* = 7.0 Hz, 3H, CHC*H*$_3$) ppm. **^{13}C NMR** (126 MHz, CD_2Cl_2) δ 200.1 (*C*=O), 120.1 (q, *J* = 322 Hz, F_3*C*(SO_2)), 99.0 ((C=O)C*H*), 94.0 (*spiro-C*), 62.1 (N*C*H$_2$), 61.9 (N*C*H$_2$), 16.9 (CH*C*H$_3$) ppm. **^{19}F NMR** (377 MHz, $CDCl_3$) δ –75.4 ppm. (*see spectra*)

HRMS (m/z): (APCI) calc'd for $C_7H_8NO_4F_3S$ $[M+H]^+$: 260.0195, found: 260.0199.

IR (thin film) ν_{max}: 2958, 2935, 1828 (C=O), 1389 (S=O), 1227, 1203, 960 cm^{-1}.

6-Methyl-2-(2,2,2-trifluoroacetyl)-5-oxa-2-azaspiro[3.4]octan-8-one: 4bb

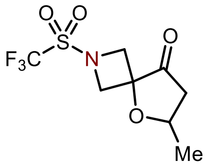

Synthesised according to General Procedure **G** from: **3g** (21.3 mg, 0.100 mmol, 1.00 equiv.), 2,6-lutidine (23.2 µL, 2.00 equiv.) and Tf_2O (33.6 µL, 2.00 equiv.). Purified by flash column chromatography (SiO_2; 9:1 to 3:1 hexane:EtOAc) to afford **4bb** (9.80 mg, 0.036 mmol, 36%) as a white solid.

TLC: R_f = 0.30 (3:1 hexane:EtOAc).

^{1}H NMR (400 MHz, $CDCl_3$) δ 4.42 – 4.26 (m, 4H, NC*H*$_2$, C*H*CH$_3$), 4.22 (d, *J* = 8.6 Hz, 1H, NC*H*$_2$), 2.67 (dd, *J* = 18.4, 6.2 Hz, 1H, (C=O)C*H*$_2$), 2.22 (dd, *J* = 18.4, 8.6 Hz, 1H, (C=O)C*H*$_2$), 1.41 (d, *J* = 6.2 Hz, 3H, CHC*H*$_3$) ppm. **^{13}C NMR** (126 MHz, $CDCl_3$) δ 211.6 (*C*=O), 120.0 (q, *J* = 322 Hz, F_3*C*(SO_2)), 75.7 (*spiro-C*), 72.5 (O*C*HCH$_3$), 62.7 (N*C*H$_2$), 61.8 (N*C*H$_2$), 42.9 ((C=O)*C*H$_2$), 21.5 (CH*C*H$_3$) ppm. **^{19}F NMR** (377 MHz, $CDCl_3$) δ –75.3 ppm. (*see spectra*)

HRMS (m/z): (APCI) calc'd for $C_8H_{10}NO_4F_3S$ $[M+H]^+$: 274.0355, found: 274.0345.

IR (thin film) ν_{max}: 2963, 1765 (C=O), 1389 (S=O), 1258, 1221, 1091, 1010 cm^{-1}.

2-((Trifluoromethyl)sulfonyl)-5-oxa-2-azadispiro[3.1.56.24]tridecan-13-one: 4cc

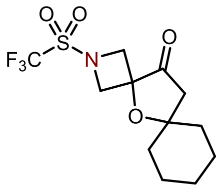

Synthesised according to General Procedure **G** from: **3p** (26.7 mg, 0.100 mmol, 1.00 equiv.), 2,6-lutidine (23.2 µL, 2.00 equiv.) and Tf_2O (33.6 µL, 2.00 equiv.). Purified by flash column chromatography (SiO_2; 5.7:1 to 3:1 hexane:EtOAc) to afford **4cc** (18.0 mg, 0.055 mmol, 55%) as a white solid.

TLC: R_f = 0.31 (4:1 hexane:EtOAc).

^{1}**H NMR** (500 MHz, $CDCl_3$) δ 4.35 (d, J = 8.4 Hz, 2H, NCH_2), 4.26 (d, J = 8.4 Hz, 2H, NCH_2), 2.43 (s, 2H, (C=O)CH_2), 1.83 – 1.72 (m, 2H, cy-CH_2), 1.67 – 1.58 (m, 2H, cy-CH_2), 1.57 – 1.47 (m, 3H, cy-CH_2), 1.48 – 1.37 (m, 3H, cy-CH_2) ppm. ^{13}**C NMR** (126 MHz, $CDCl_3$) δ 212.5 (*C*=O), 119.9 (q, J = 322 Hz, F_3*C*(SO_2)), 82.0 (cy *spiro*-*C*), 74.8 (azetidine *spiro*-*C*), 63.1 (N*C*H_2), 47.3 ((C=O)*C*H_2), 37.5 (cy-*C*H_2), 25.0 (cy-*C*H_2), 22.9 (cy-*C*H_2) ppm. ^{19}**F NMR** (377 MHz, $CDCl_3$) δ –75.4 ppm. (*see spectra*)

HRMS (m/z): (ESI) calc'd for $C_{12}H_{16}F_3NNaO_4S$ $[M+Na]^+$: 350.0644, found: 350.0654.

IR (thin film) ν_{max}: 2937, 1762 (C=O), 1390 (S=O), 1223, 1192, 1093 cm^{-1}.

3.8 Functionalization of oxa-azaspirocycles

***tert*-butyl 2-methyl-3-oxo-1-oxa-6-azaspiro[3.3]heptane-6-carboxylate: 8a**

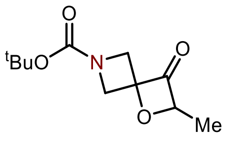

Synthesised according to GP **H** from: **4a** (44.6 mg, 0.200 mmol, 1.00 equiv.), K_2CO_3 (221 mg, 8.00 equiv.) and di-*tert*-butyl dicarbonate (91.9 µL 2.00 equiv.). Purified by flash column chromatography on silica gel to yield the desired product (30.5 mg, 0.134 mmol, 67%) as a white solid.

TLC: R_f = 0.30 (3:1 hexane:EtOAc).

^{1}**H NMR** (400 MHz, $CDCl_3$) δ 5.44 (q, J = 7.0 Hz, 1H, (C=O)C*H*), 4.34 – 4.29 (m, 2H, NCH_2), 4.22 – 4.19 (m, 2H, NCH_2), 1.46 (d, J = 7.0 Hz, 3H, CHCH_3), 1.43 (s, 9H, OC(CH_3)$_3$) ppm. ^{13}**C NMR** (101 MHz, $CDCl_3$) δ 202.5 (*C*=O), 156.0 ((*C*=O)OC(CH_3)$_3$), 97.1 ((C=O)C*H*), 96.2 (*spiro*-*C*), 80.5 (O*C*(CH_3)$_3$), 59.1 (br, N*C*H_2), 28.4 (OC(*C*H_3)$_3$), 16.9 (CH*C*H_3) ppm. (*see spectra*)

HRMS (m/z): (ESI) calc'd for $C_{11}H_{17}NNaO_4$ $[M+Na]^+$: 250.1050, found: 250.1044.

IR (thin film) ν_{max}: 2976, 2931, 1822 (C=O), 1699 (C=O), 1392, 1367, 1245, 1167, 1102 cm^{-1}.

tert-butyl 13-oxo-5-oxa-2-azadispiro[3.1.5^6.2^4]tridecane-2-carboxylate: 8p

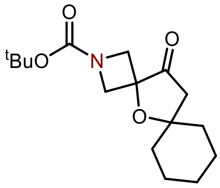

Synthesised according to GP **H** from: **4p** (58.3 mg, 0.200 mmol, 1.00 equiv.), K_2CO_3 (221 mg, 8.00 equiv.) and di-*tert*-butyl dicarbonate (91.9 µL 2.00 equiv.). Purified by flash column chromatography on silica gel to yield the desired product (48.9 mg, 0.166 mmol, 83%) as a white solid.

TLC: R_f = 0.22 (4:1 hexane:EtOAc).

^{1}H NMR (400 MHz, $CDCl_3$) δ 4.09 (dd, *J* = 8.9, 1.1 Hz, 2H, NC*H*$_2$), 3.95 (dd, *J* = 8.9, 1.1 Hz, 2H, NC*H*$_2$), 2.38 (s, 2H, (C=O)C*H*$_2$), 1.84 – 1.70 (m, 2H, cy-C*H*$_2$), 1.68 – 1.56 (m, 2H, cy-C*H*$_2$), 1.56 – 1.47 (m, 3H, cy-C*H*$_2$), 1.43– 1.39 (m, 12H, cy-C*H*$_2$, OC(C*H*$_3$)$_3$) ppm. **^{13}C NMR** (126 MHz, $CDCl_3$) δ 214.9 (*C*=O), 155.9 ((*C*=O)OC(CH$_3$)$_3$), 81.0 (cy *spiro*-*C*), 80.1 (O*C*(CH$_3$)$_3$), 76.1 (azetidine *spiro*-*C*), 61.0 (br, N*C*H$_2$), 60.2 (br, N*C*H$_2$), 47.6 ((C=O)*C*H$_2$), 37.5 (cy-*C*H$_2$), 28.5 (OC(*C*H$_3$)$_3$), 25.1, (cy-*C*H$_2$), 22.9 (cy-*C*H$_2$) ppm. (*see spectra*)

HRMS (m/z): (ESI) calc'd for $C_{16}H_{25}NNaO_4$ [M+Na]$^+$: 318.167579, found: 318.169008.

IR (thin film) ν$_{max}$: 2932, 2855, 1756 (C=O), 1697 (C=O), 1389, 1364, 1259, 1160, 1055 cm^{-1}.

tert-butyl 3'-oxo-3'H-spiro[azetidine-3,2'-naphtho[2,3-b]furan]-1-carboxylate: 8v

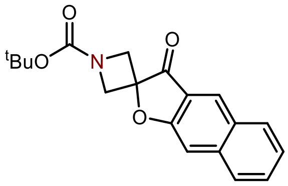

Synthesised according to GP **H** from: **4v** (64.3 mg, 0.200 mmol, 1.00 equiv.), K_2CO_3 (221 mg, 8.00 equiv.) and di-*tert*-butyl dicarbonate (91.9 µL 2.00 equiv.). Purified by flash column chromatography on silica gel to yield the desired product (52.6 mg, 0.162 mmol, 81%) as a white solid.

TLC: R_f = 0.35 (4:1 hexane:EtOAc).

^{1}H NMR (400 MHz, $CDCl_3$) δ 8.30 (s, 1H, ArC*H*), 7.93 (d, *J* = 8.4 Hz, 1H, ArC*H*), 7.80 (d, *J* = 8.4 Hz, 1H, ArC*H*), 7.58 (ddd, *J* = 8.4, 6.7, 1.3 Hz, 1H, ArC*H*), 7.47 – 7.38 (m, 2H, ArC*H*), 4.35 (dd, *J* = 9.5, 1.2 Hz, 2H, NC*H*$_2$), 4.27 (dd, *J* = 9.5, 1.2 Hz, 2H, NC*H*$_2$), 1.49 (s, 9H, OC(C*H*$_3$)$_3$) ppm. **^{13}C NMR** (126 MHz, $CDCl_3$) δ 199.6 (*C*=O), 164.3 (Ar*C*), 156.0 ((*C*=O)OC(CH$_3$)$_3$), 139.6 (Ar*C*), 131.1 (Ar*C*H), 130.1 (Ar*C*H), 129.5 (Ar*C*), 127.6 (Ar*C*H),

126.6 (Ar*C*H), 125.2 (Ar*C*H), 120.4 (Ar*C*), 107.7 (Ar*C*H), 81.6 (*spiro-C*), 80.6 (O*C*(CH_3)$_3$), 58.8 (br, N*C*H_2), 58.2 (br, N*C*H_2), 28.5 (OC(*C*H_3)$_3$) ppm. (*see spectra*)

HRMS (m/z): (ESI) calc'd for $C_{19}H_{19}NNaO_4$ [M+Na]$^+$: 348.1206, found: 348.1219.

IR (thin film) ν$_{max}$: 2979, 1702 (C=O), 1634 (C=O), 1393, 1160, 1090 cm^{-1}.

4 Further studies of the spirocyclization-desilylation reaction

4.1 Unsuccessful substrates

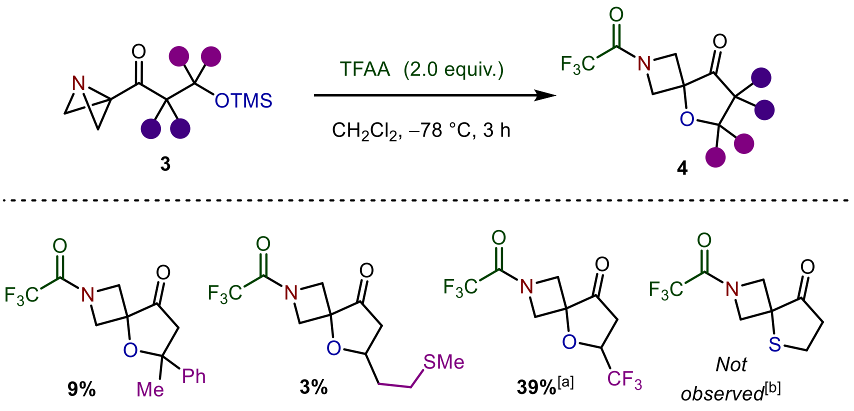

All reactions were carried out using **3** (0.10 mmol) in CH_2Cl_2 (1.0 mL) according to General Procedure **G**. Yields were determined by ^{1}H NMR analysis using dibromomethane as an internal standard. [a] Isolation of product was unsuccessful. [b] From methyl sulfide.

Scheme S2. Unsuccessful substrates in the spirocyclization-desilylation reaction.

Attempted synthesis of 1-(2,2,2-trifluoroacetyl)-3'H-spiro[azetidine-3,2'-furo[3,2-b]pyridin]-3'-one: 4u

Desired product **4u** was not observed in the spirocyclization reaction of **3u** which only yielded methyl ester **4ua** (Scheme S3; *see spectra*). Presumably this is formed *via* pyridine protonation and methanolysis of the ketone upon quenching the reaction with MeOH. This could then be followed by hemi-acetal collapse and deacylation.[15] Attempts to supress this undesired reactivity failed to provide spirocyclic compoud **4u**.

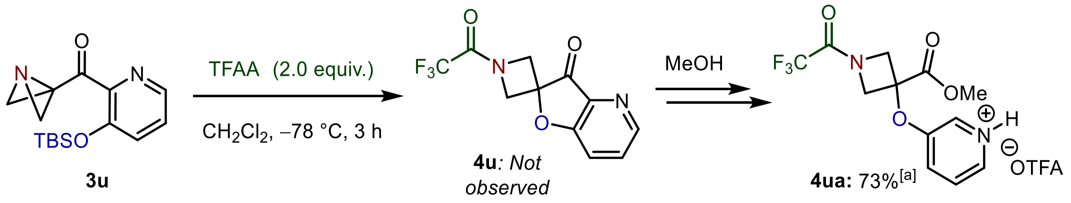

Scheme S3. Attempted spirocyclization-desilylation reaction of substrate **3u**. Reaction was carried out using **3u** (0.10 mmol) in CH_2Cl_2 (1.0 mL) according to General Procedure **G**. [a] Isolated yield.

4.2 Comparison of secondary alcohol TES and TMS protecting groups

Typically, secondary alcohol substrates showed little difference in yield between TMS and TES protecting groups (Table S6; entries 1-3). However, in the case where the build-up of positive charge on the ABB-ketone β-carbon can be effectively stabilised then little to no spirocyclic product is observed when the larger silyl group (TES) is used (entries 4 and 5). This is believed to be a consequence of the slower rate of oxonium desilylation which results in competing C-O bond cleavage.

Table S6. Comparison of the TES and TMS protecting groups for secondary alcohol substrates in the spirocyclization-desilylation reaction.

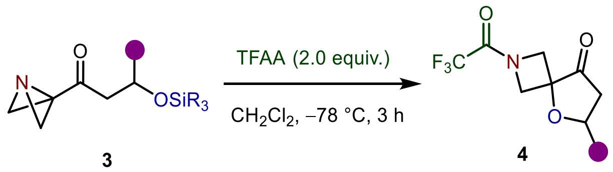

Entry	●	TES: % Yield	TMS: % Yield
1	Me	84	82
2	CH_2OBn	36	41
3	CH_2Cl	38	45
4	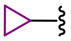	5[a]	35
5	Ph	Trace	42

All reactions were carried out using **3** (0.10 mmol) in CH_2Cl_2 (1.0 mL) according to General Procedure **G**. Isolated yields given. [a] NMR yield as determined by ^{1}H NMR analysis using dibromomethane as an internal standard.

4.3 Effect of trifluoroacetate concentration

Increasing the relative concentration of trifluoroacetate anion was achieved with the addition of NBu_4OTFA. Increasing the equivalents of this additive decreased the yield of product whilst promoting the formation of **6h** (Table S7). This suggested that **6h** is an undesired intermediate and is unlikely to be transformed to **4h** under the reaction conditions. During purification, **6h** decomposed rapidly to give a complex mixture of uncharacterizable species.

Table S7. Effect of trifluoroacetate concentration in the spirocyclization-desilylation reaction.

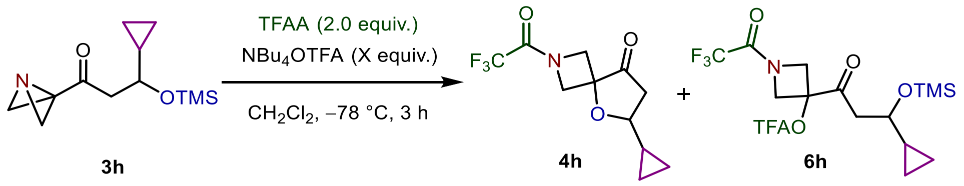

Entry	X	4h: % Yield	6h: % Yield
1	0.5	16	26
2	1.0	9	39
3[a]	1.0	21	23
4	2.0	-	44

All reactions were carried out using **3h** (0.10 mmol) in CH_2Cl_2 (1.0 mL) according to a modified General Procedure **G**. Yields were determined by ^{1}H NMR analysis using dibromomethane as an internal standard. [a] NBu_4OTFA added after 15 minutes.

4.4 Investigation of suboptimal substrates: Representative example 3j′

Trifluoroacetate **7** was observed for substrates that could stabilise positive adjacent to the silyl ether of the ABB-ketone. In the case of ABB-ketone **3j′** product formation was almost entirely supressed by this deleterious side reaction (Scheme S4).

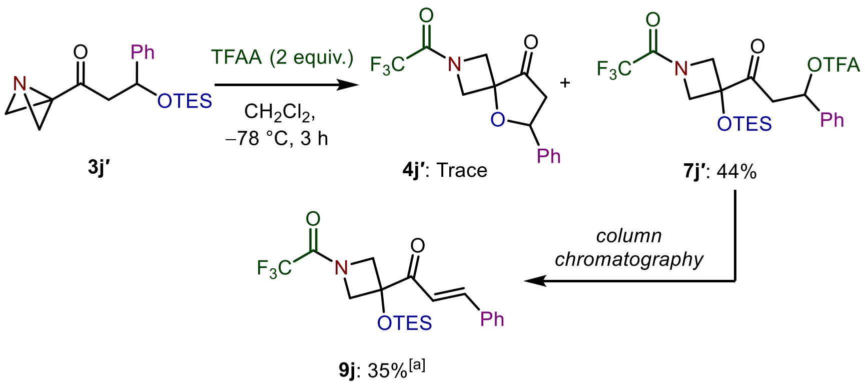

Scheme S4. Observations of **7j′** in the spirocyclization-desilylation reaction of substrate **3j′**. Yields were determined by ^{1}H NMR analysis using dibromomethane as an internal standard. [a] Isolated yield.

7j′ can be easily identified by crude ^{1}H NMR analysis by the indicative downfield chemical shift of the H atom adjacent to the trifluoroacetate group (6.40 ppm: Figure S2). However, during attempted isolation **7j′** readily eliminates to enone **9j**. Despite this, minor amounts of **7j′** could be observed after chromatography and was isolated as an inseparable mixture with

9j. Although **9j** was observed (>5%) in the crude reaction mixture (7.16 and 7.85 ppm) the yield obtained of **9j** after purification (35%) indicates this species forms directly from **7j′** during chromatography.

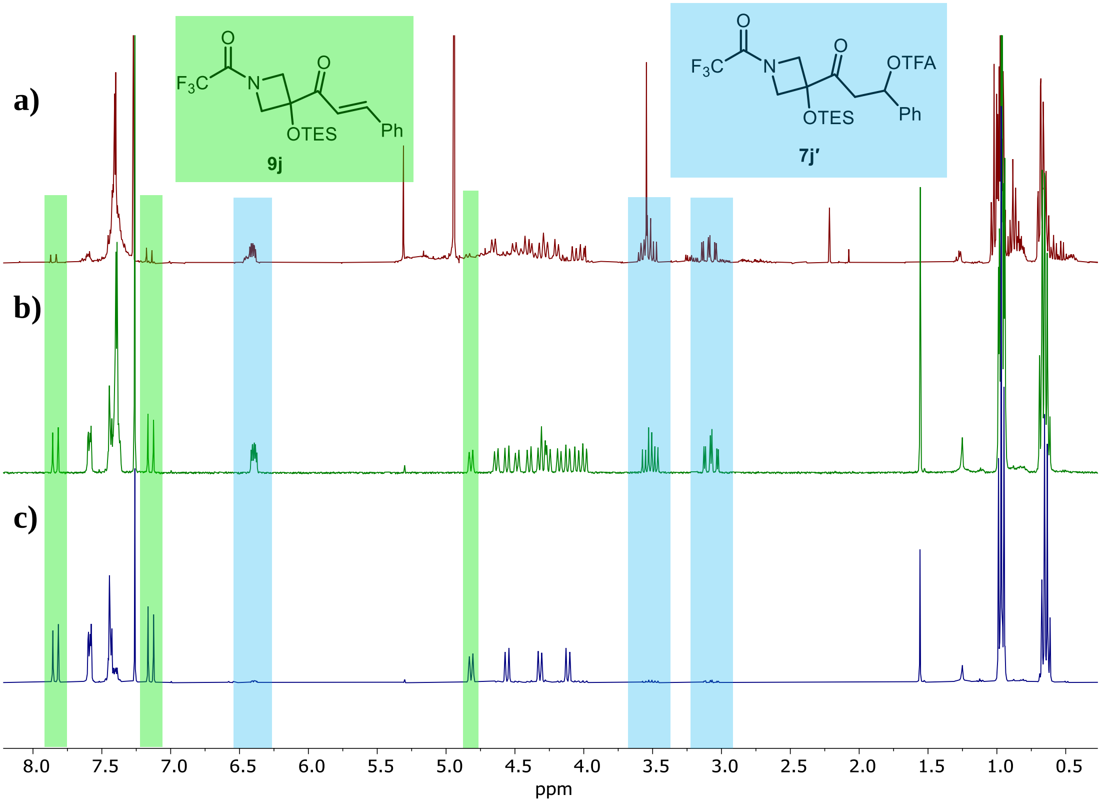

Figure S2. a) Crude ^{1}H NMR spectra of the spirocyclization-desilylation reaction from **3j′**. b) Isolated sample of the mixture of **7j′** and **9j**. c) Isolated sample of **9j**. Identifiable signals from **7j′** highlighted in blue. Identifiable signals from **9j** highlighted in green.

(E)-3-phenyl-1-(3-((triethylsilyl)oxy)-1-(2,2,2-trifluoroacetyl)azetidin-3-yl)prop-2-en-1-one: 9j

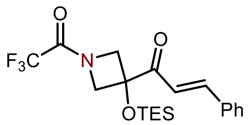

TLC: R_f = 0.37 (9:1 hexane:EtOAc).

^{1}H NMR (400 MHz, $CDCl_3$) δ 7.84 (d, *J* = 16.0 Hz, 1H, PhC*H*CH), 7.62 – 7.56 (m, 2H, ArC*H*), 7.47 – 7.37 (m, 3H, ArC*H*), 7.14 (d, *J* = 16.0 Hz, 1H, PhCHC*H*), 4.82 (d, *J* = 10.1 Hz, 1H, NCH_2), 4.56 (d, *J* = 11.0 Hz, 1H, NCH_2), 4.32 (d, *J* = 10.1 Hz, 1H, NCH_2), 4.12 (d, *J* = 11.5 Hz, 1H, NCH_2), 0.97 (t, *J* = 7.9 Hz, 9H, Si(CH_2CH_3)$_3$), 0.64 (q, *J* = 7.9 Hz, 6H, Si(CH_2CH_3)$_3$) ppm. **^{13}C NMR** (126 MHz, $CDCl_3$) δ 195.9 (*C*=O), 156.6 (q, *J* = 37.7 Hz, F_3C(*C*=O)), 146.8 ((Ph)H*C*=CH), 134.3 (Ar*C*), 131.4 (Ar*C*H), 129.3 (Ar*C*H), 128.8 (Ar*C*H), 118.8 ((Ph)HC=*C*H), 116.1 (q, *J* = 288 Hz, F_3*C*(C=O)), 75.9 (*C*OSi), 62.0 (N*C*H_2), 59.5 (N*C*H_2), 6.8

(Si(CH$_2$*C*H$_3$)$_3$), 5.9 (Si(*C*H$_2$CH$_3$)$_3$) ppm. **^{19}F NMR** (377 MHz, CDCl$_3$) δ –72.5 ppm. (*see spectra*)

HRMS (m/z): (APCI) calc'd for $C_{20}H_{26}NO_3F_3Si$ [M+H]$^+$: 414.1707, found: 414.1697.

IR (thin film) ν$_{max}$: 2960, 2881, 1699 (C=O), 1608 (C=O), 1576 (C=C), 1457, 1252, 1149 cm^{-1}.

5 X-ray crystal structure of 4a

X-ray diffraction experiments on **4a** were carried out at 100(2) K on a Bruker APEX II CCD diffractometer using Mo-K_α radiation (λ = 0.71073 Å). Intensities were integrated in SAINT[16] and absorption corrections based on equivalent reflections were applied using SADABS.[17] The structure was solved using ShelXT[18] and refined by full matrix least squares against F^2 in ShelXL[18, 19] using Olex2.[20] All of the non-hydrogen atoms were refined anisotropically. While all of the hydrogen atoms were located geometrically and refined using a riding model. Crystal structure and refinement data are given in Table S8.

Table S8. Crystal data and structure refinement for **4a**

CCDC	2057843
Empirical formula	$C_8H_8F_3NO_3$
Formula weight	223.15
Temperature/K	100(2)
Crystal system	orthorhombic
Space group	$P2_12_12_1$
a/Å	9.3776(3)
b/Å	9.3907(3)
c/Å	10.4759(3)
α/°	90
β/°	90
γ/°	90
Volume/Å^3	922.53(5)
Z	4
ρ_{calc}g/cm^3	1.607
μ/mm^{-1}	0.160
F(000)	456.0
Crystal size/mm^3	0.498 × 0.44 × 0.394
Radiation	MoKα (λ = 0.71073)
2θ range for data collection/°	5.826 to 55.926
Index ranges	-12 ≤ h ≤ 9, -12 ≤ k ≤ 12, -13 ≤ l ≤ 13
Reflections collected	17072
Independent reflections	2226 [R_{int} = 0.0376, R_{sigma} = 0.0228]
Data/restraints/parameters	2226/0/137
Goodness-of-fit on F^2	1.073
Final R indexes [I>=2σ (I)]	R_1 = 0.0282, wR_2 = 0.0700
Final R indexes [all data]	R_1 = 0.0301, wR_2 = 0.0712
Largest diff. peak/hole / e Å^{-3}	0.27/-0.18

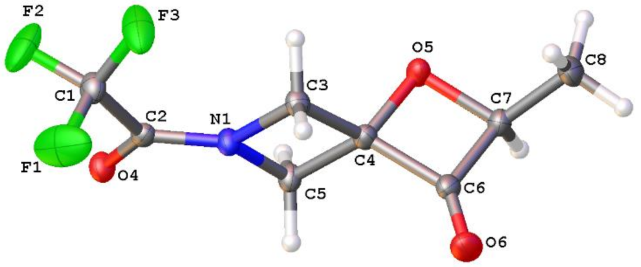

.

Figure S3. Crystal structure of **4a** with the anisotropic displacement parameters depicted at the 50% probability level (CCDC 2057843).

6 References

[1] G. R. Stanton, G. Koz, P. J. Walsh, *J. Am. Chem. Soc.* **2011**, *133*, 7969.

[2] K. Anderson, S. Atkinson, T. Fujiwara, M. Giles, T. Matsumoto, E. Merifield, J. Singleton, T. Saito, T. Sotoguchi, J. Tornos, E. Way, *Org. Process Res. Dev.* **2010**, *14*, 58.

[3] C.-M. Martinez, A. Neudörffer, M. Largeron, *Org. Biomol. Chem.* **2012**, *10*, 3739.

[4] A. K. Ghosh, H. Yuan, *Tetrahedron Lett.* **2009**, *50*, 1416.

[5] A. Fawcett, A. Murtaza, C. H. U. Gregson, V. K. Aggarwal, *J. Am. Chem. Soc.* **2019**, *141*, 4573.

[6] S. R. Angle, I. Choi, F. S. Tham, *J. Org. Chem.* **2008,** *73*, 6268.

[7] J. Marshall, B. Johns, *J. Org. Chem.* **1998**, *63*, 7885.

[8] I. Paterson. K. Ashton, R. Britton, G. Cecere, G. Chouraqui, G. Florence, H. Knust, J. Stafford, *Chem. Asian J.* **2008**, *3*, 367.

[9] D.-Y. Zhu, Z. Zhang, X.-Q. Mou, Y.-Q. Tu, F.-M. Zhang, J.-B. Peng, S.-H. Wang, S.-Y. Zhang, *Adv. Synth. Catal*. **2015**, *357*, 747.

[10] T. Durham, M. Miller, *J. Org. Chem.* **2003**, 68, 27.

[11] C. Palomo, J. Aizpurua, N. Aurrejoetxea, M. C. López, *Tetrahedron Lett*. **1991**, *32*, 2525.

[12] J.-A. Funel, J. Prunet, *J. Org. Chem*. **2004**, *69*, 4555.

[13] V. K. Chatare, R. B. Andrade, *Angew. Chem. Int. Ed*. **2017**, *56*, 5909; *Angew. Chem.* **2017**, *129*, 6003.

[14] R. Carrillo, M. Hynes, V. Martín, T. Martín, F. P. Crisóstomo, *Org. Lett.* **2015**, *17*, 2912.

[15] R. Williams, S. Neergaard, *J. Heterocycl. Chem.* **1982**, *19*, 1549.

[16] Bruker, SAINT+ v8.38A Integration Engine, Data Reduction Software, Bruker Analytical X-ray Instruments Inc. Madison, WI, USA, **2015**.

[17] Bruker, SADABS, Bruker AXS area detector scaling and absorption correction, Bruker Analytical X-ray Instruments Inc. Madison, Wisconsin, USA, **2001**.

[18] G. M. Sheldrick, *Acta Crystallogr. C* **2015**, *71*, 3.

[19] G. M. Sheldrick, *Acta Crystallogr. Sect. A: Found. Crystallogr.* **2008**, *64*, 112.

[20] O. V. Dolomanov, L. J. Bourhis, R. J. Gildea, J. A. K. Howard, H. Puschmann, *J. Appl. Crystallogr*. **2009**, *42*, 339.

Strain-Release Driven Spirocyclization of Azabicyclo[1.1.0]butyl Ketones: Full author list

[4] b) F. Velázquez, M. Chelliah, M. Clasby, Z. Guo, J. Howe, R. Miller, S. Neelamkavil, U. Shah, A. Soriano, Y. Xia, S. Venkatraman, S. Chackalamannil, I. W. Davies, *ACS Med. Chem. Lett*. **2016**, *12*, 1173. c) H. Huang, D. S. La, A. C. Cheng, D. A. Whittington, V. F. Patel, K. Chen, T. A. Dineen, O. Epstein, R. Graceffa, D. Hickman, Y. H. Kiang, S. Louie, Y. Luo, R. C. Wahl, P. H. Wen, S. Wood, R. T. Fremeau, *J. Med. Chem*. **2012**, *21*, 9156.

[5] b) A. Johansson, C. Löfberg, M. Antonsson, S. von Unge, M. A. Hayes, R. Judkins, K. Ploj, L. Benthem, D. Lindén, P. Brodin, M. Wennerberg, M. Fredenwall, L. Li, J. Persson, R. Bergman, A. Pettersen, P. Gennemark, A. Hogner, *J. Med. Chem.* **2016**, *59*, 2497. c) D. W. Kung, S. B. Coffey, R. M. Jones, S. Cabral, W. Jiao, M. Fichtner, P. A. Carpino, C. R. Rose, R. F. Hank, M. G. Lopaze, R. Swartz, H. Chen, Z. Hendsch, B. Posner, C. F. Wielis. B. Manning, J. Dubins, I. A. Stock, S. Varma, M. Campbell, D. DeBartola, R. Kosa-Maines, S. J. Steyn, K. F. McClure, *Bioorg. Med. Chem. Lett.* **2012**, *22*, 4281.

[18] d) G. Wuitschik, M. Rogers-Evans, A. Buckl, M. Bernasconi, M. Märki, T. Godel, H. Fischer, B. Wagner, I. Parrilla, F. Schuler, J. Schneider, A. Alker, W. B. Schweizer, K. Müller, E. M. Carreira, *Angew. Chem. Int. Ed*. **2008**, *47*, 4512; *Angew. Chem.* **2008**, *120*, 4588.

7 NMR spectra

^{1}H NMR (400 MHz, $CDCl_3$) of **Ethyl 2-((triethylsilyl)oxy)propanoate: 2a** (*see procedure*)

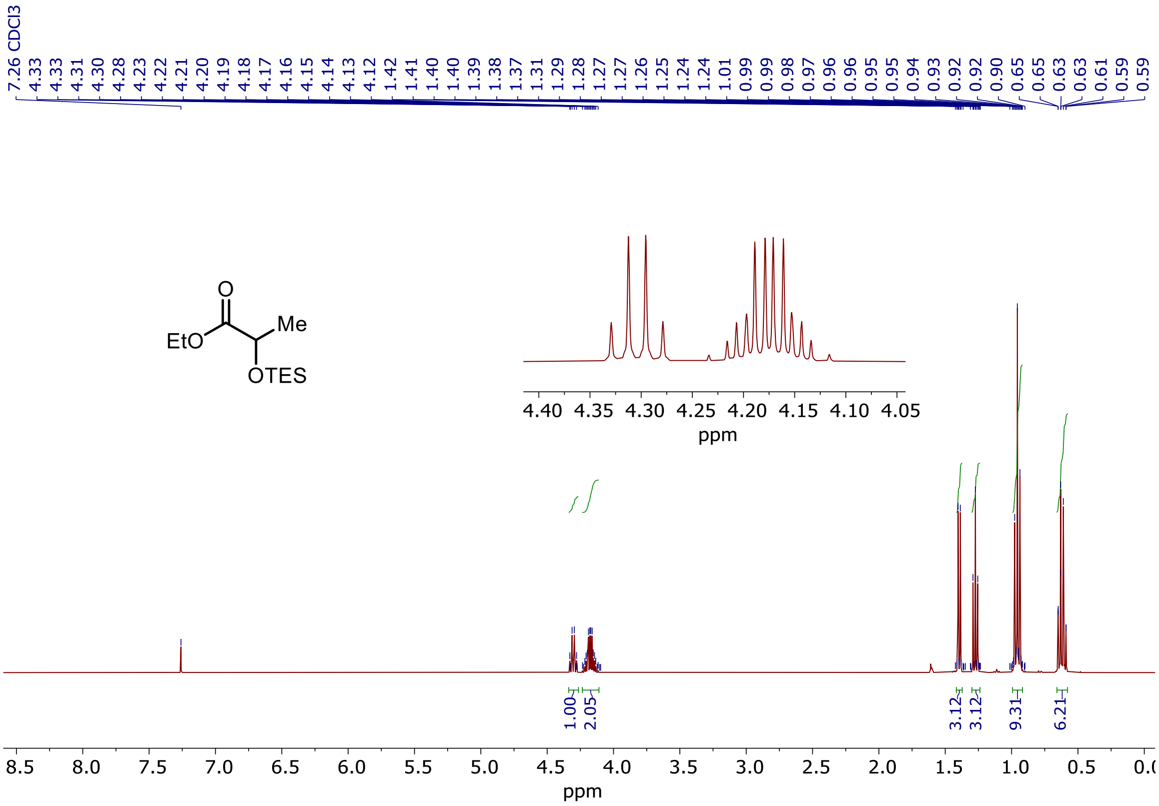

^{13}C NMR (101 MHz, $CDCl_3$) of **Ethyl 2-((triethylsilyl)oxy)propanoate: 2a**

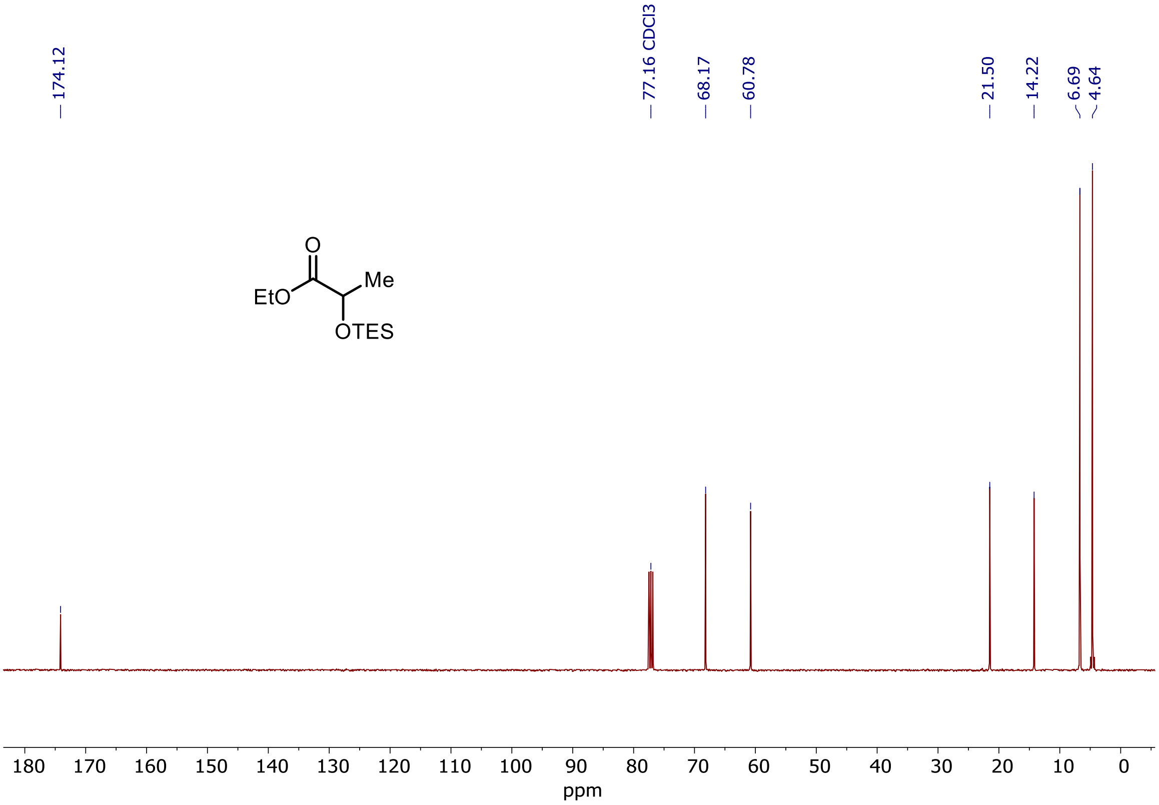

¹H NMR (400 MHz, $CDCl_3$) of **Ethyl 3-phenyl-2-((triethylsilyl)oxy)propanoate: 2b** (*see procedure*)

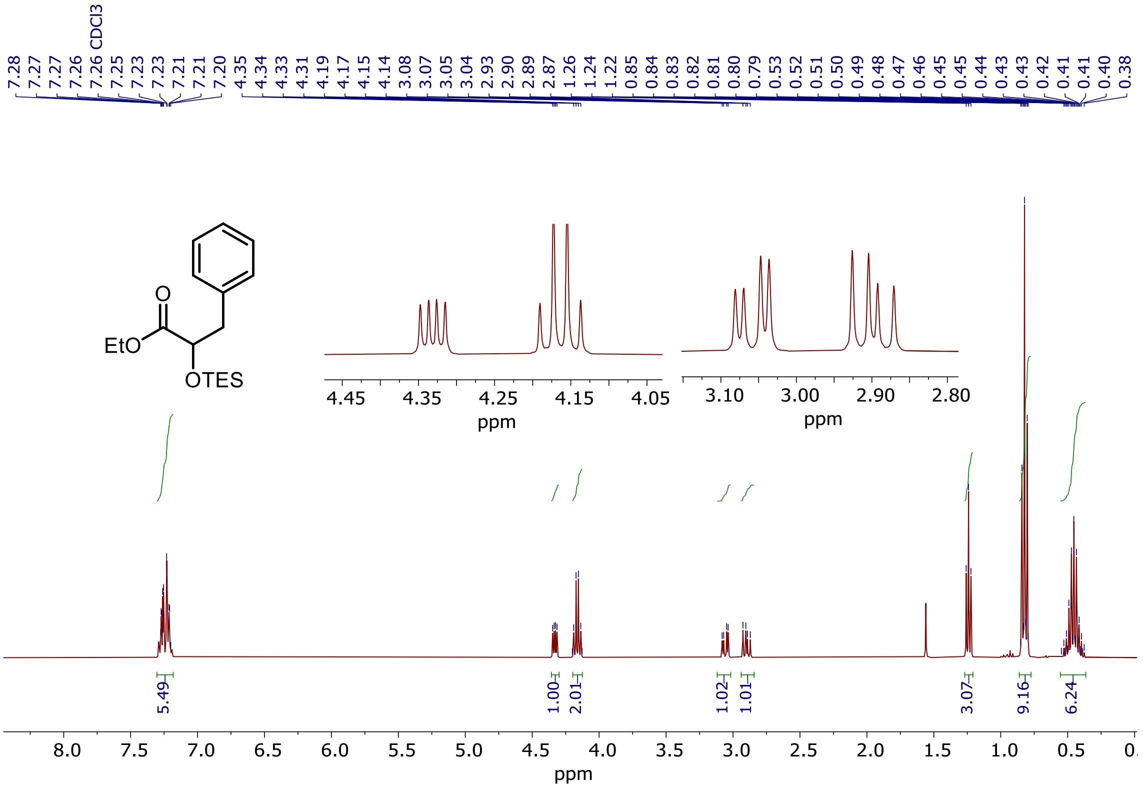

¹³C NMR (101 MHz, $CDCl_3$) of **Ethyl 3-phenyl-2-((triethylsilyl)oxy)propanoate: 2b**

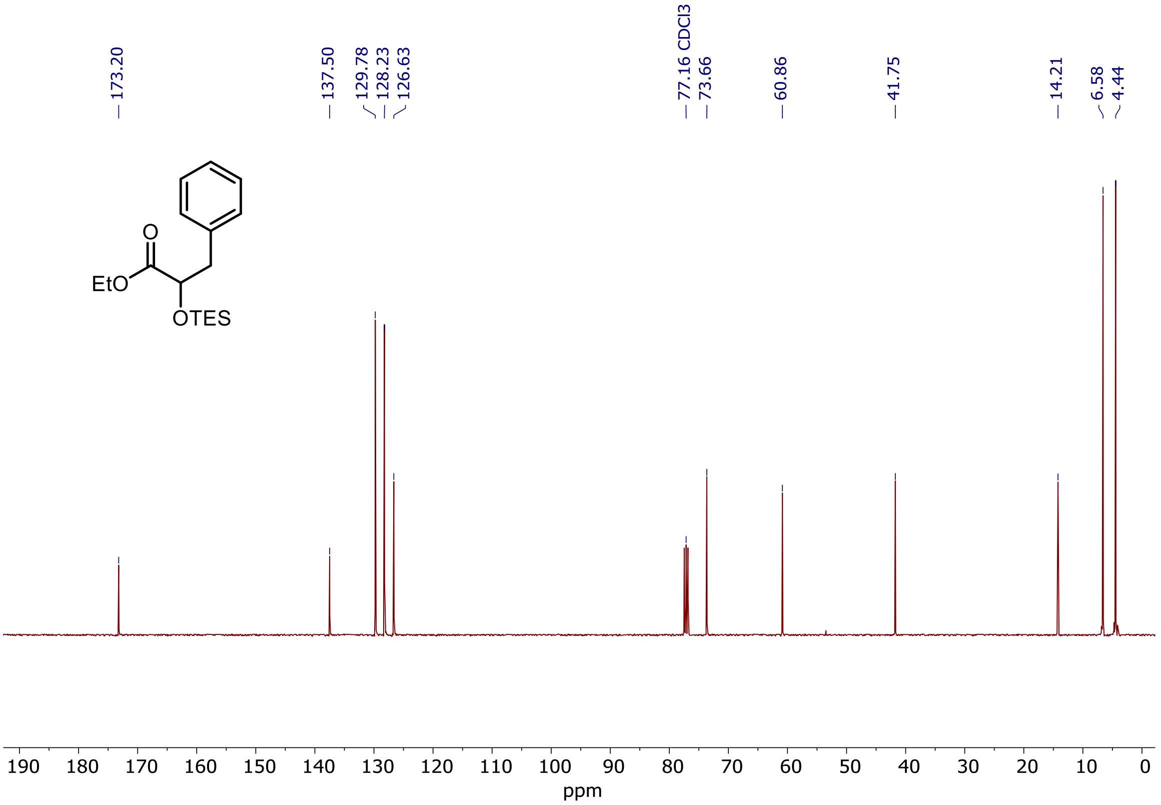

^{1}H NMR (400 MHz, $CDCl_3$) of **Ethyl 2-methyl-2-((triethylsilyl)oxy)propanoate: 2c** (*see procedure*)

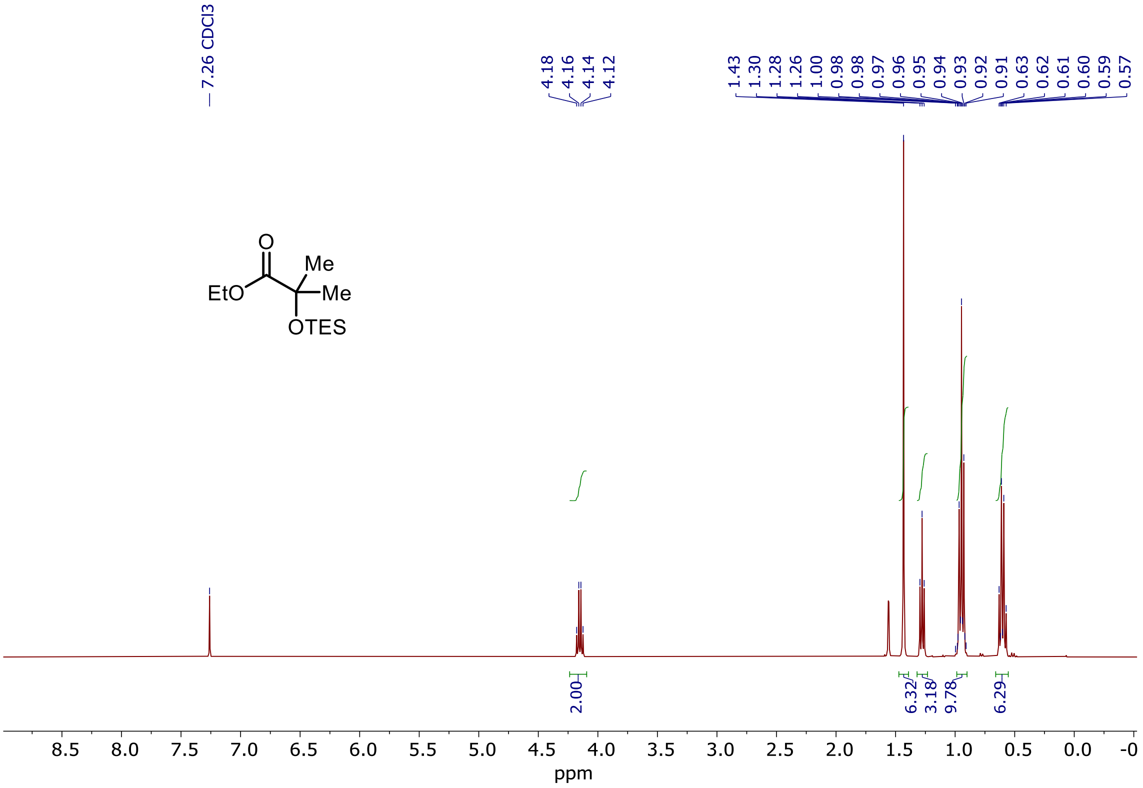

^{13}C NMR (126 MHz, $CDCl_3$) of **Ethyl 2-methyl-2-((triethylsilyl)oxy)propanoate: 2c**

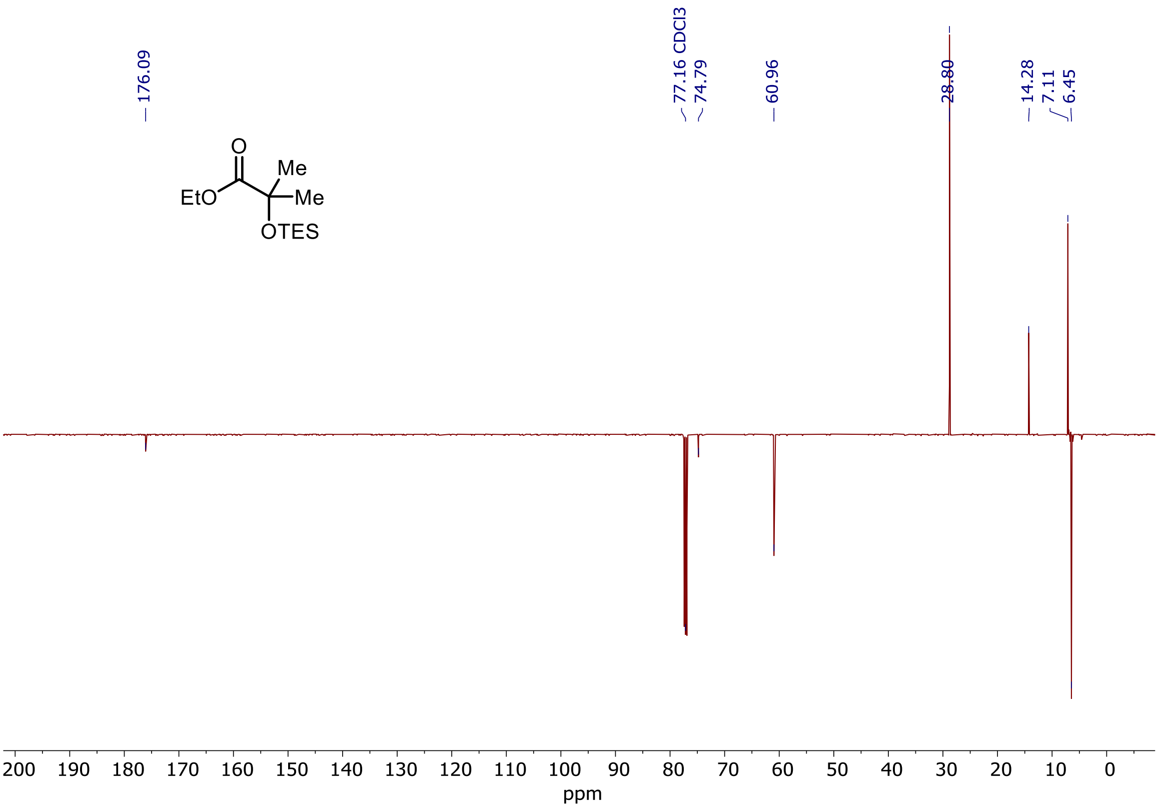

¹H NMR (500 MHz, $CDCl_3$) of **Methyl 2-methyl-3-((triethylsilyl)oxy)propanoate** (see procedure)

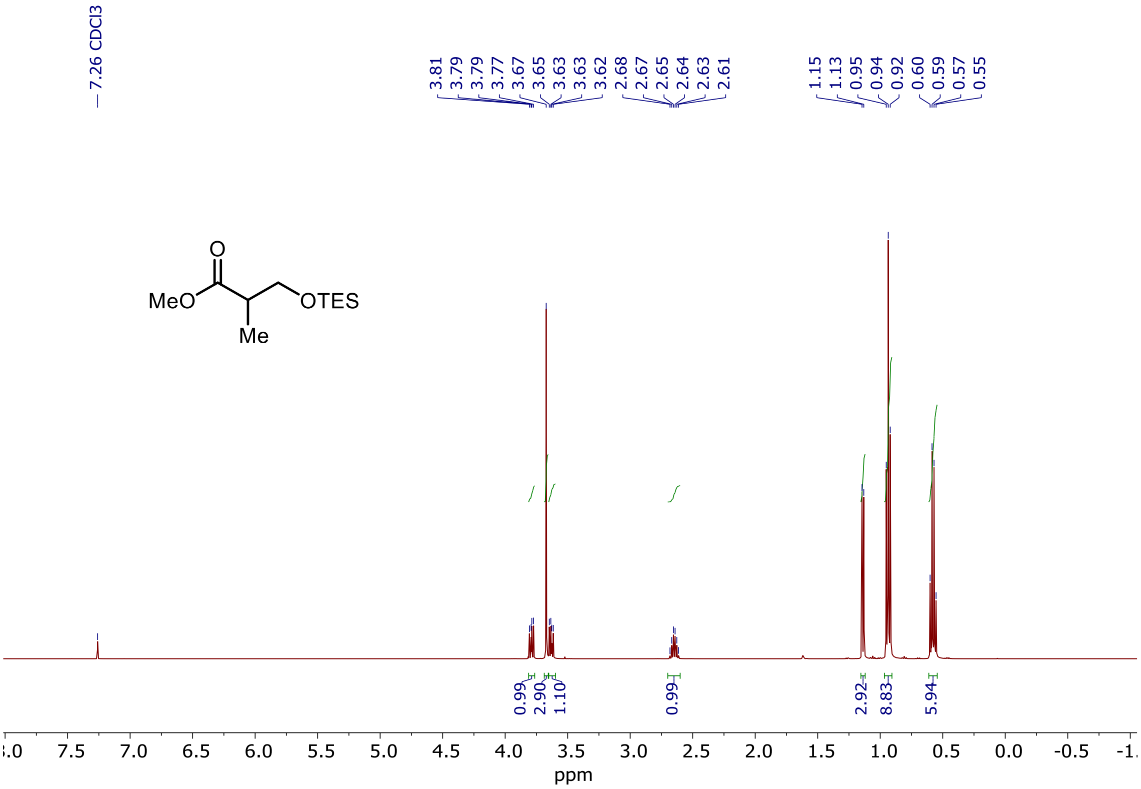

¹³C NMR (126 MHz, $CDCl_3$) of **Methyl 2-methyl-3-((triethylsilyl)oxy)propanoate**

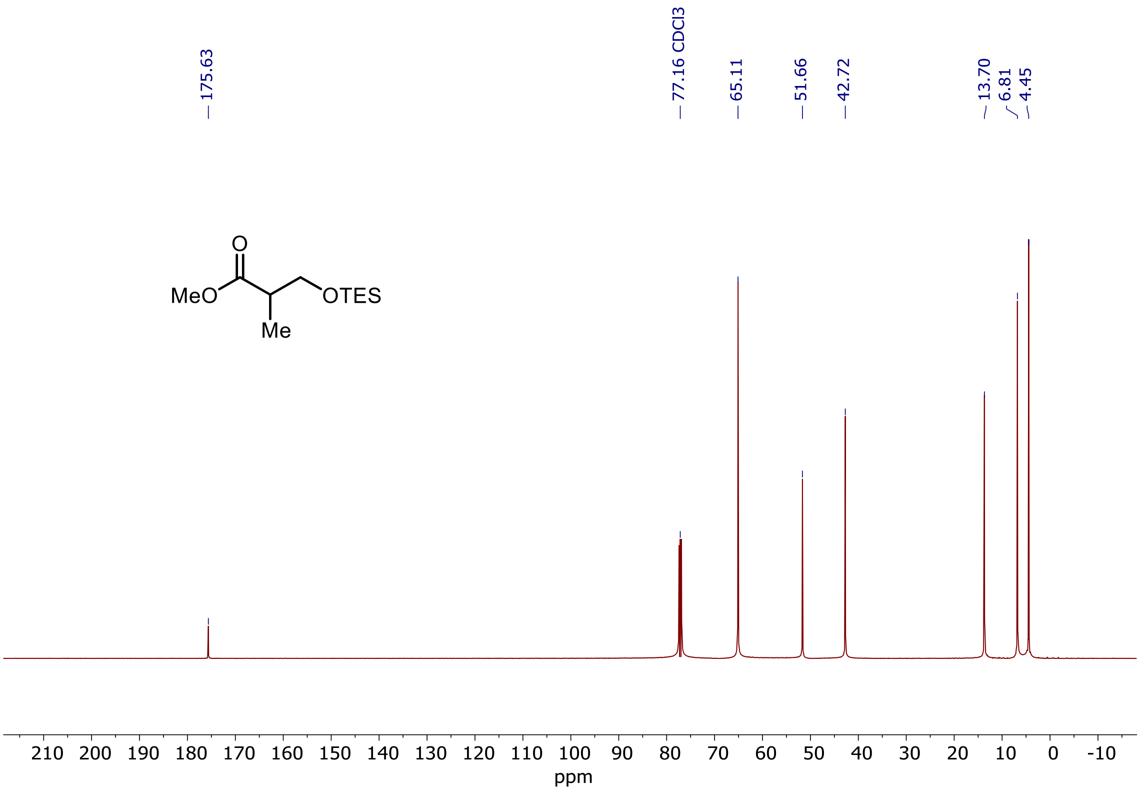

^{1}H NMR (400 MHz, $CDCl_3$) of **N-Methoxy-N,2-dimethyl-3-((triethylsilyl)oxy)propanamide: 2d**
(*see procedure*)

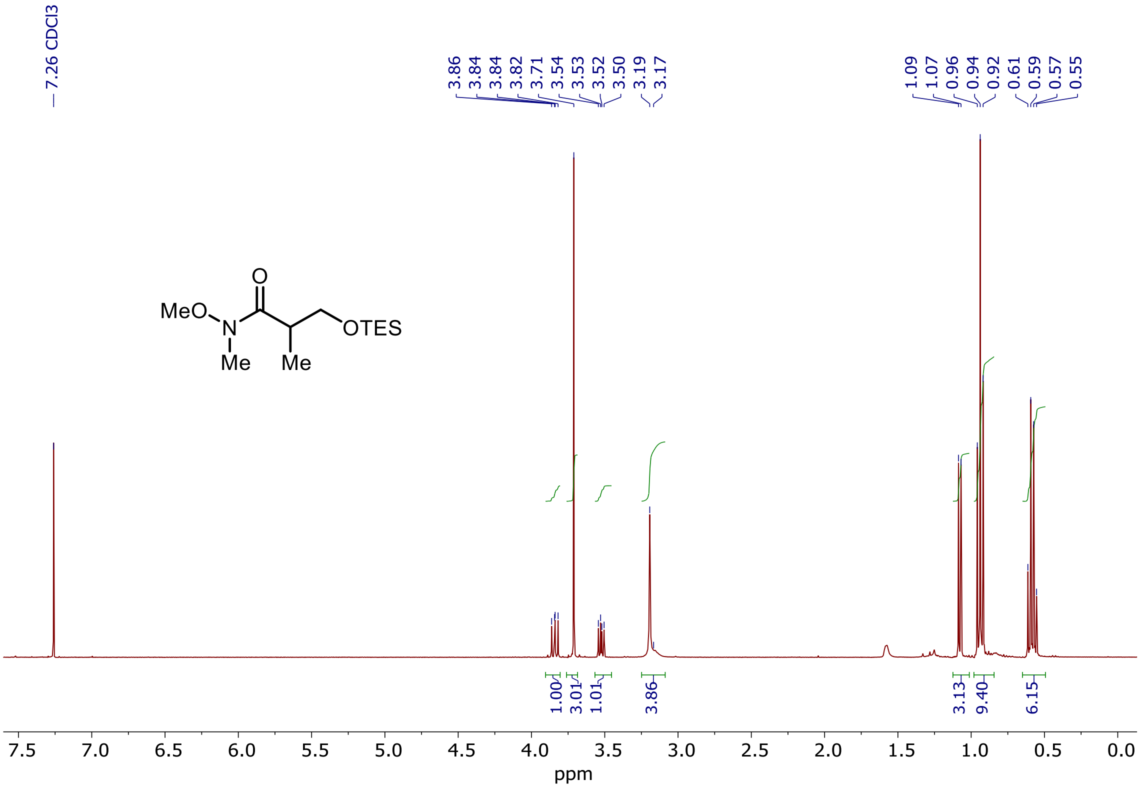

^{13}C NMR (101 MHz, $CDCl_3$) of **N-Methoxy-N,2-dimethyl-3-((triethylsilyl)oxy)propanamide: 2d**

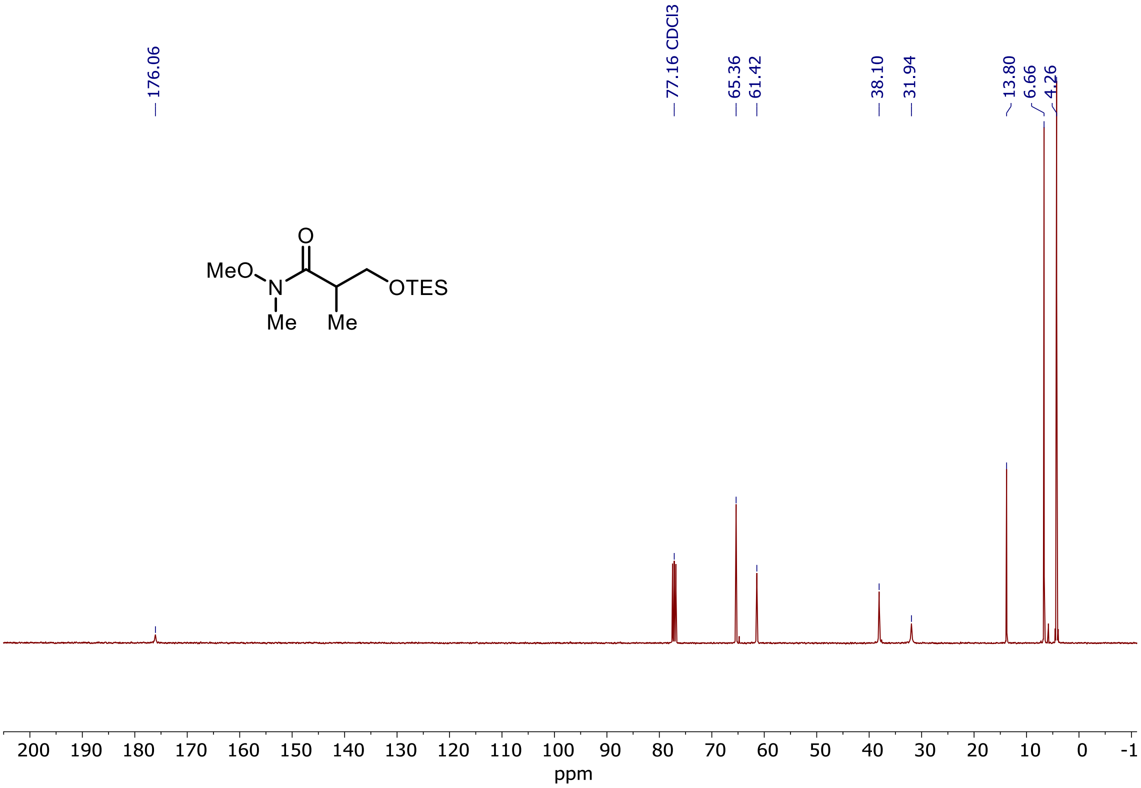

^{1}H NMR (500 MHz, $CDCl_3$) of **N-Methoxy-N-methyl-2-phenyl-3-((triethylsilyl)oxy)propanamide: 2e** (*see procedure*)

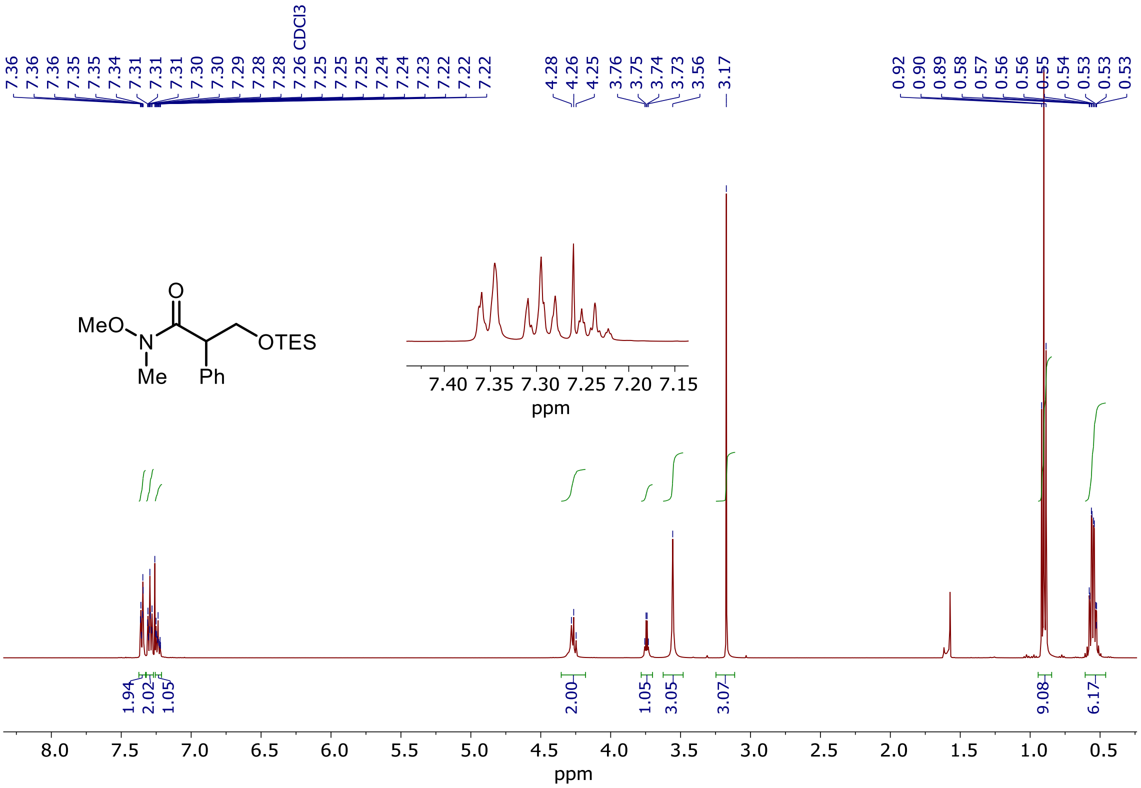

^{13}C NMR (126 MHz, $CDCl_3$) of **N-Methoxy-N-methyl-2-phenyl-3-((triethylsilyl)oxy)propanamide: 2e**

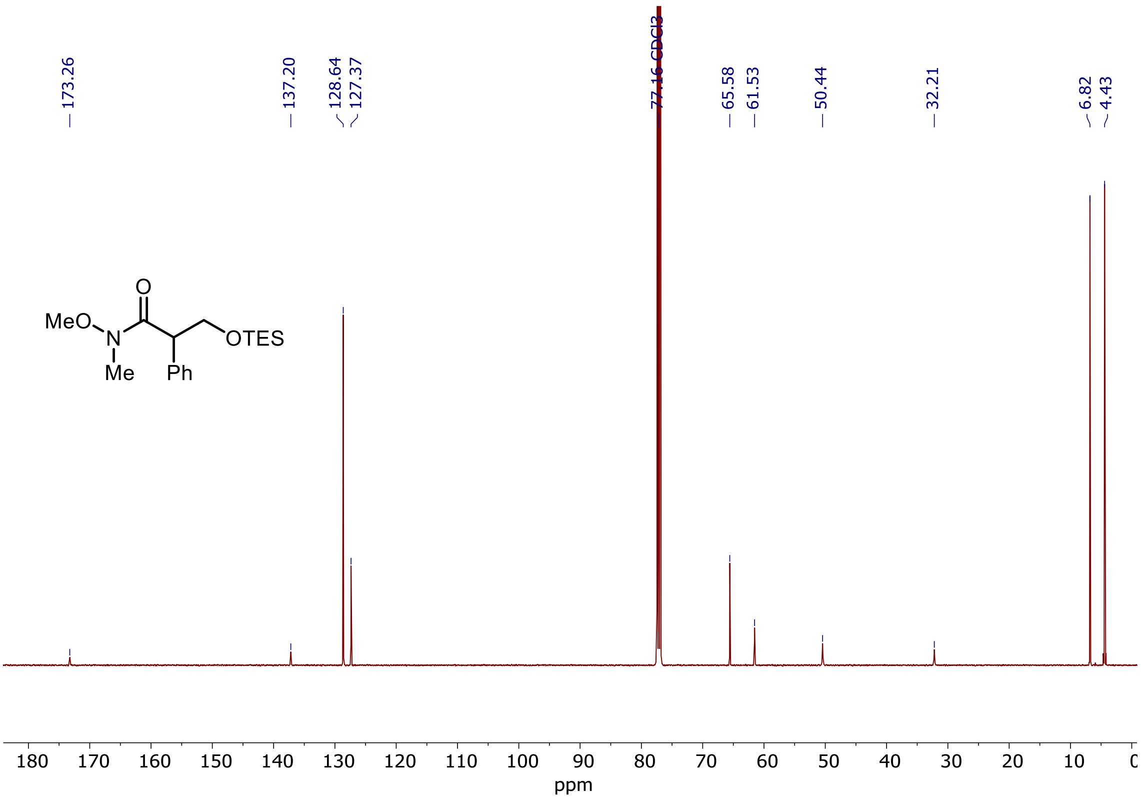

^{1}H NMR (400 MHz, $CDCl_3$) of **Methyl 2,2-dimethyl-3-((triethylsilyl)oxy)propanoate** (*see procedure*)

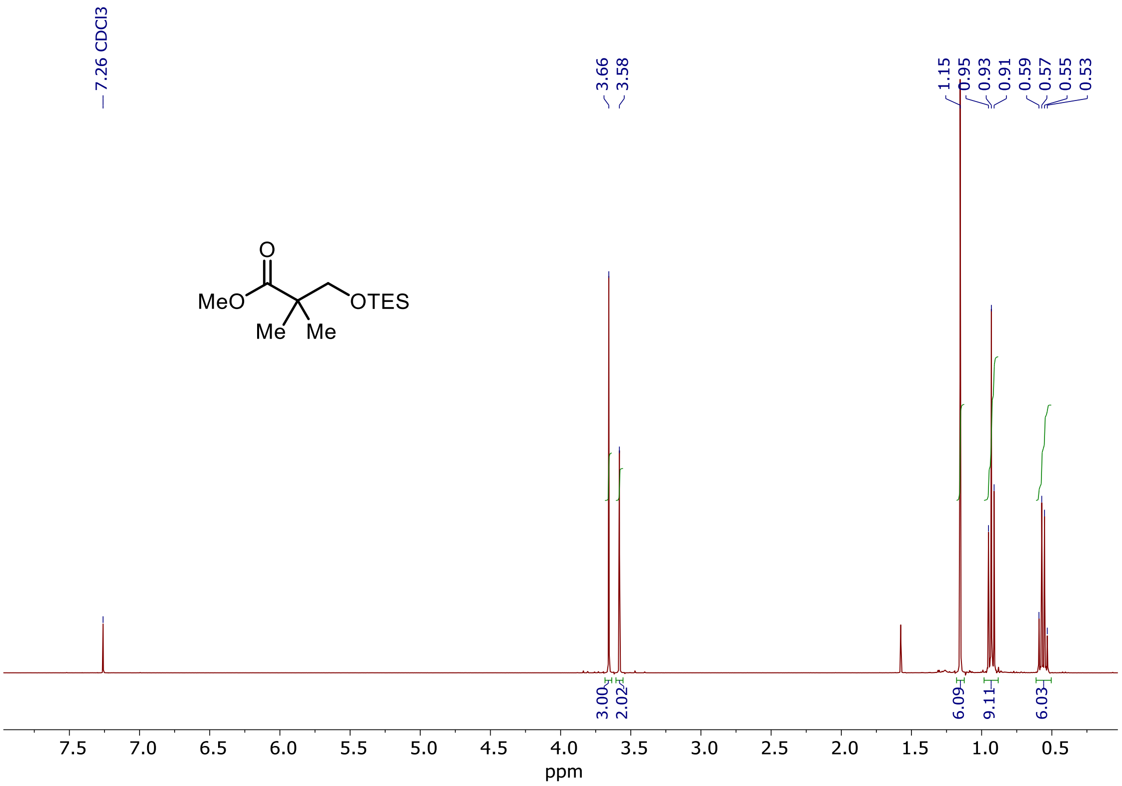

^{13}C NMR (101 MHz, $CDCl_3$) of **Methyl 2,2-dimethyl-3-((triethylsilyl)oxy)propanoate**

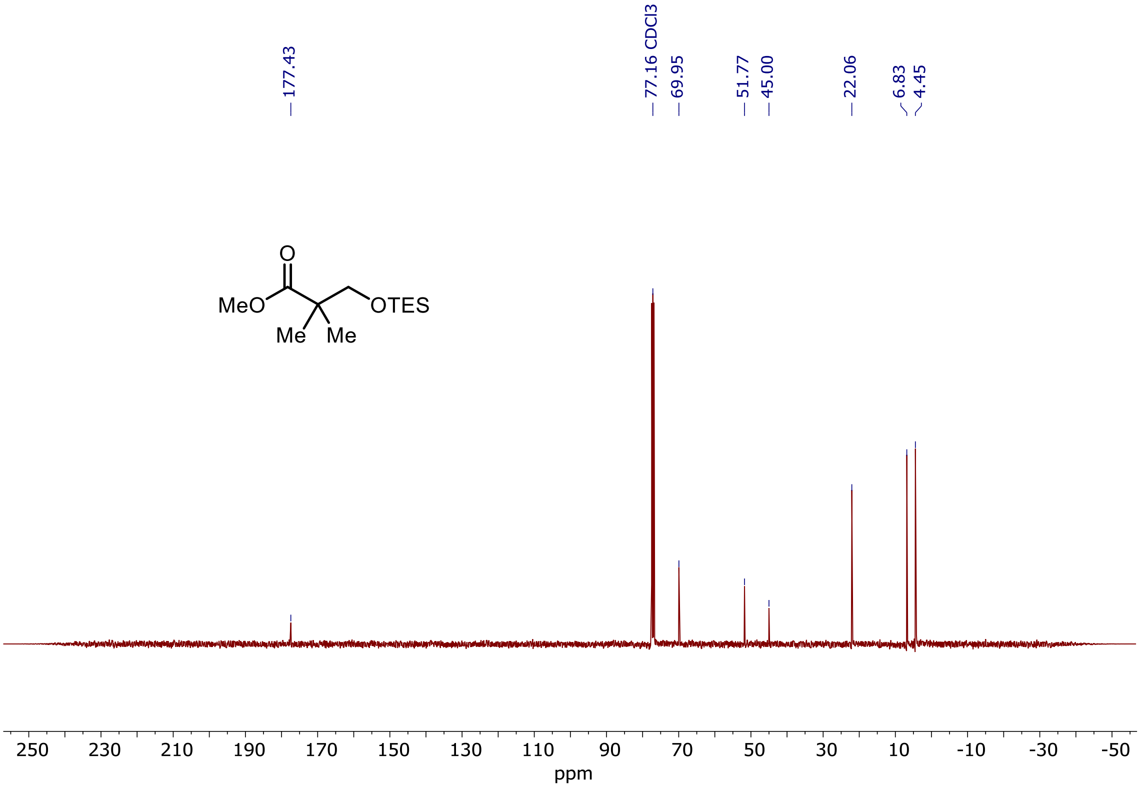

^{1}H NMR (400 MHz, $CDCl_3$) of **N-Methoxy-N,2-dimethyl-3-((triethylsilyl)oxy)propanamide: 2f**

(see procedure)

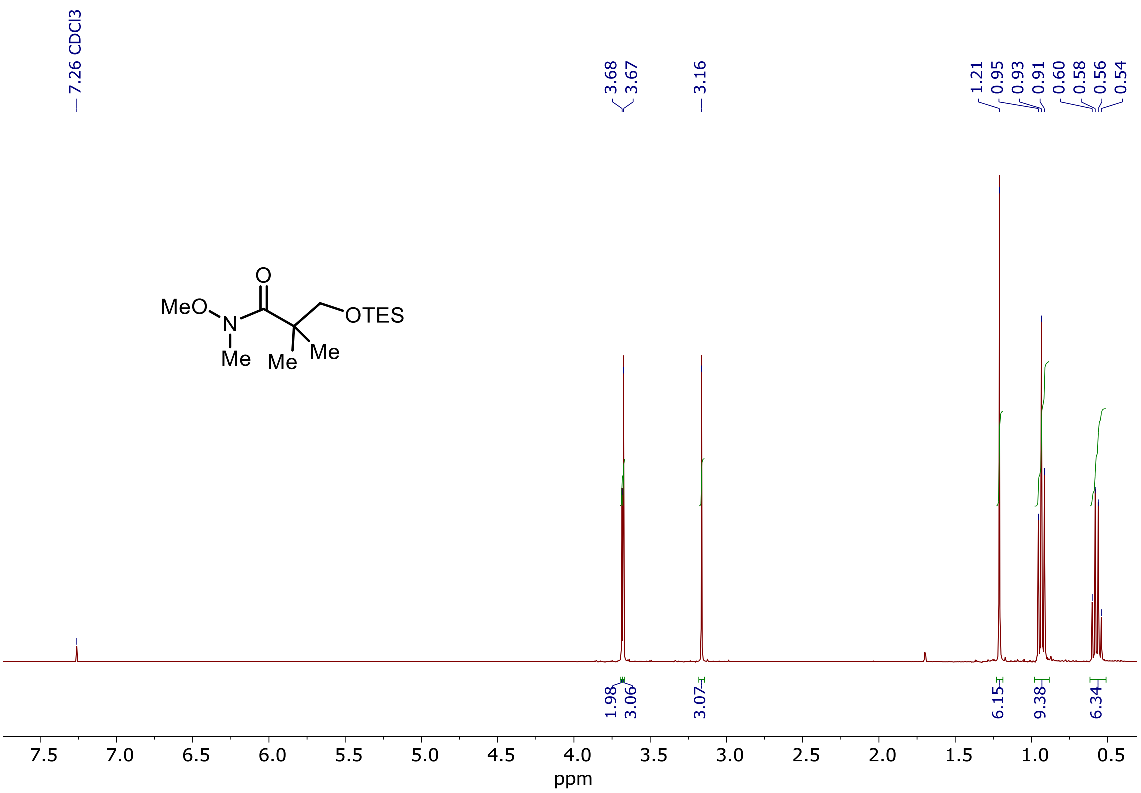

^{13}C NMR (101 MHz, $CDCl_3$) of **N-Methoxy-N,2-dimethyl-3-((triethylsilyl)oxy)propanamide: 2f**

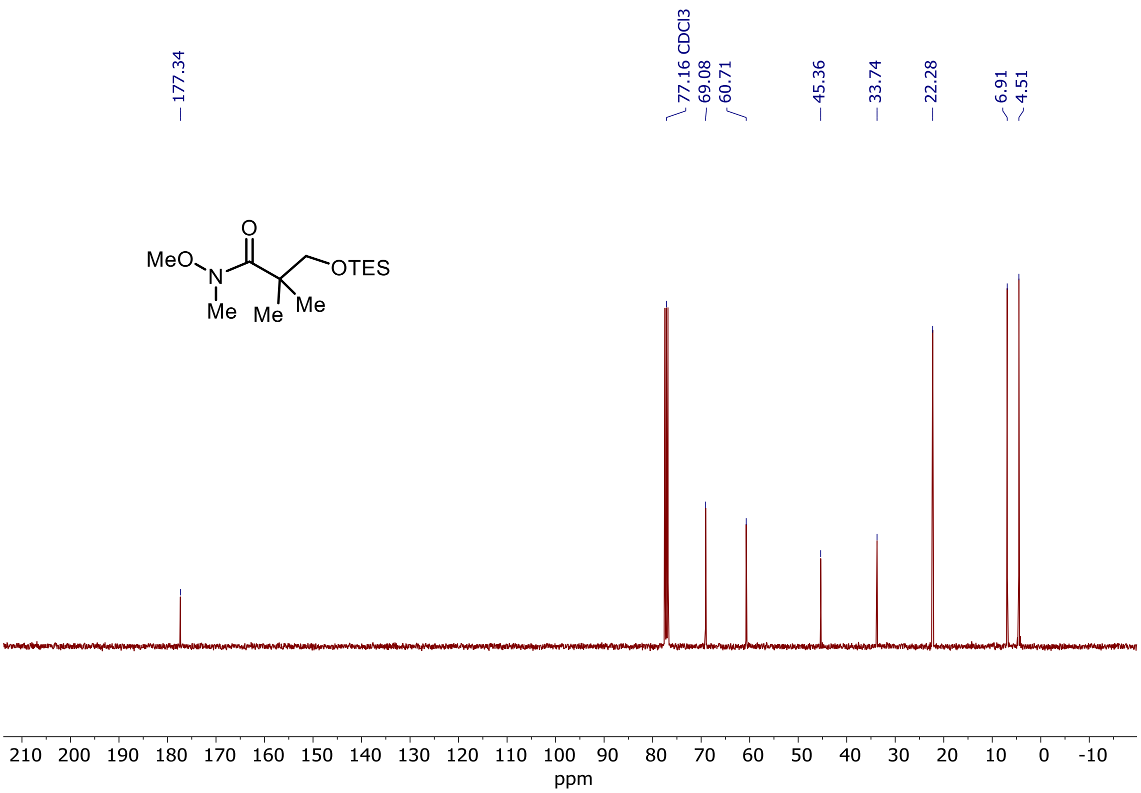

^{1}H **NMR** (400 MHz, $CDCl_3$) of **N-Methoxy-N-methyl-3-((trimethylsilyl)oxy)butanamide: 2g** (*see procedure*)

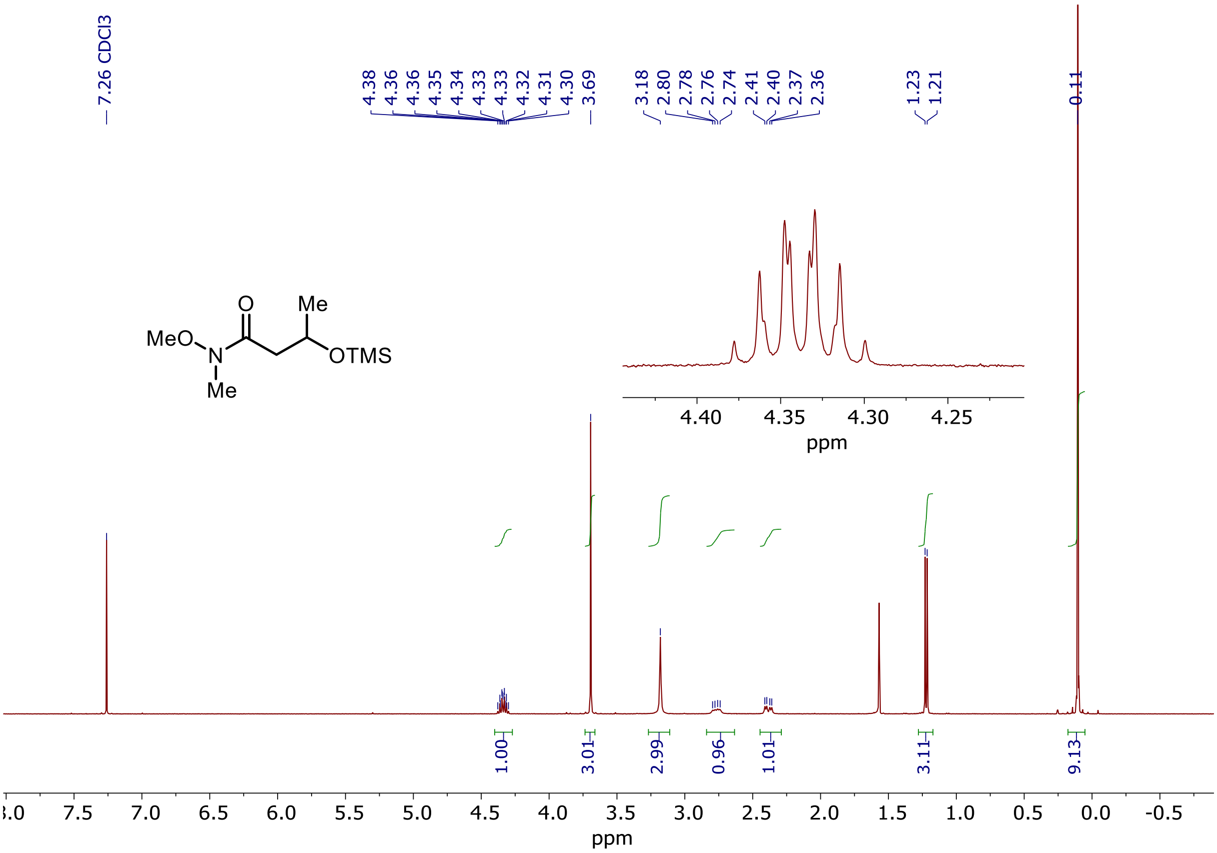

^{13}C **NMR** (126 MHz, $CDCl_3$) of **N-Methoxy-N-methyl-3-((trimethylsilyl)oxy)butanamide: 2g**

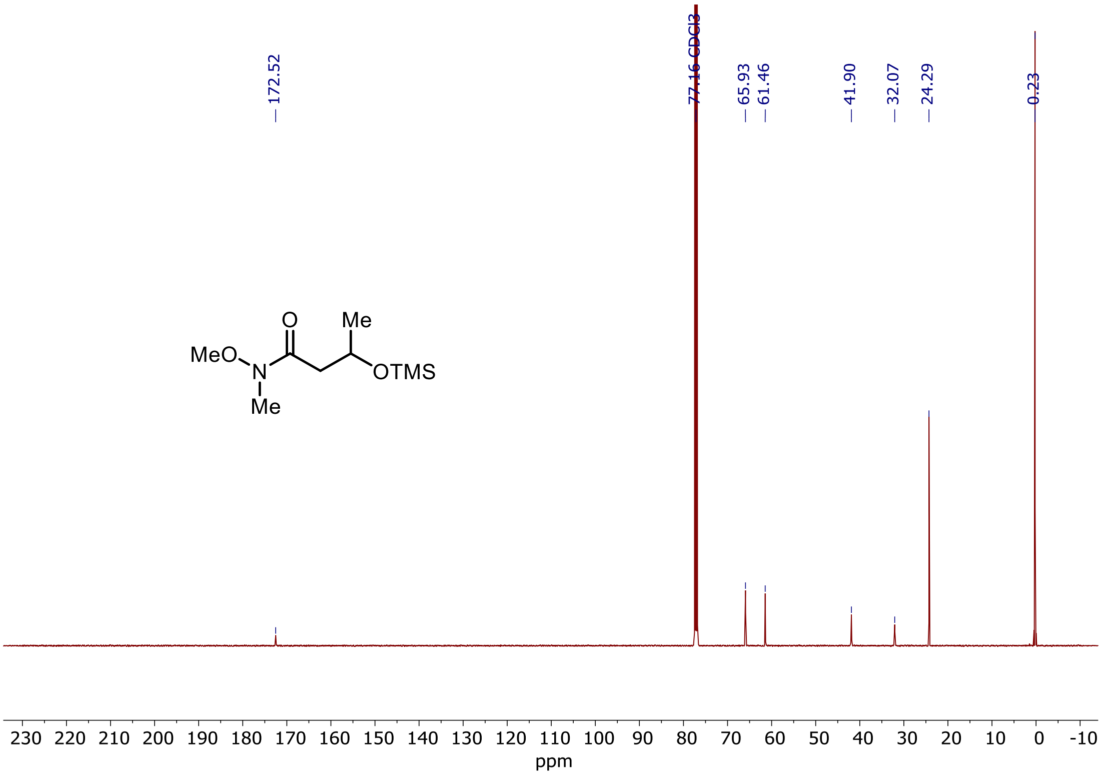

^{1}H NMR (400 MHz, $CDCl_3$) of **3-Cyclopropyl-N-methoxy-N-methyl-3-((trimethylsilyl) oxy)propanamide: 2h** (*see procedure*)

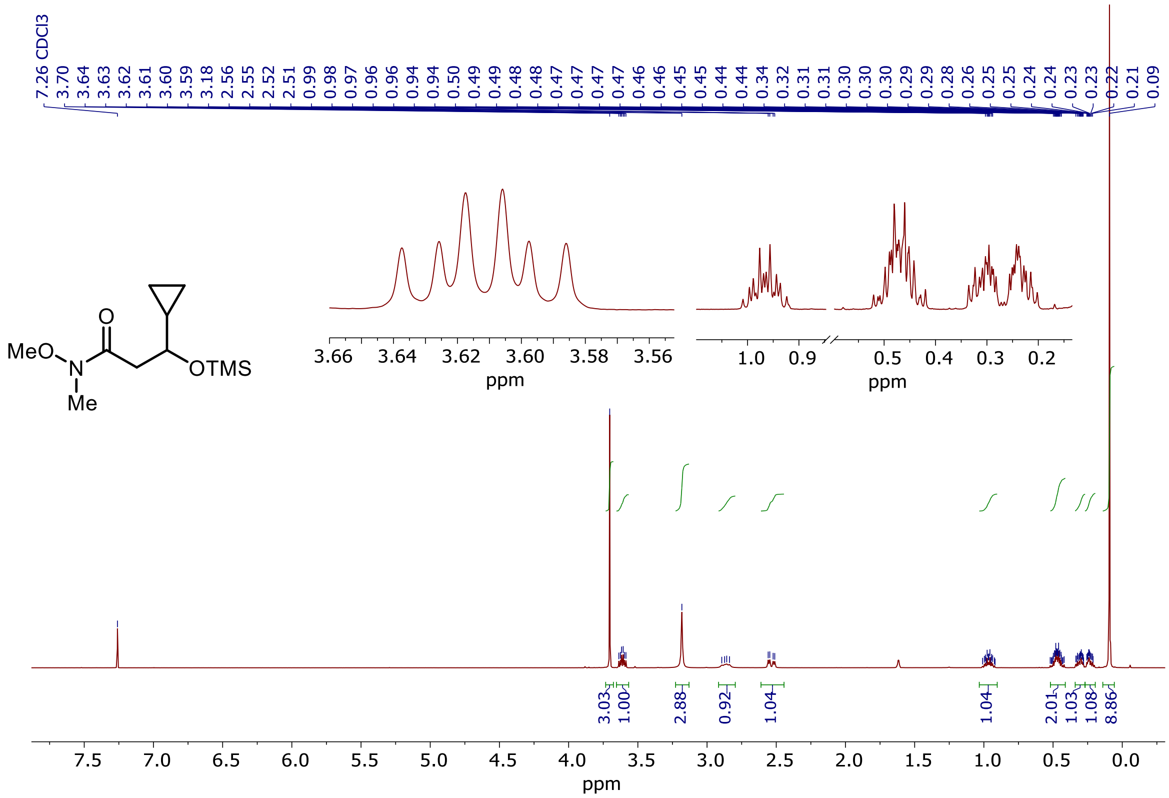

^{13}C NMR (126 MHz, $CDCl_3$) of **3-Cyclopropyl-N-methoxy-N-methyl-3-((trimethylsilyl)oxy)propanamide: 2h**

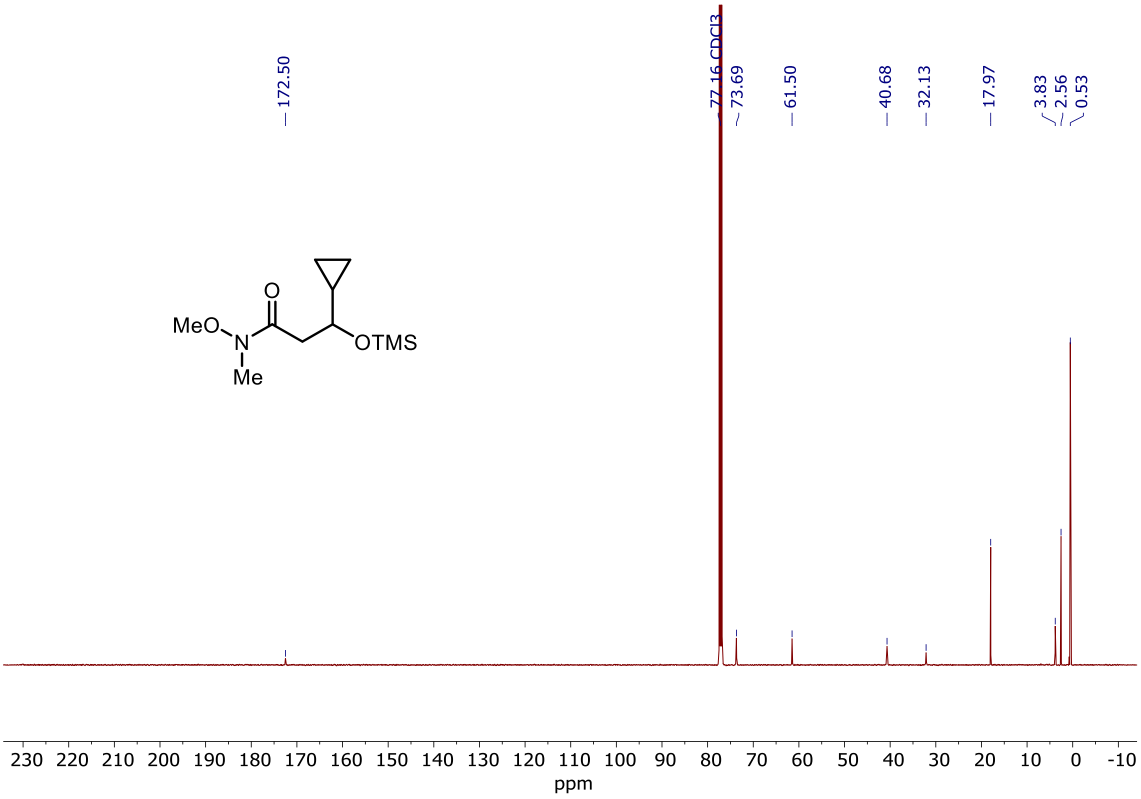

^{1}H NMR (400 MHz, $CDCl_3$) of **N-Methoxy-N,4-dimethyl-3-((trimethylsilyl)oxy)pentanamide: 2i** (<u>*see procedure*</u>)

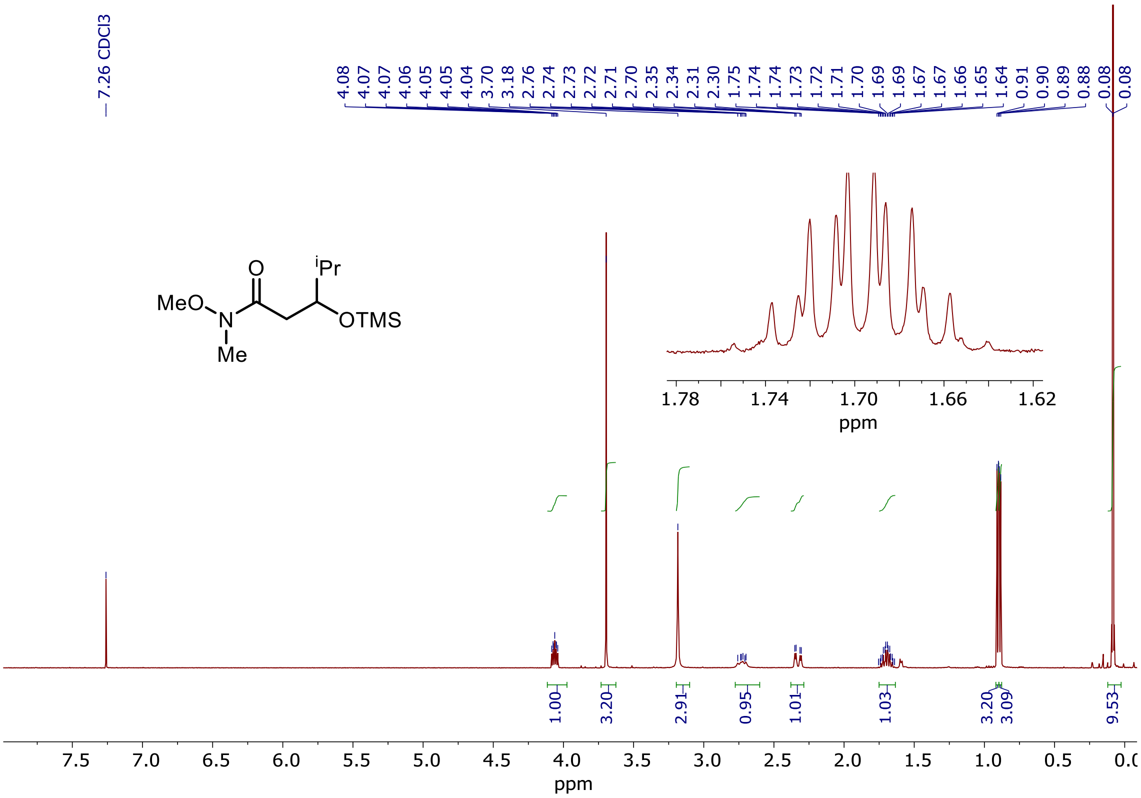

^{13}C NMR (126 MHz, $CDCl_3$) of **N-Methoxy-N,4-dimethyl-3-((trimethylsilyl)oxy)pentanamide: 2i**

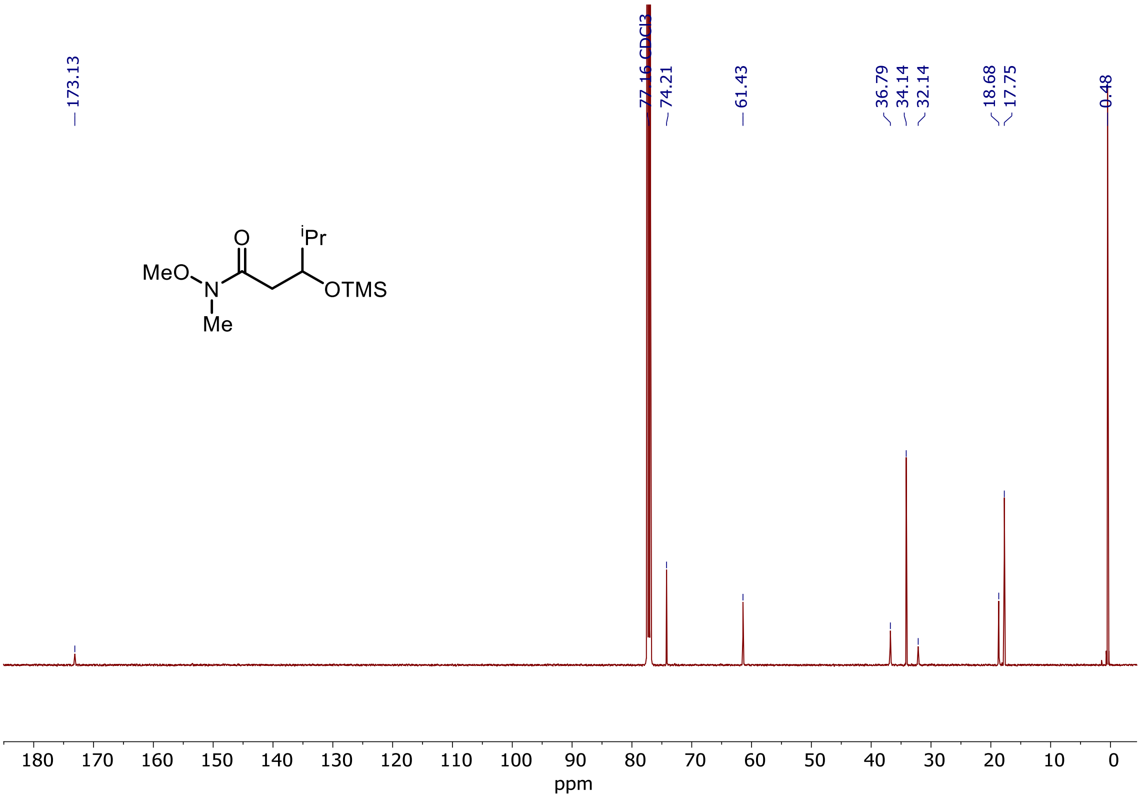

^{1}H NMR (400 MHz, $CDCl_3$) of **N-Methoxy-N-methyl-3-phenyl-3-((trimethylsilyl)oxy)propanamide: 2j** (*see procedure*)

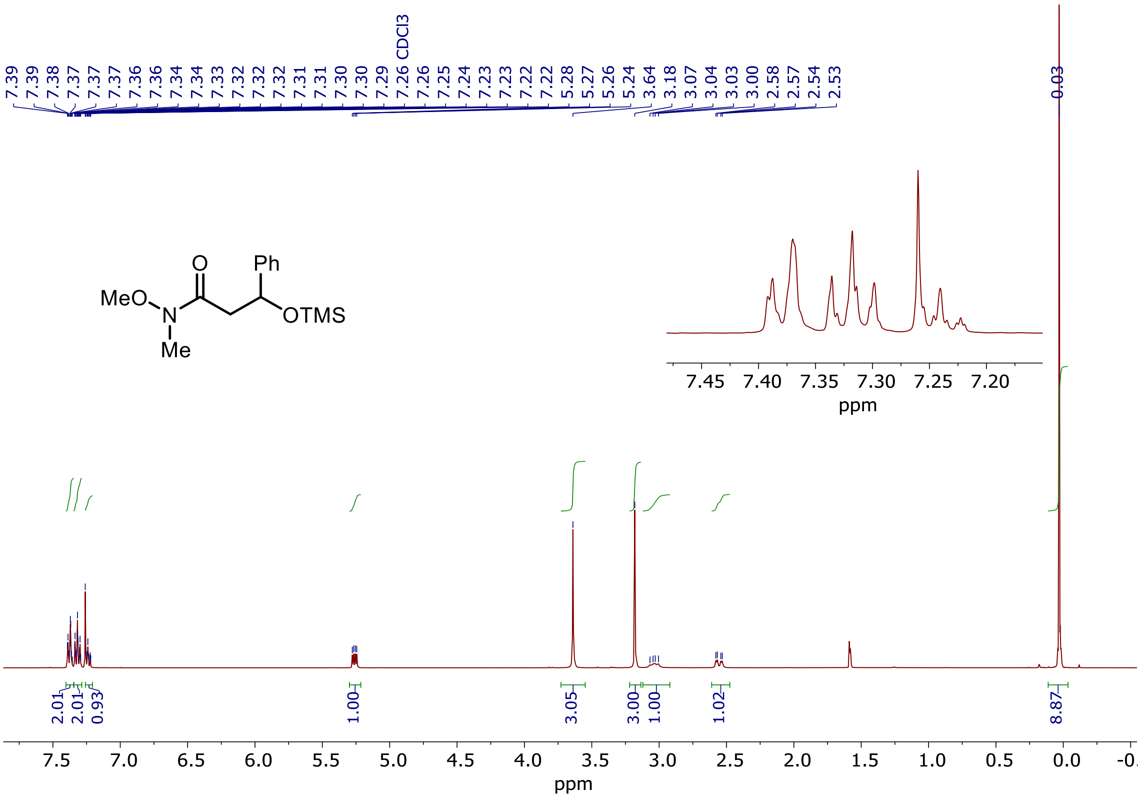

^{13}C NMR (126 MHz, $CDCl_3$) of **N-Methoxy-N-methyl-3-phenyl-3-((trimethylsilyl)oxy)propanamide: 2j**

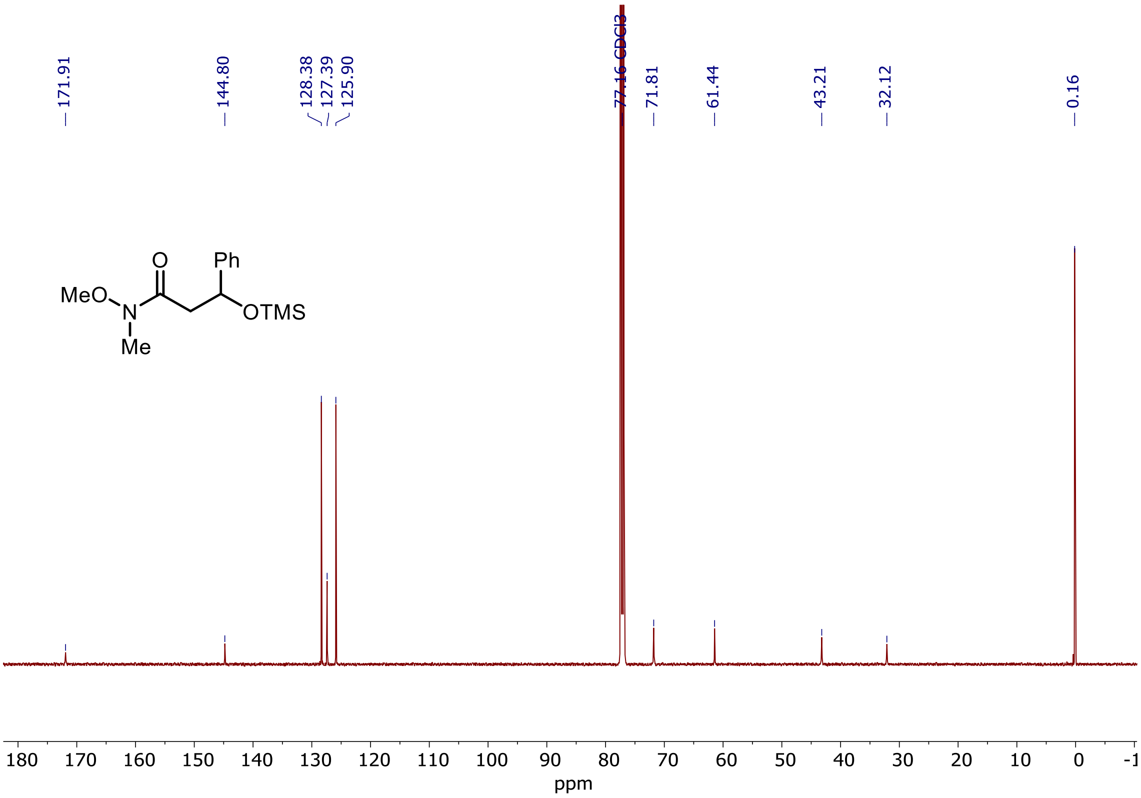

^{1}H NMR (400 MHz, $CDCl_3$) of N-Methoxy-N-methyl-3-(pyridin-3-yl)-3-((trimethylsilyl)oxy)propanamide: 2k (*see procedure*)

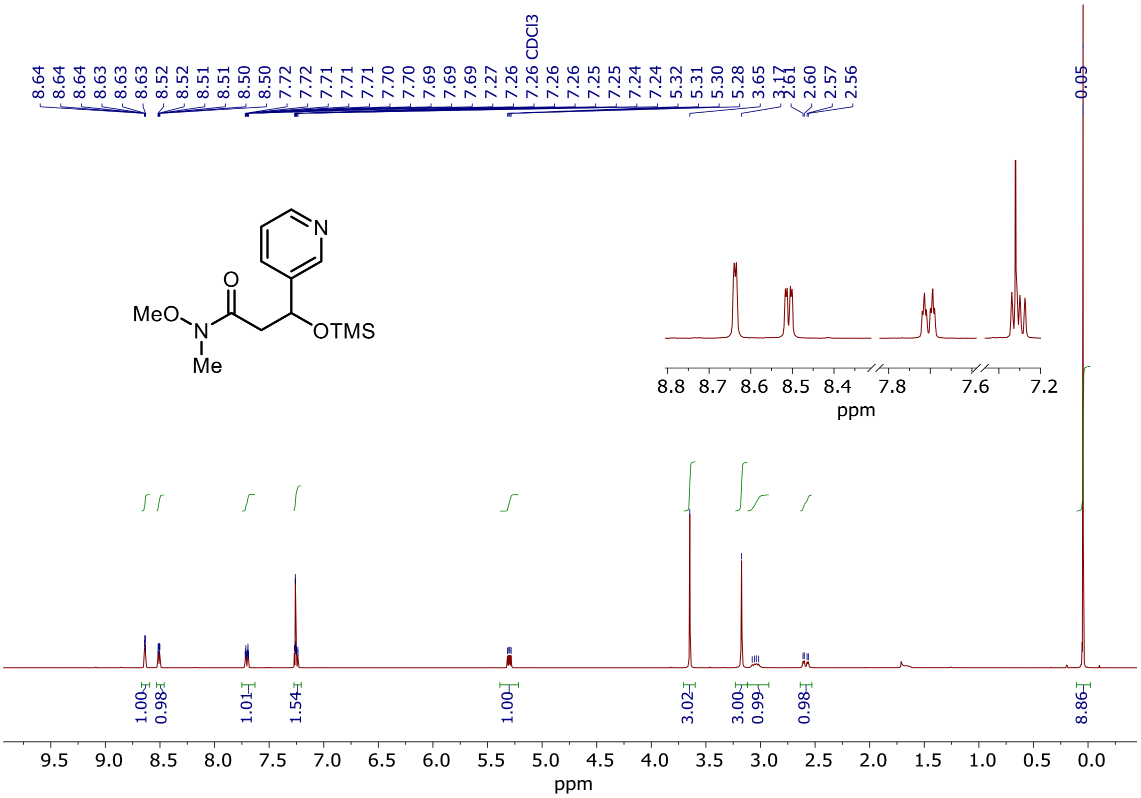

^{13}C NMR (126 MHz, $CDCl_3$) of N-Methoxy-N-methyl-3-(pyridin-3-yl)-3-((trimethylsilyl)oxy)propanamide: 2k

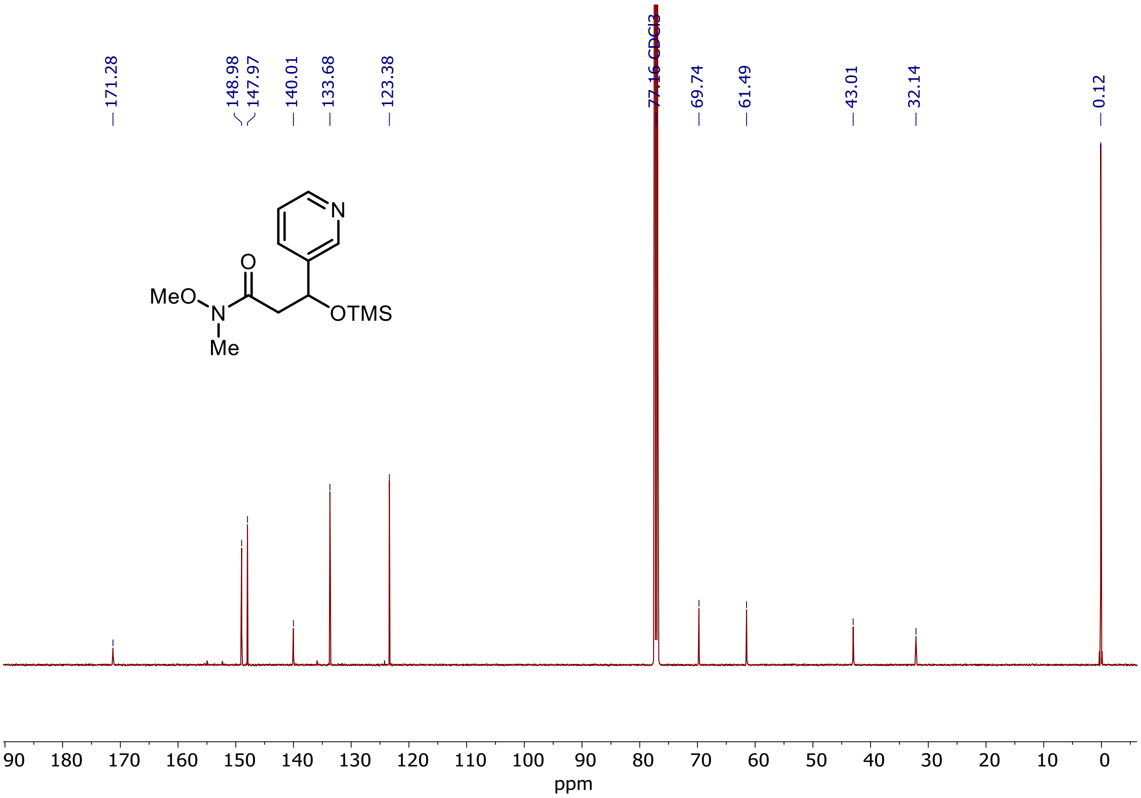

^{1}H NMR (400 MHz, $CDCl_3$) of **N-Methoxy-N-methyl-3-((trimethylsilyl)oxy)pent-4-enamide: 2l** (*see procedure*)

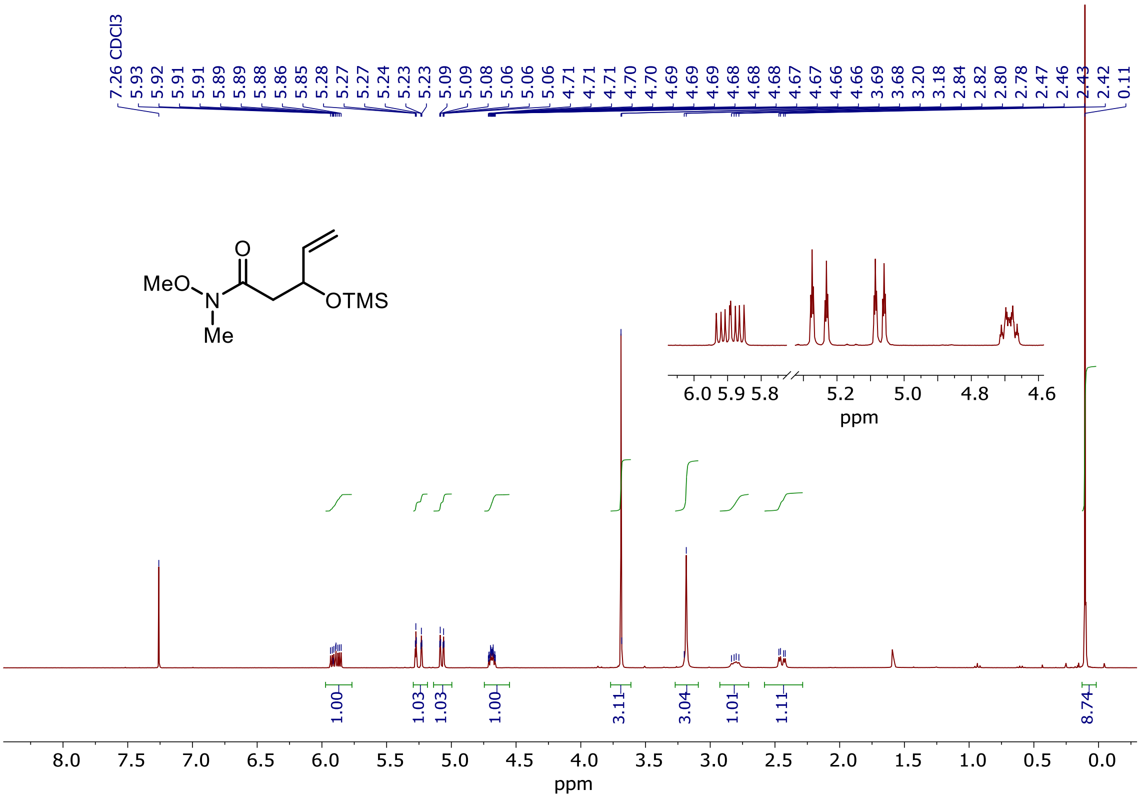

^{13}C NMR (126 MHz, $CDCl_3$) of **N-Methoxy-N-methyl-3-((trimethylsilyl)oxy)pent-4-enamide: 2l**

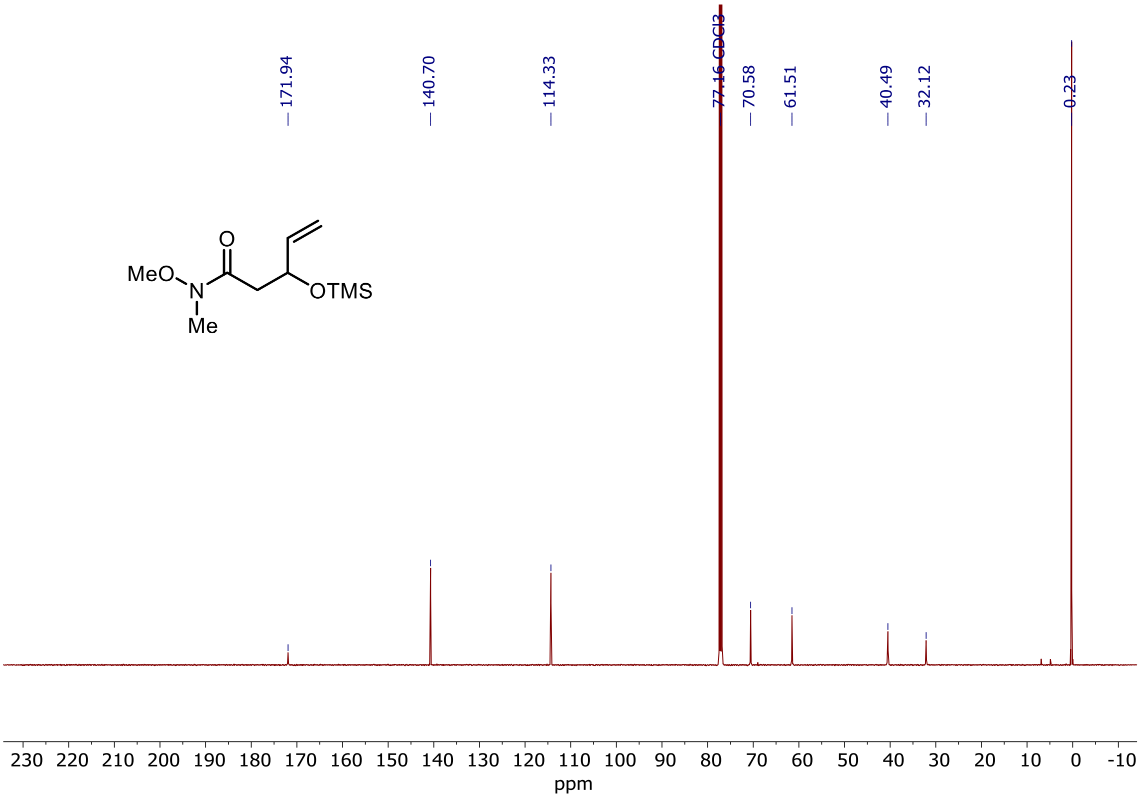

[1]**H NMR** (400 MHz, $CDCl_3$) of **4-Chloro-N-methoxy-N-methyl-3-((trimethylsilyl)oxy)butanamide: 2m** (*see procedure*)

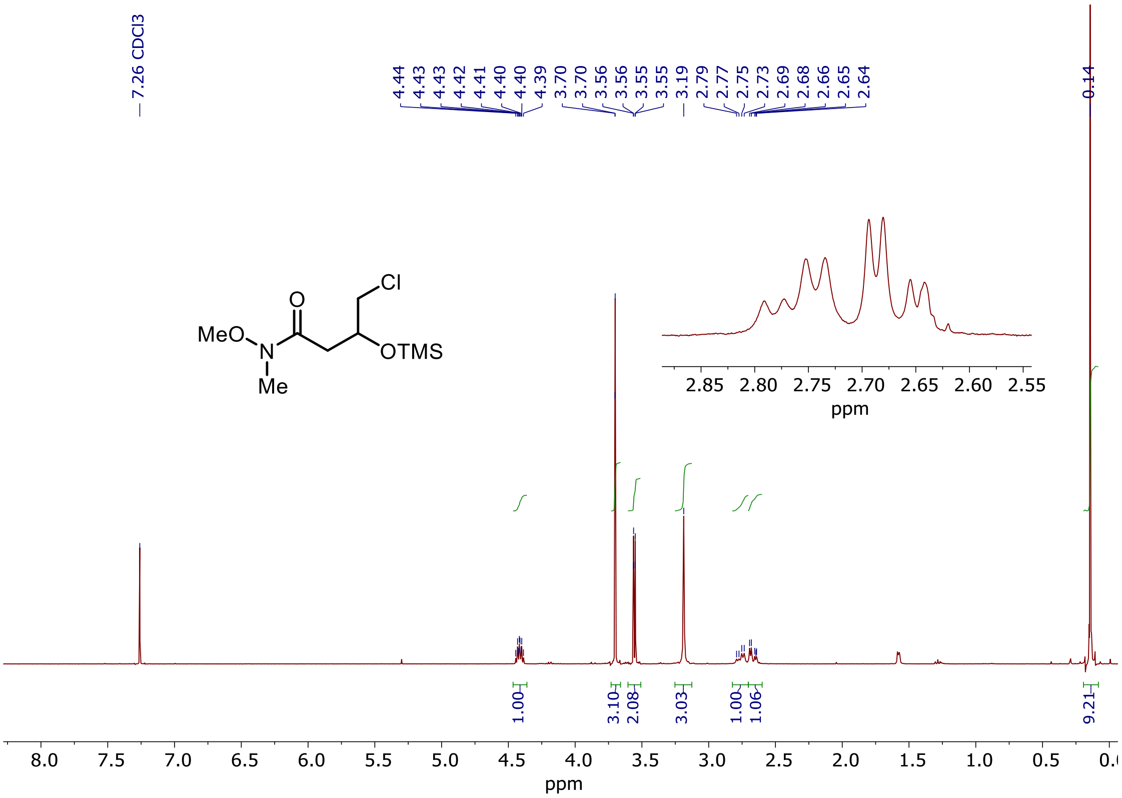

[13]**C NMR** (126 MHz, $CDCl_3$) of **4-Chloro-N-methoxy-N-methyl-3-((trimethylsilyl)oxy)butanamide: 2m**

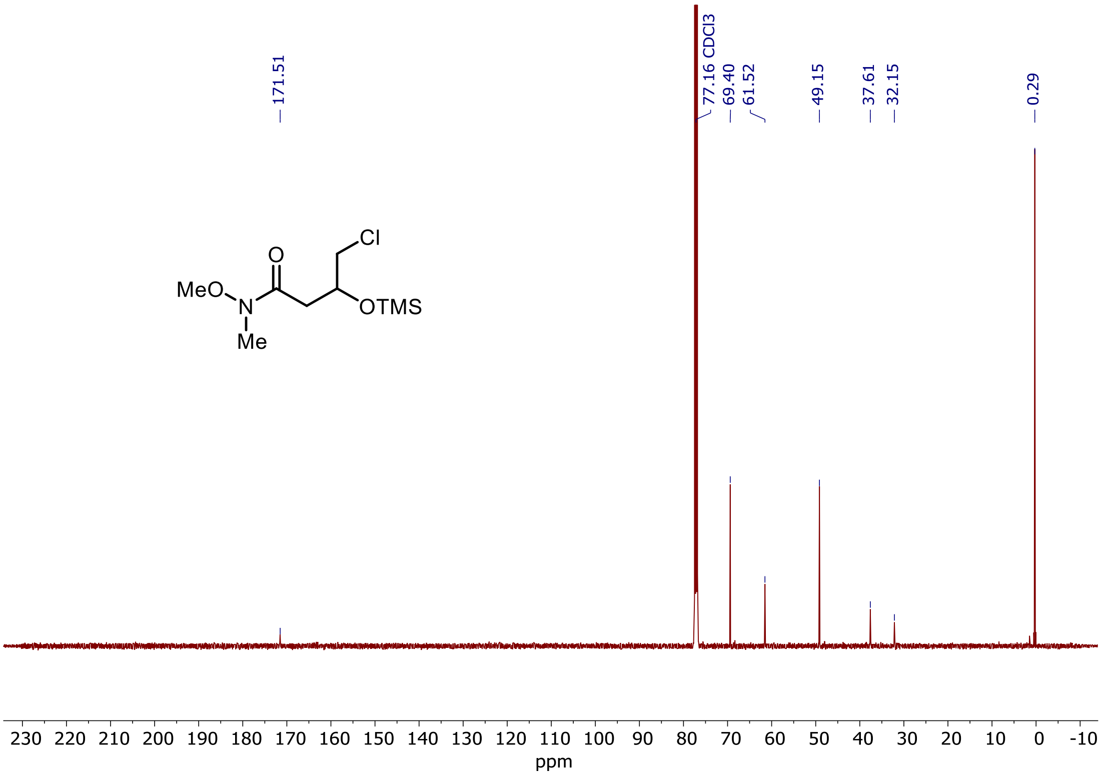

^{1}H NMR (400 MHz, $CDCl_3$) of **4-(Benzyloxy)-N-methoxy-N-methyl-3-((trimethylsilyl)oxy)butanamide: 2n** (*see procedure*)

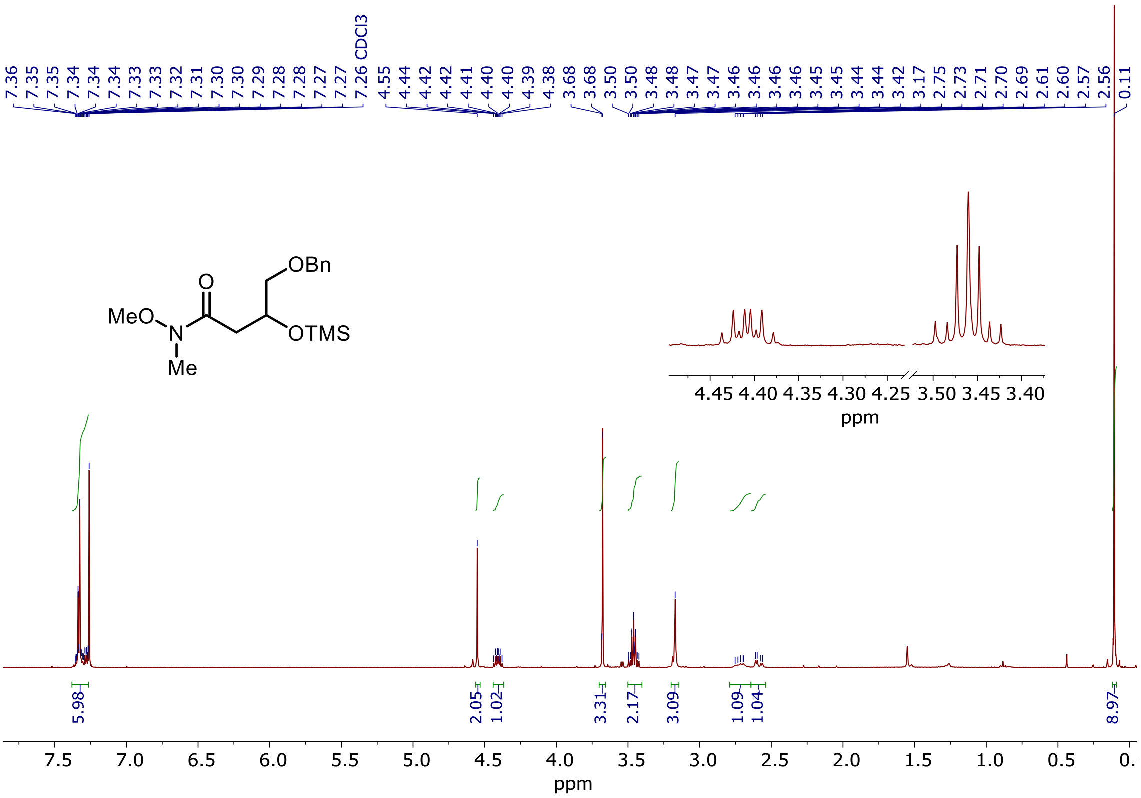

^{13}C NMR (126 MHz, $CDCl_3$) of **4-(Benzyloxy)-N-methoxy-N-methyl-3-((trimethylsilyl)oxy)butanamide: 2n**

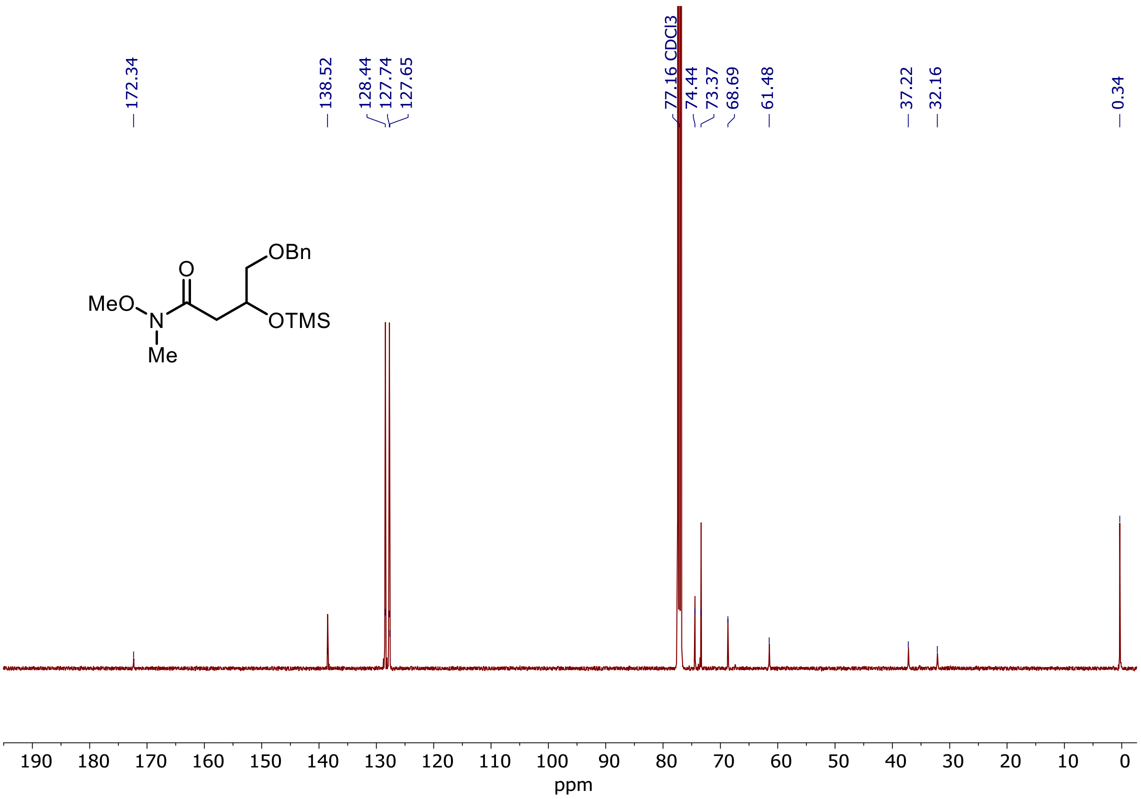

^{1}H NMR (400 MHz, $CDCl_3$) of **N-Methoxy-N,3-dimethyl-3-((trimethylsilyl)oxy)butanamide: 2o**

(*see procedure*)

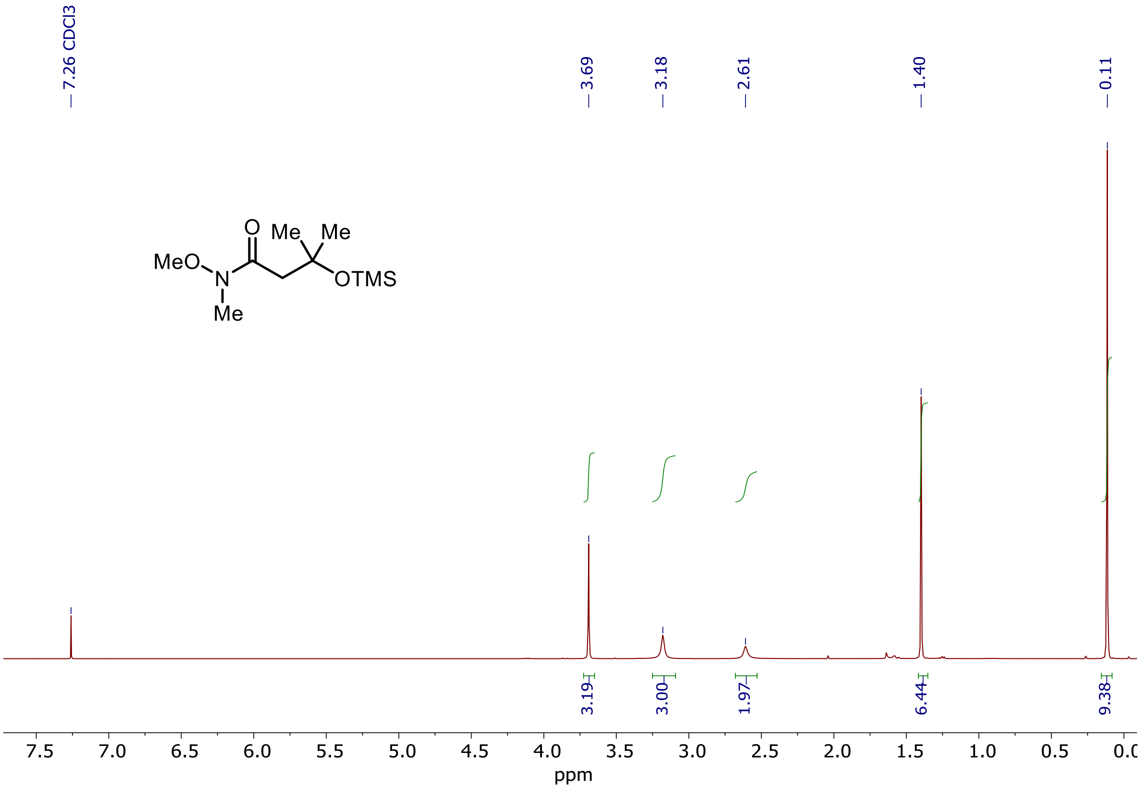

^{13}C NMR (126 MHz, $CDCl_3$) of **N-Methoxy-N,3-dimethyl-3-((trimethylsilyl)oxy)butanamide: 2o**

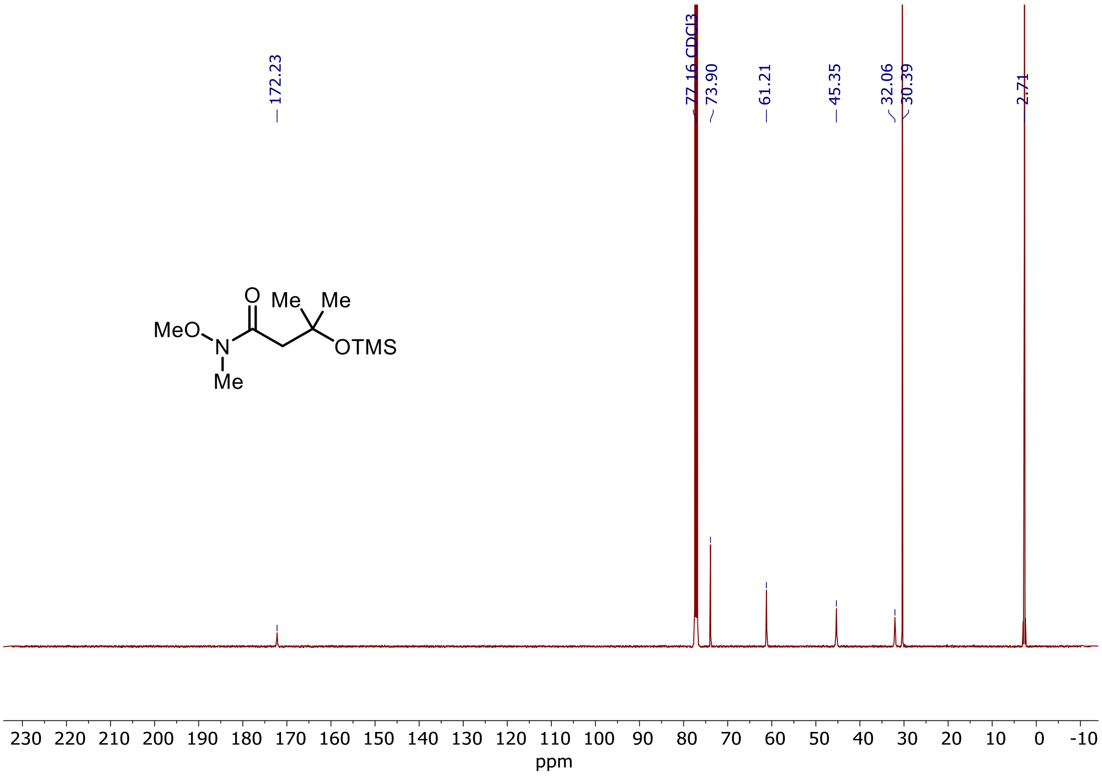

^{1}H NMR (400 MHz, $CDCl_3$) of N-Methoxy-N-methyl-2-(1-((trimethylsilyl)oxy)cyclohexyl)acetamide: 2p (*see procedure*)

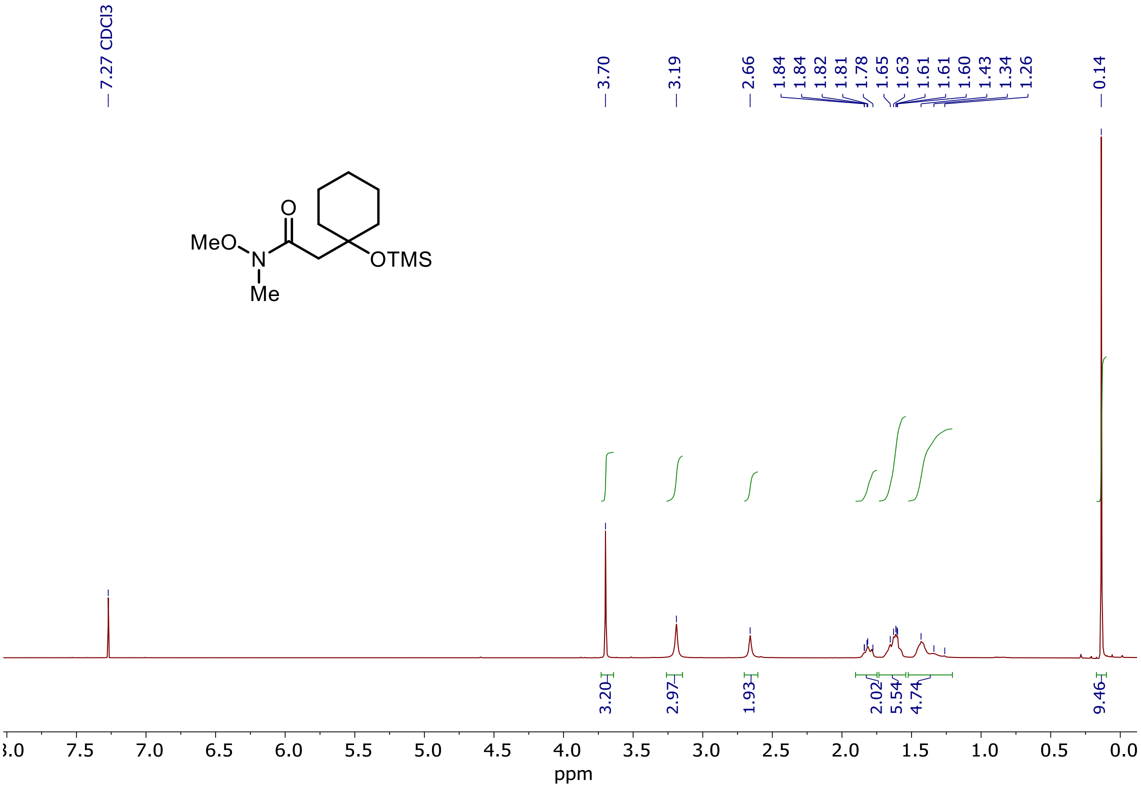

^{13}C NMR (126 MHz, $CDCl_3$) of N-Methoxy-N-methyl-2-(1-((trimethylsilyl)oxy)cyclohexyl)acetamide: 2p

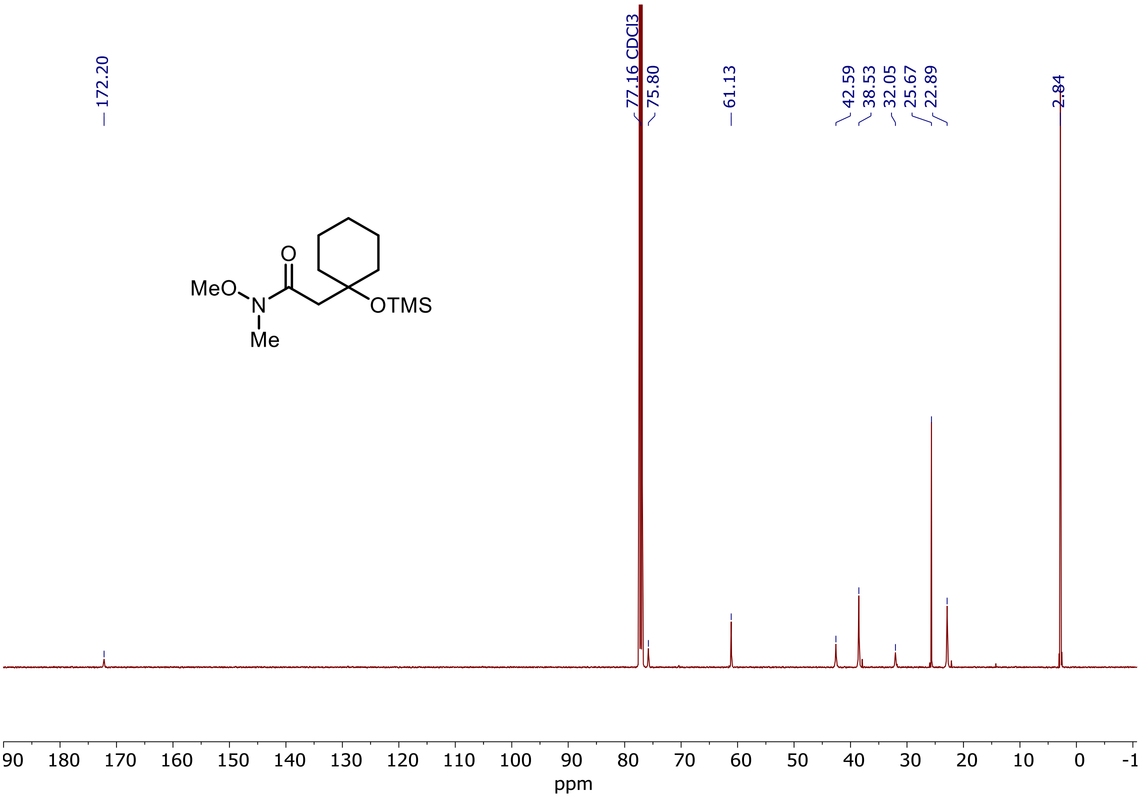

^{1}H NMR (400 MHz, $CDCl_3$) of *syn*-**Ethyl 2-methyl-3-((trimethylsilyl)oxy)pent-4-enoate** (*see procedure*)

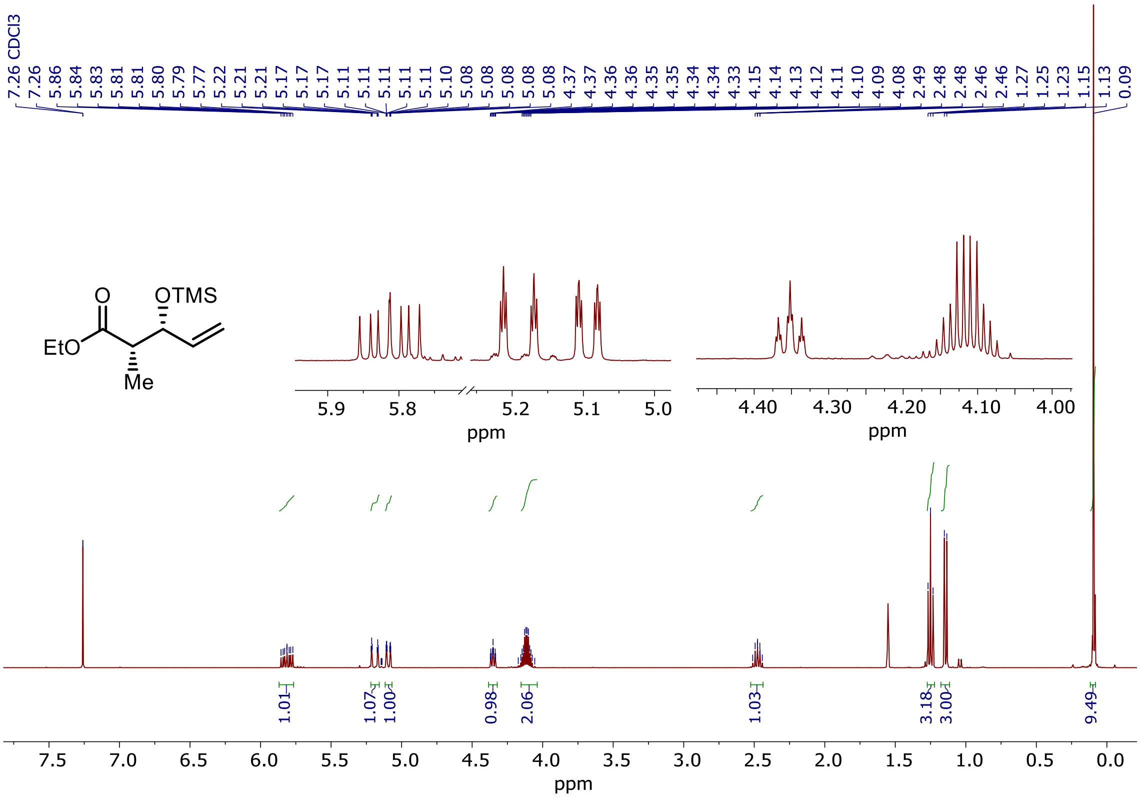

^{13}C NMR (126 MHz, $CDCl_3$) of *syn*-**Ethyl 2-methyl-3-((trimethylsilyl)oxy)pent-4-enoate**

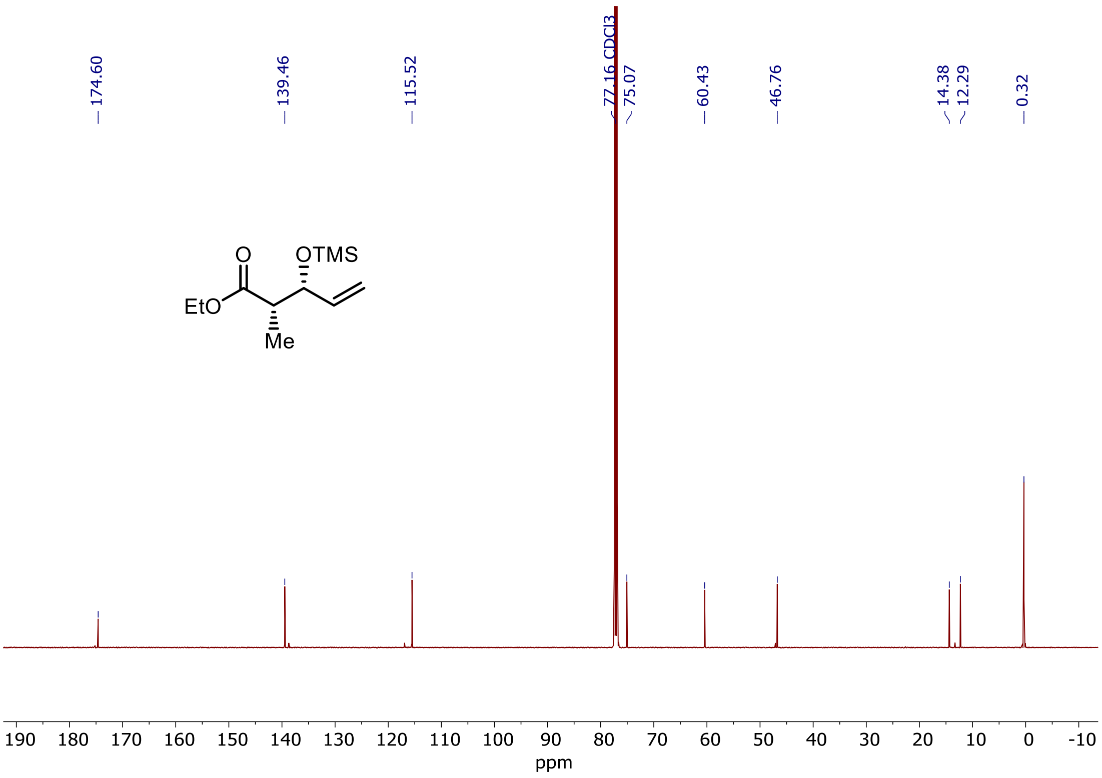

^{1}H NMR (400 MHz, $CDCl_3$) of *syn*-**N-Methoxy-N,2-dimethyl-3-((trimethylsilyl)oxy)pent-4-enamide:** *syn*-**2q** (*see procedure*)

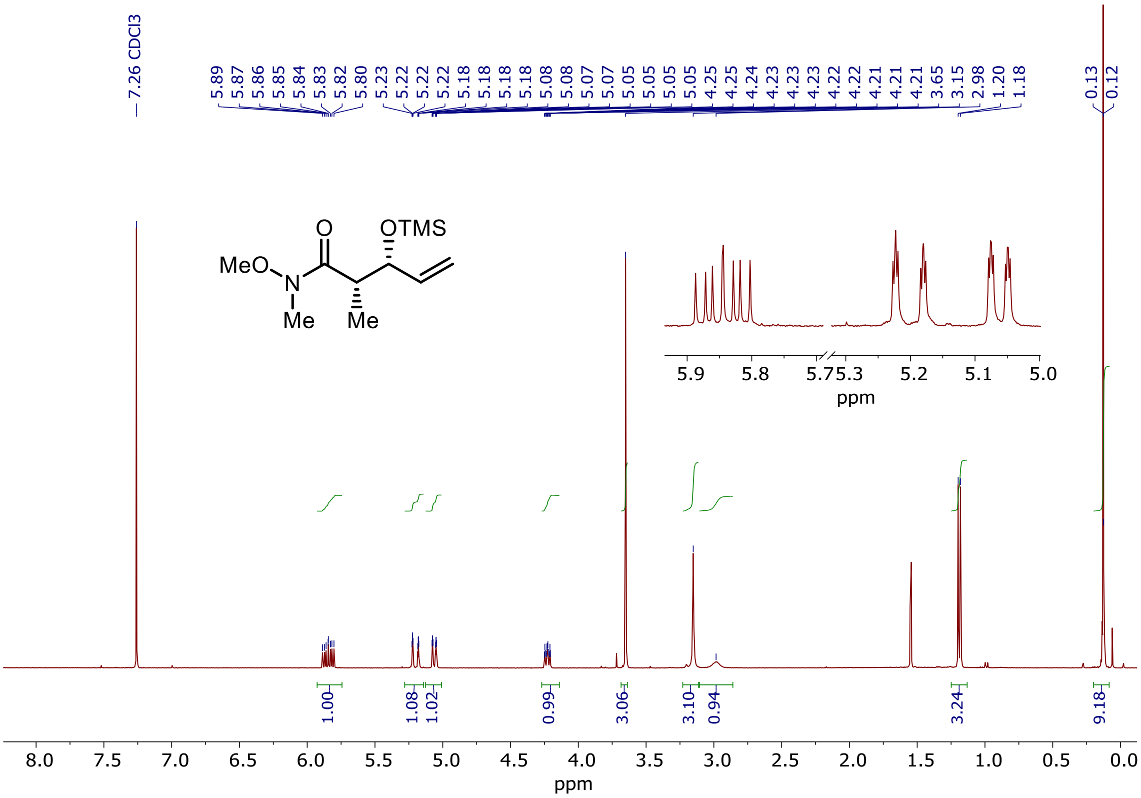

^{13}C NMR (126 MHz, $CDCl_3$) of *syn*-**N-Methoxy-N,2-dimethyl-3-((trimethylsilyl)oxy)pent-4-enamide:** *syn*-**2q**

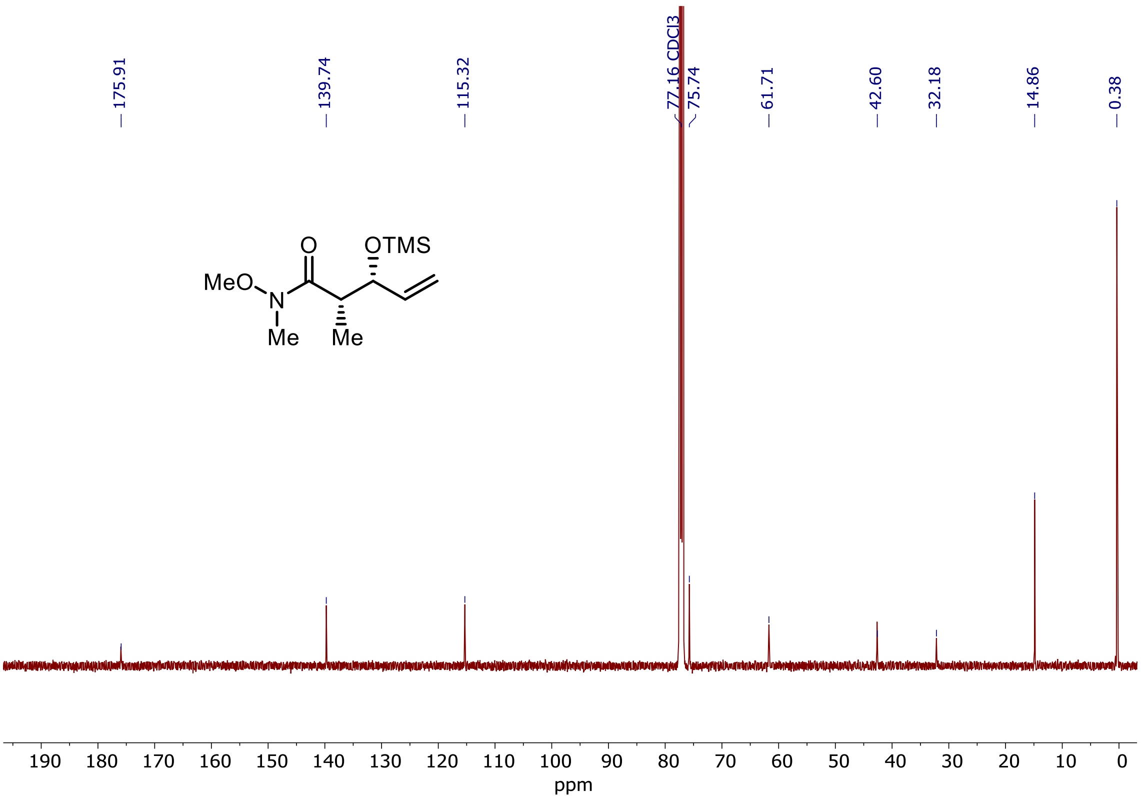

^{1}H NMR (400 MHz, $CDCl_3$) of *anti*-**Ethyl 2-methyl-3-((trimethylsilyl)oxy)pent-4-enoate** (*see procedure*)

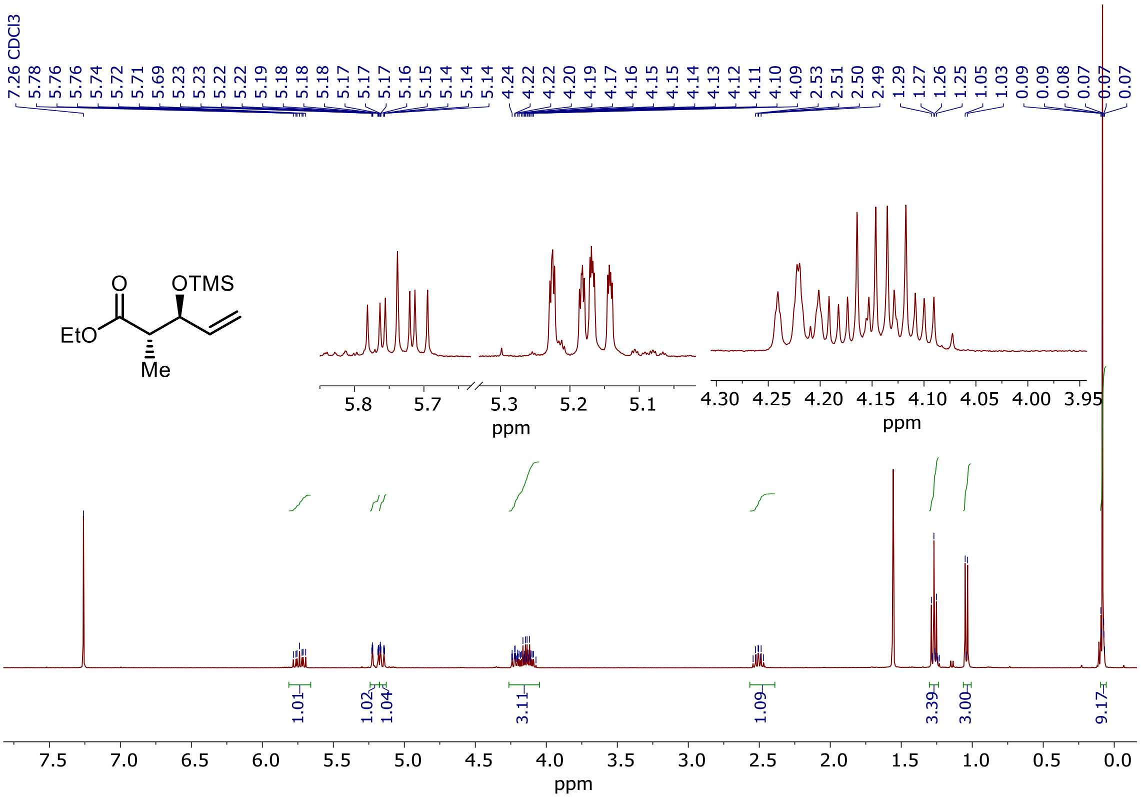

^{13}C NMR (126 MHz, $CDCl_3$) of *anti*-**Ethyl 2-methyl-3-((trimethylsilyl)oxy)pent-4-enoate**

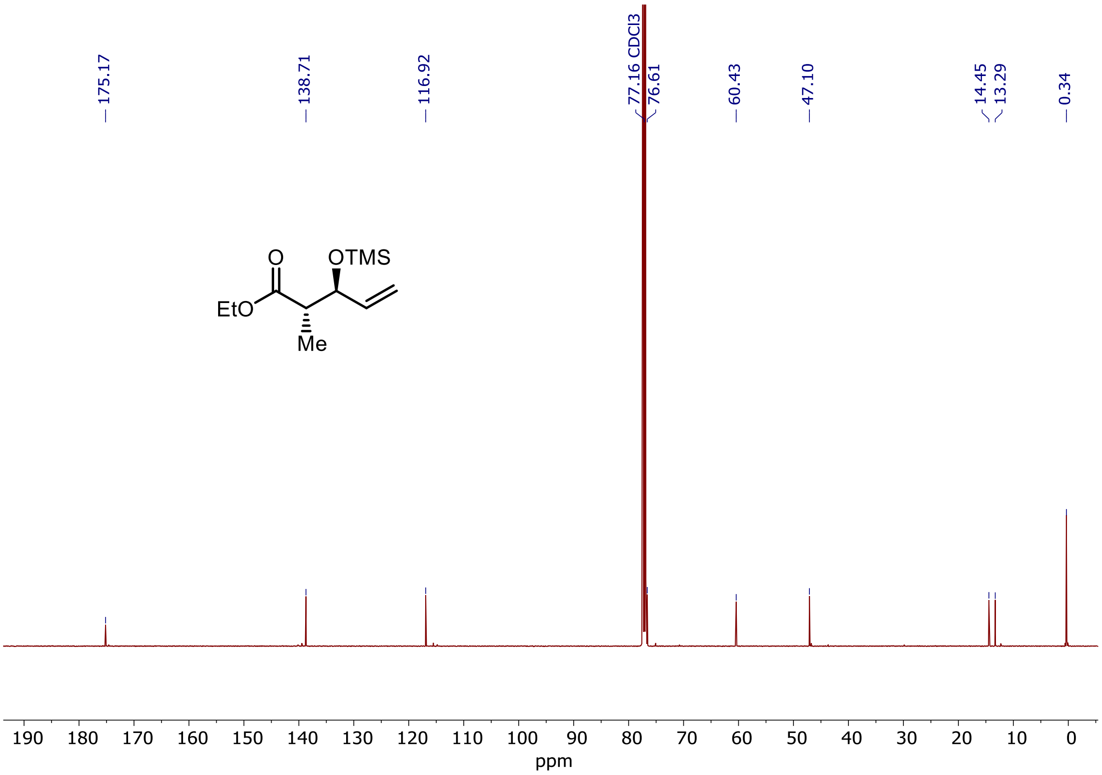

^{1}H NMR (400 MHz, $CDCl_3$) of *anti*-**N-Methoxy-N,2-dimethyl-3-((trimethylsilyl)oxy)pent-4-enamide:** *anti*-**2q** (*see procedure*)

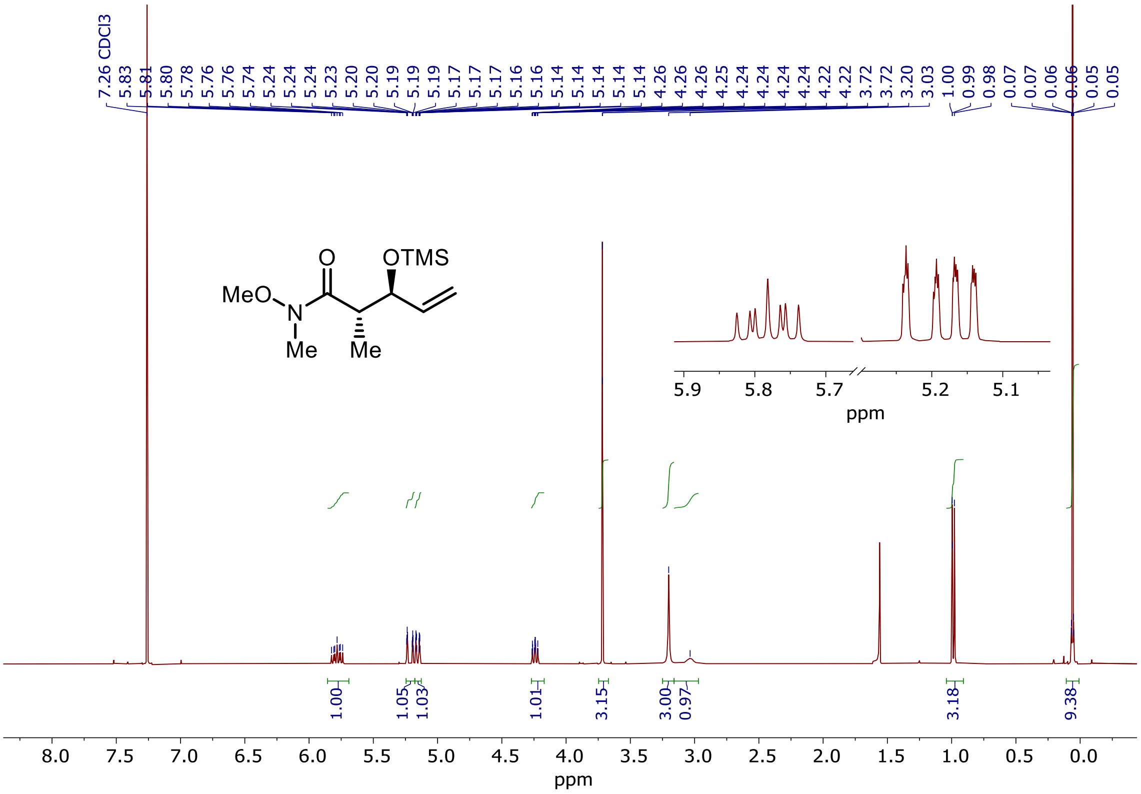

^{13}C NMR (126 MHz, $CDCl_3$) of *anti*-**N-Methoxy-N,2-dimethyl-3-((trimethylsilyl)oxy)pent-4-enamide:** *anti*-**2q**

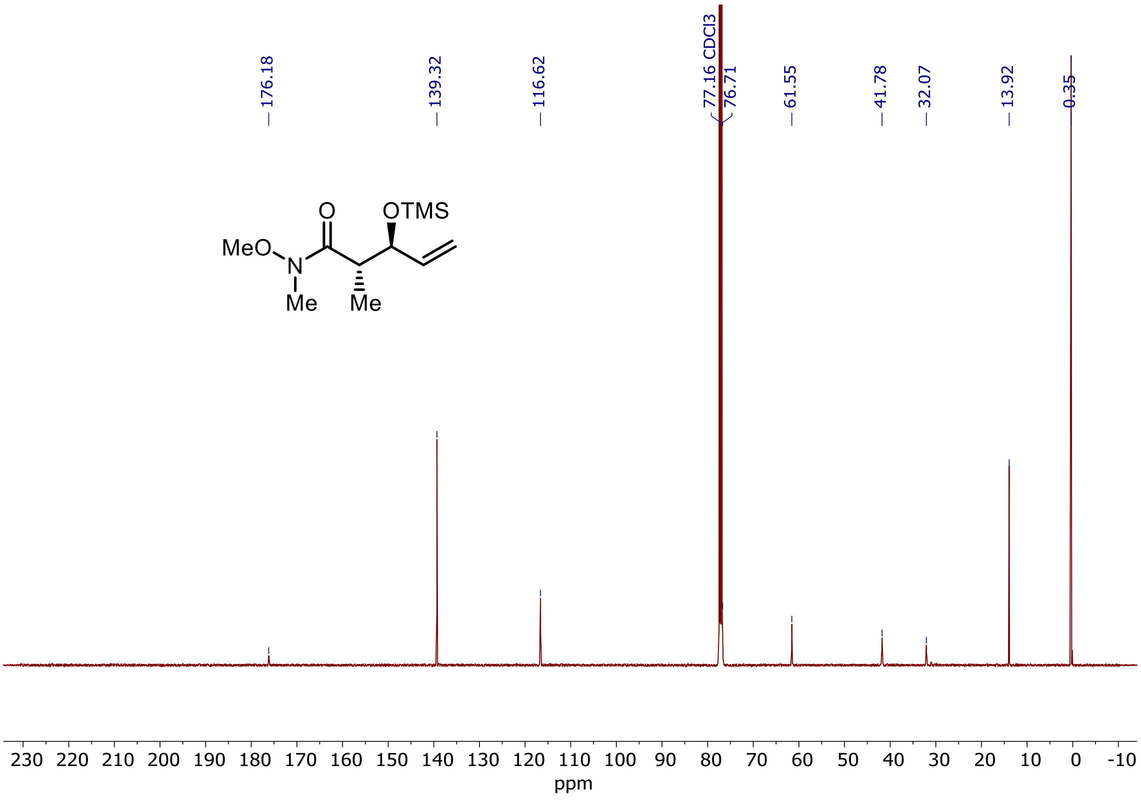

^{1}H NMR (400 MHz, $CDCl_3$) of **N-Methoxy-N-methyl-4-((trimethylsilyl)oxy)pentanamide: 2r**

(*see procedure*)

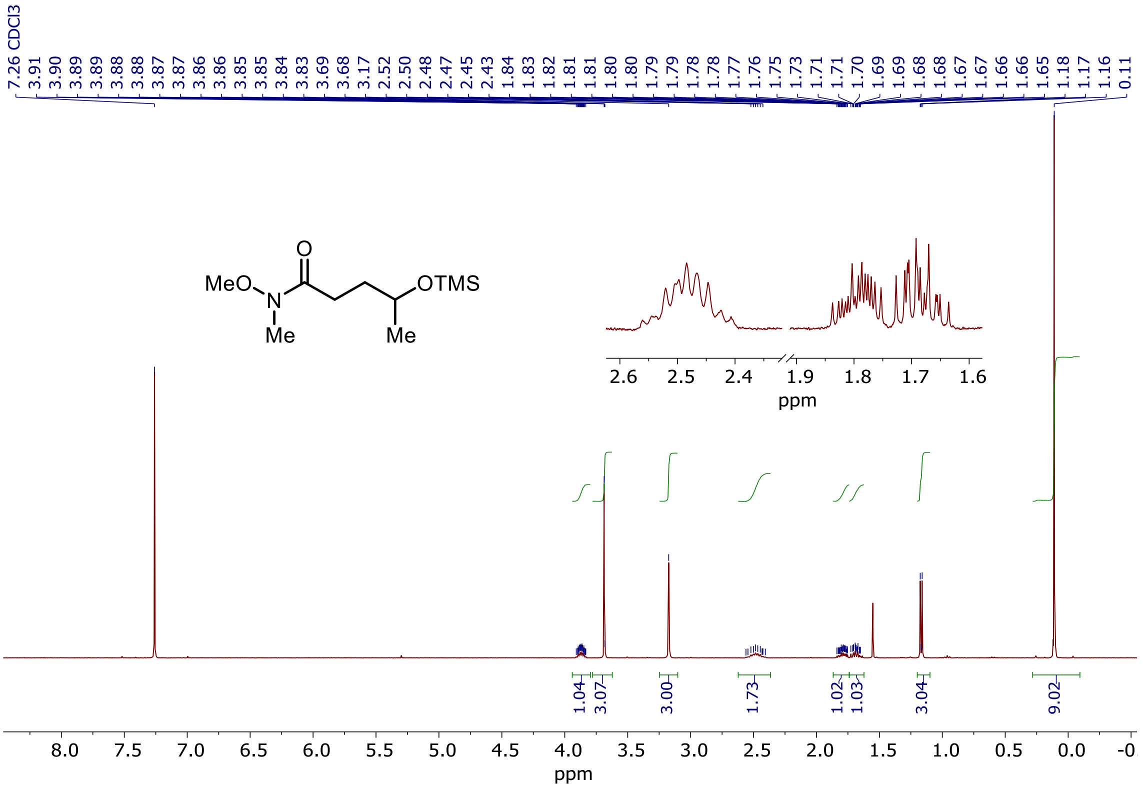

^{13}C NMR (126 MHz, $CDCl_3$) of **N-Methoxy-N-methyl-4-((trimethylsilyl)oxy)pentanamide: 2r**

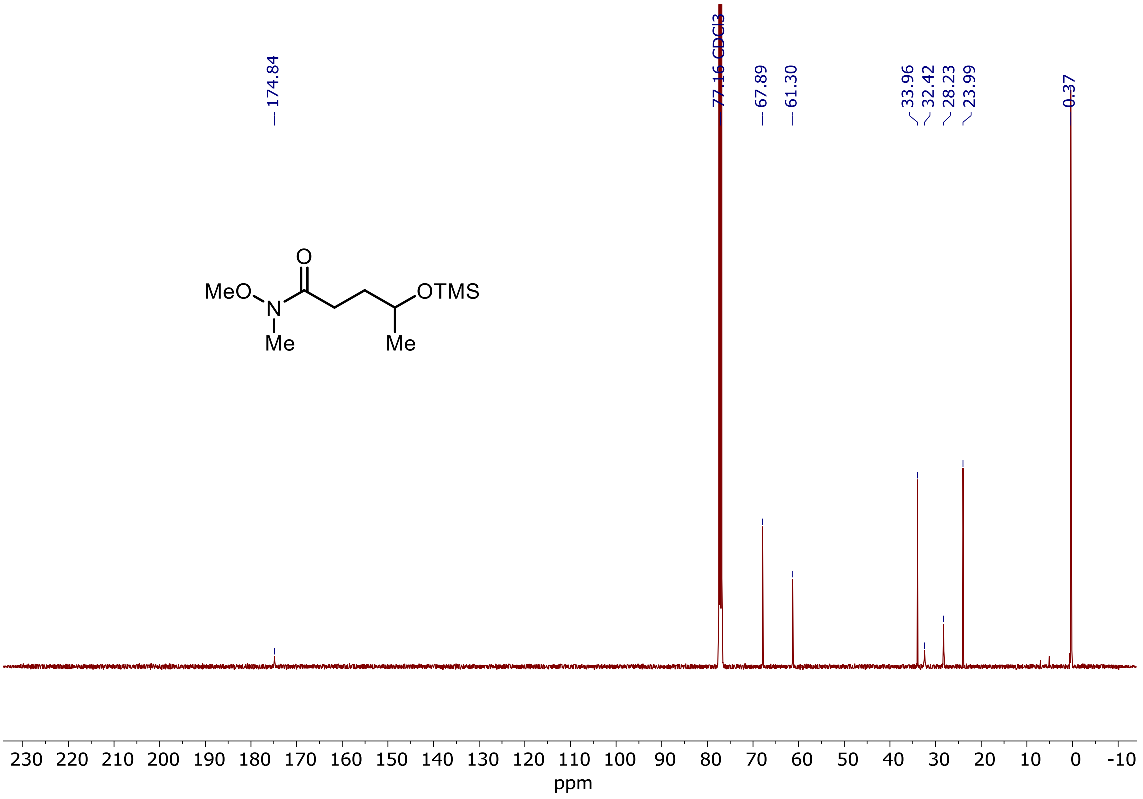

^{1}H NMR (400 MHz, $CDCl_3$) of **N-Methoxy-N-methyl-5-((trimethylsilyl)oxy)pentanamide: 2s** (*see procedure*)

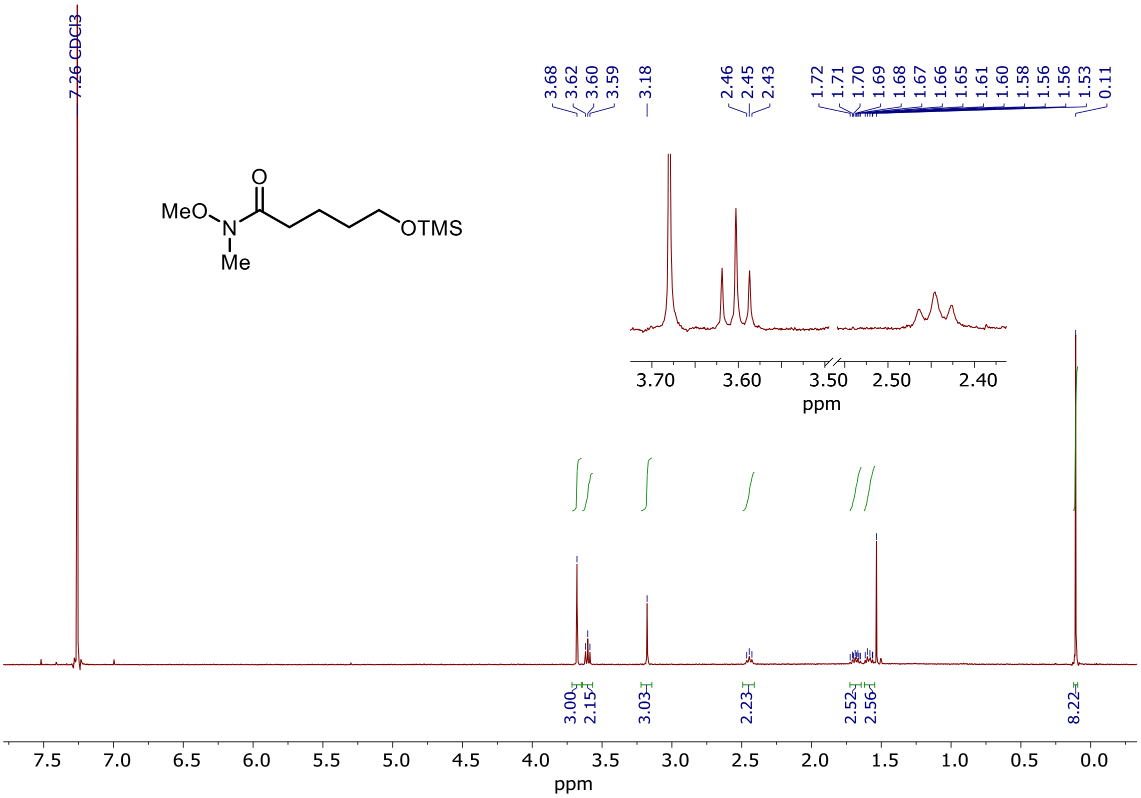

^{13}C NMR (126 MHz, $CDCl_3$) of **N-Methoxy-N-methyl-5-((trimethylsilyl)oxy)pentanamide: 2s**

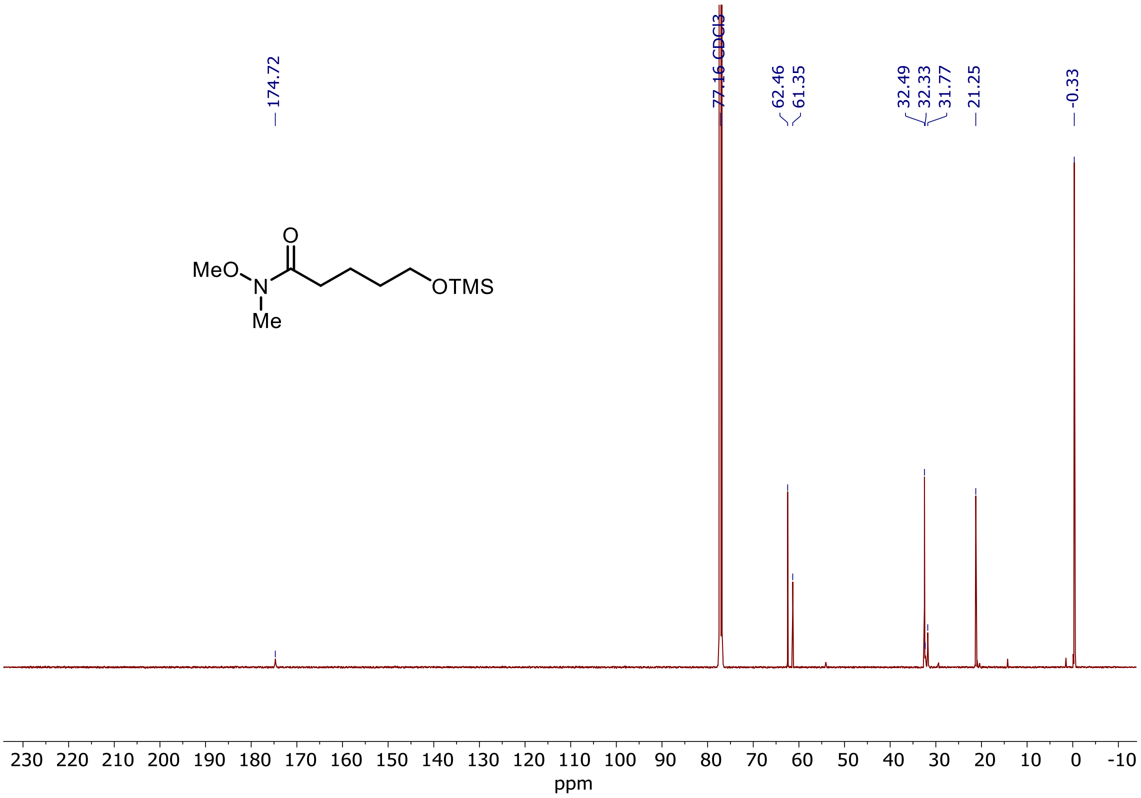

^{1}H NMR (400 MHz, $CDCl_3$) of **Ethyl 2-((*tert*-butyldimethylsilyl)oxy)benzoate: 2t** (see procedure)

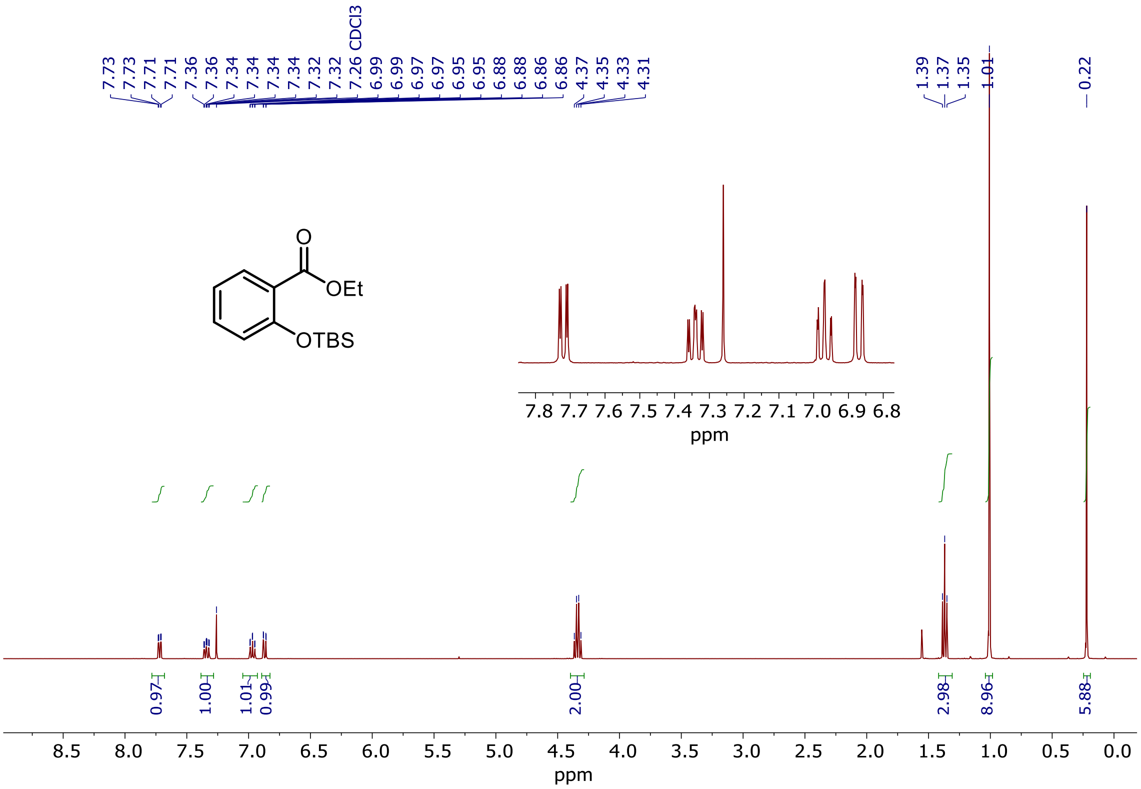

^{13}C NMR (126 MHz, $CDCl_3$) of **Ethyl 2-((*tert*-butyldimethylsilyl)oxy)benzoate: 2t**

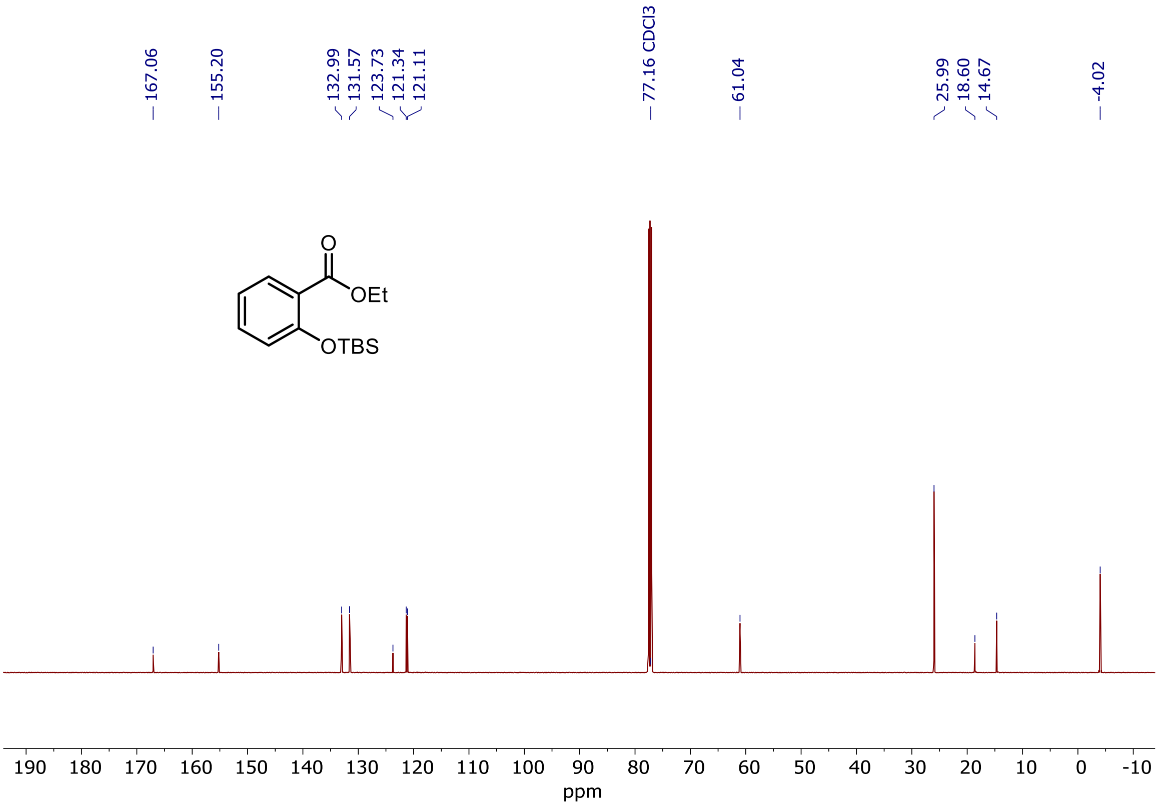

[1]**H NMR** (400 MHz, $CDCl_3$) of **Methyl 3-((*tert*-butyldimethylsilyl)oxy)picolinate: 2u**

(*see procedure*)

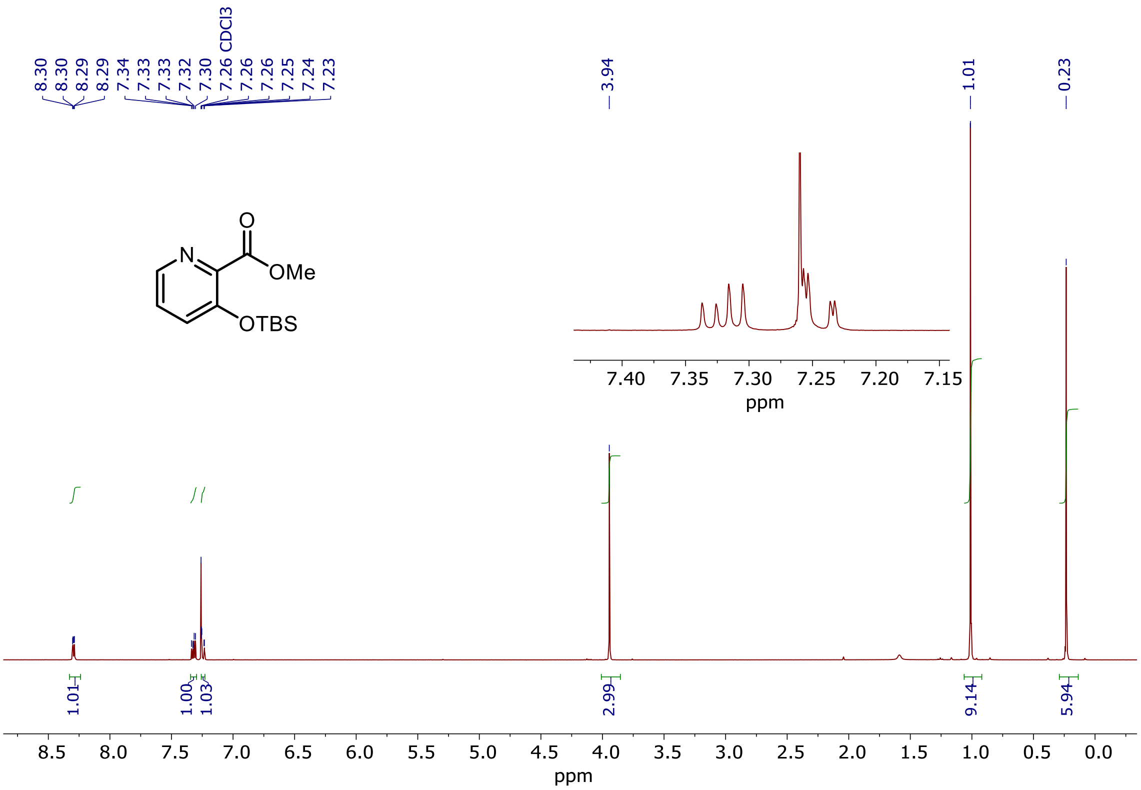

[13]**C NMR** (126 MHz, $CDCl_3$) of **Methyl 3-((*tert*-butyldimethylsilyl)oxy)picolinate: 2u**

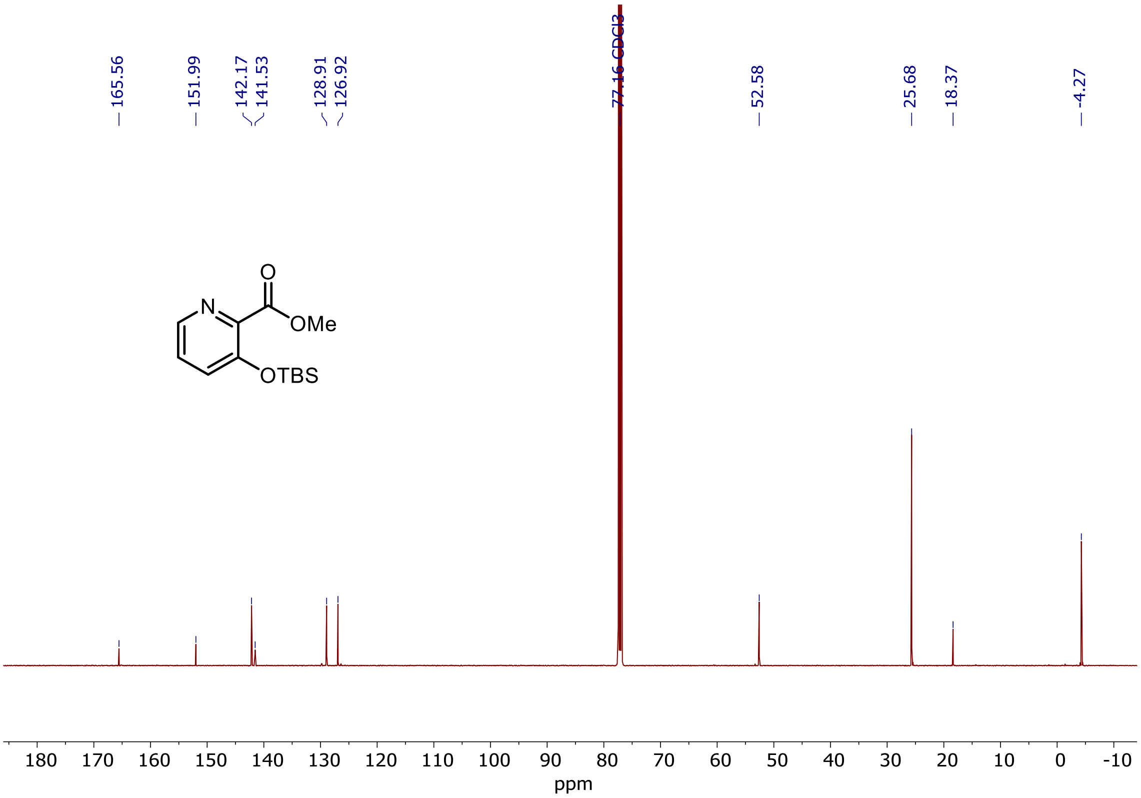

^{1}H NMR (400 MHz, $CDCl_3$) of **3-((*tert*-Butyldimethylsilyl)oxy)picolinaldehyde: 2u**′ (see procedure)

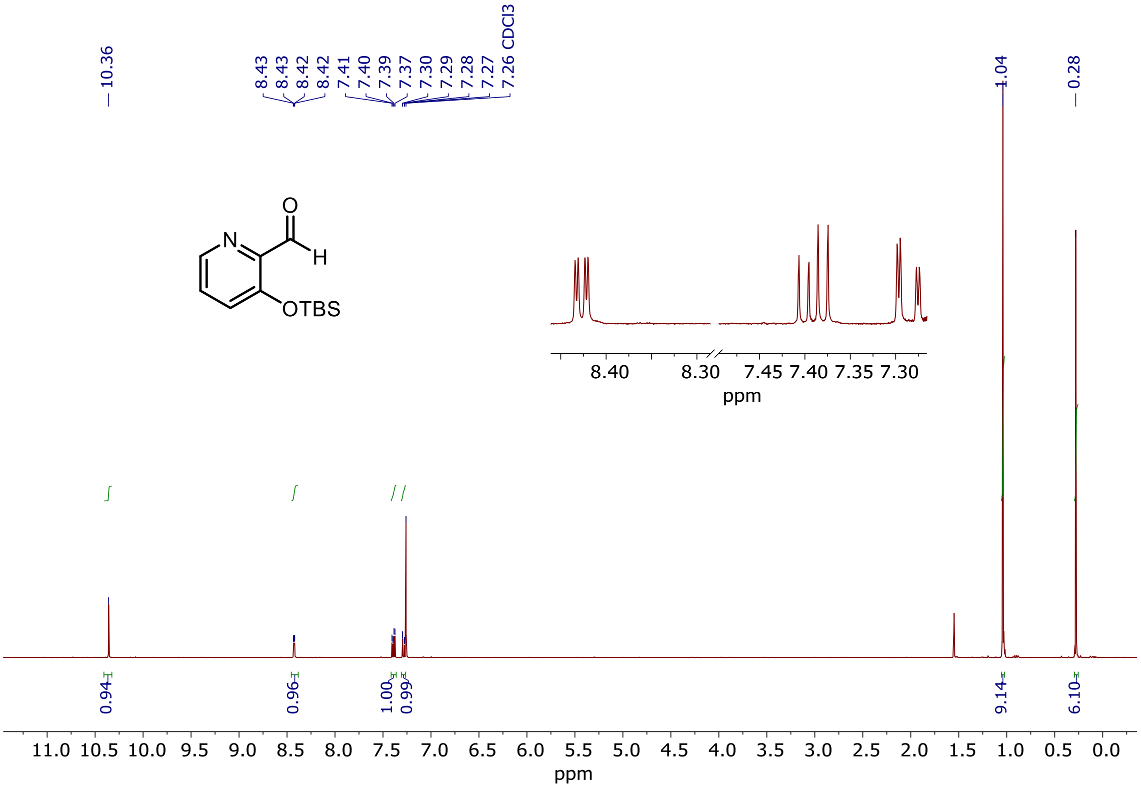

^{13}C NMR (126 MHz, $CDCl_3$) of **3-((*tert*-Butyldimethylsilyl)oxy)picolinaldehyde: 2u** ′

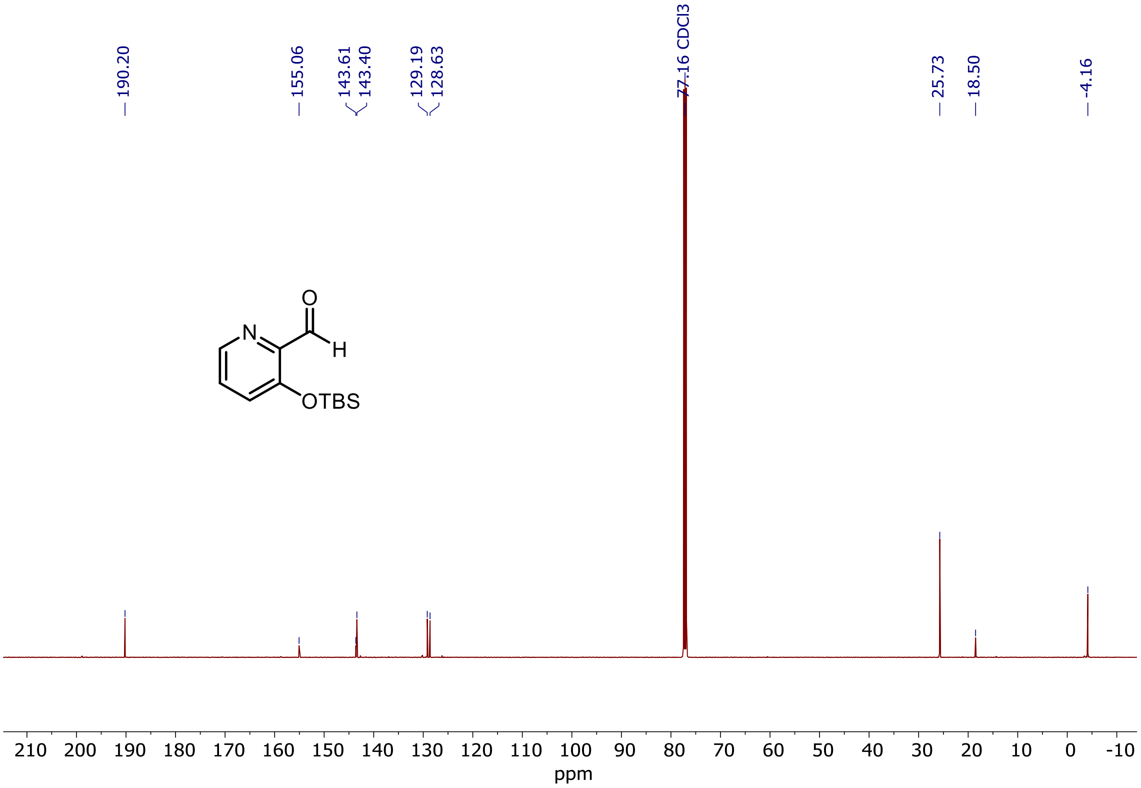

^{1}H NMR (400 MHz, $CDCl_3$) of **Methyl 3-((*tert*-butyldimethylsilyl)oxy)-2-naphthoate: 2v** (*see procedure*)

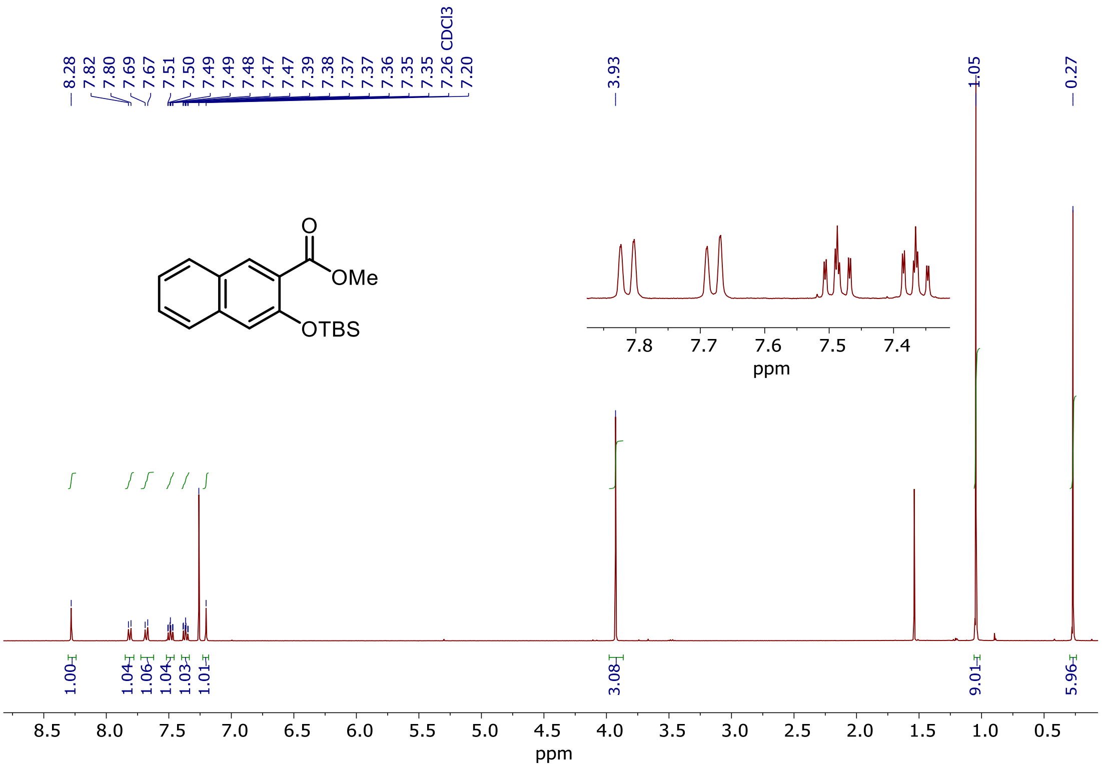

^{13}C NMR (126 MHz, $CDCl_3$) of **Methyl 3-((*tert*-butyldimethylsilyl)oxy)-2-naphthoate: 2v**

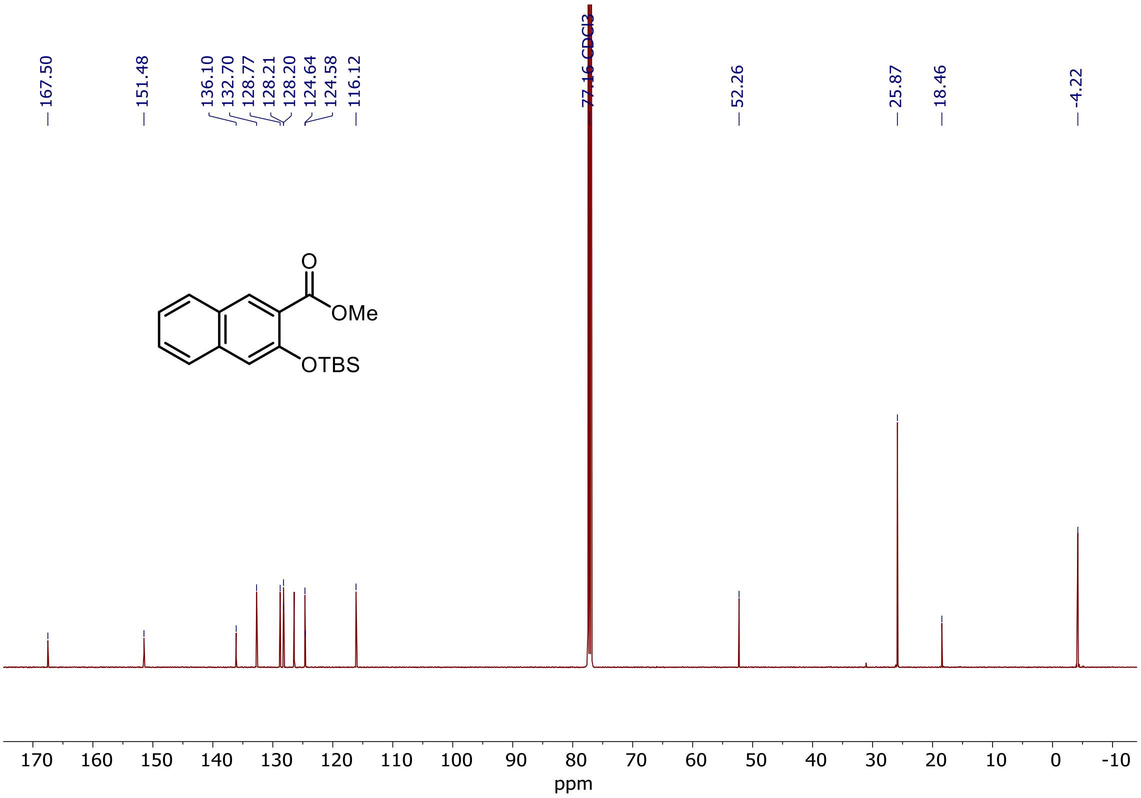

^{1}H NMR (400 MHz, CD_3OD) of **2,3-Dibromopropan-1-amine hydrobromide: 5** (*see procedure*)

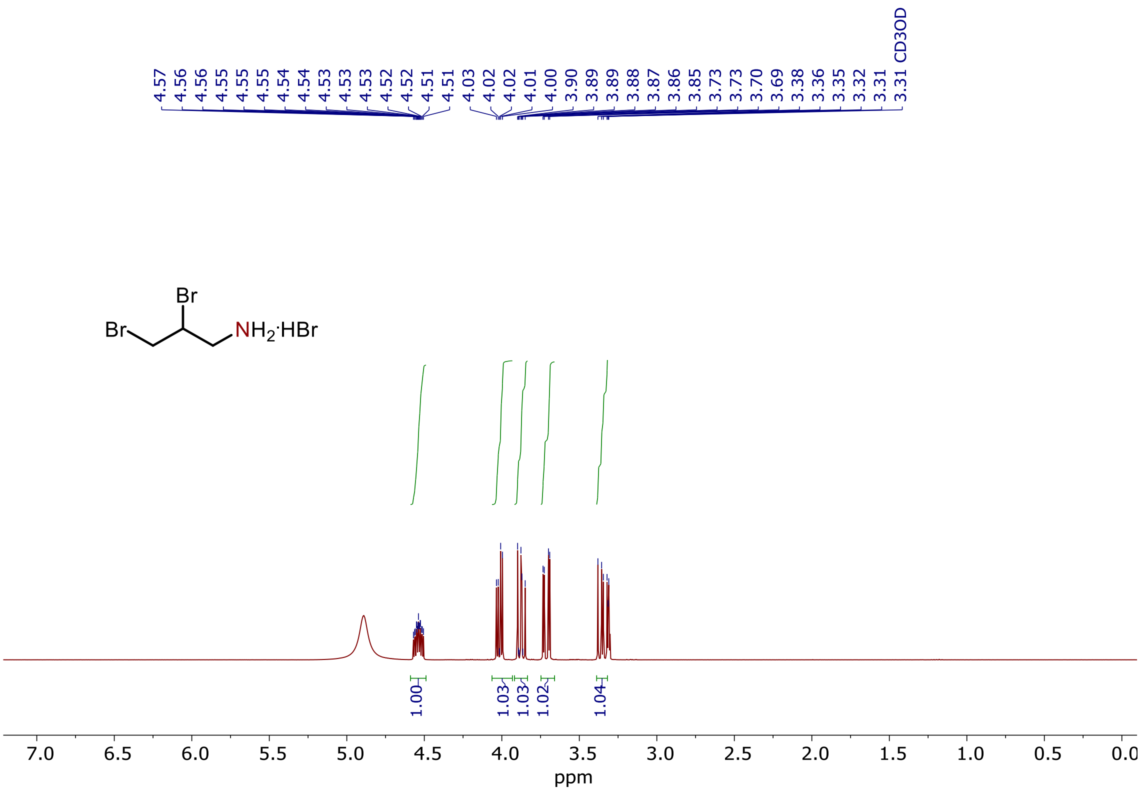

^{13}C NMR (101 MHz, CD_3OD) of **2,3-Dibromopropan-1-amine hydrobromide: 5**

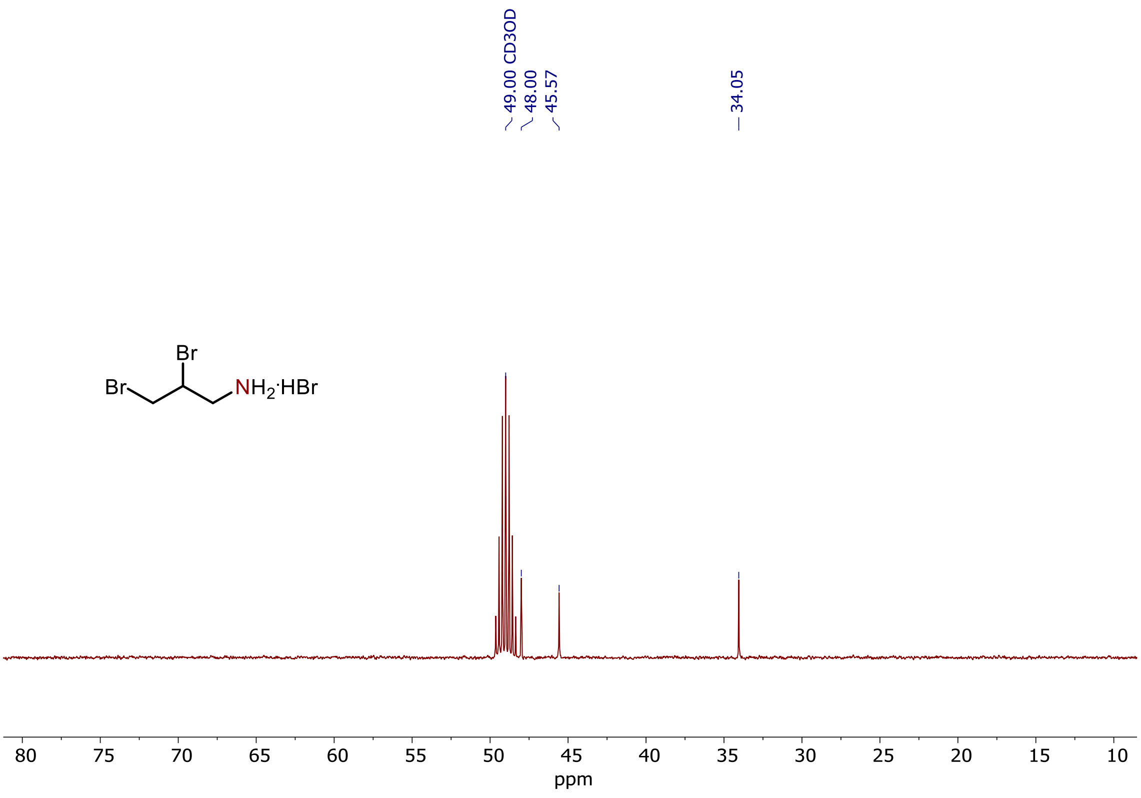

^{1}H NMR (400 MHz, $CDCl_3$) of **1-(1-Azabicyclo[1.1.0]butan-3-yl)-2-((triethylsilyl)oxy)propan-1-one: 3a** (*see procedure*)

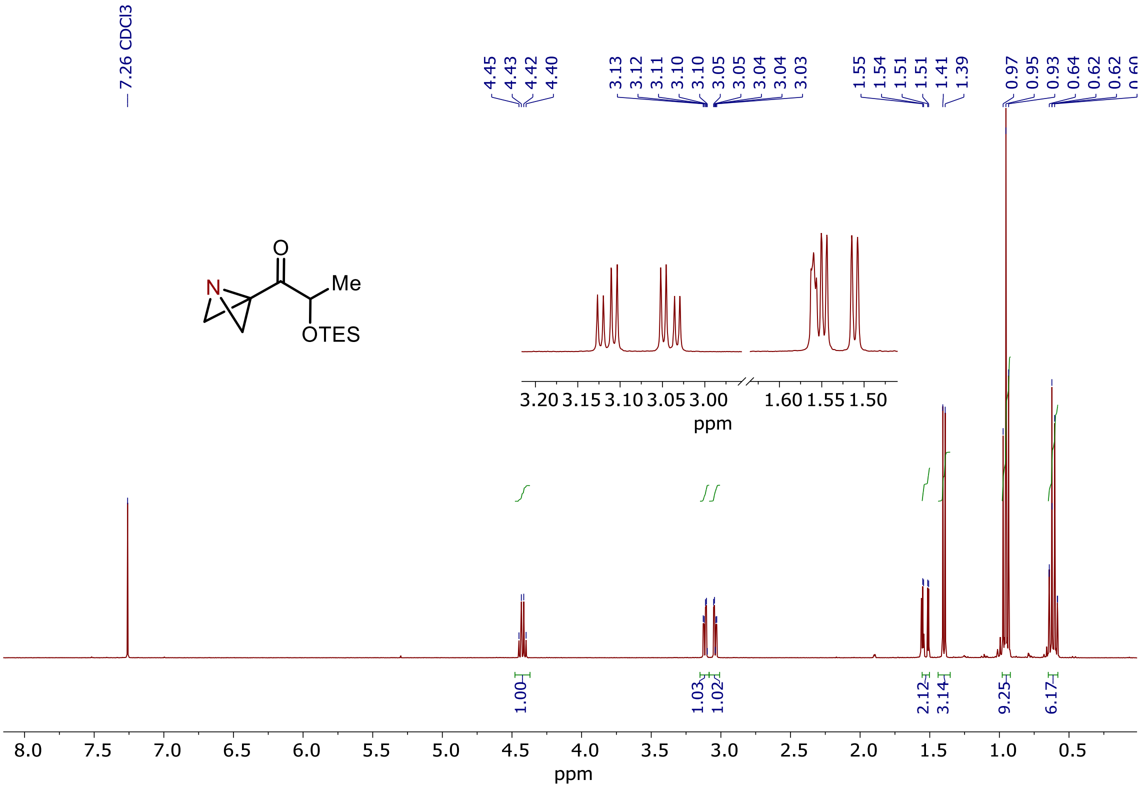

^{13}C NMR (126 MHz, $CDCl_3$) of **1-(1-Azabicyclo[1.1.0]butan-3-yl)-2-((triethylsilyl)oxy)propan-1-one: 3a**

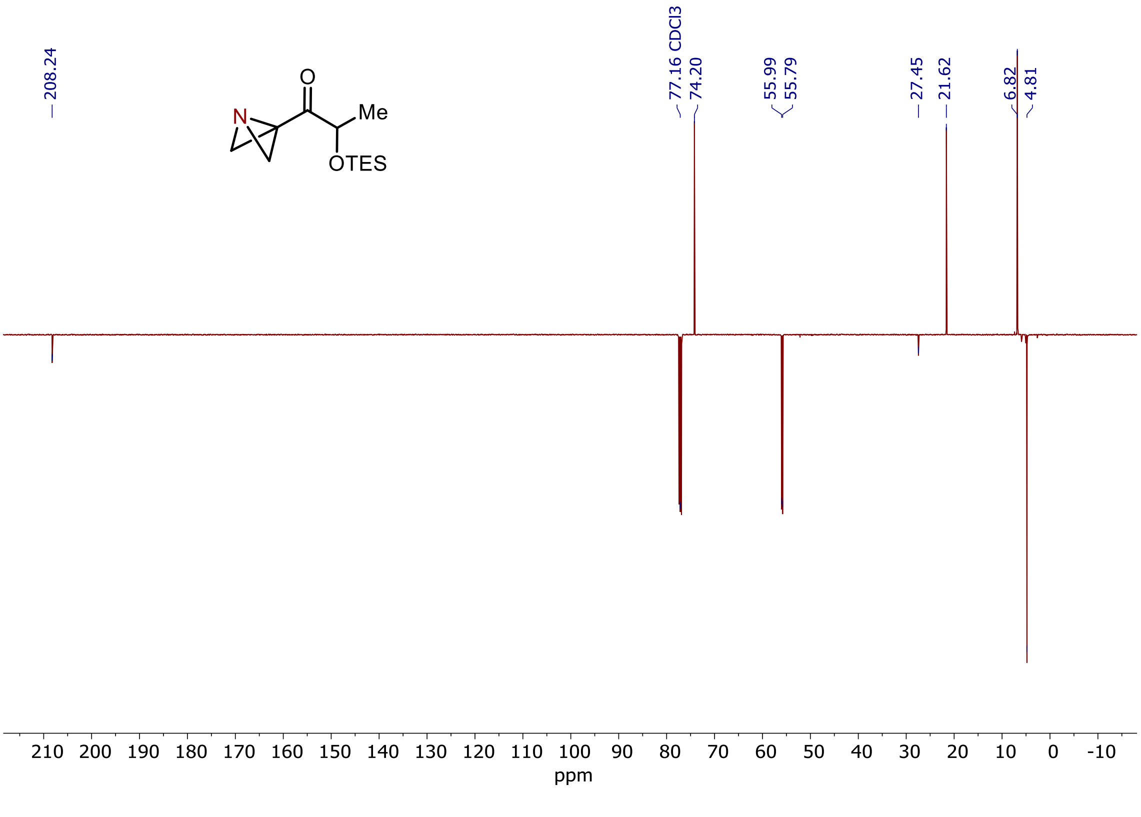

^{1}H NMR (400 MHz, $CDCl_3$) of **1-(1-Azabicyclo[1.1.0]butan-3-yl)-3-phenyl-2-((triethylsilyl)oxy)propan-1-one: 3b** (*see procedure*)

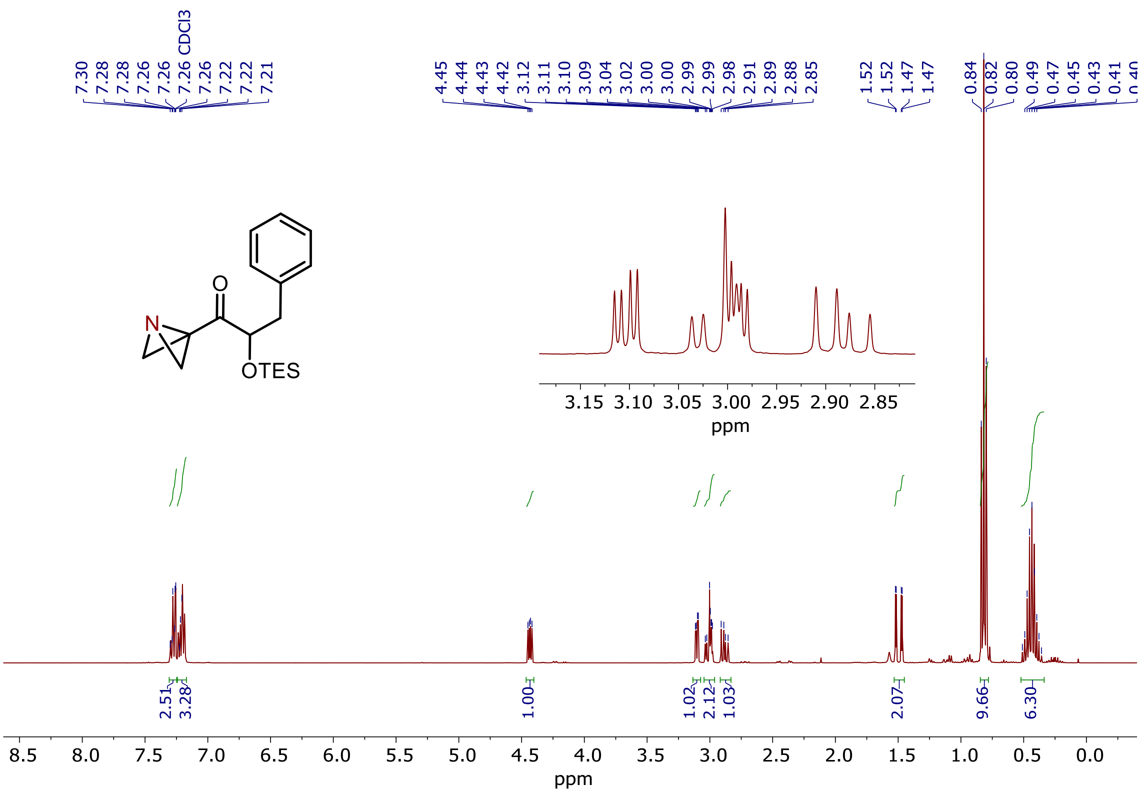

^{13}C NMR (101 MHz, $CDCl_3$) of **1-(1-Azabicyclo[1.1.0]butan-3-yl)-3-phenyl-2-((triethylsilyl)oxy)propan-1-one: 3b**

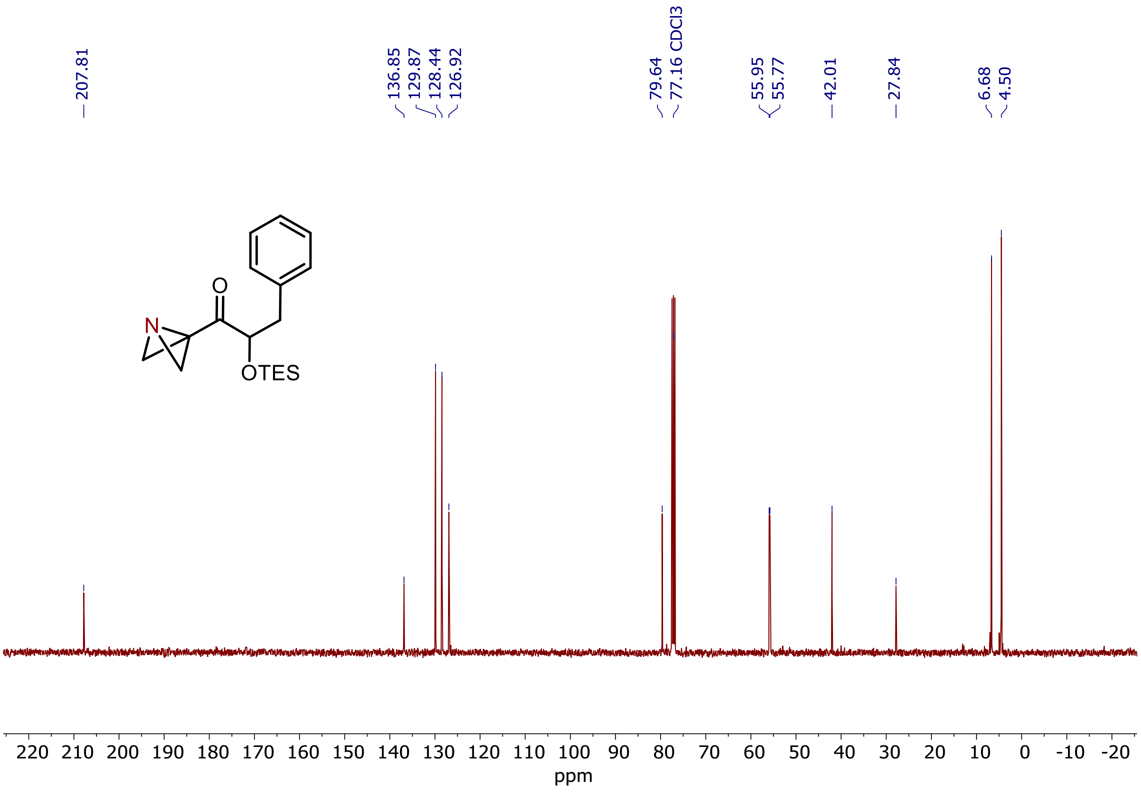

^{1}H NMR (400 MHz, $CDCl_3$) of **1-(1-Azabicyclo[1.1.0]butan-3-yl)-2-methyl-2-((triethylsilyl)oxy)propan-1-one: 3c** (*see procedure*)

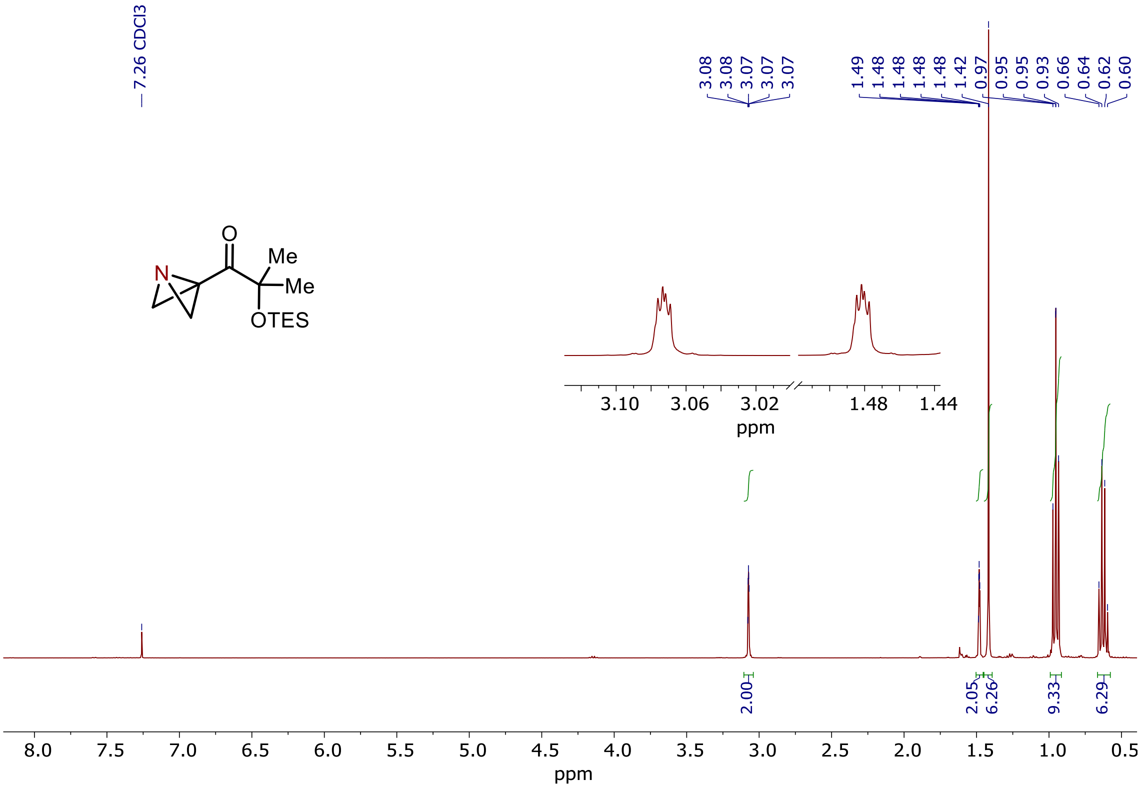

^{13}C NMR (101 MHz, $CDCl_3$) of **1-(1-Azabicyclo[1.1.0]butan-3-yl)-2-methyl-2-((triethylsilyl)oxy)propan-1-one: 3c**

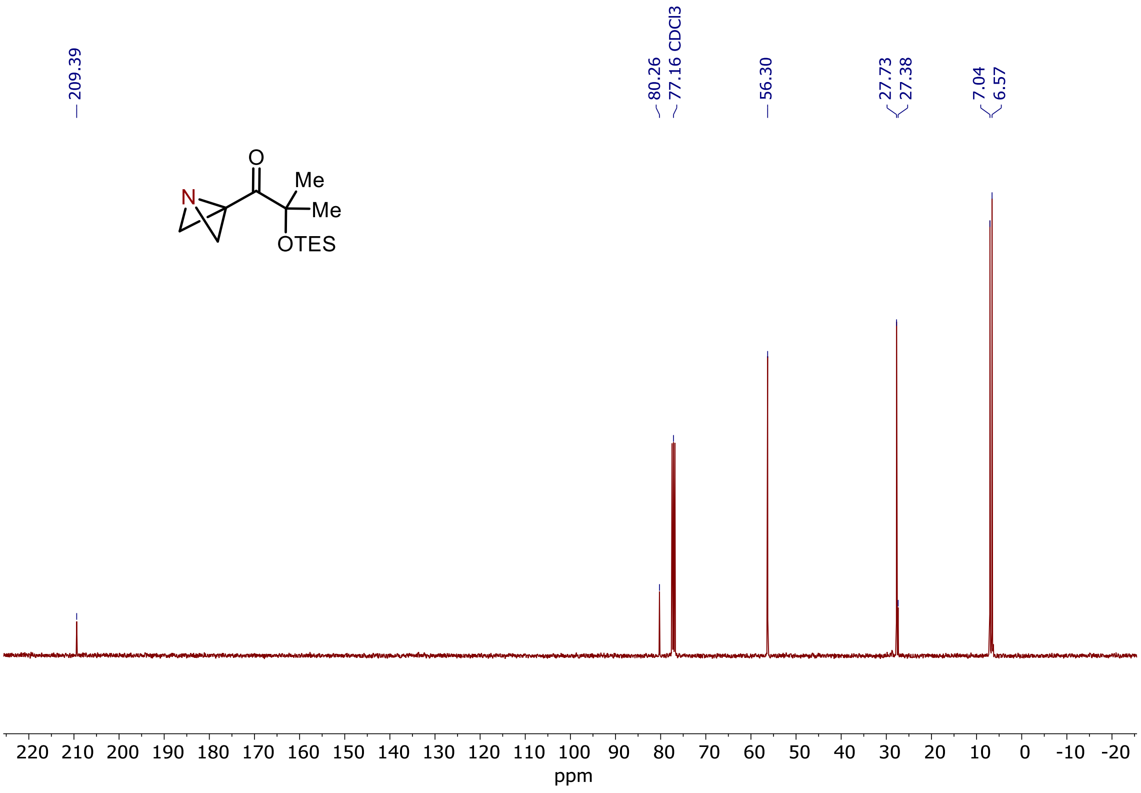

^{1}H NMR (400 MHz, $CDCl_3$) of 1-(1-Azabicyclo[1.1.0]butan-3-yl)-2-methyl-3-((triethylsilyl)oxy)propan-1-one: 3d (*see procedure*)

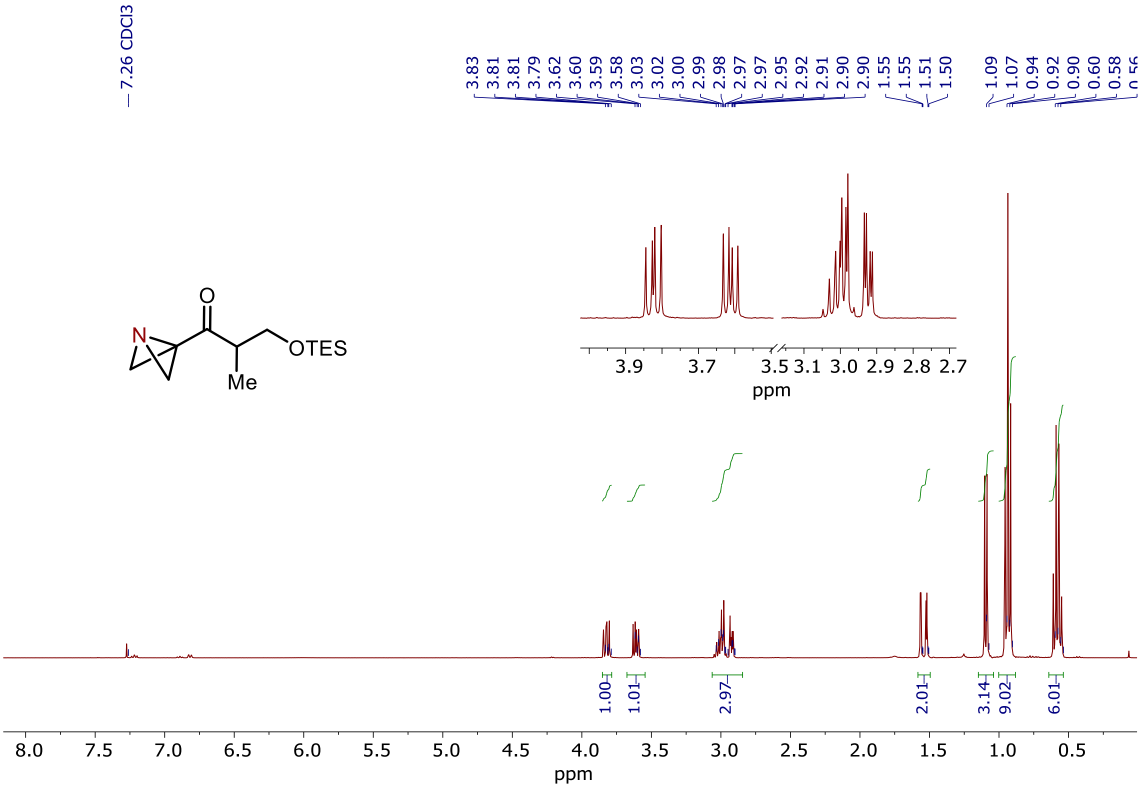

^{13}C NMR (101 MHz, $CDCl_3$) of 1-(1-Azabicyclo[1.1.0]butan-3-yl)-2-methyl-3-((triethylsilyl)oxy)propan-1-one: 3d

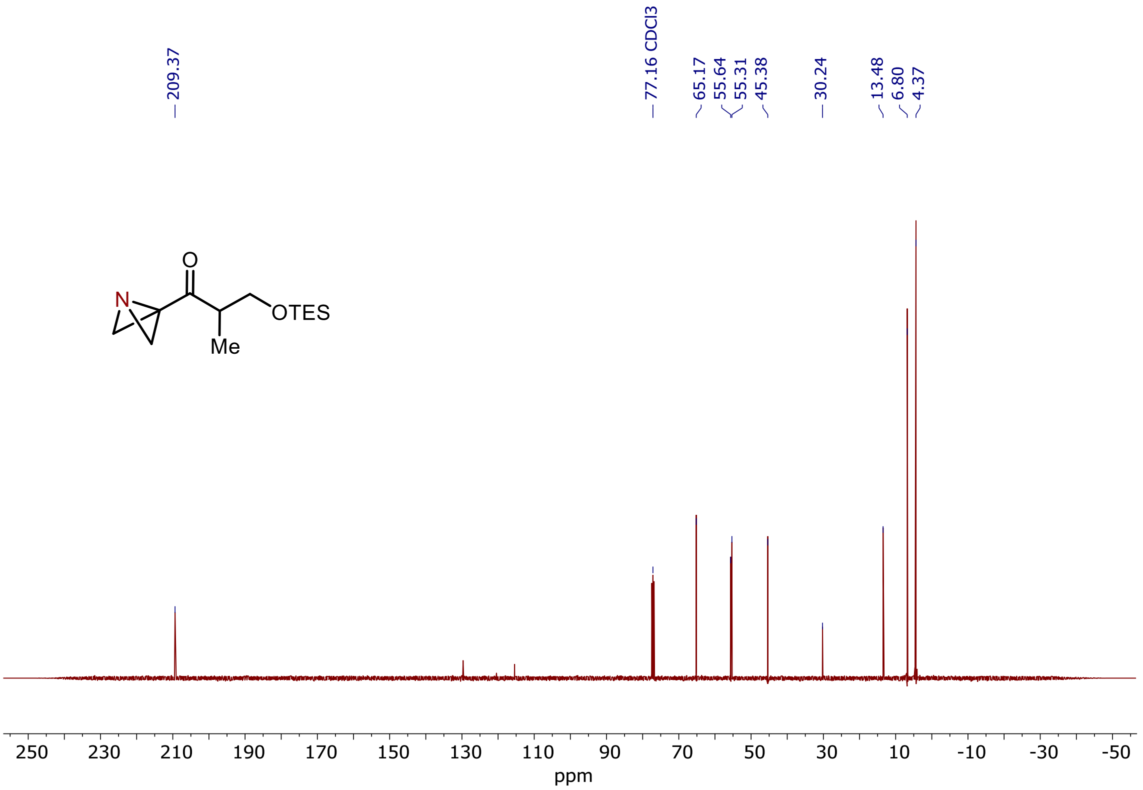

^{1}H NMR (400 MHz, $CDCl_3$) of **1-(1-Azabicyclo[1.1.0]butan-3-yl)-2-phenyl-3-((triethylsilyl)oxy)propan-1-one: 3e** (*see procedure*)

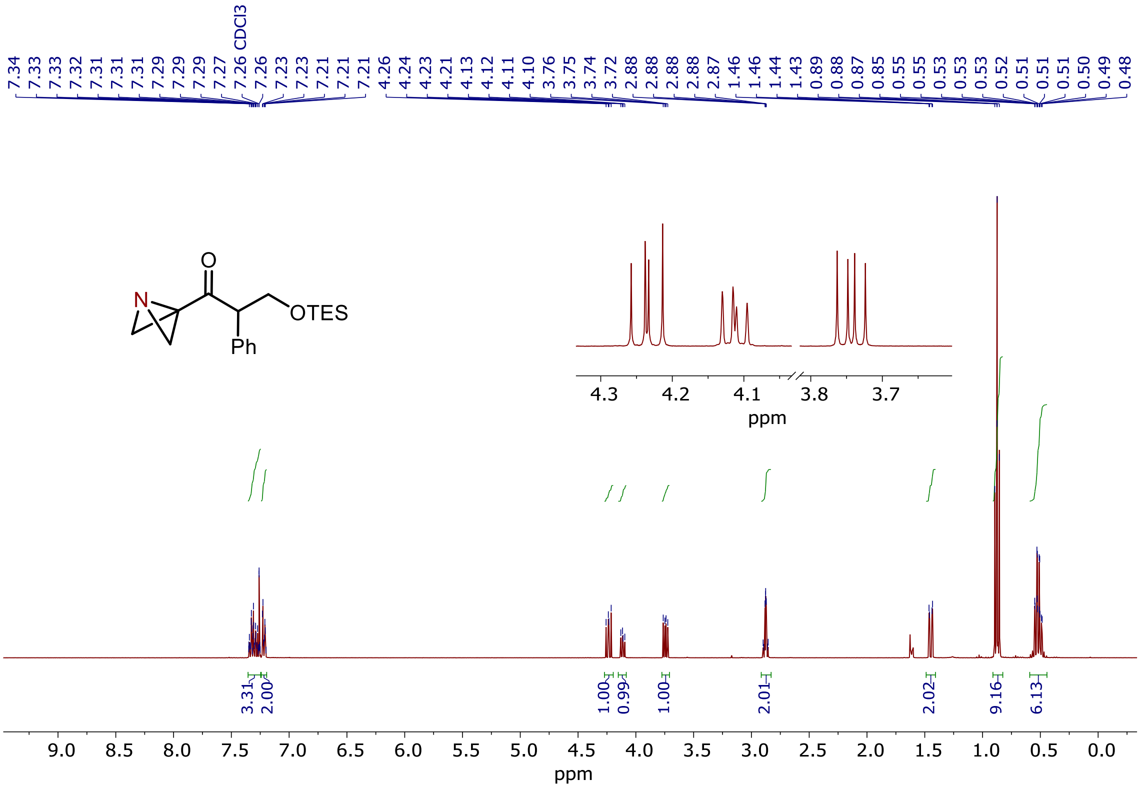

^{13}C NMR (126 MHz, $CDCl_3$) of **1-(1-Azabicyclo[1.1.0]butan-3-yl)-2-phenyl-3-((triethylsilyl)oxy)propan-1-one: 3e**

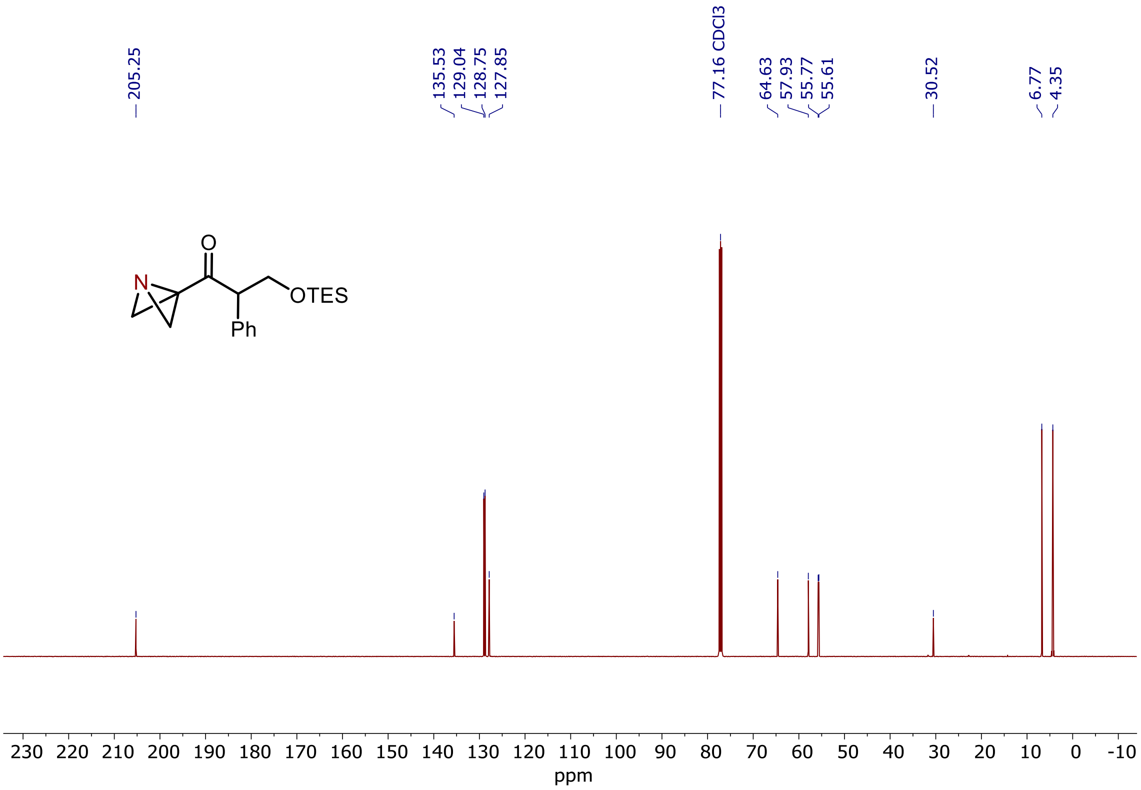

^{1}H NMR (400 MHz, $CDCl_3$) of **1-(1-Azabicyclo[1.1.0]butan-3-yl)-2,2-dimethyl-3-((triethylsilyl)oxy)propan-1-one 3f** (*see procedure*)

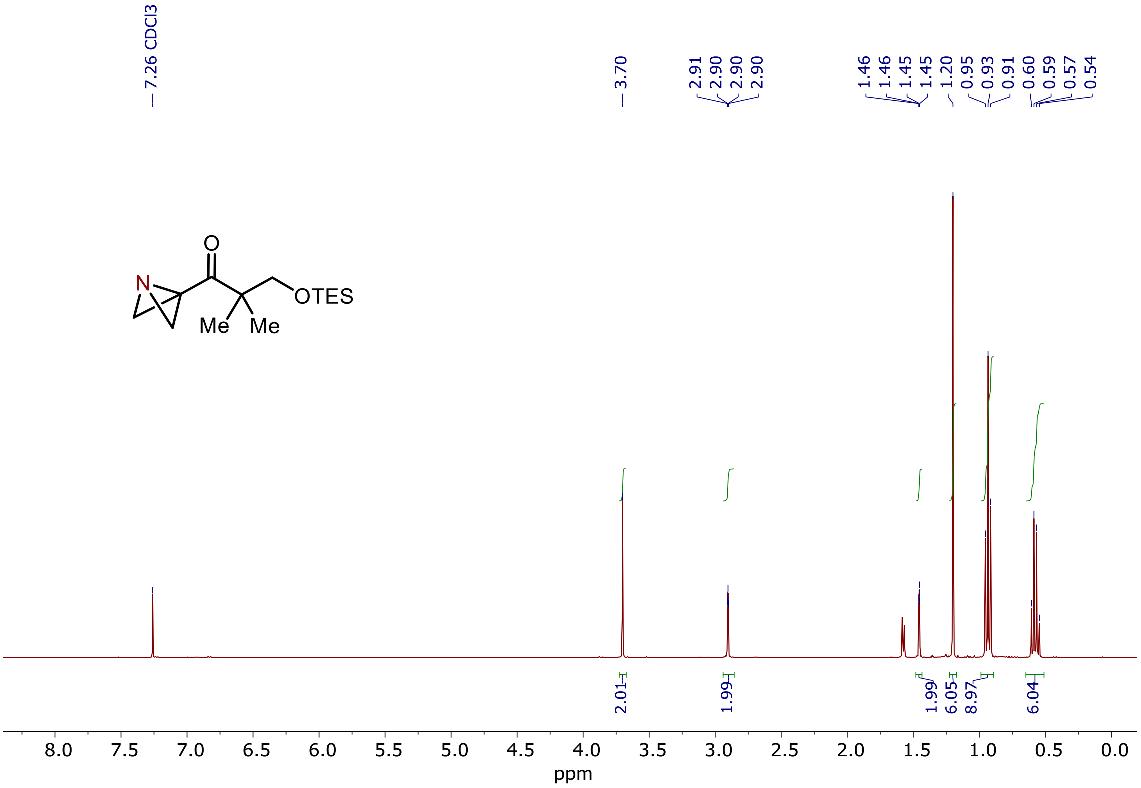

^{13}C NMR (126 MHz, $CDCl_3$) of **1-(1-Azabicyclo[1.1.0]butan-3-yl)-2,2-dimethyl-3-((triethylsilyl)oxy)propan-1-one 3f**

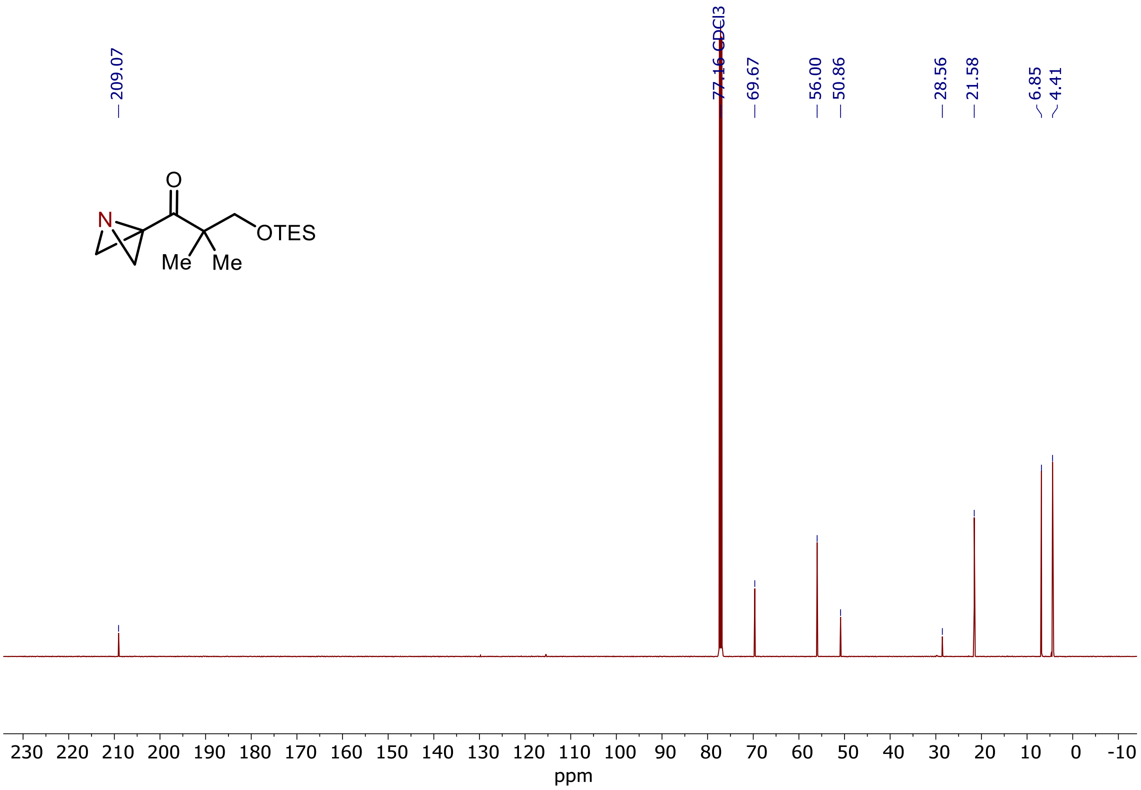

[1]**H NMR** (400 MHz, $CDCl_3$) of **1-(1-Azabicyclo[1.1.0]butan-3-yl)-3-((trimethylsilyl)oxy)butan-1-one: 3g** (*see procedure*)

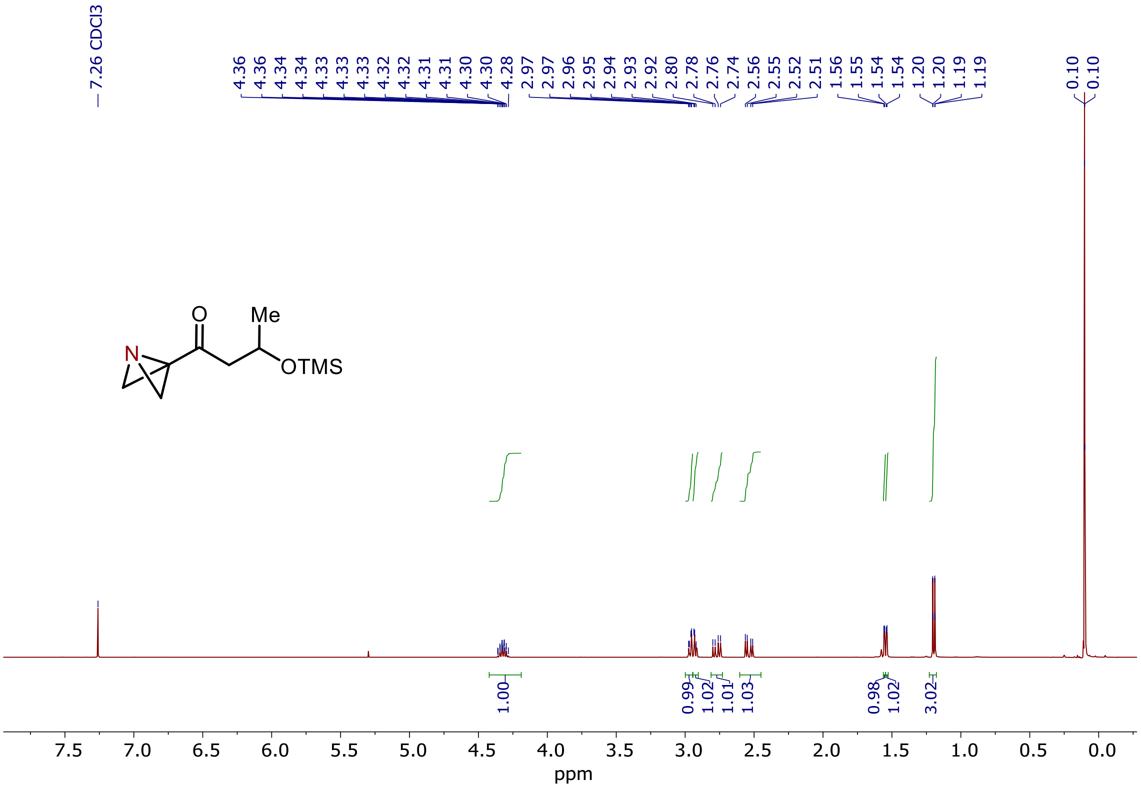

[13]**C NMR** (126 MHz, $CDCl_3$) of **1-(1-Azabicyclo[1.1.0]butan-3-yl)-3-((trimethylsilyl)oxy)butan-1-one: 3g**

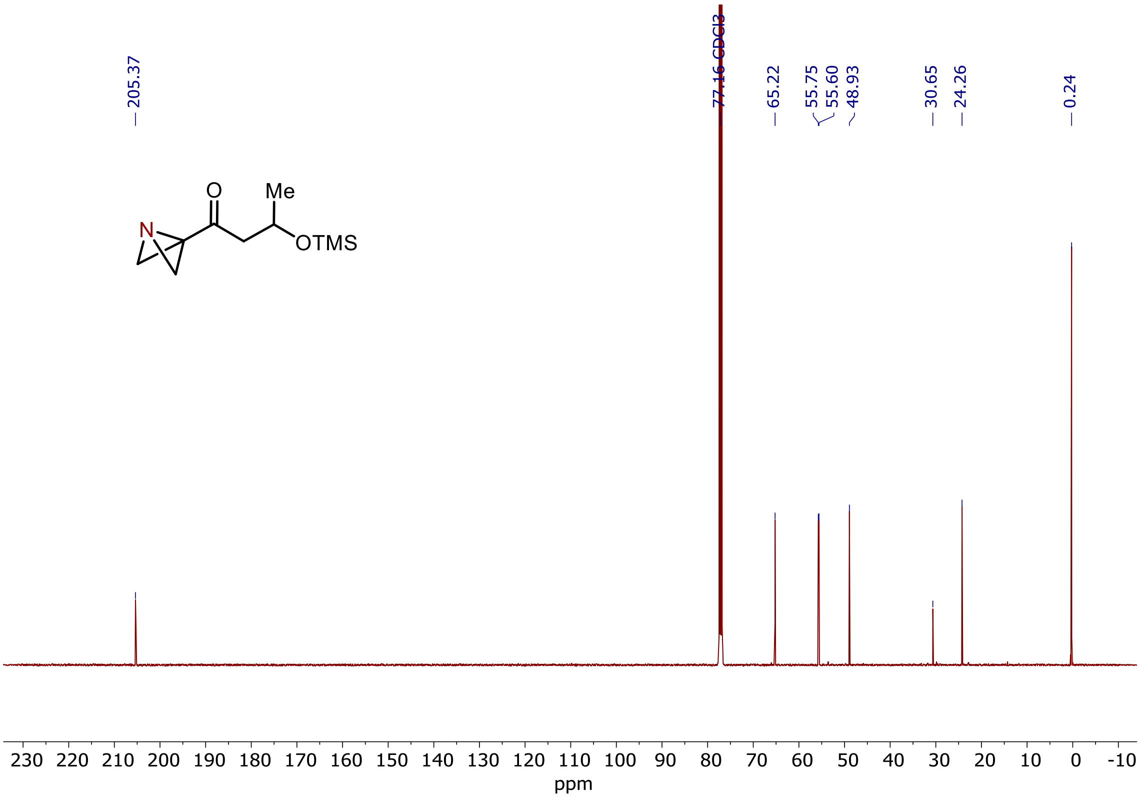

^{1}H NMR (400 MHz, $CDCl_3$) of **1-(1-Azabicyclo[1.1.0]butan-3-yl)-3-cyclopropyl-3-((trimethylsilyl)oxy)propan-1-one: 3h** (*see procedure*)

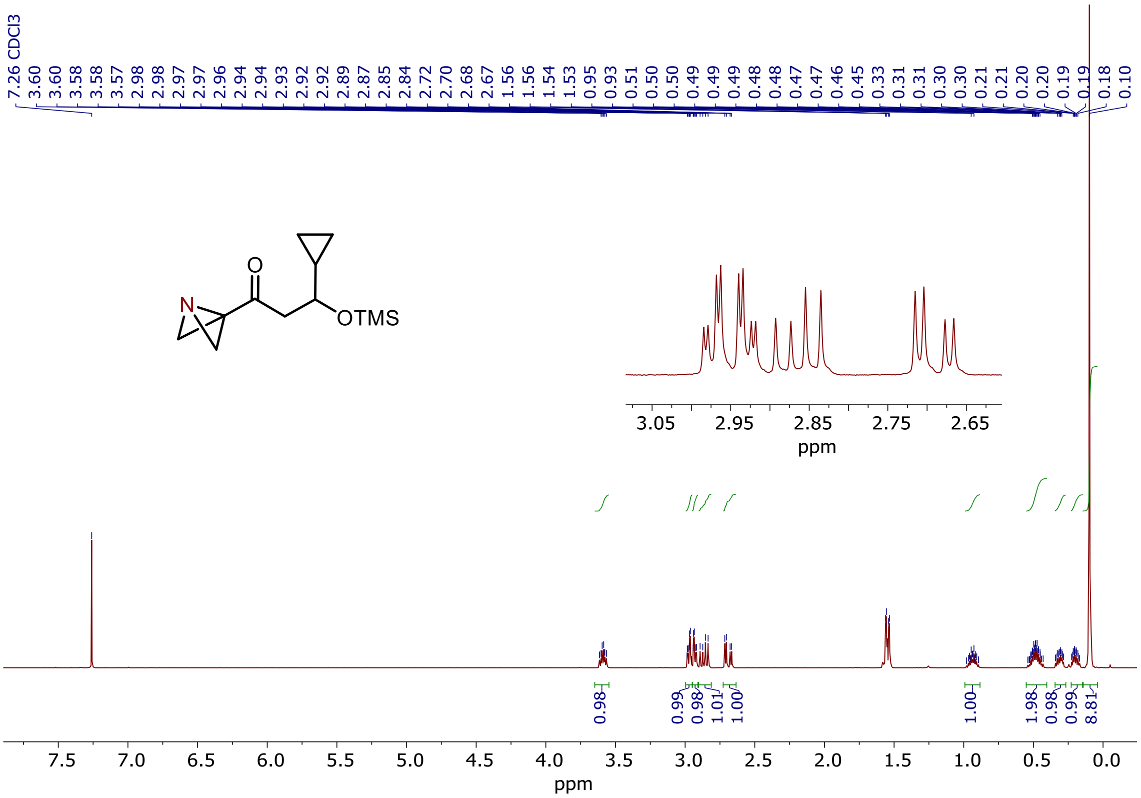

^{13}C NMR (126 MHz, $CDCl_3$) of **1-(1-Azabicyclo[1.1.0]butan-3-yl)-3-cyclopropyl-3-((trimethylsilyl)oxy)propan-1-one: 3h**

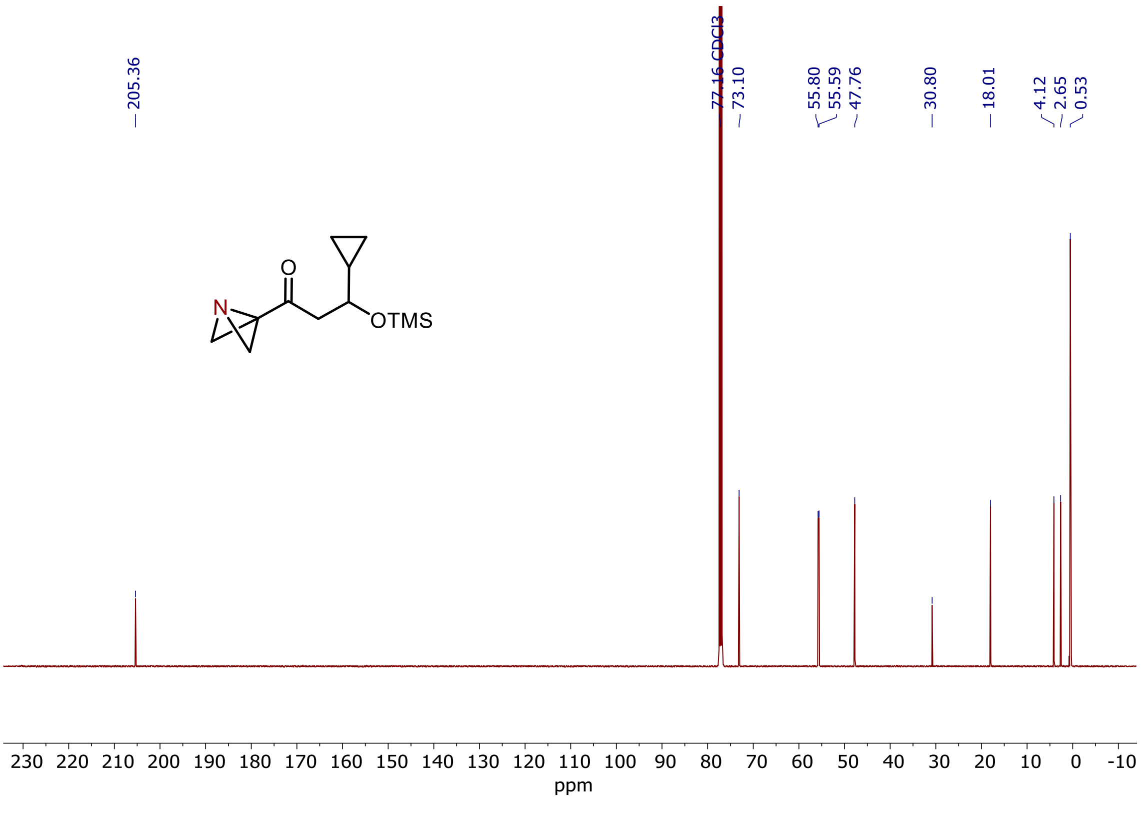

^{1}H NMR (500 MHz, $CDCl_3$) of **1-(1-Azabicyclo[1.1.0]butan-3-yl)-4-methyl-3-((trimethylsilyl)oxy)pentan-1-one: 3i** (*see procedure*)

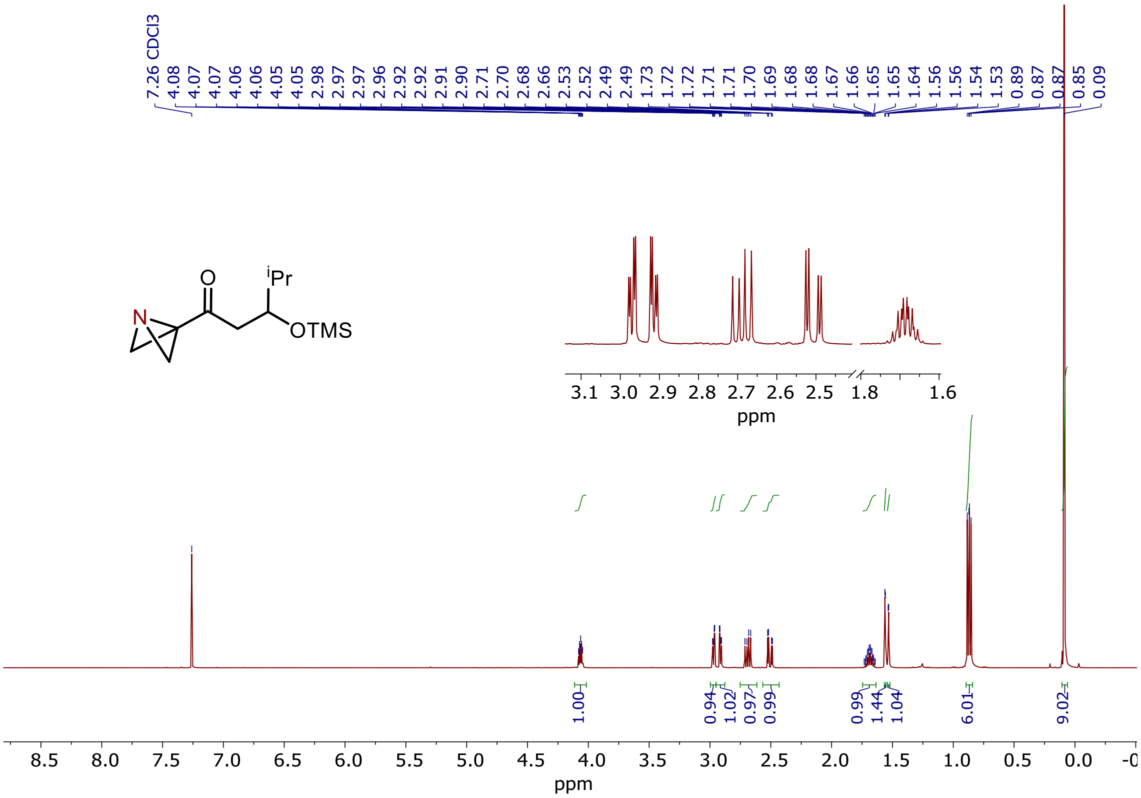

^{13}C NMR (126 MHz, $CDCl_3$) of **1-(1-Azabicyclo[1.1.0]butan-3-yl)-4-methyl-3-((trimethylsilyl)oxy)pentan-1-one: 3i**

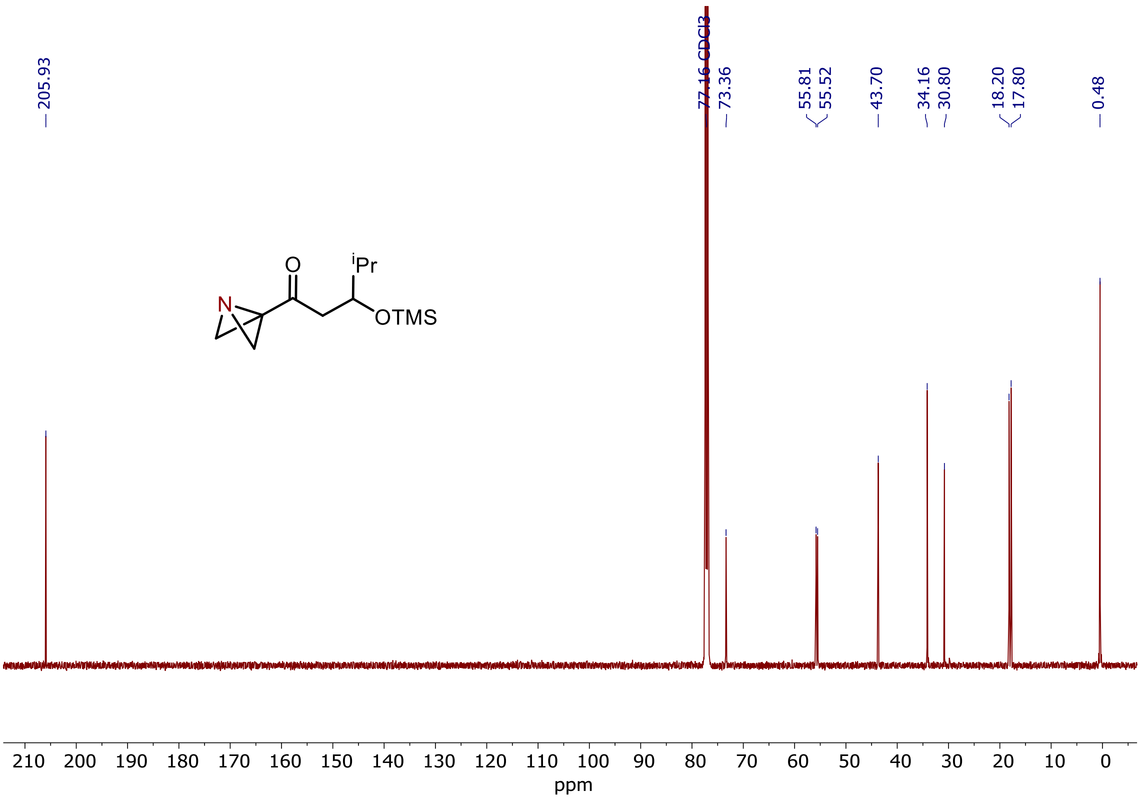

^{1}H NMR (500 MHz, $CDCl_3$) of 1-(1-Azabicyclo[1.1.0]butan-3-yl)-3-phenyl-3-((trimethylsilyl)oxy)propan-1-one: 3j (*see procedure*)

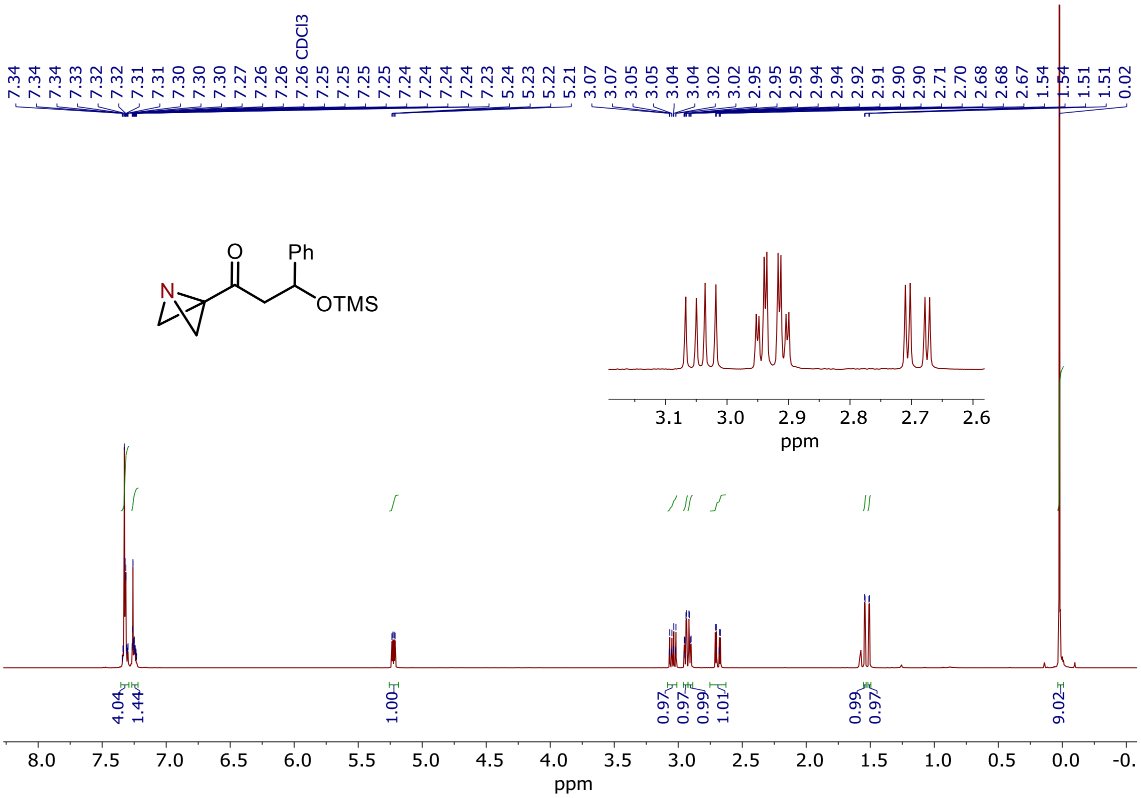

^{13}C NMR (126 MHz, $CDCl_3$) of 1-(1-Azabicyclo[1.1.0]butan-3-yl)-3-phenyl-3-((trimethylsilyl)oxy)propan-1-one: 3j

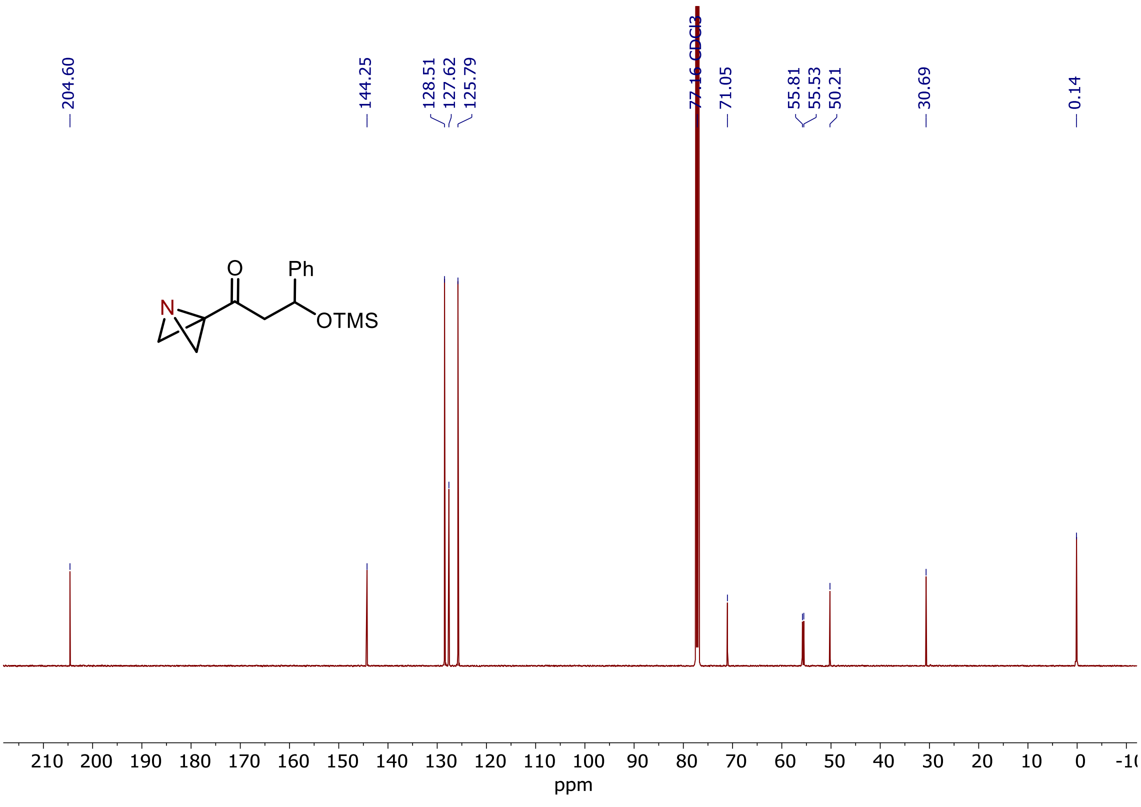

^{1}H NMR (400 MHz, $CDCl_3$) of **1-(1-Azabicyclo[1.1.0]butan-3-yl)-3-(pyridin-3-yl)-3-((trimethylsilyl)oxy)propan-1-one: 3k** (*see procedure*)

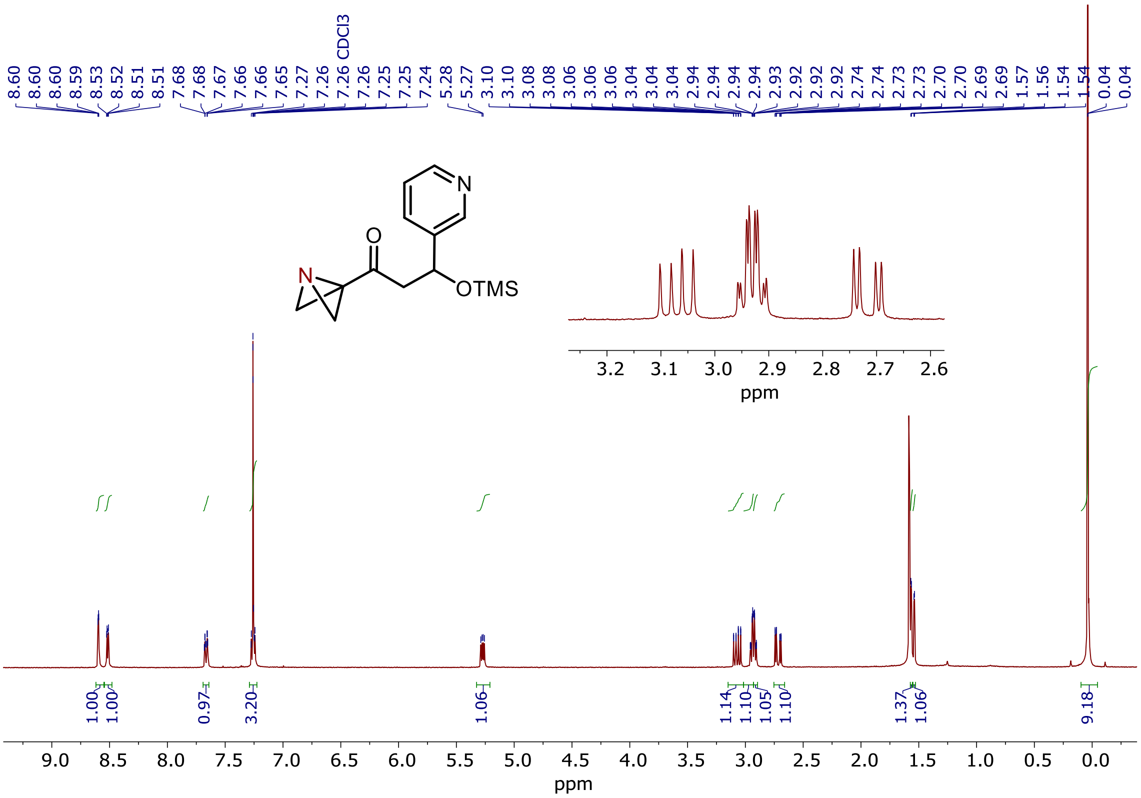

^{13}C NMR (126 MHz, $CDCl_3$) of **1-(1-Azabicyclo[1.1.0]butan-3-yl)-3-(pyridin-3-yl)-3-((trimethylsilyl)oxy)propan-1-one: 3k**

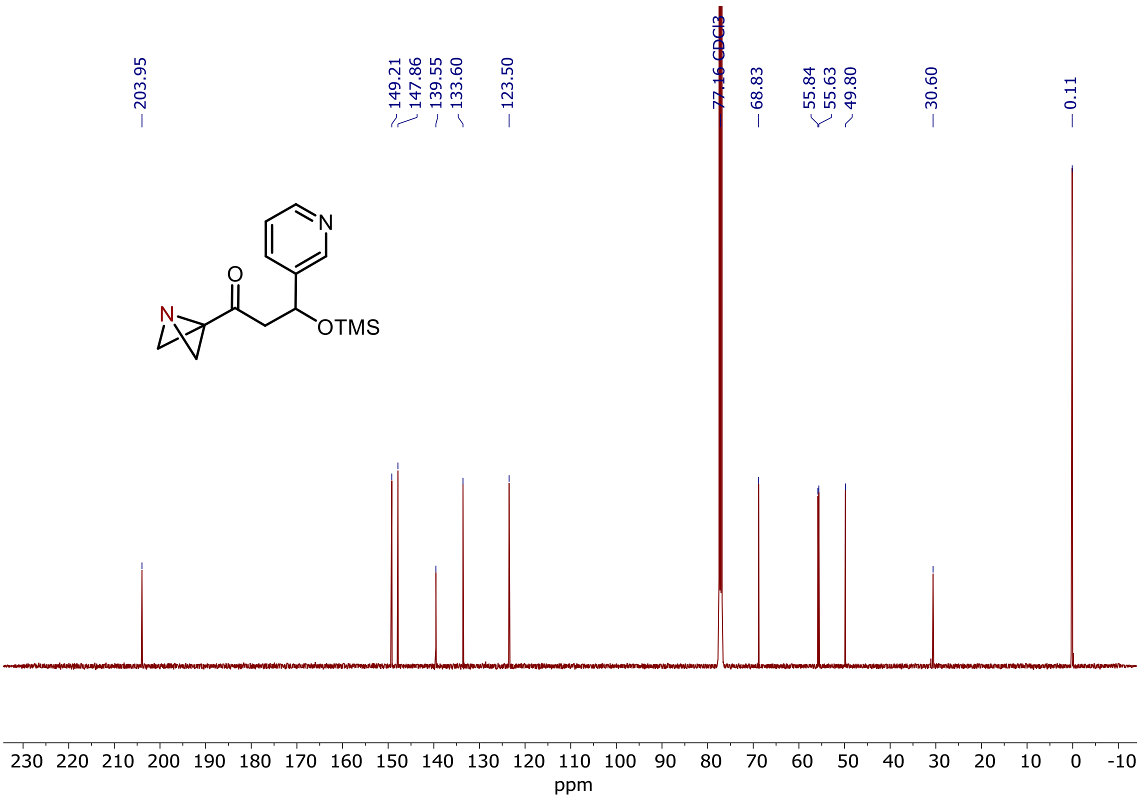

^{1}H NMR (400 MHz, $CDCl_3$) of **1-(1-Azabicyclo[1.1.0]butan-3-yl)-3-((trimethylsilyl)oxy)pent-4-en-1-one: 3l** (*see procedure*)

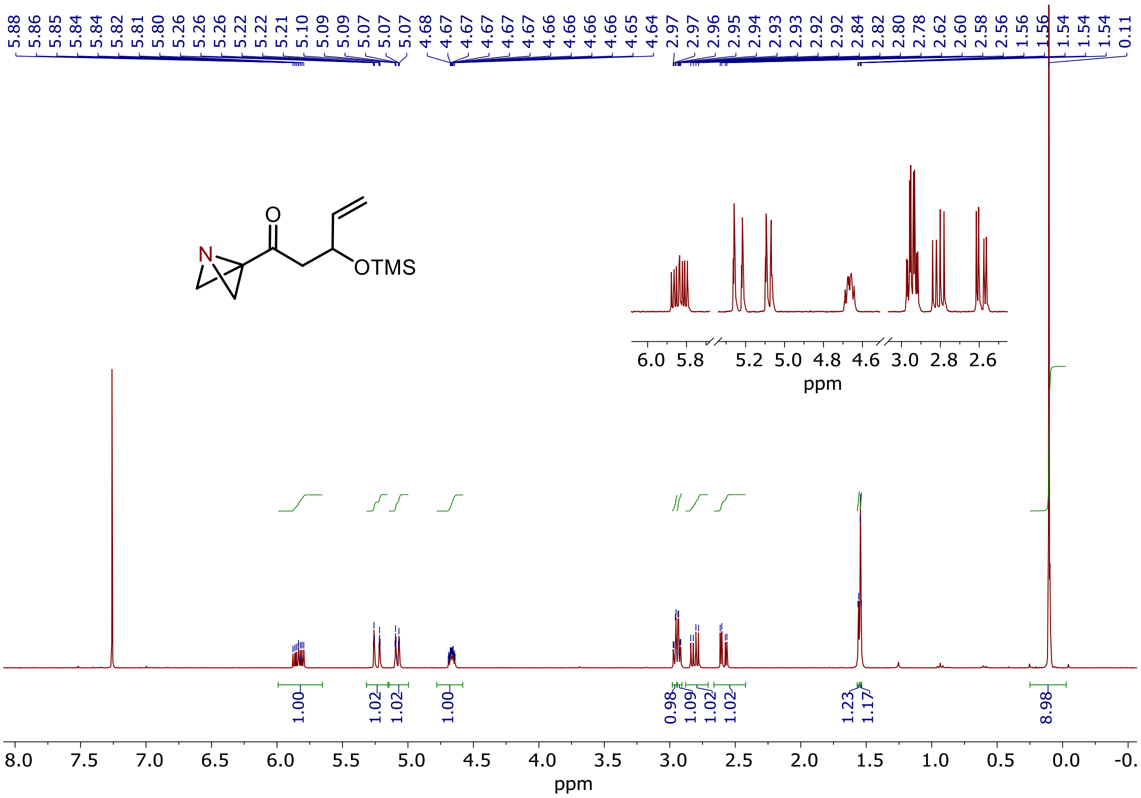

^{13}C NMR (126 MHz, $CDCl_3$) of **1-(1-Azabicyclo[1.1.0]butan-3-yl)-3-((trimethylsilyl)oxy)pent-4-en-1-one: 3l**

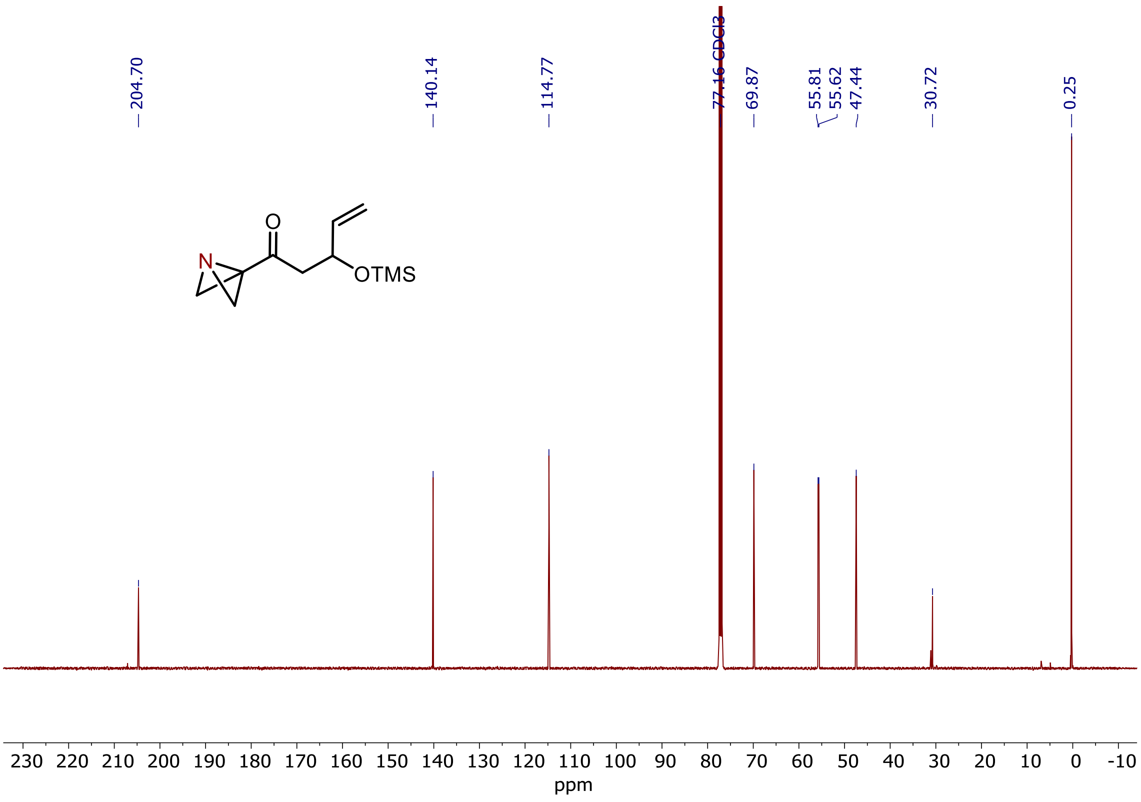

^{1}H NMR (400 MHz, $CDCl_3$) of **1-(1-Azabicyclo[1.1.0]butan-3-yl)-4-chloro-3-((trimethylsilyl)oxy)butan-1-one: 3m** (see procedure)

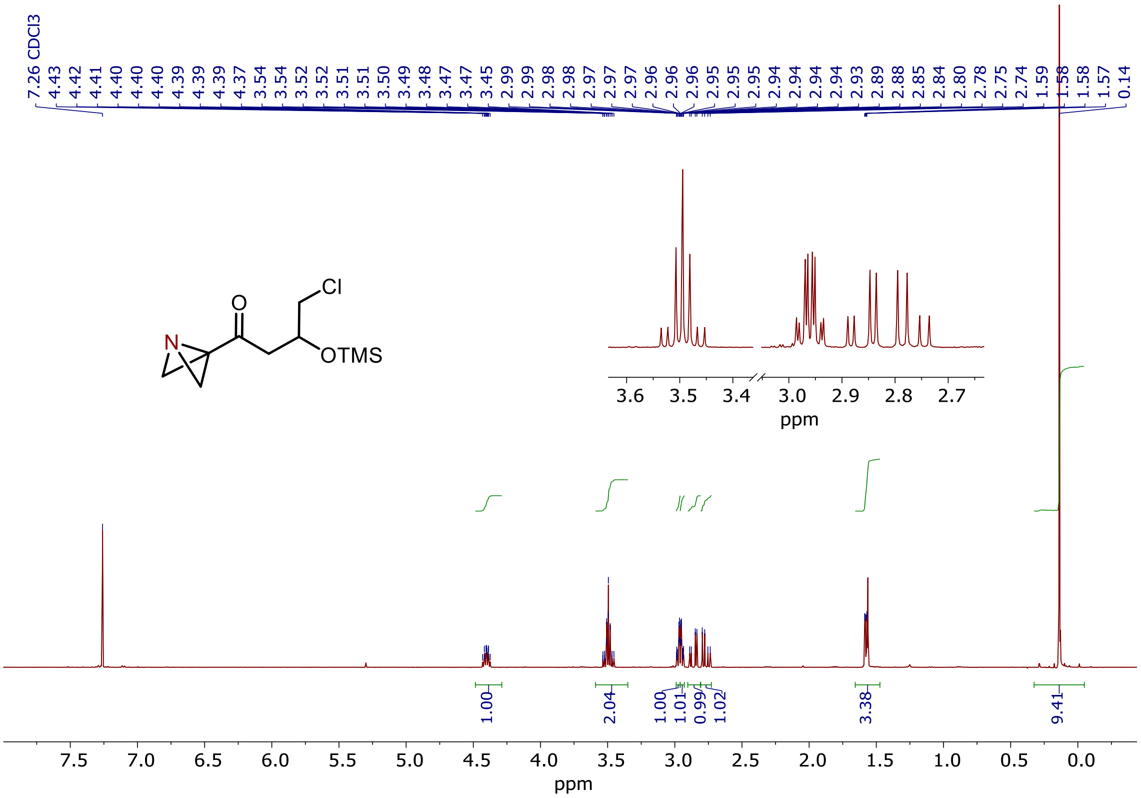

^{13}C NMR (126 MHz, $CDCl_3$) of **1-(1-Azabicyclo[1.1.0]butan-3-yl)-4-chloro-3-((trimethylsilyl)oxy)butan-1-one: 3m**

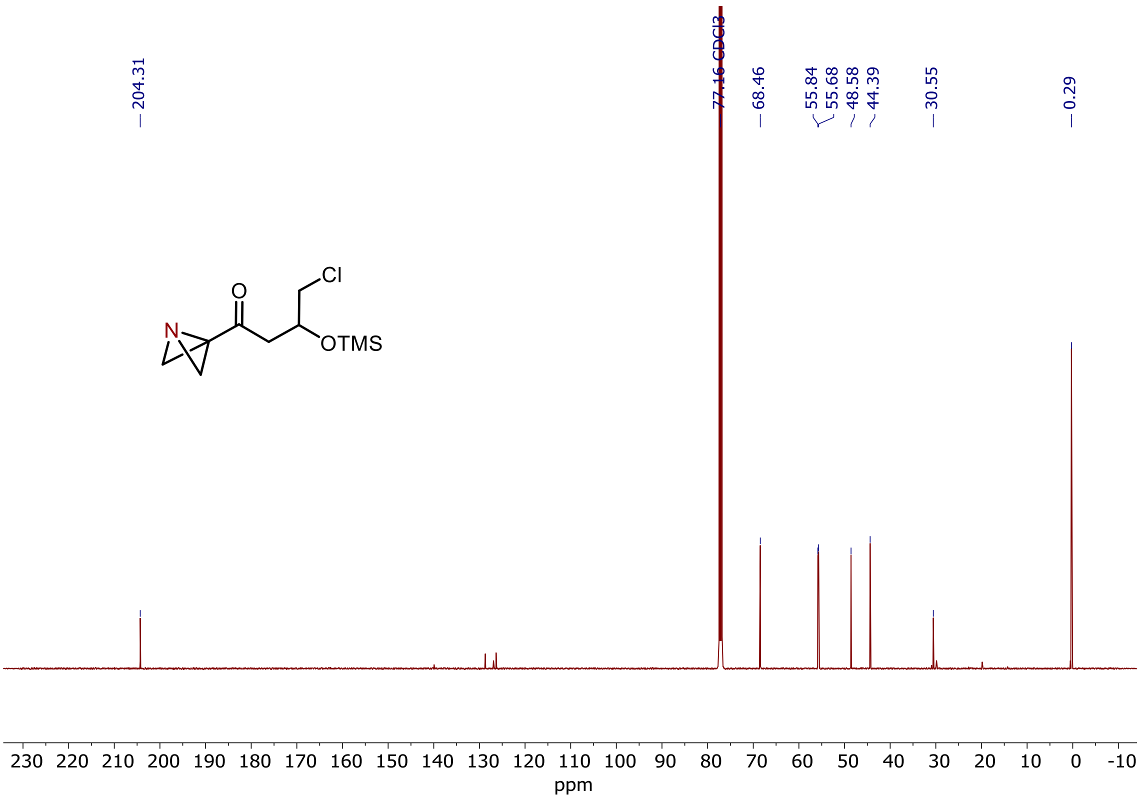

^{1}H NMR (400 MHz, $CDCl_3$) of **4-(Benzyloxy)-1-(1-azabicyclo[1.1.0]butan-3-yl)-3-((trimethylsilyl)oxy)butan-1-one: 3n** (*see procedure*)

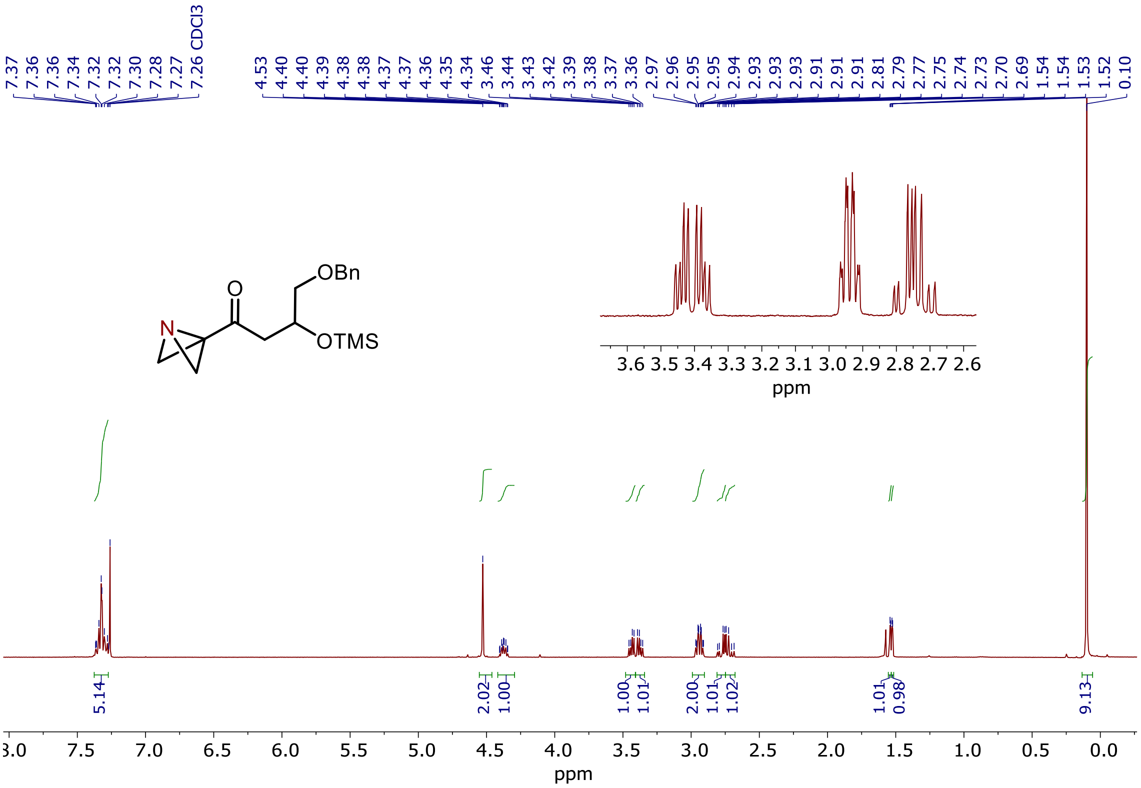

^{13}C NMR (126 MHz, $CDCl_3$) of **4-(Benzyloxy)-1-(1-azabicyclo[1.1.0]butan-3-yl)-3-((trimethylsilyl)oxy)butan-1-one: 3n**

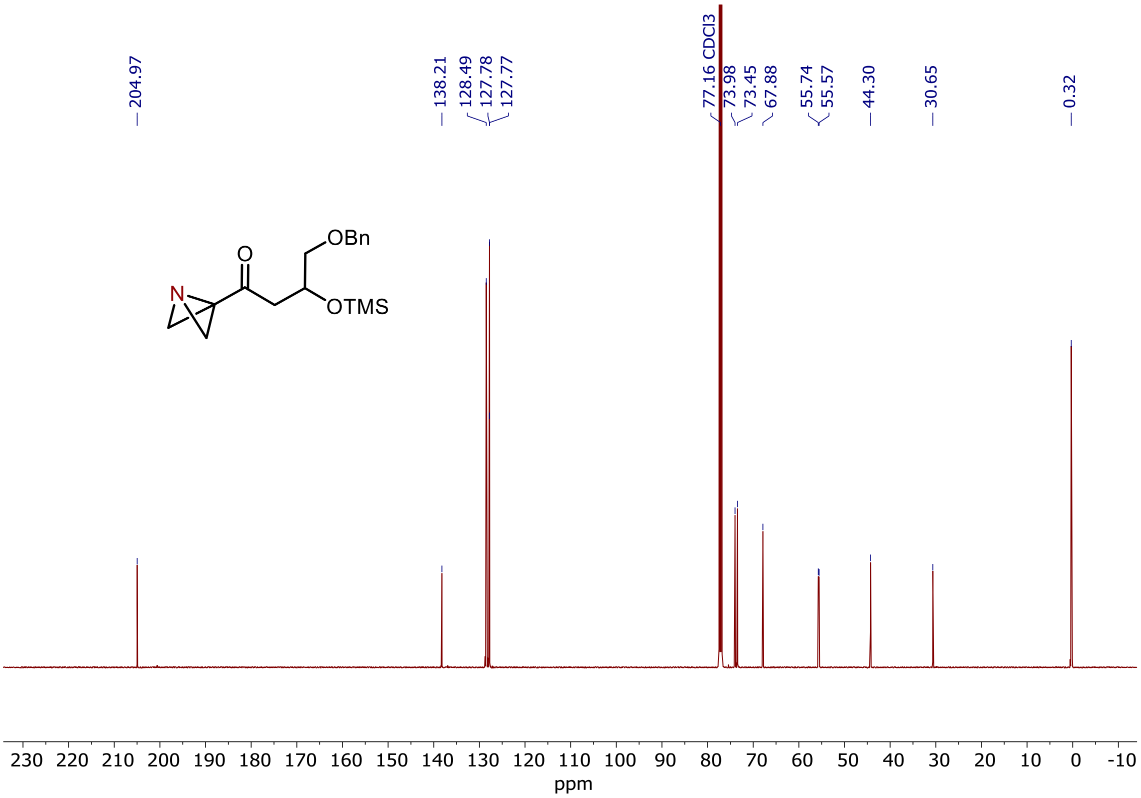

^{1}H NMR (400 MHz, $CDCl_3$) of 1-(1-Azabicyclo[1.1.0]butan-3-yl)-3-methyl-3-((trimethylsilyl)oxy)butan-1-one: 3o (*see procedure*)

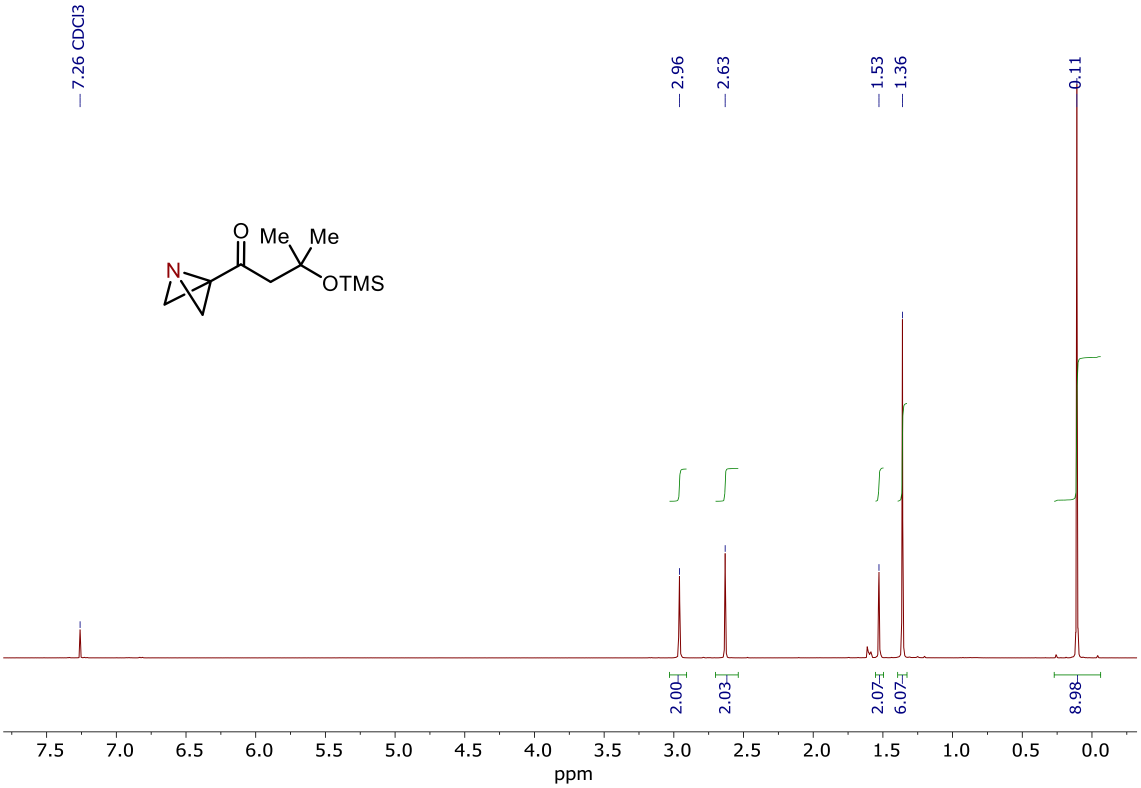

^{13}C NMR (126 MHz, $CDCl_3$) of 1-(1-Azabicyclo[1.1.0]butan-3-yl)-3-methyl-3-((trimethylsilyl)oxy)butan-1-one: 3o

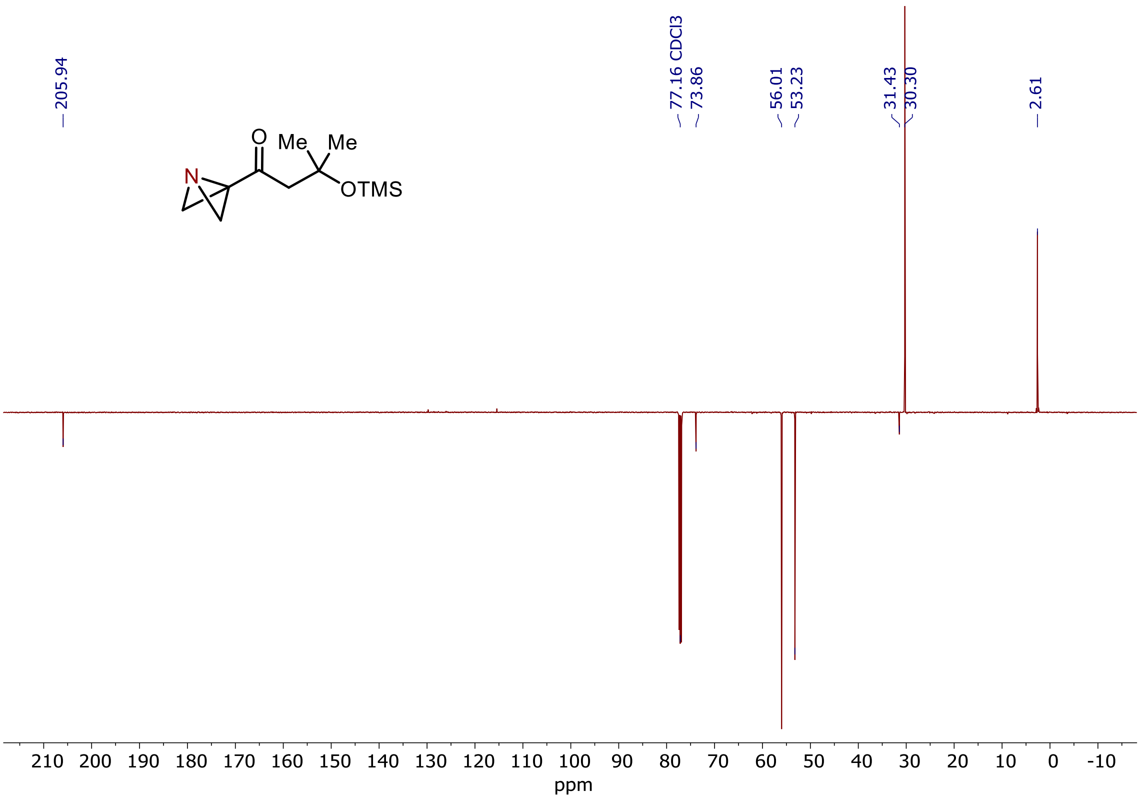

^{1}H NMR (400 MHz, $CDCl_3$) of 1-(1-Azabicyclo[1.1.0]butan-3-yl)-2-(1-((trimethylsilyl)oxy)cyclohexyl)ethan-1-one: 3p (*see procedure*)

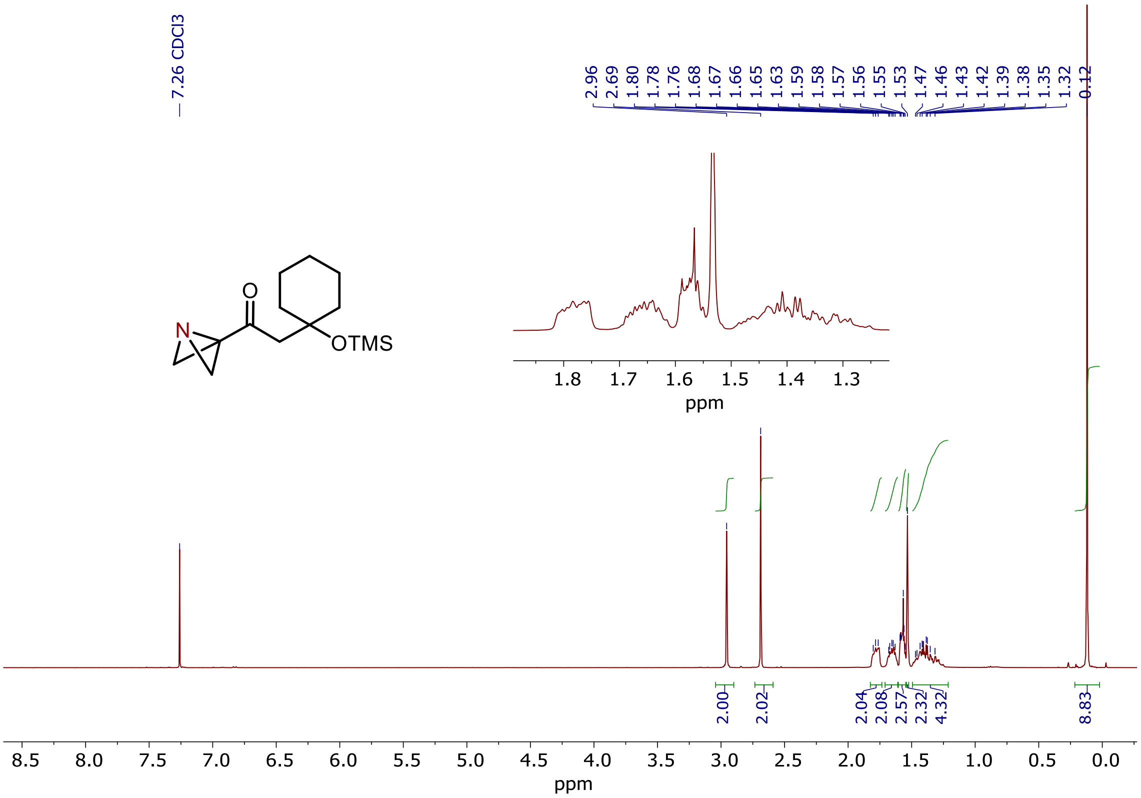

^{13}C NMR (126 MHz, $CDCl_3$) of 1-(1-Azabicyclo[1.1.0]butan-3-yl)-2-(1-((trimethylsilyl)oxy)cyclohexyl)ethan-1-one: 3p

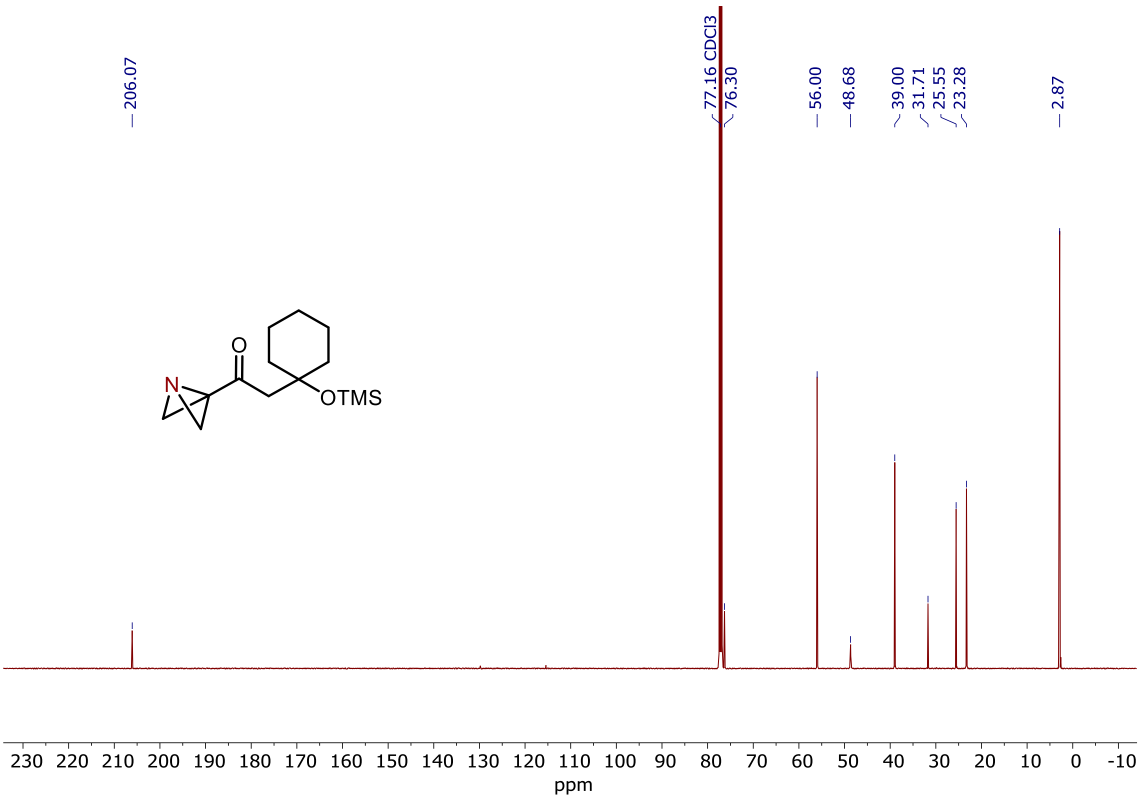

^{1}H NMR (400 MHz, $CDCl_3$) of *syn*-**1-(1-Azabicyclo[1.1.0]butan-3-yl)-2-methyl-3-((trimethylsilyl)oxy)pent-4-en-1-one:** *syn*-**3q** (*see procedure*)

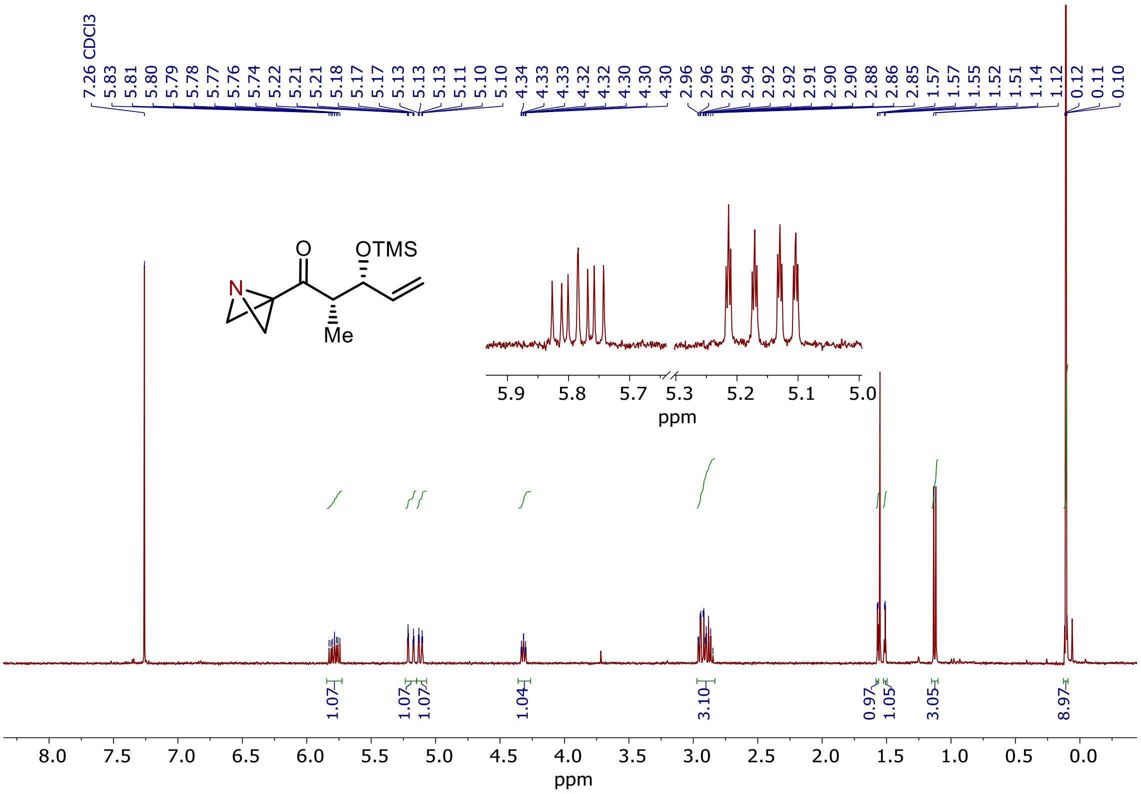

^{13}C NMR (126 MHz, $CDCl_3$) of *syn*-**1-(1-Azabicyclo[1.1.0]butan-3-yl)-2-methyl-3-((trimethylsilyl)oxy)pent-4-en-1-one:** *syn*-**3q**

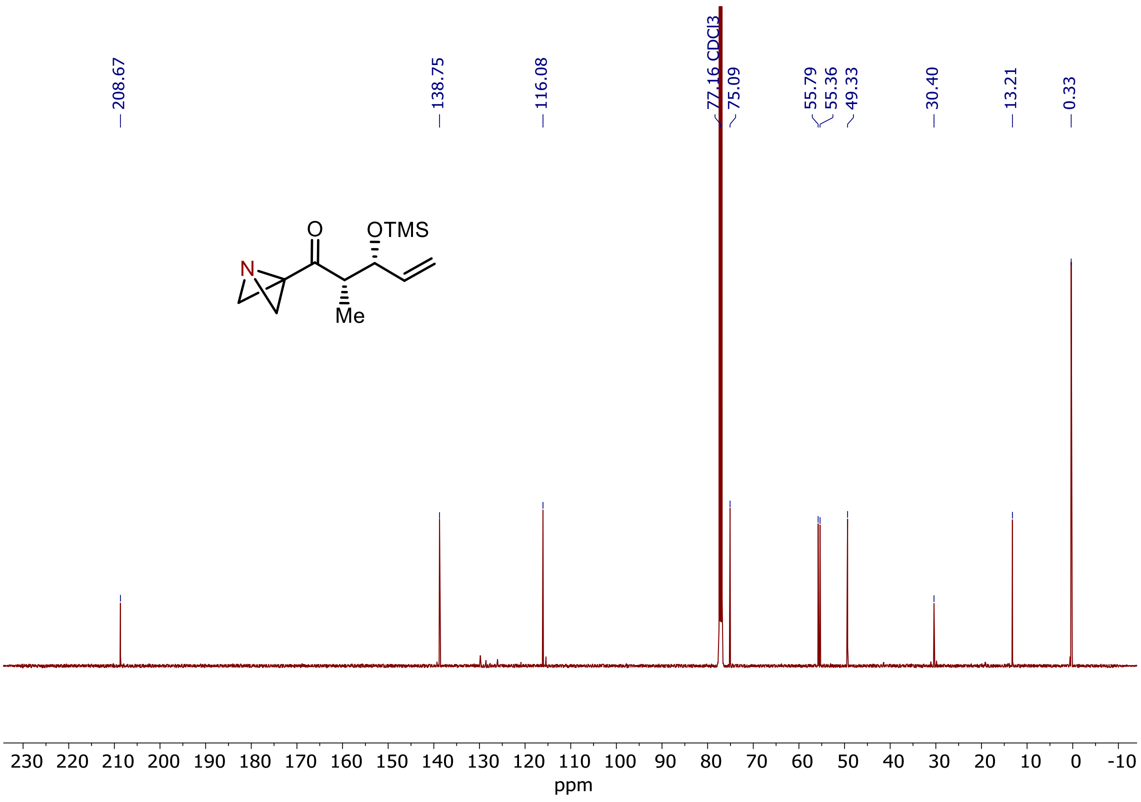

^{1}H NMR (400 MHz, $CDCl_3$) of *anti*-**1-(1-Azabicyclo[1.1.0]butan-3-yl)-2-methyl-3-((trimethylsilyl)oxy)pent-4-en-1-one**: *anti*-**3q** (*see procedure*)

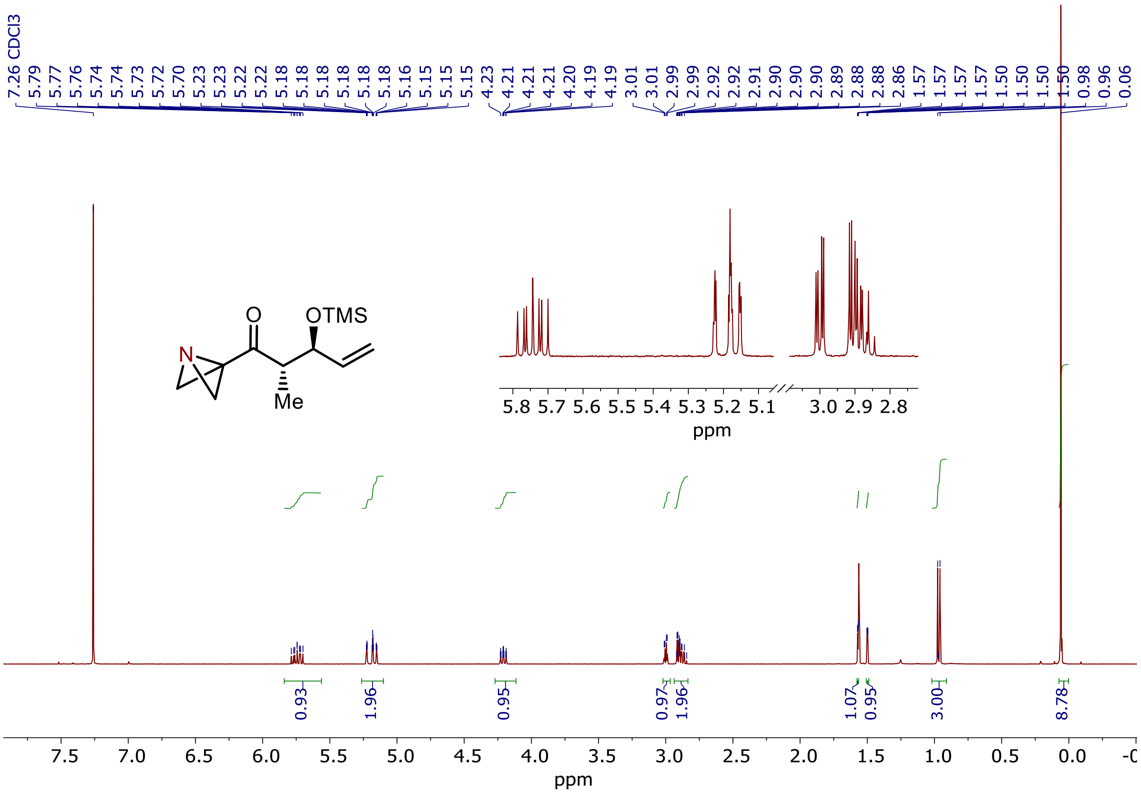

^{13}C NMR (126 MHz, $CDCl_3$) of *anti*-**1-(1-Azabicyclo[1.1.0]butan-3-yl)-2-methyl-3-((trimethylsilyl)oxy)pent-4-en-1-one:** *anti*-**3q**

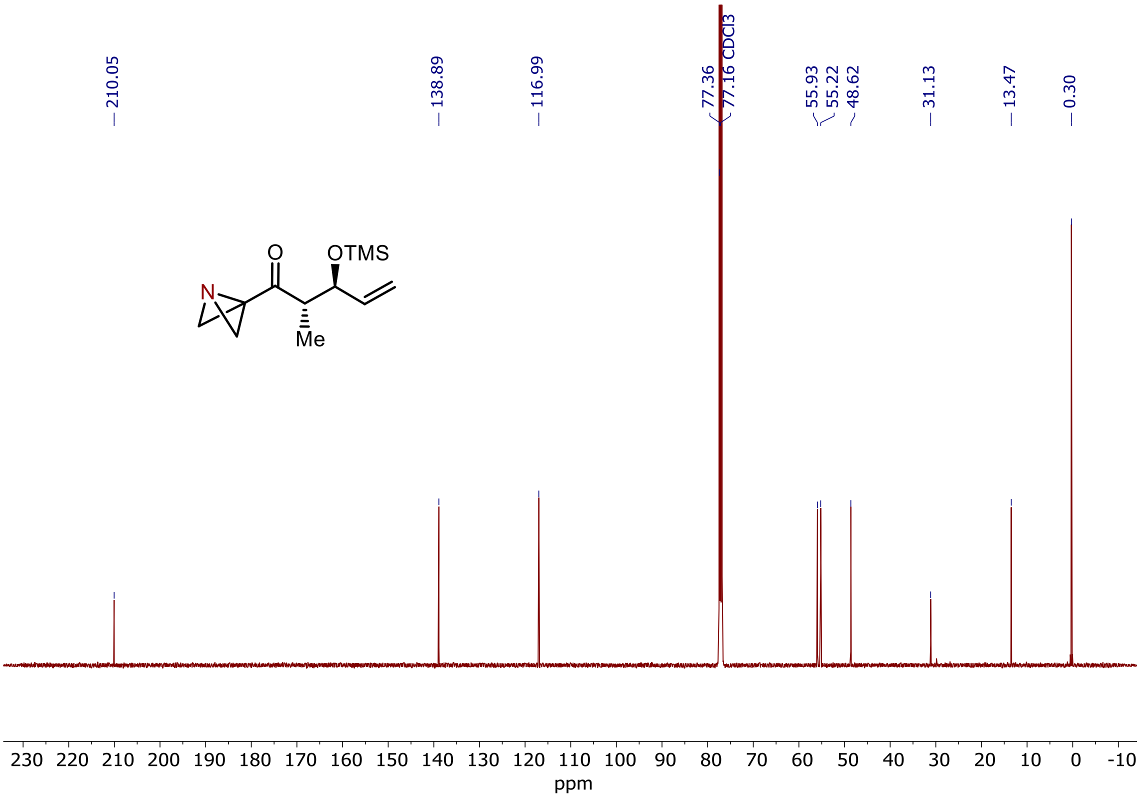

¹H NMR (400 MHz, $CDCl_3$) of **1-(1-Azabicyclo[1.1.0]butan-3-yl)-4-((trimethylsilyl)oxy)pentan-1-one: 3r** (*see procedure*)

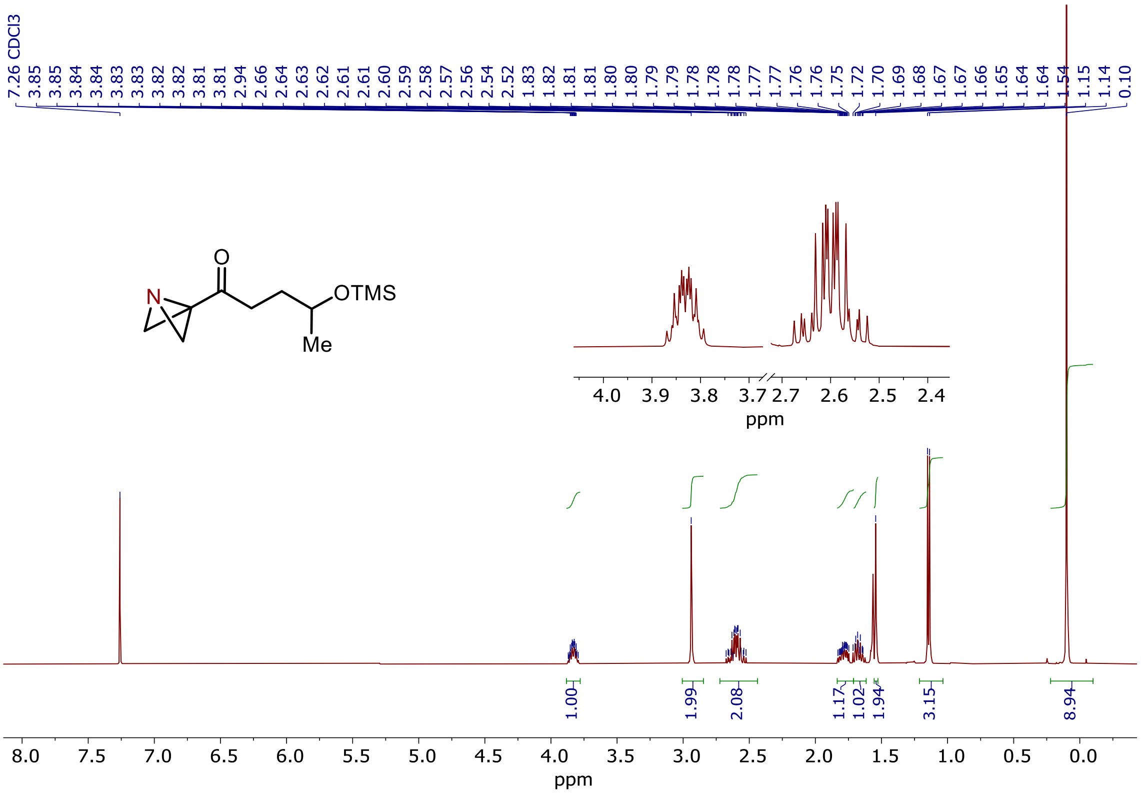

¹³C NMR (126 MHz, $CDCl_3$) of **1-(1-Azabicyclo[1.1.0]butan-3-yl)-4-((trimethylsilyl)oxy)pentan-1-one: 3r**

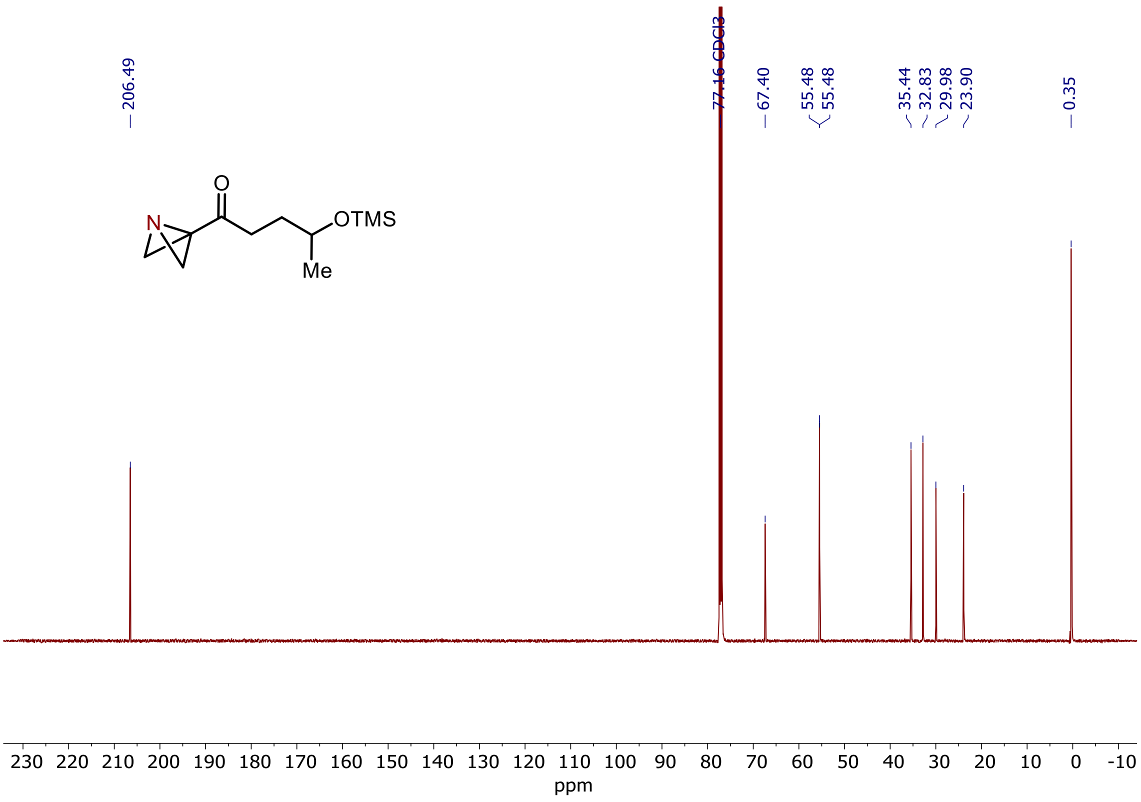

[1]**H NMR** (400 MHz, $CDCl_3$) of **1-(1-Azabicyclo[1.1.0]butan-3-yl)-5-((trimethylsilyl)oxy)pentan-1-one: 3s** (*see procedure*)

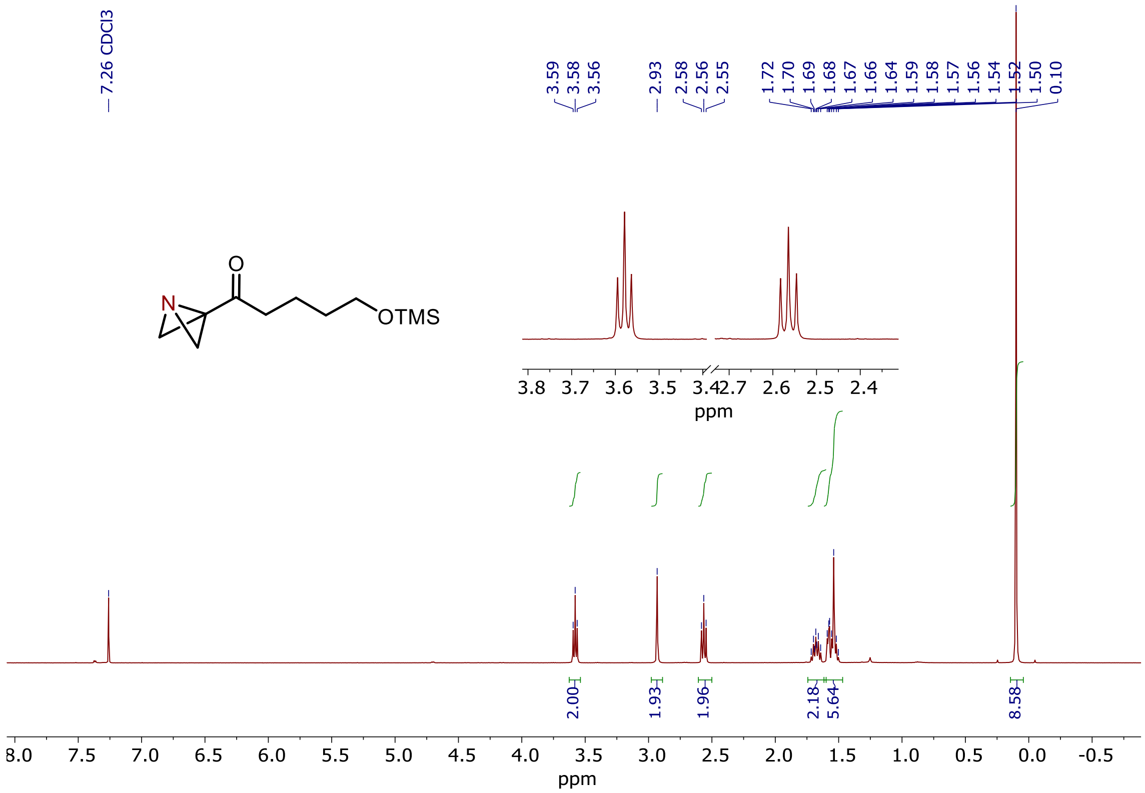

[13]**C NMR** (126 MHz, $CDCl_3$) of **1-(1-Azabicyclo[1.1.0]butan-3-yl)-5-((trimethylsilyl)oxy)pentan-1-one: 3s**

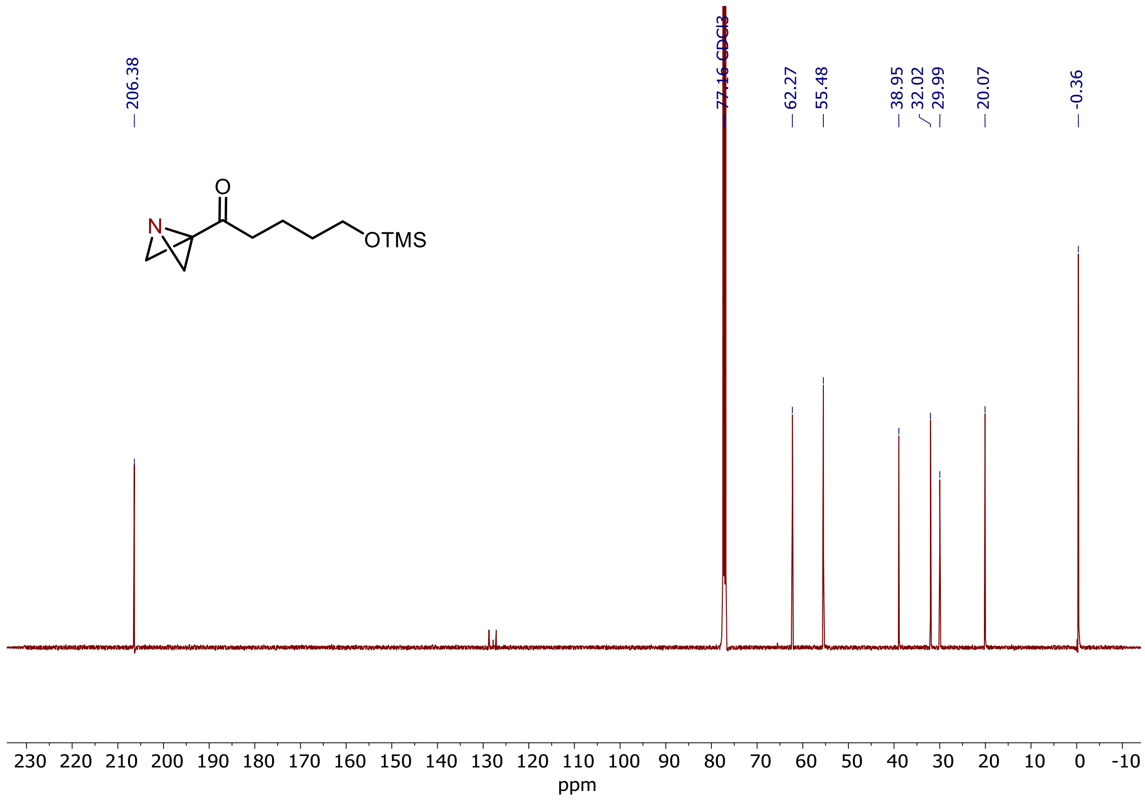

^{1}H NMR (400 MHz, $CDCl_3$) of **(1-Azabicyclo[1.1.0]butan-3-yl)(2-((*tert*-butyldimethylsilyl)oxy)phenyl)methanone: 3t** (*see procedure*)

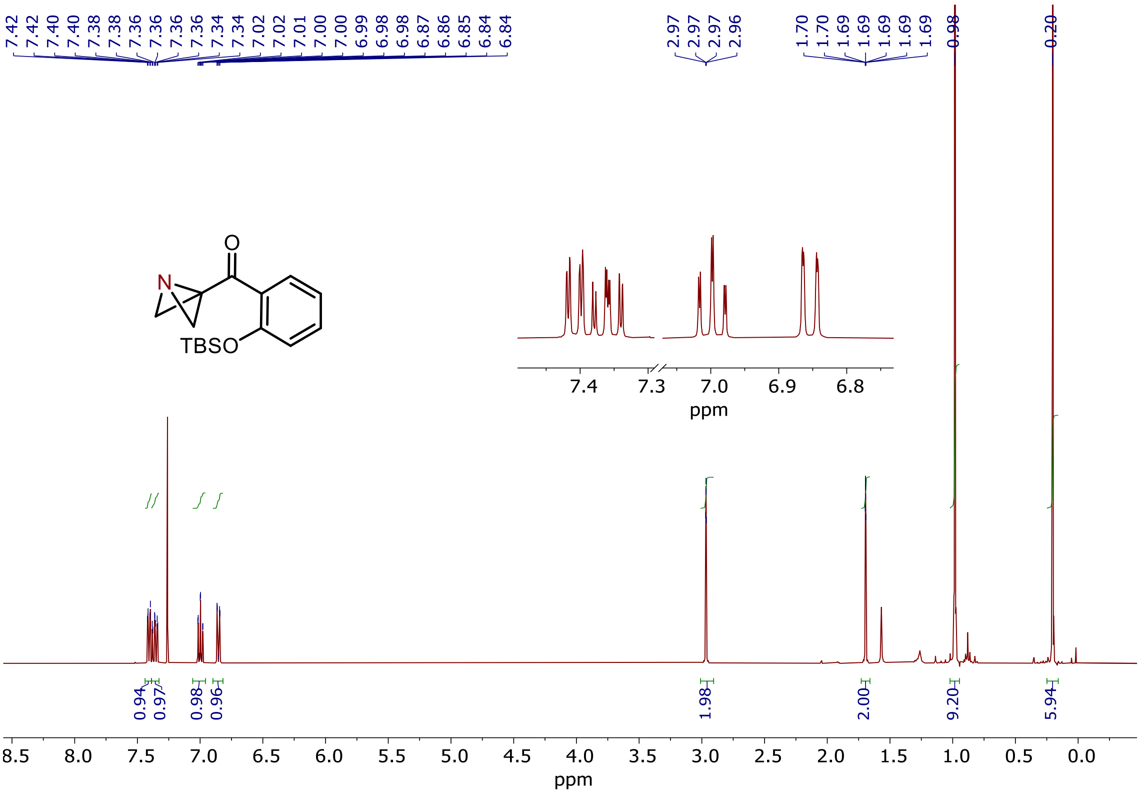

^{13}C NMR (126 MHz, $CDCl_3$) of **(1-Azabicyclo[1.1.0]butan-3-yl)(2-((*tert*-butyldimethylsilyl)oxy)phenyl)methanone: 3t**

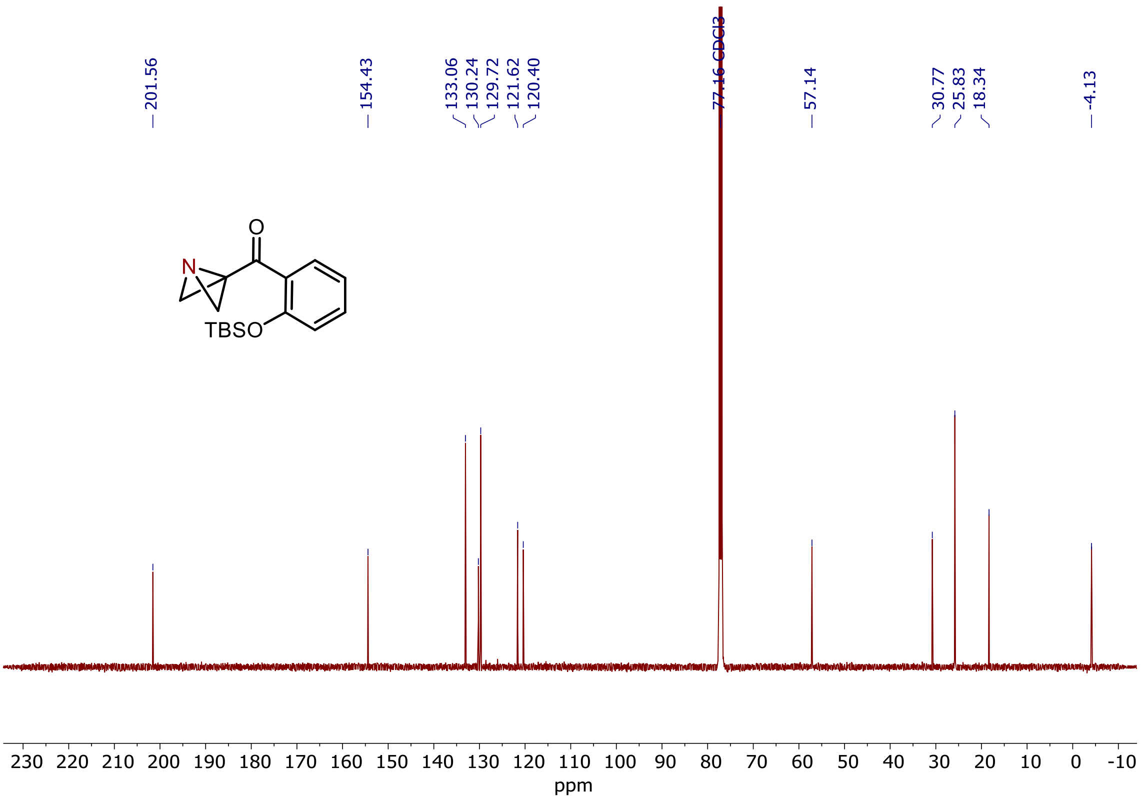

^{1}H NMR (400 MHz, CDCl$_3$) of **(1-Azabicyclo[1.1.0]butan-3-yl)(3-((*tert*-butyldimethylsilyl)oxy)pyridin-2-yl)methanone: 3u** (*see procedure*)

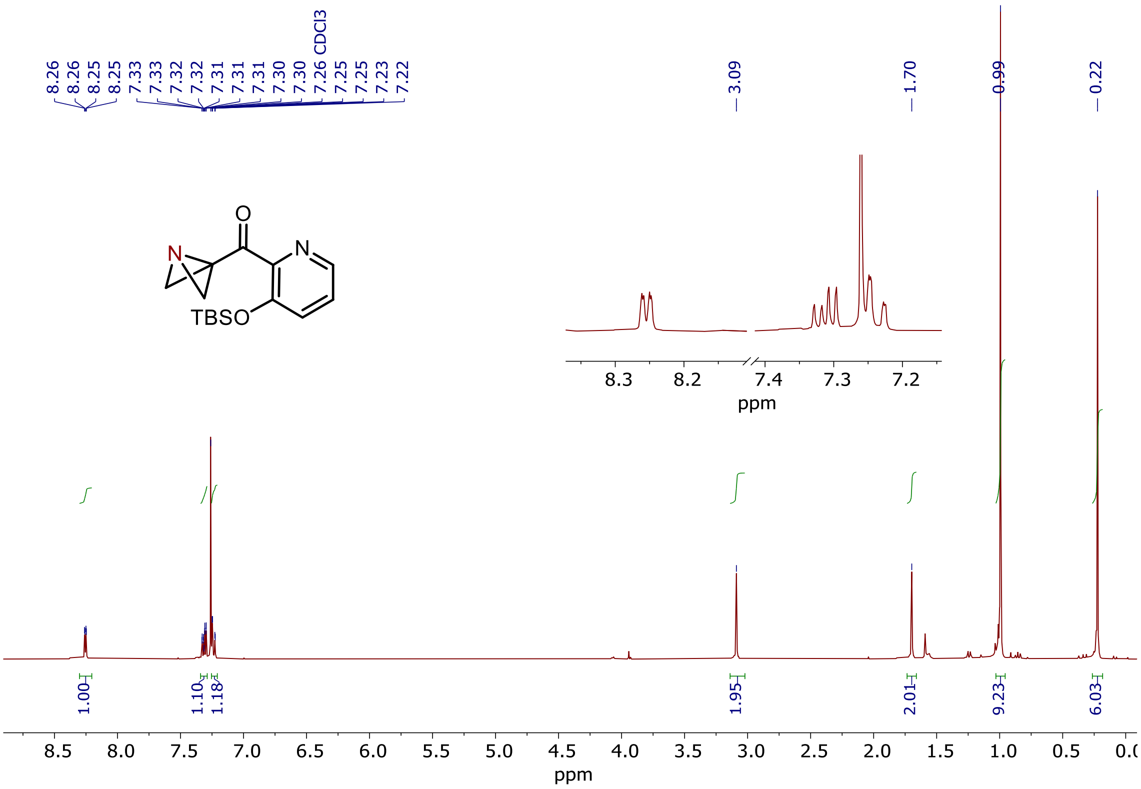

^{13}C NMR (126 MHz, CDCl$_3$) of **(1-Azabicyclo[1.1.0]butan-3-yl)(3-((*tert*-butyldimethylsilyl)oxy)pyridin-2-yl)methanone: 3u**

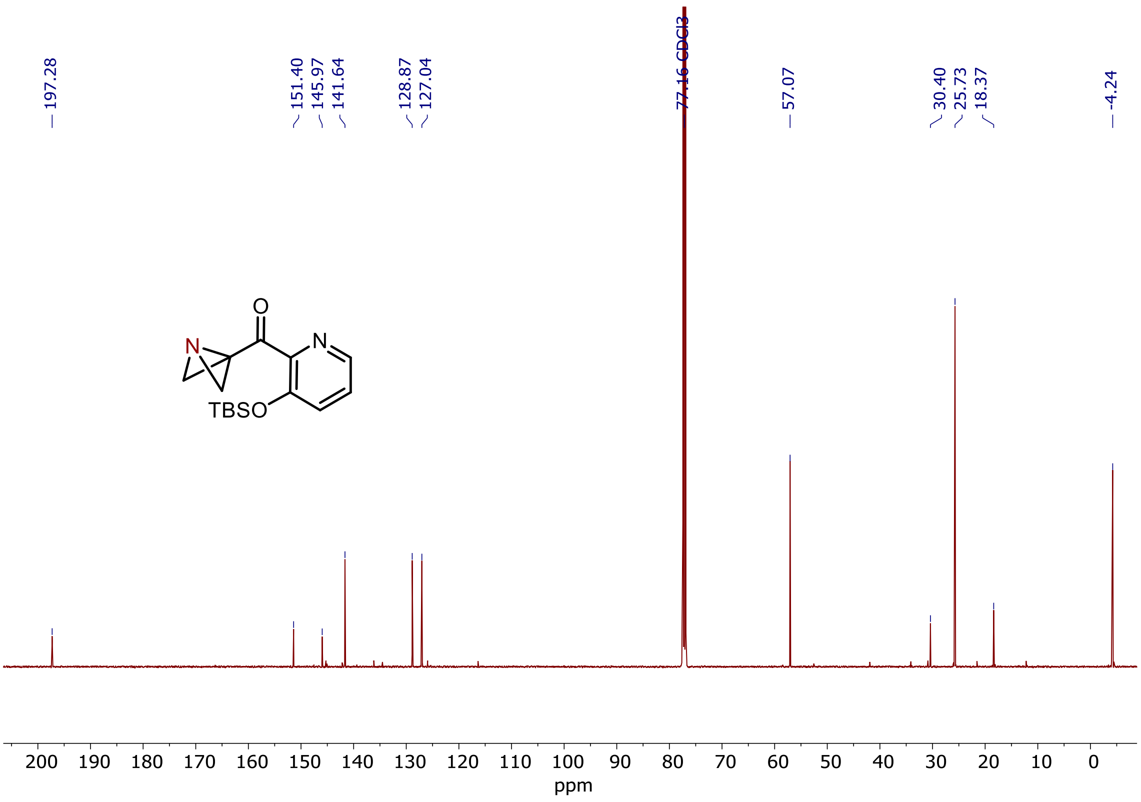

^{1}H NMR (400 MHz, $CDCl_3$) of **3-((3-((*tert*-Butyldimethylsilyl)oxy)pyridin-2-yl)((trimethylsilyl)oxy)methyl)-1-azabicyclo[1.1.0]butane: 3u′** (see procedure)

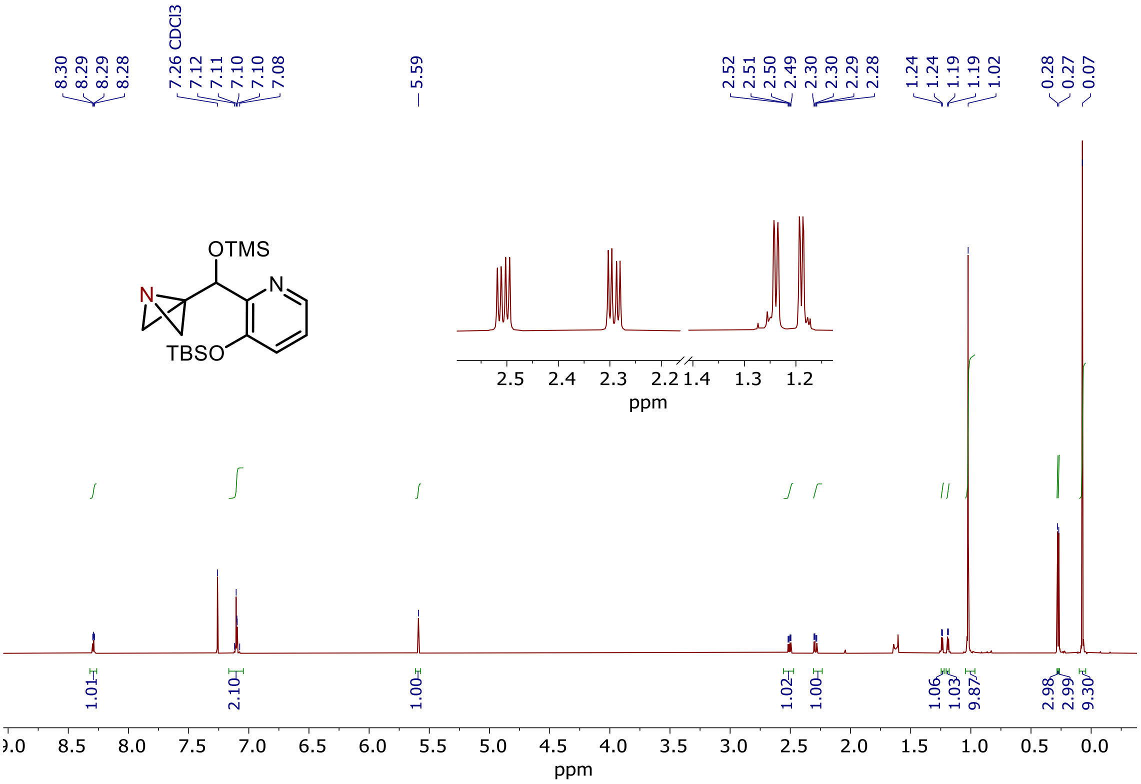

^{13}C NMR (126 MHz, $CDCl_3$) of **3-((3-((*tert*-Butyldimethylsilyl)oxy)pyridin-2-yl)((trimethylsilyl)oxy)methyl)-1-azabicyclo[1.1.0]butane: 3u′**

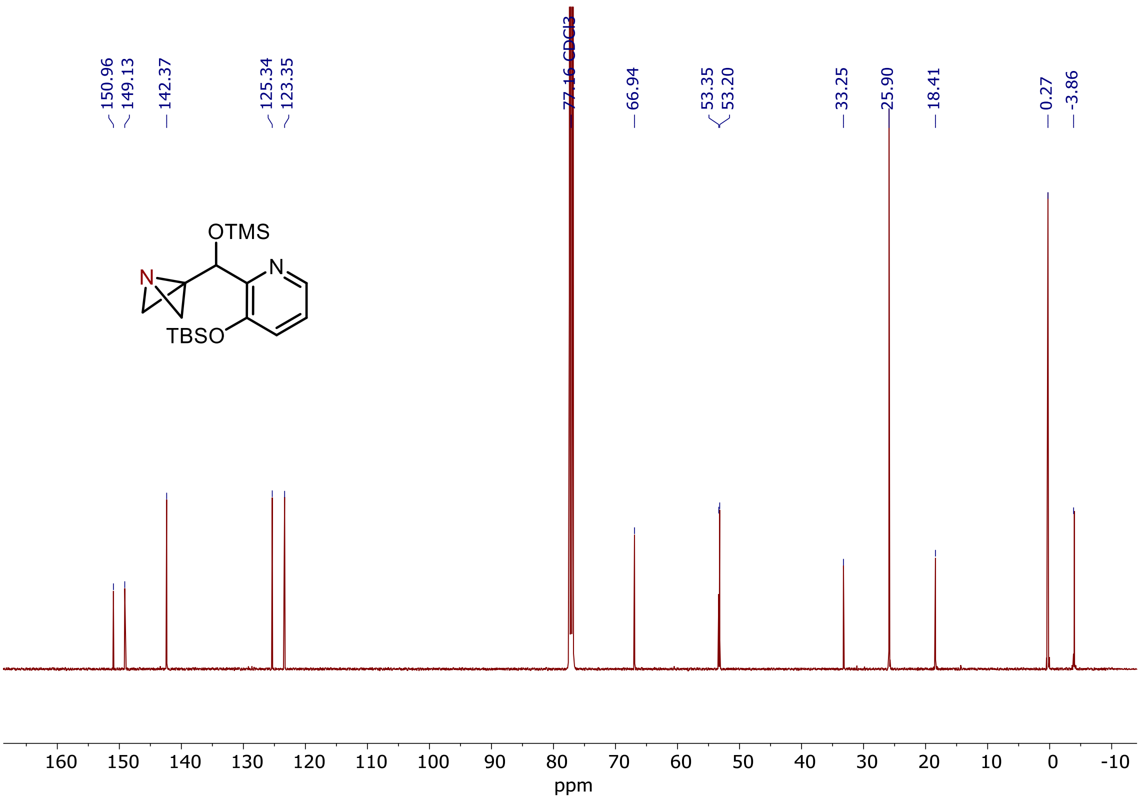

^{1}H NMR (500 MHz, $CDCl_3$) of **(1-Azabicyclo[1.1.0]butan-3-yl)(3-((*tert*-butyldimethylsilyl)oxy)naphthalen-2-yl)methanone: 3v** (*see procedure*)

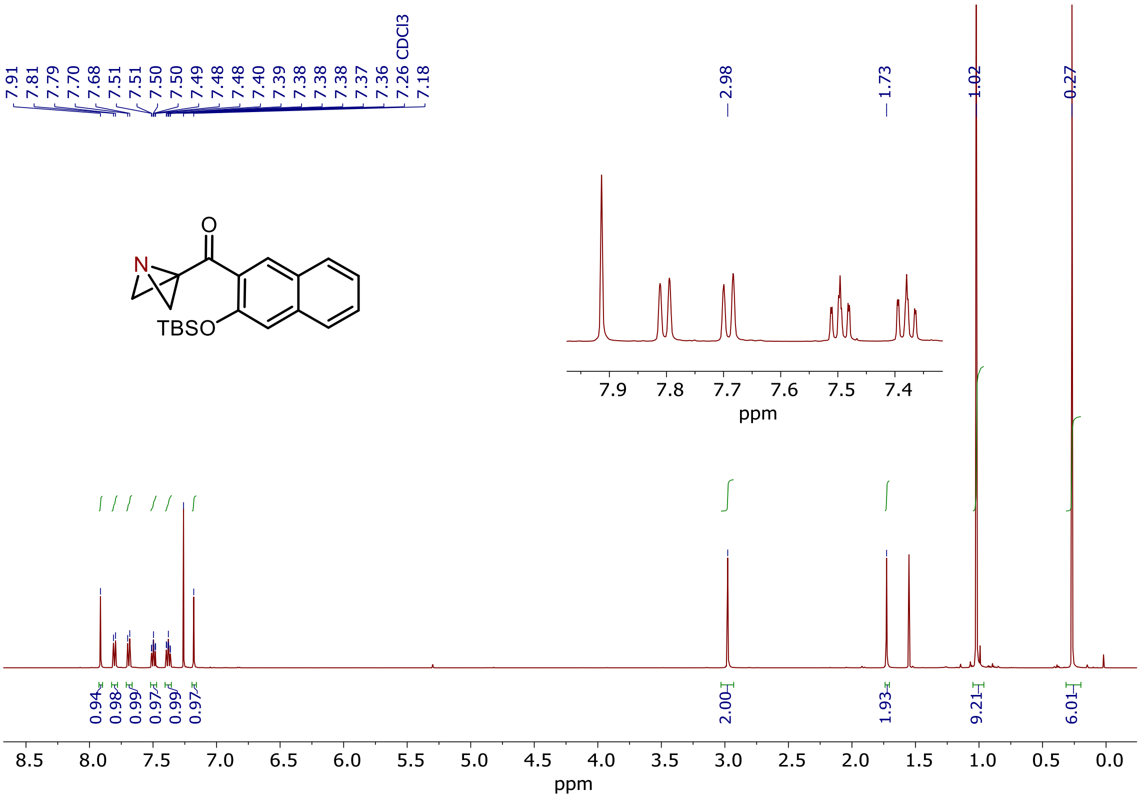

^{13}C NMR (126 MHz, $CDCl_3$) of **(1-Azabicyclo[1.1.0]butan-3-yl)(3-((*tert*-butyldimethylsilyl)oxy)naphthalen-2-yl)methanone: 3v**

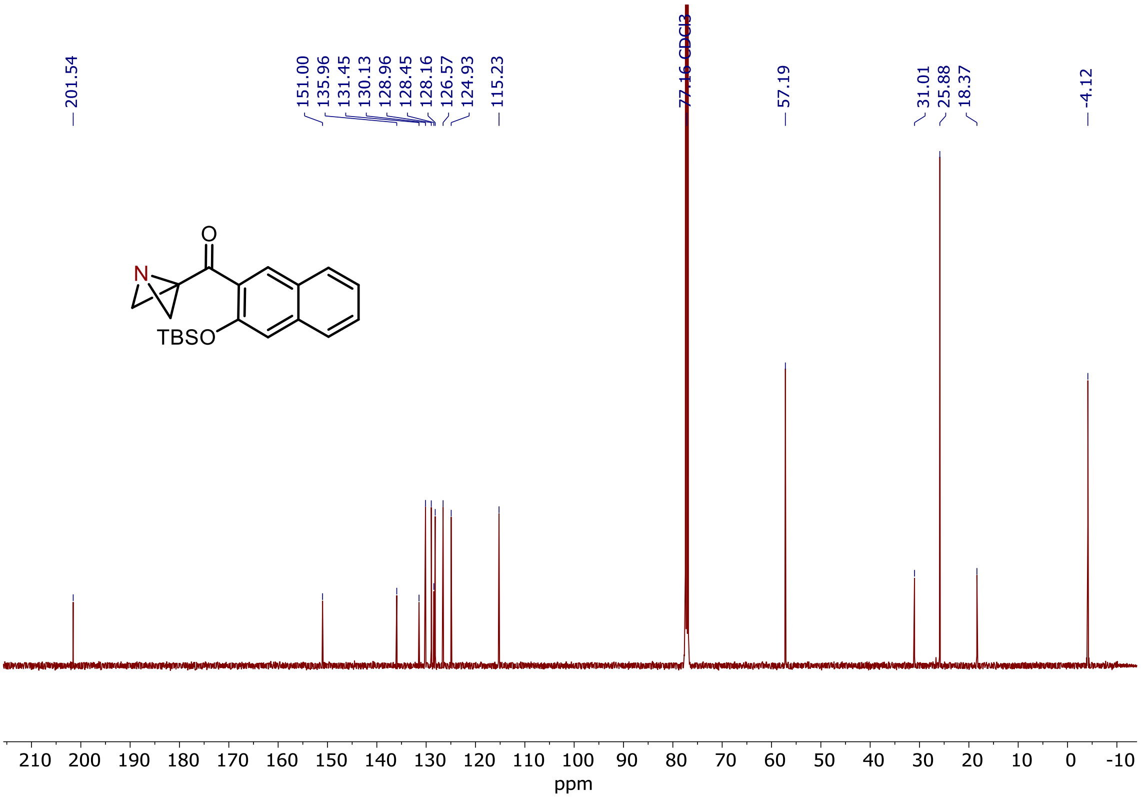

^{1}H NMR (400 MHz, $CDCl_3$) of **2-Methyl-6-(2,2,2-trifluoroacetyl)-1-oxa-6-azaspiro[3.3]heptan-3-one: 4a** (*see procedure*)

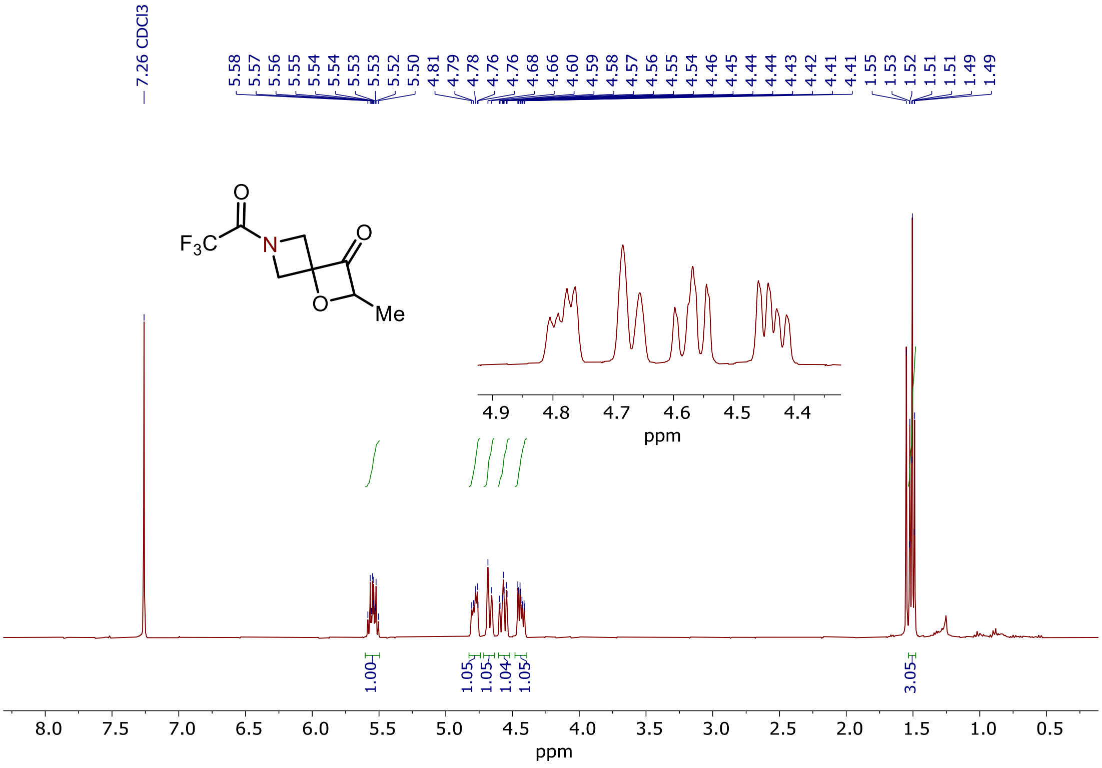

^{13}C NMR (126 MHz, $CDCl_3$) of **2-Methyl-6-(2,2,2-trifluoroacetyl)-1-oxa-6-azaspiro[3.3]heptan-3-one: 4a**

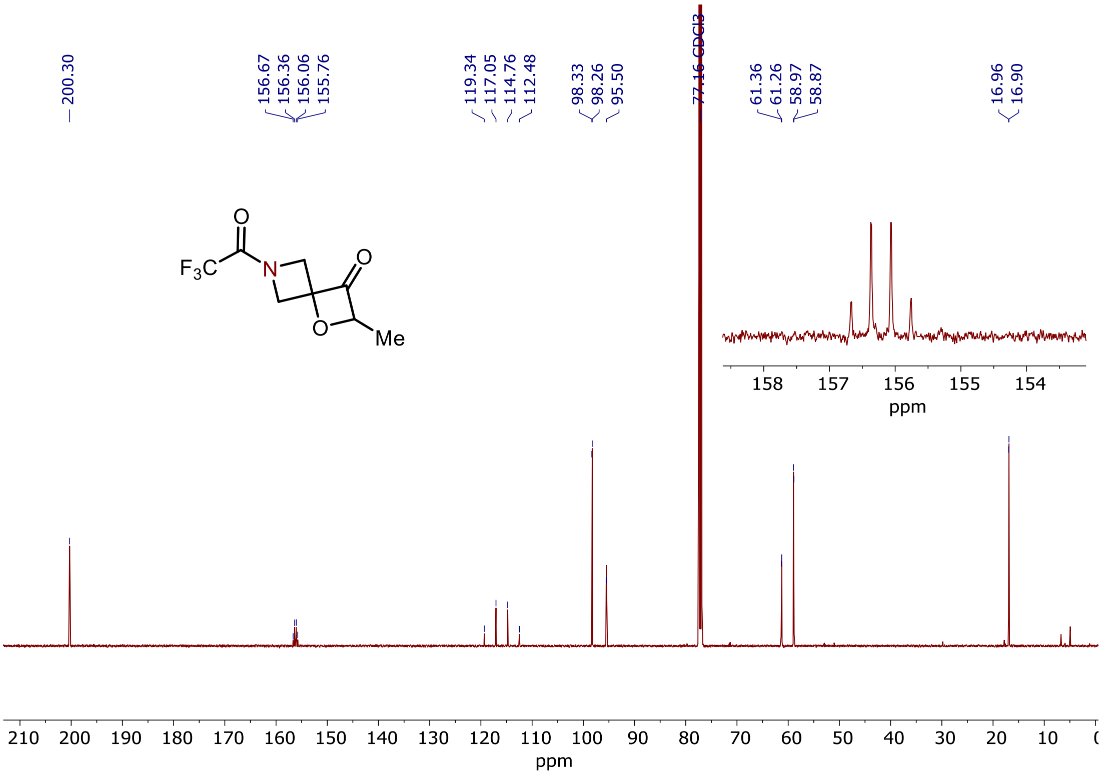

^{19}F NMR (377 MHz, $CDCl_3$) of **2-Methyl-6-(2,2,2-trifluoroacetyl)-1-oxa-6-azaspiro[3.3]heptan-3-one: 4a**

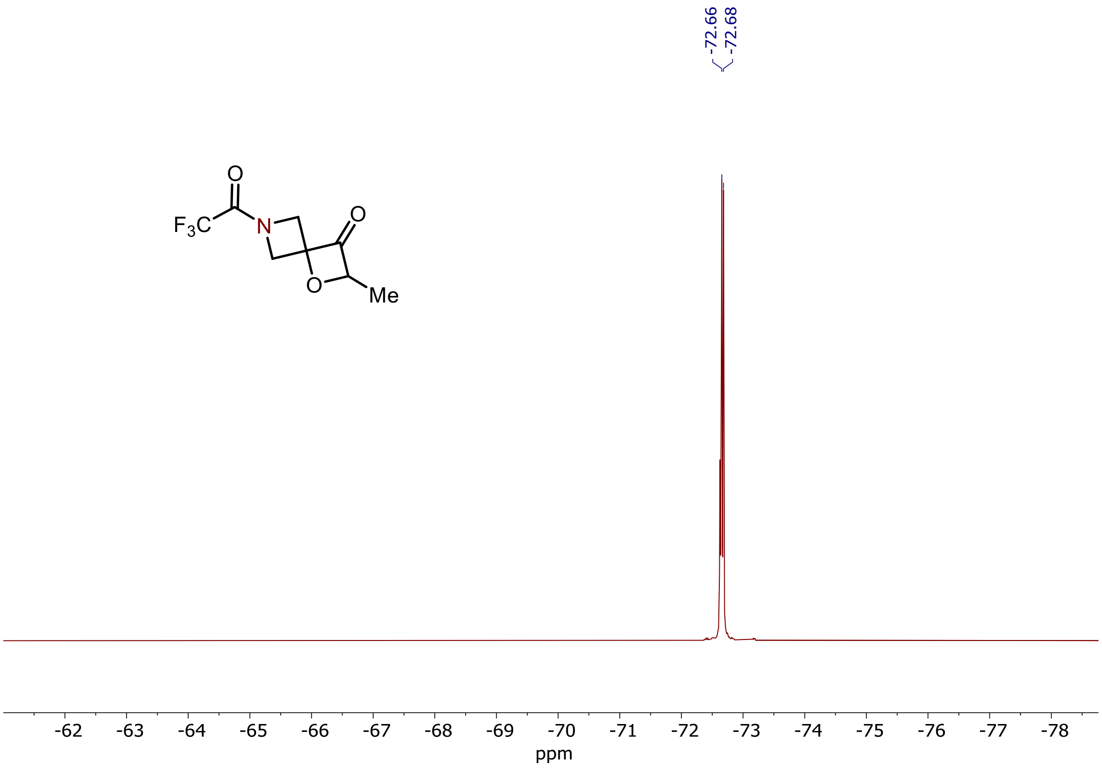

^{1}H NMR (500 MHz, $CDCl_3$) of **2-Benzyl-6-(2,2,2-trifluoroacetyl)-1-oxa-6-azaspiro[3.3]heptan-3-one: 4b** (*see procedure*)

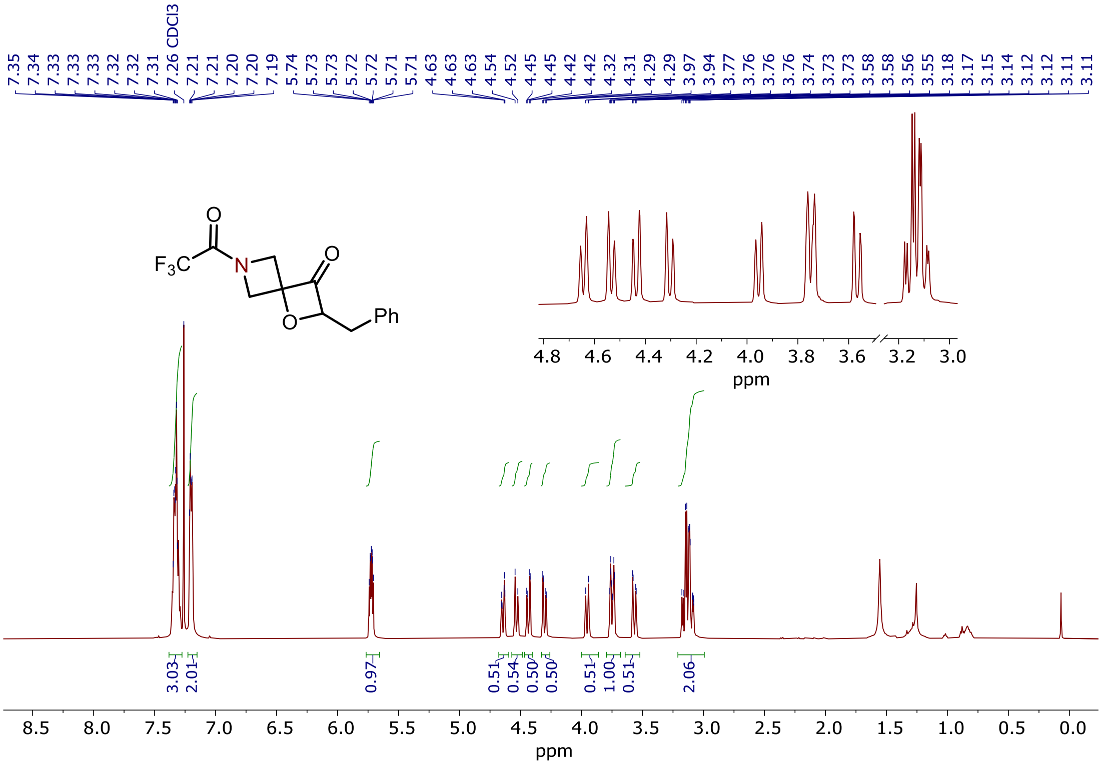

^{13}C NMR (126 MHz, $CDCl_3$) of **2-Benzyl-6-(2,2,2-trifluoroacetyl)-1-oxa-6-azaspiro[3.3]heptan-3-one: 4b**

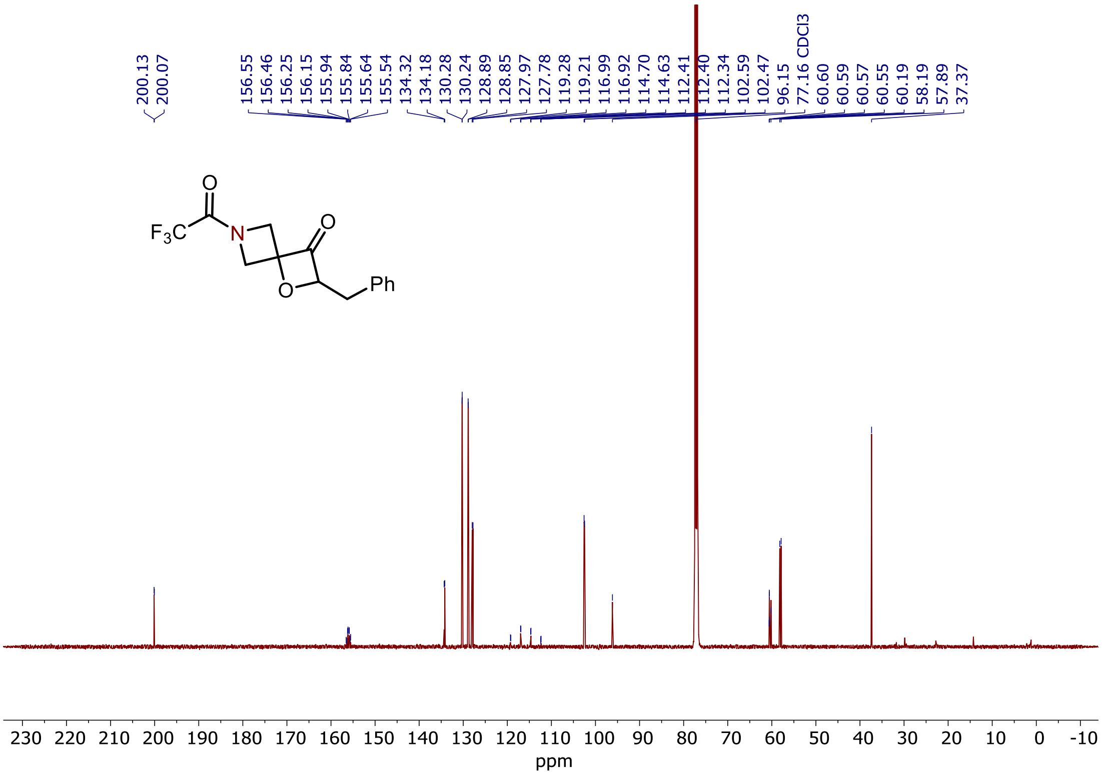

^{19}F NMR (377 MHz, $CDCl_3$) of **2-Benzyl-6-(2,2,2-trifluoroacetyl)-1-oxa-6-azaspiro[3.3]heptan-3-one: 4b**

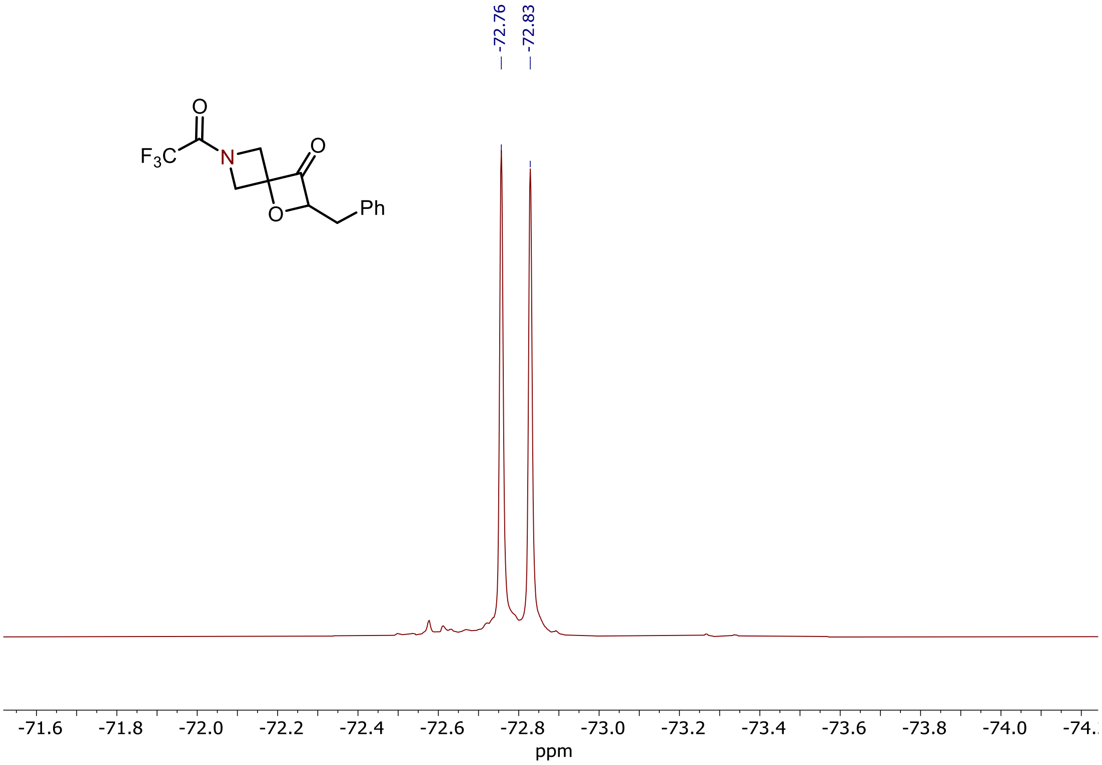

[1]**H NMR** (400 MHz, $CDCl_3$) of **2,2-Dimethyl-6-(2,2,2-trifluoroacetyl)-1-oxa-6-azaspiro[3.3]heptan-3-one: 4c** (*see procedure*)

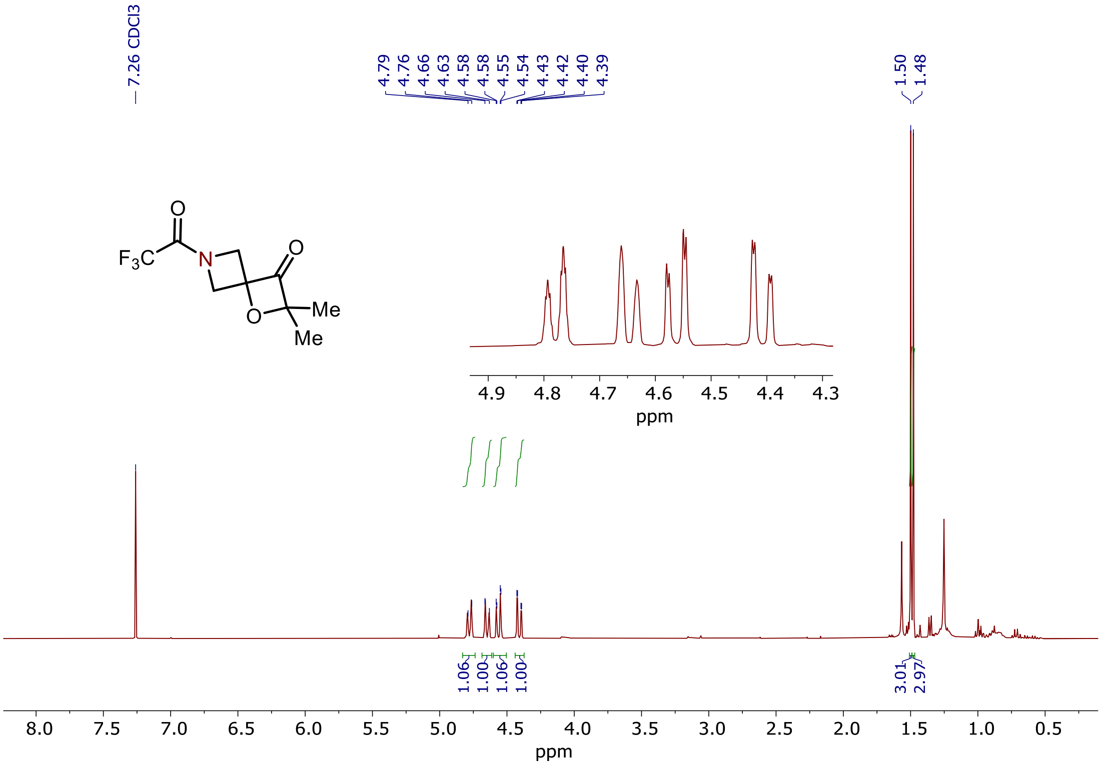

[13]**C NMR** (126 MHz, $CDCl_3$) of **2,2-Dimethyl-6-(2,2,2-trifluoroacetyl)-1-oxa-6-azaspiro[3.3]heptan-3-one: 4c**

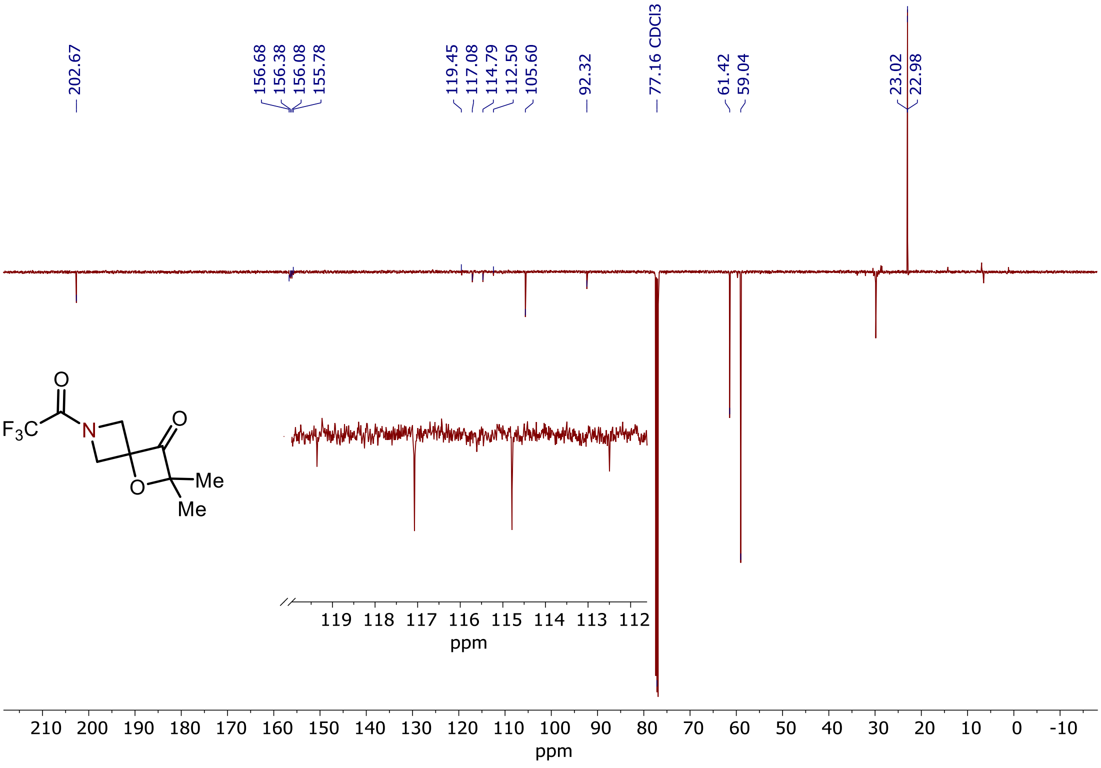

^{19}F NMR (377 MHz, $CDCl_3$) of **2,2-Dimethyl-6-(2,2,2-trifluoroacetyl)-1-oxa-6-azaspiro[3.3]heptan-3-one: 4c**

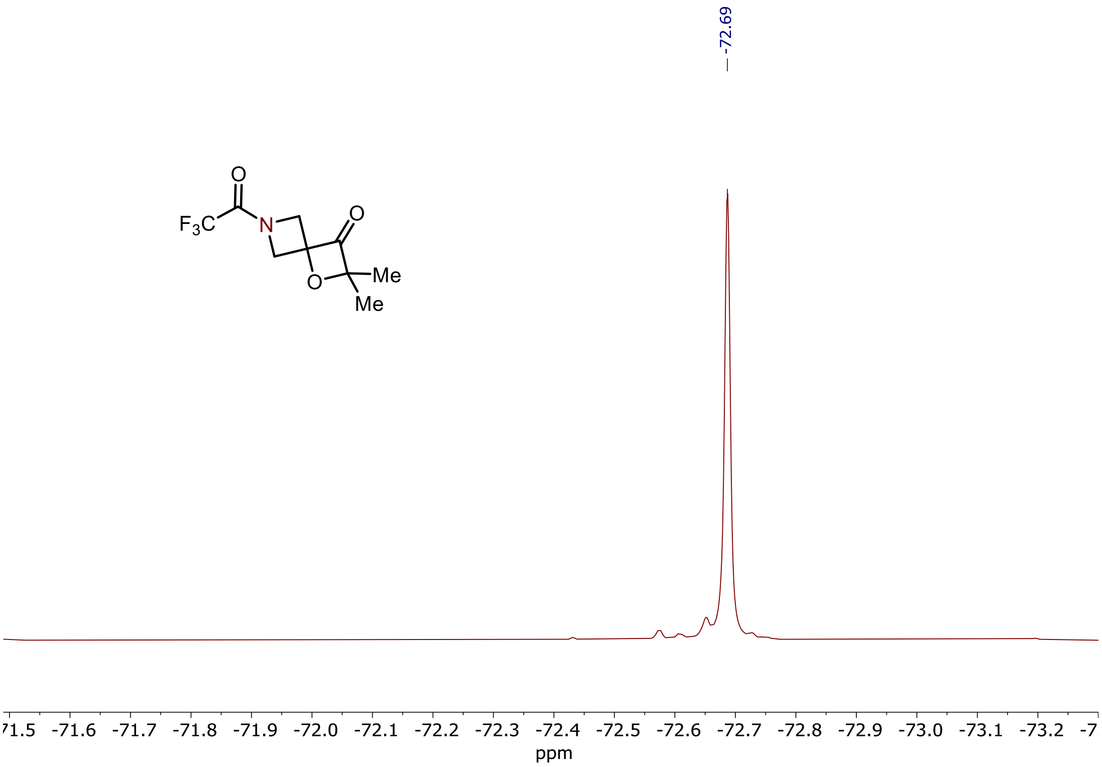

^{1}H NMR (400 MHz, $CDCl_3$) of **7-Methyl-2-(2,2,2-trifluoroacetyl)-5-oxa-2-azaspiro[3.4]octan-8-one: 4d** (*see procedure*)

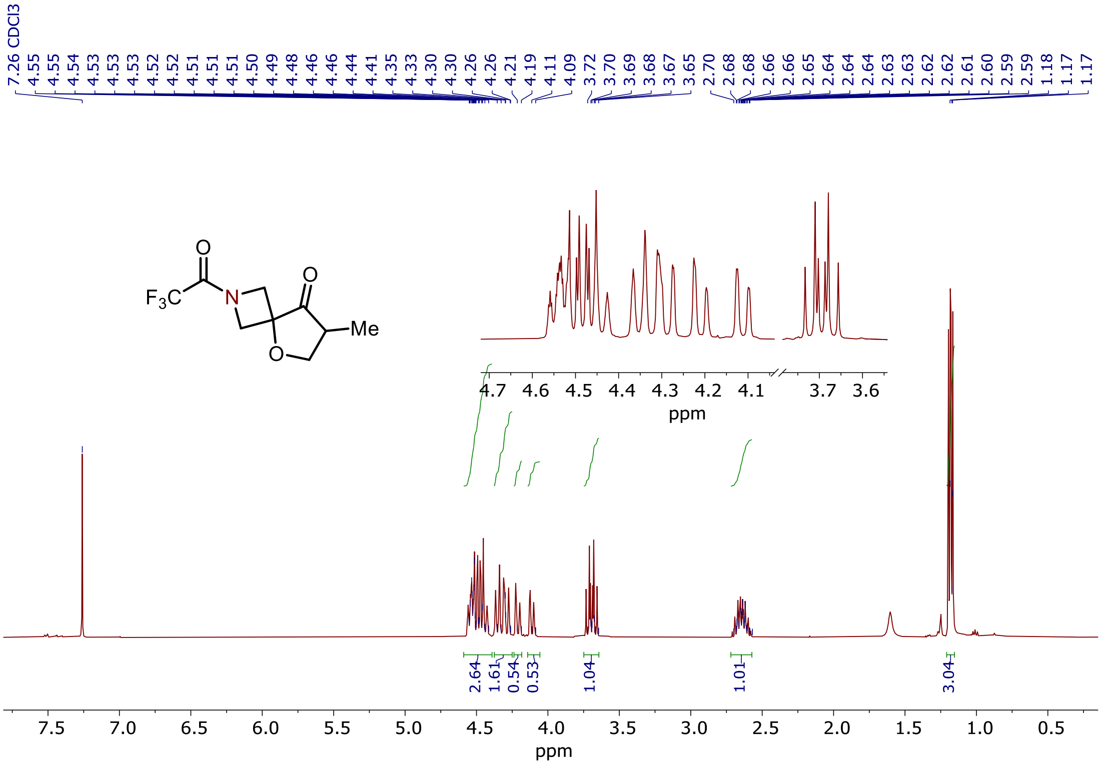

^{13}C NMR (126 MHz, $CDCl_3$) of **7-Methyl-2-(2,2,2-trifluoroacetyl)-5-oxa-2-azaspiro[3.4]octan-8-one: 4d**

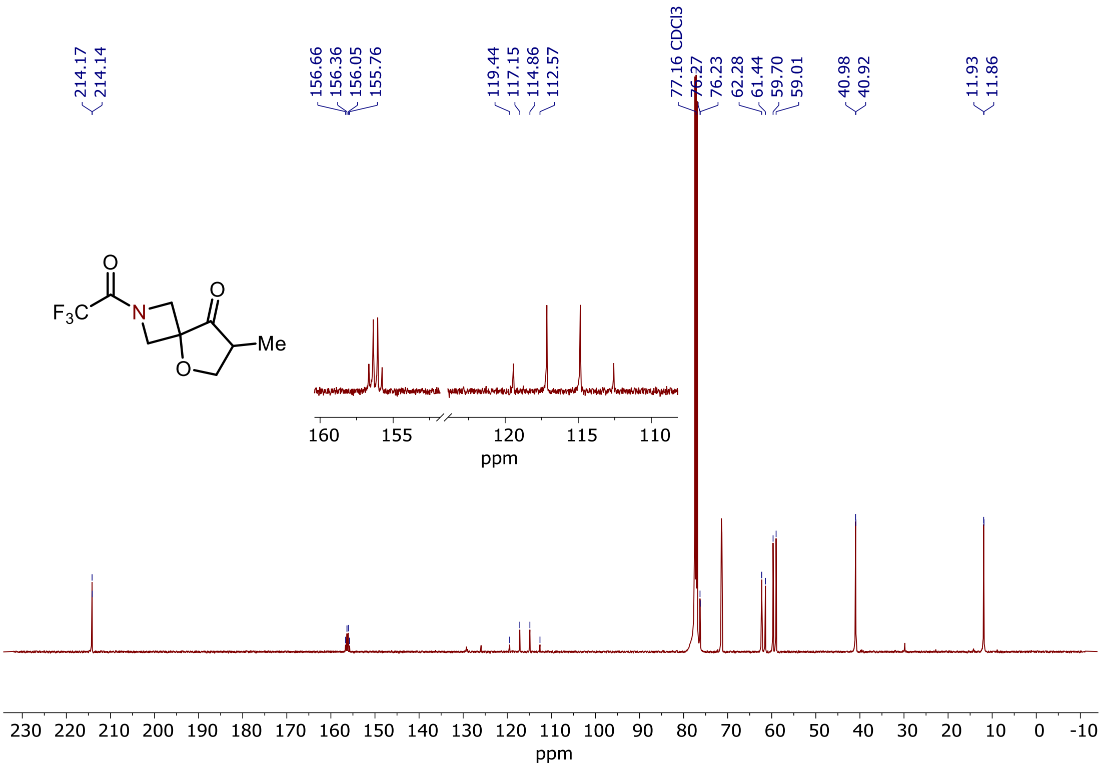

^{19}F NMR (377 MHz, $CDCl_3$) of **7-Methyl-2-(2,2,2-trifluoroacetyl)-5-oxa-2-azaspiro[3.4]octan-8-one: 4d**

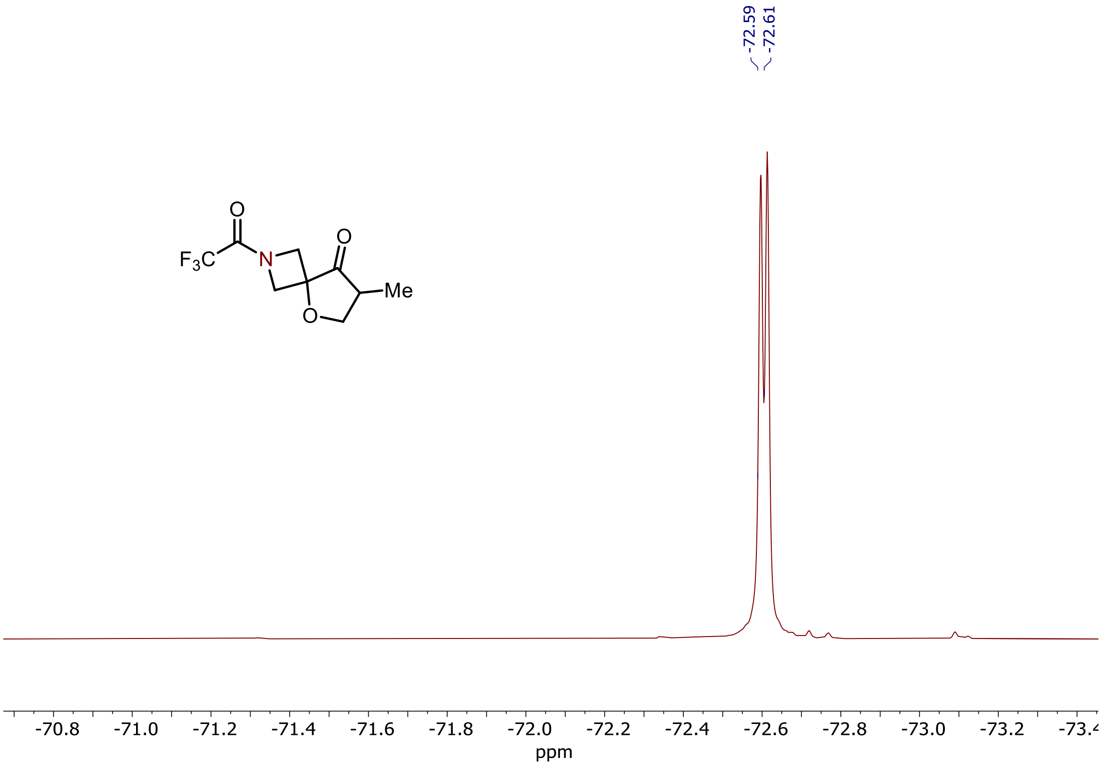

^{1}H NMR (500 MHz, $CDCl_3$) of **7-Phenyl-2-(2,2,2-trifluoroacetyl)-5-oxa-2-azaspiro[3.4]octan-8-one: 4e** (*see procedure*)

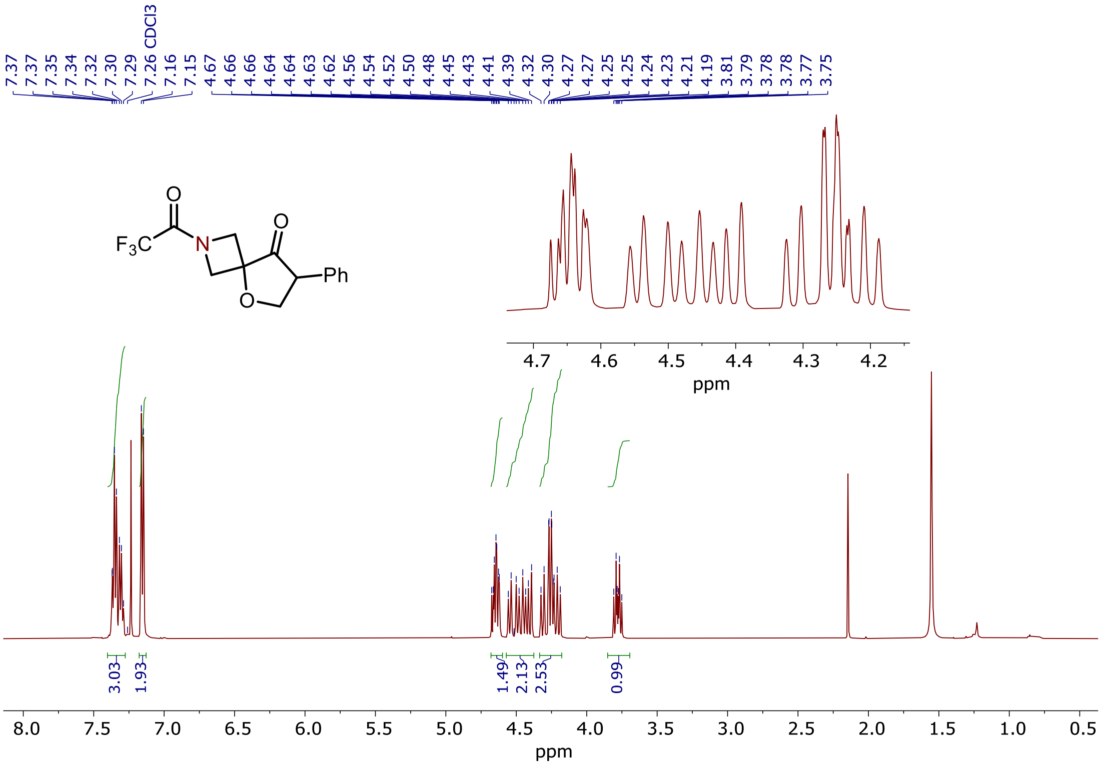

^{13}C NMR (126 MHz, $CDCl_3$) of **7-Phenyl-2-(2,2,2-trifluoroacetyl)-5-oxa-2-azaspiro[3.4]octan-8-one: 4e**

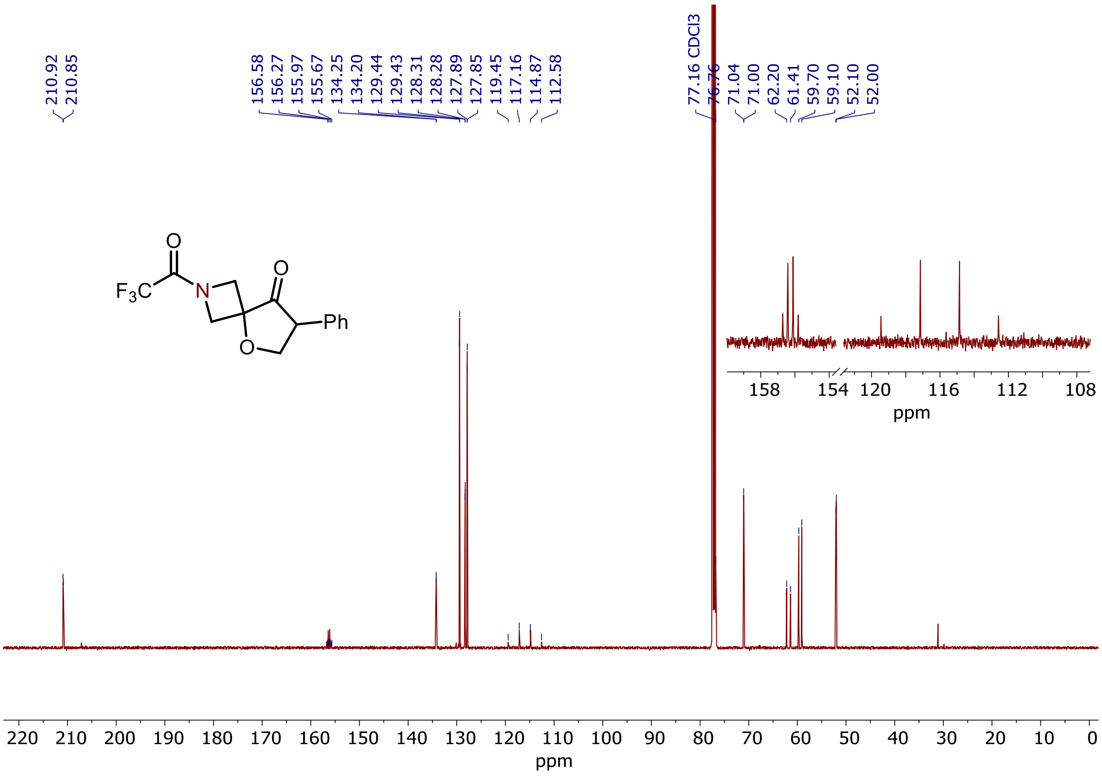

^{19}F NMR (377 MHz, $CDCl_3$) of **7-Phenyl-2-(2,2,2-trifluoroacetyl)-5-oxa-2-azaspiro[3.4]octan-8-one: 4e**

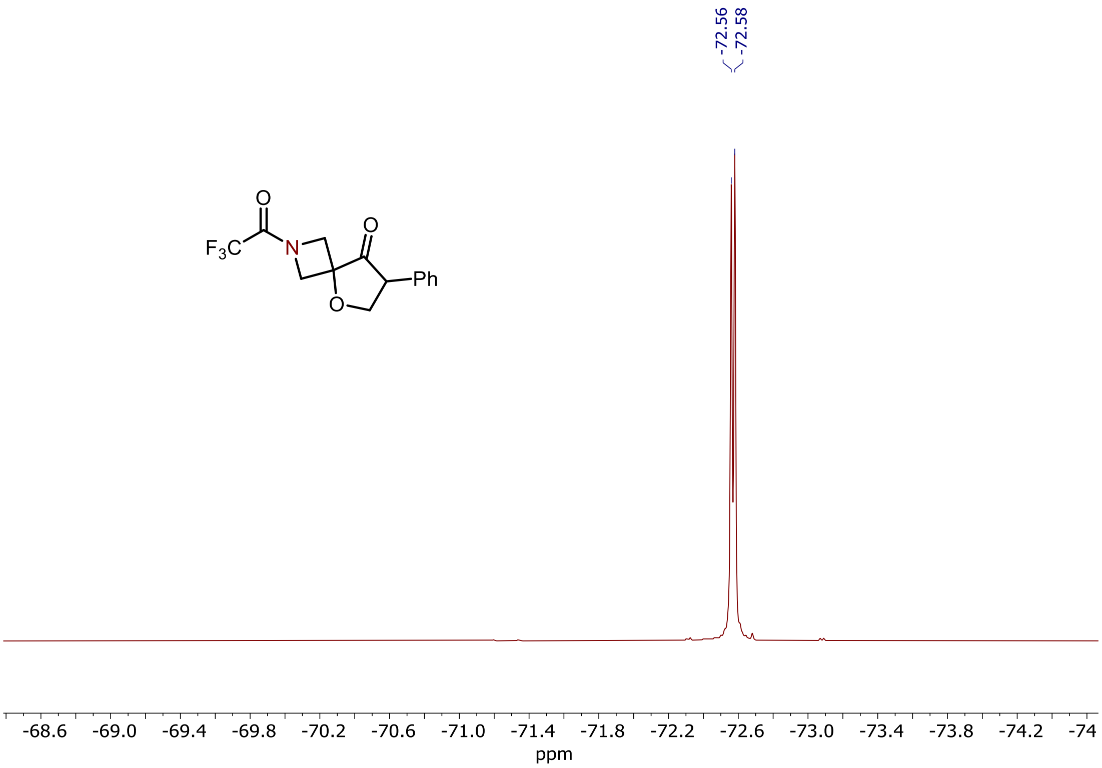

^{1}H NMR (500 MHz, $CDCl_3$) of **7,7-Dimethyl-2-(2,2,2-trifluoroacetyl)-5-oxa-2-azaspiro[3.4]octan-8-one: 4f** (*see procedure*)

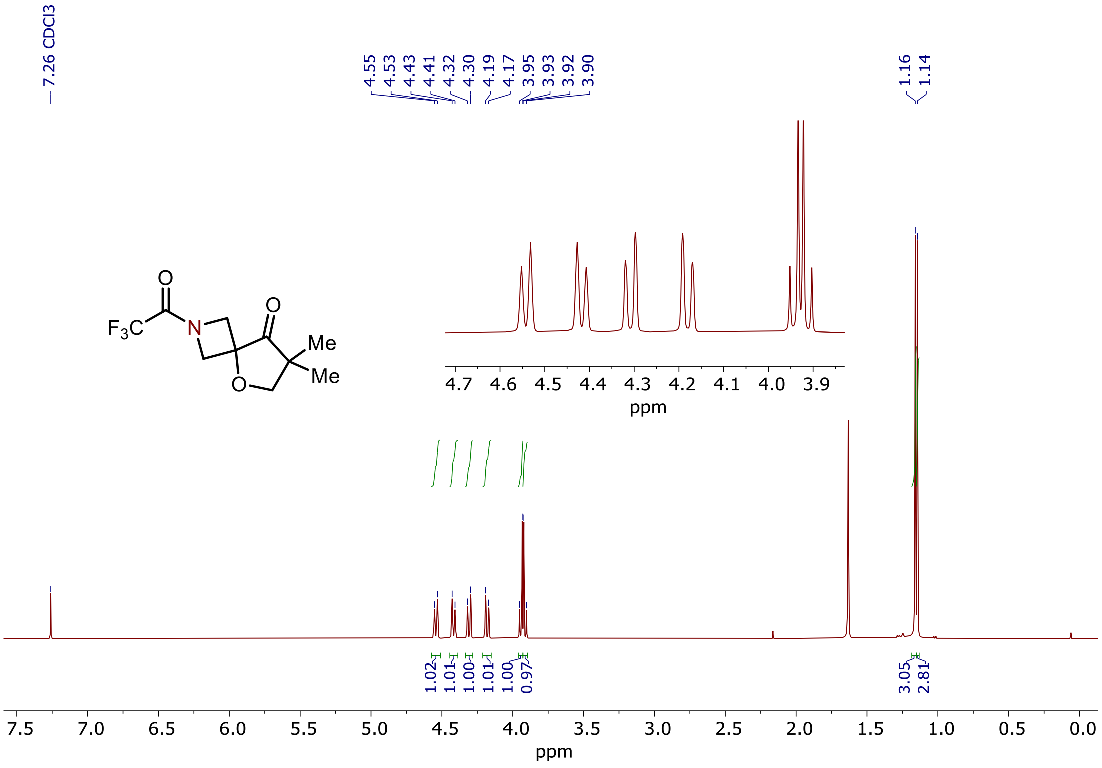

^{13}C NMR (126 MHz, $CDCl_3$) of **7,7-Dimethyl-2-(2,2,2-trifluoroacetyl)-5-oxa-2-azaspiro[3.4]octan-8-one: 4f**

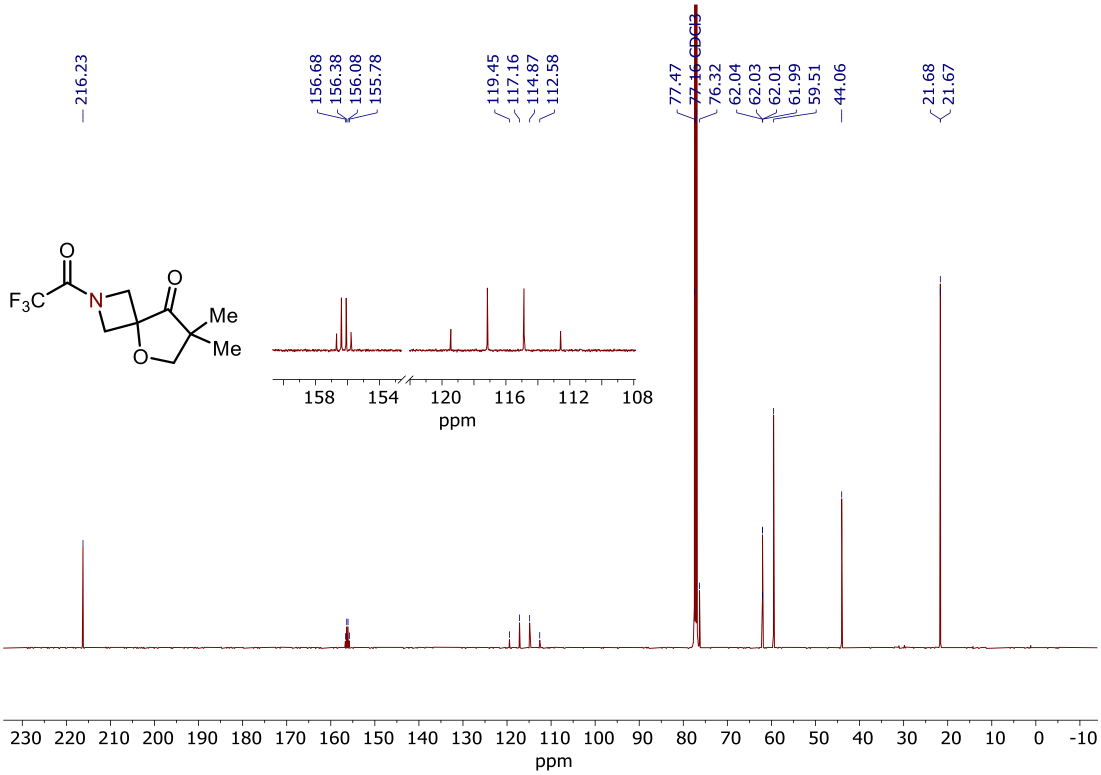

^{19}F NMR (377 MHz, $CDCl_3$) of **7,7-Dimethyl-2-(2,2,2-trifluoroacetyl)-5-oxa-2-azaspiro[3.4]octan-8-one: 4f**

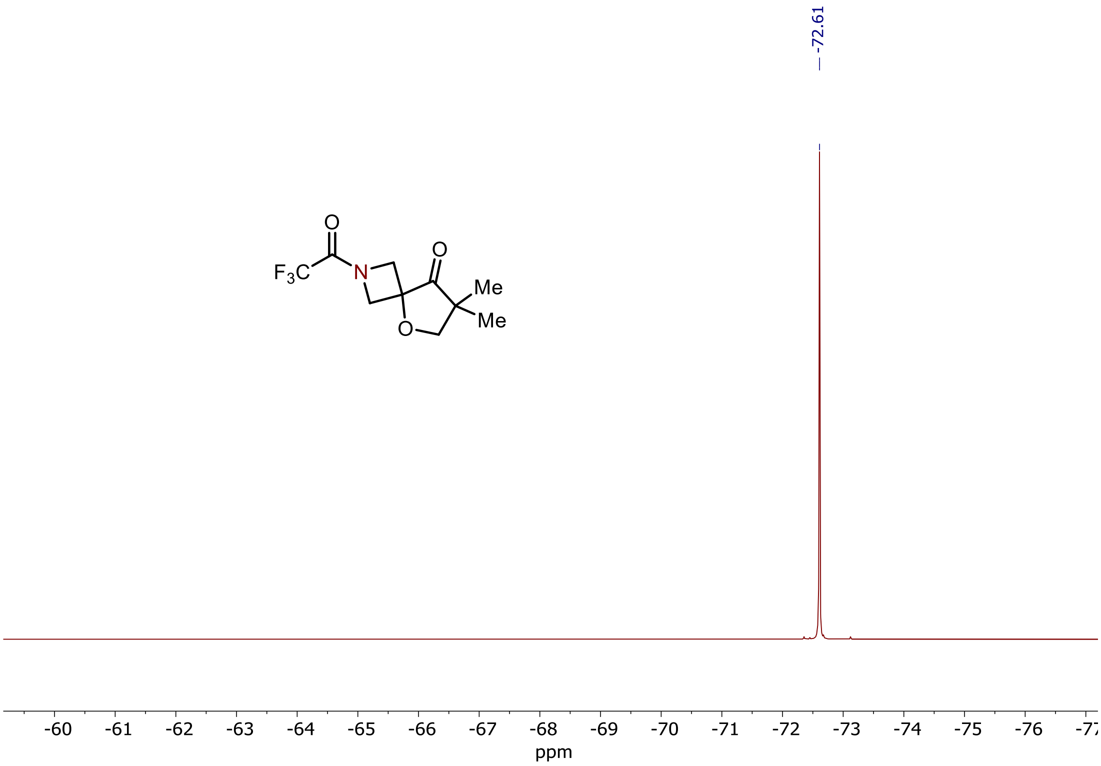

^{1}H NMR (400 MHz, $CDCl_3$) of **6-Methyl-2-(2,2,2-trifluoroacetyl)-5-oxa-2-azaspiro[3.4]octan-8-one: 4g** (*see procedure*)

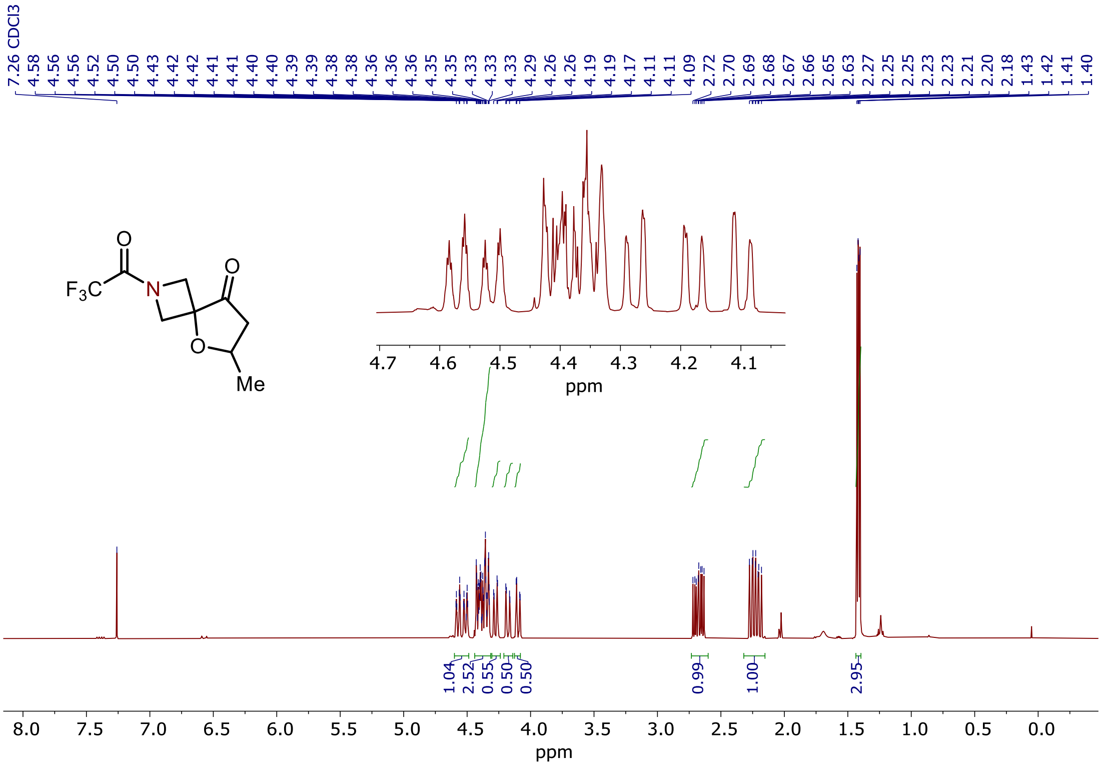

^{13}C NMR (126 MHz, $CDCl_3$) of **6-Methyl-2-(2,2,2-trifluoroacetyl)-5-oxa-2-azaspiro[3.4]octan-8-one: 4g**

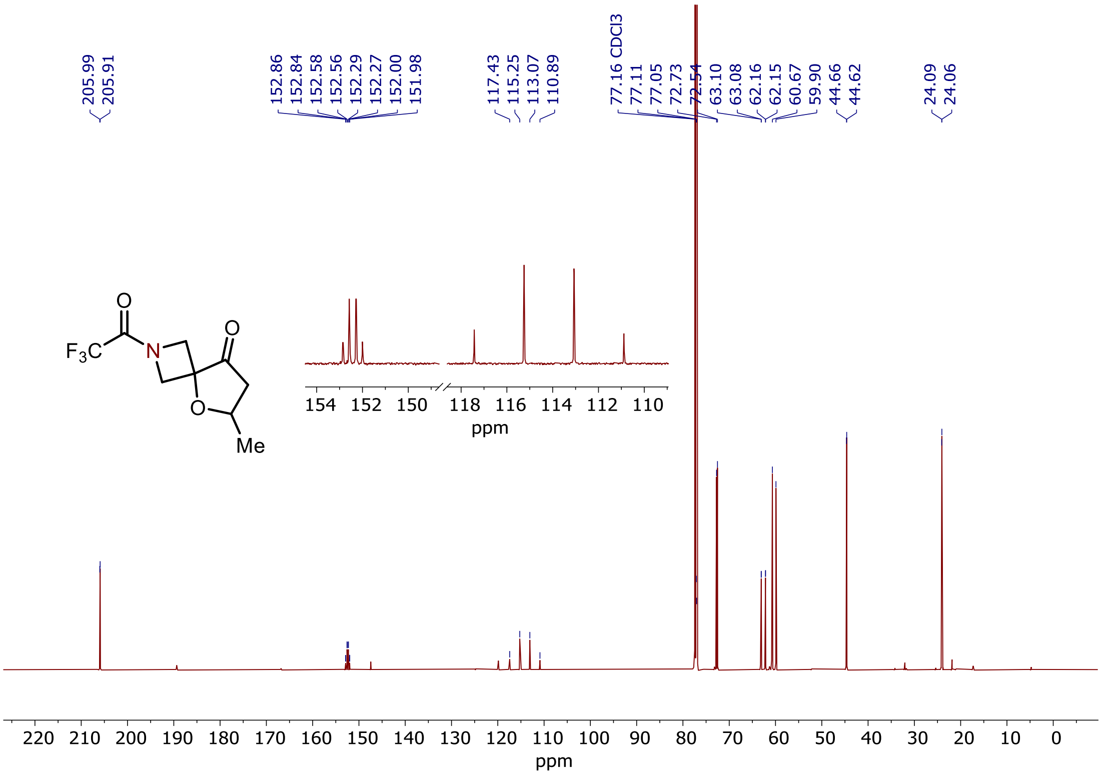

^{19}F NMR (377 MHz, $CDCl_3$) of **6-Methyl-2-(2,2,2-trifluoroacetyl)-5-oxa-2-azaspiro[3.4]octan-8-one: 4g**

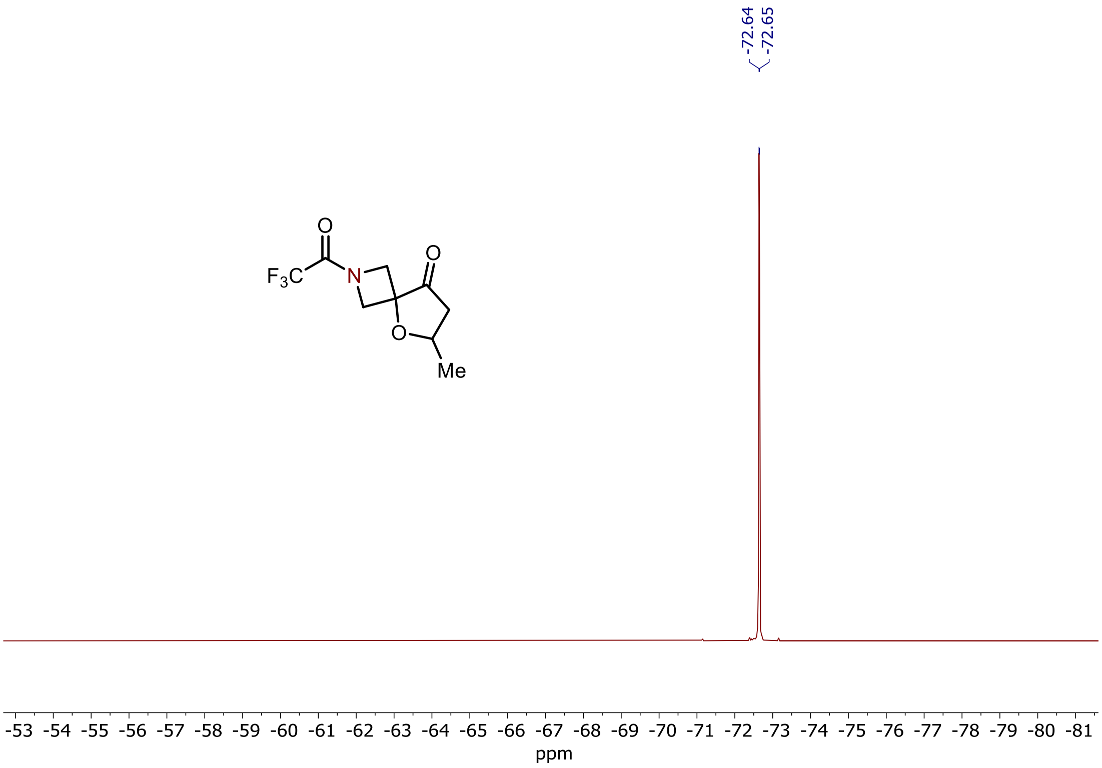

^{1}H NMR (400 MHz, $CDCl_3$) of **6-Cyclopropyl-2-(2,2,2-trifluoroacetyl)-5-oxa-2-azaspiro[3.4]octan-8-one: 4h** (*see procedure*)

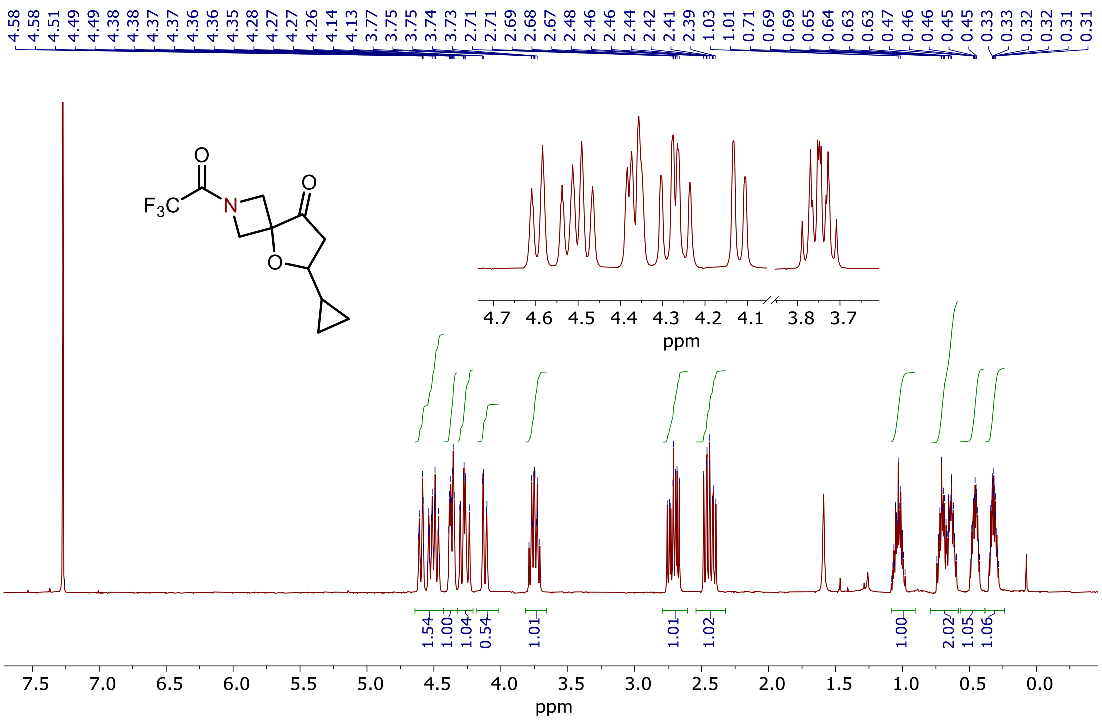

^{13}C NMR (126 MHz, $CDCl_3$) of **6-Cyclopropyl-2-(2,2,2-trifluoroacetyl)-5-oxa-2-azaspiro[3.4]octan-8-one: 4h**

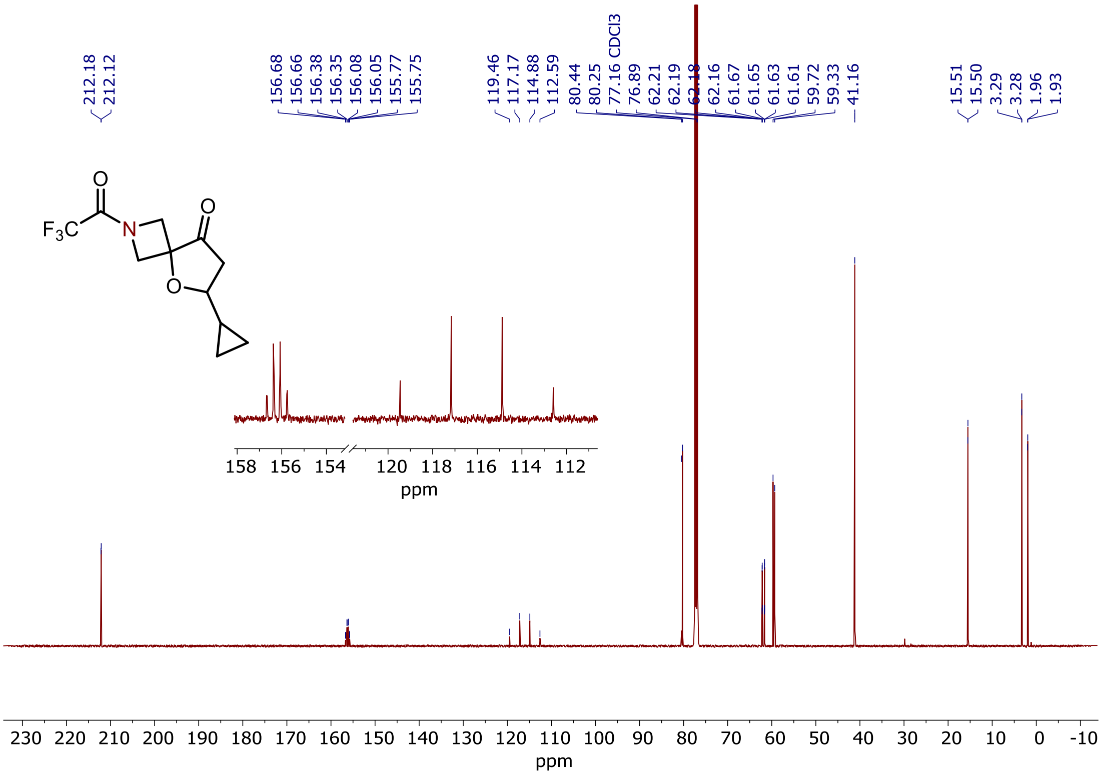

^{19}F NMR (377 MHz, $CDCl_3$) of **6-Cyclopropyl-2-(2,2,2-trifluoroacetyl)-5-oxa-2-azaspiro[3.4]octan-8-one: 4h**

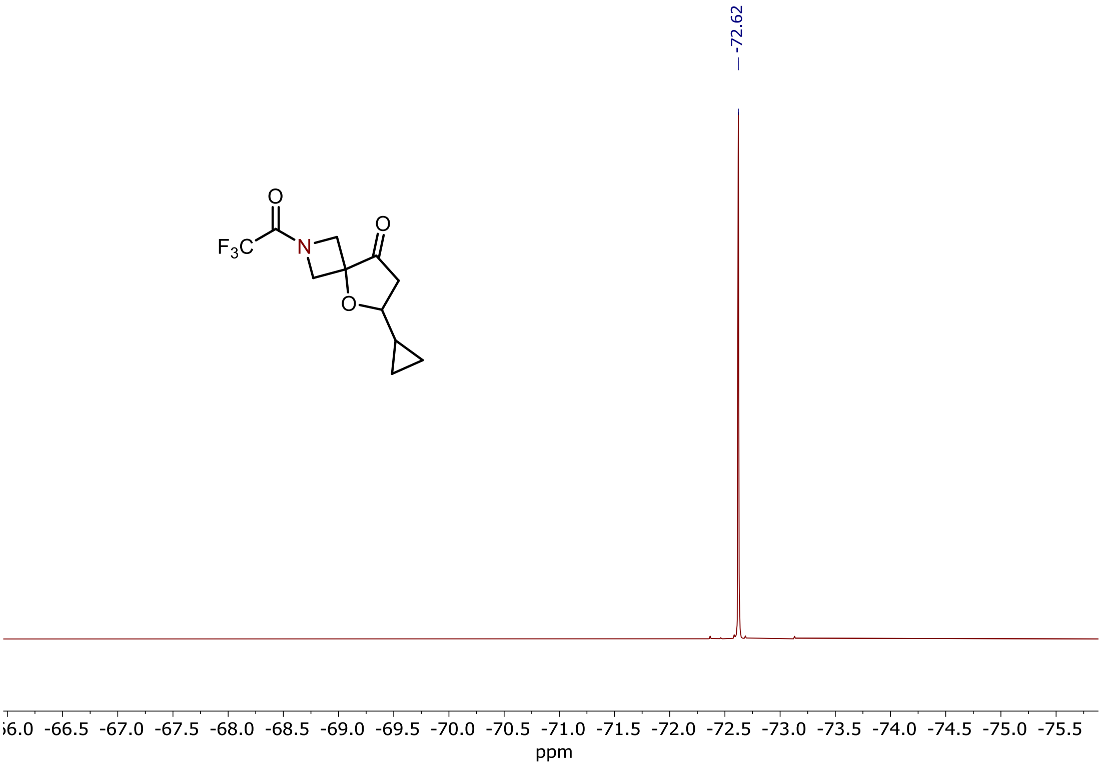

^{1}H NMR (500 MHz, $CDCl_3$) of **6-Isopropyl-2-(2,2,2-trifluoroacetyl)-5-oxa-2-azaspiro[3.4]octan-8-one: 4i** (*see procedure*)

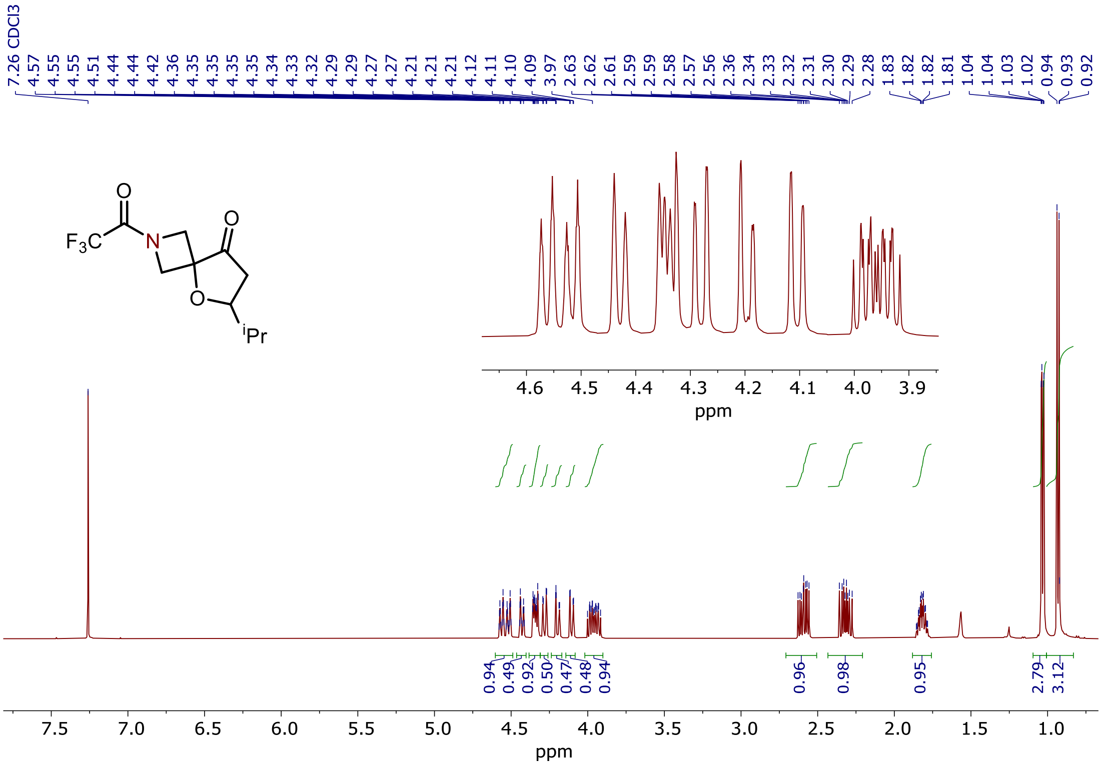

^{13}C NMR (126 MHz, $CDCl_3$) of **6-Isopropyl-2-(2,2,2-trifluoroacetyl)-5-oxa-2-azaspiro[3.4]octan-8-one: 4i**

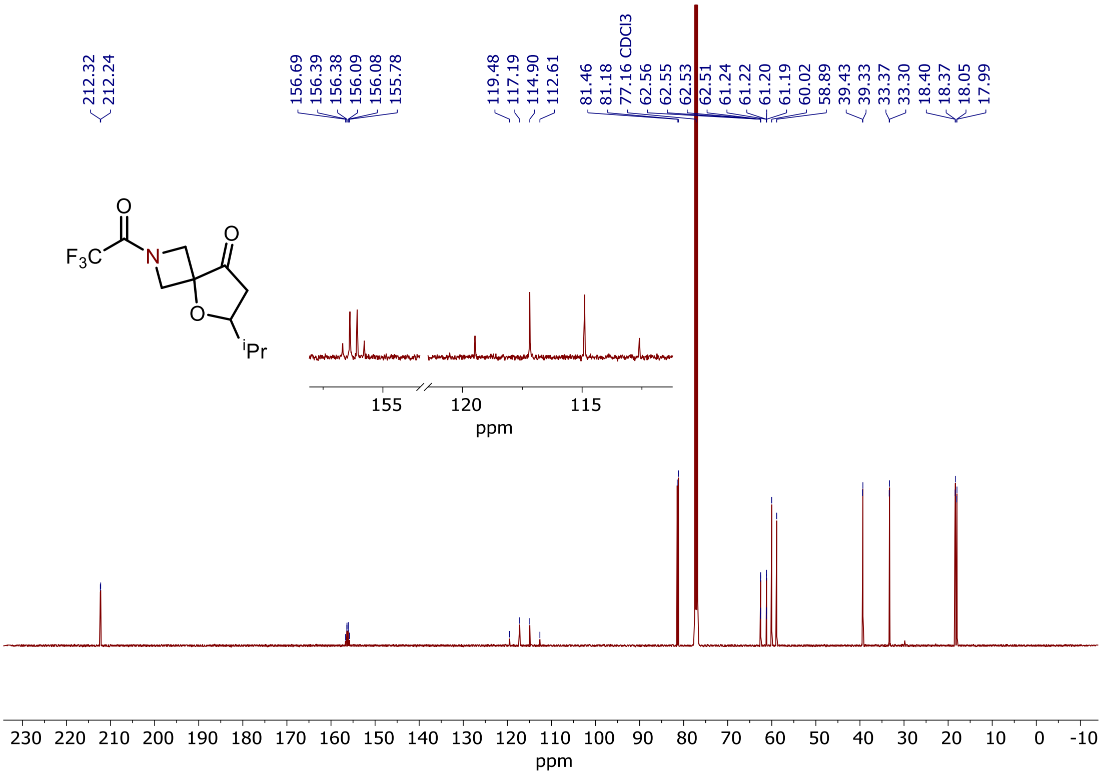

^{19}F NMR (377 MHz, $CDCl_3$) of **6-Isopropyl-2-(2,2,2-trifluoroacetyl)-5-oxa-2-azaspiro[3.4]octan-8-one: 4i**

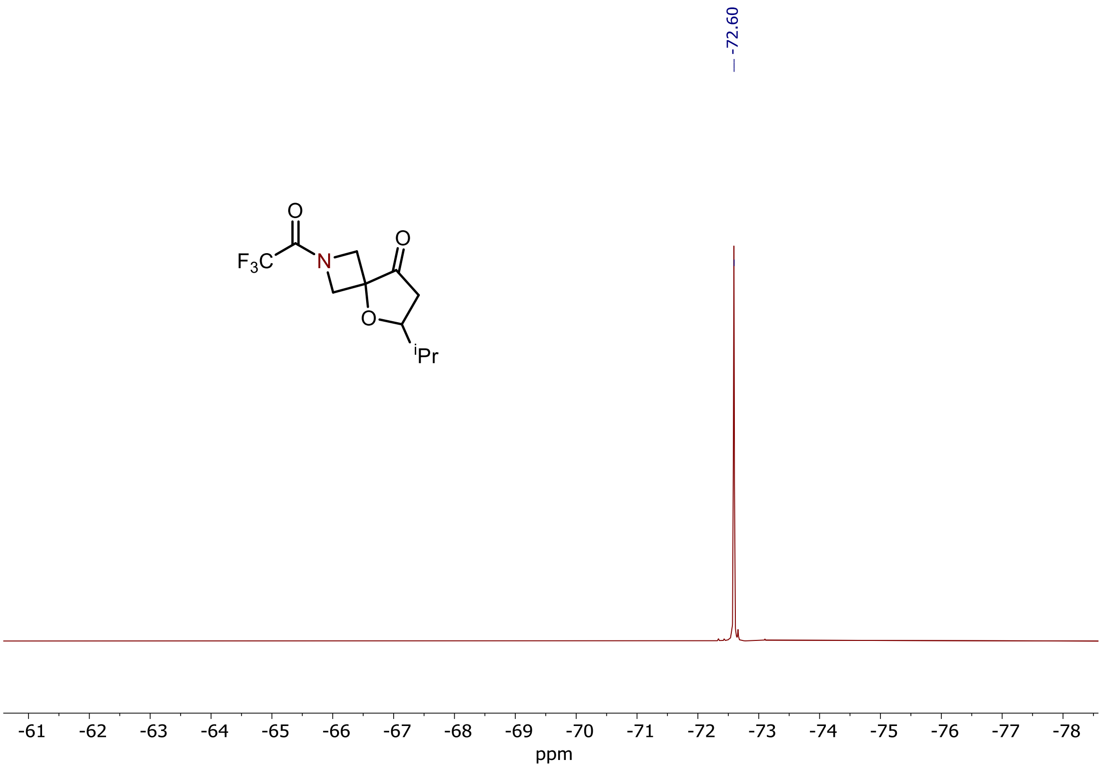

^{1}H NMR (400 MHz, $CDCl_3$) of **6-Phenyl-2-(2,2,2-trifluoroacetyl)-5-oxa-2-azaspiro[3.4]octan-8-one: 4j** (*see procedure*)

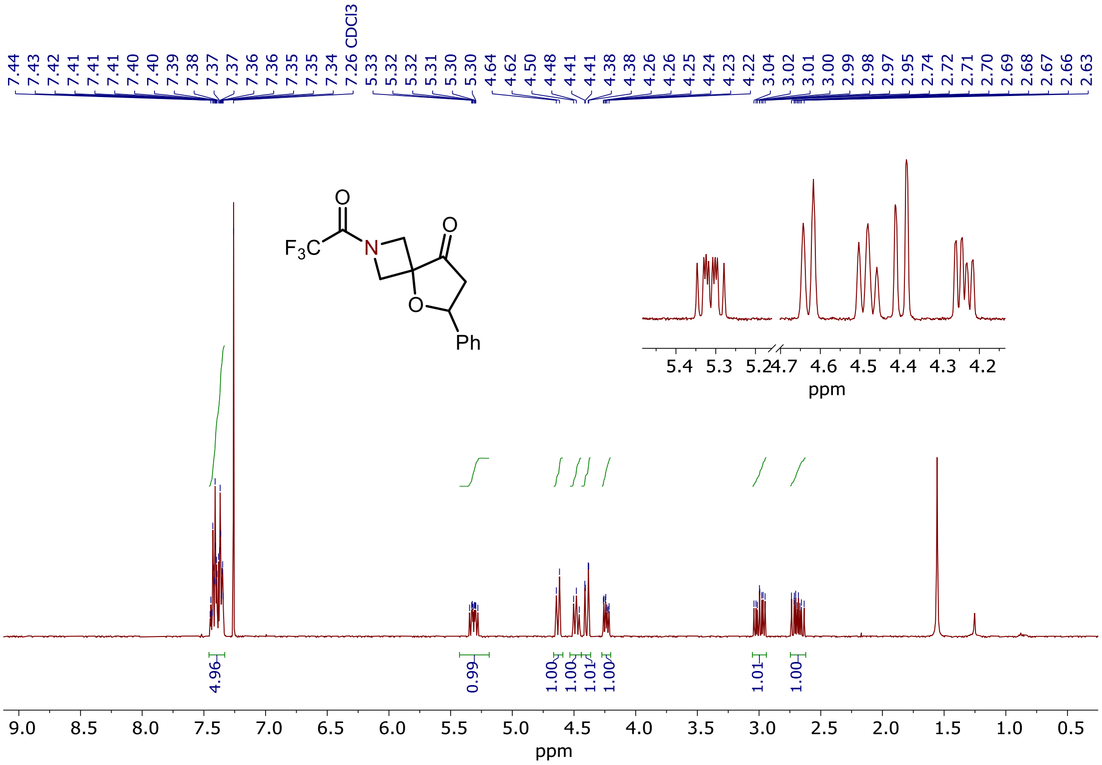

^{13}**C NMR** (126 MHz, $CDCl_3$) of **6-Phenyl-2-(2,2,2-trifluoroacetyl)-5-oxa-2-azaspiro[3.4]octan-8-one: 4j**

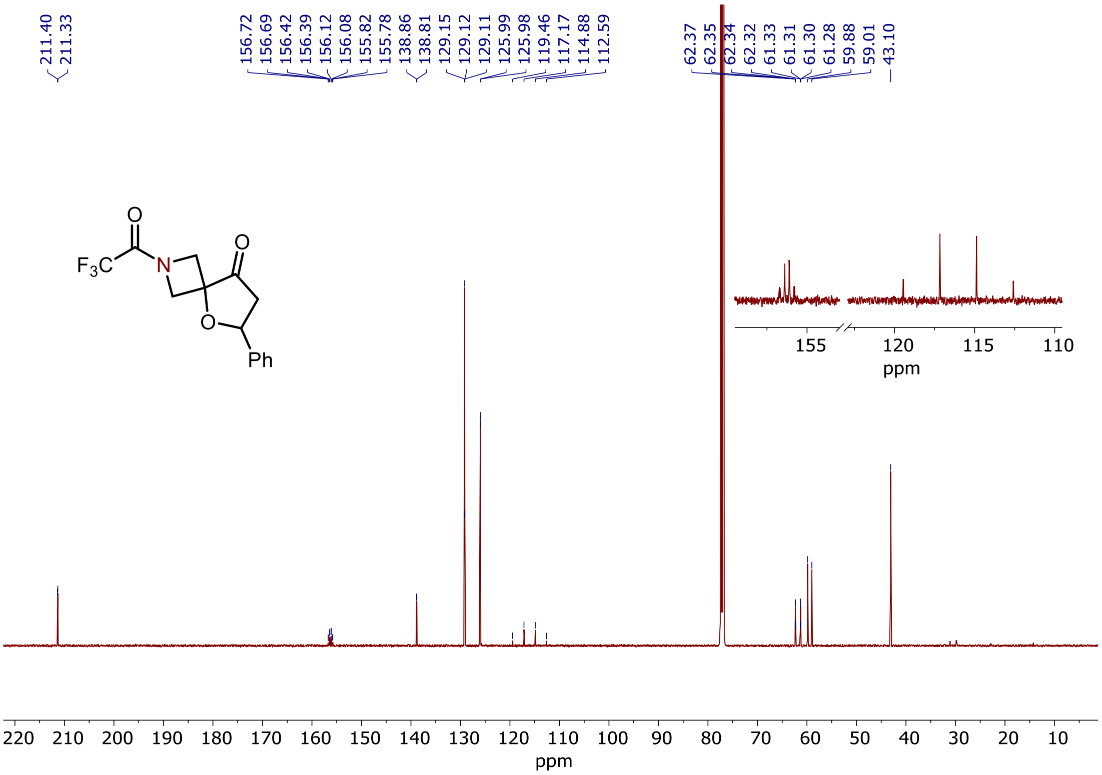

^{19}**F NMR** (377 MHz, $CDCl_3$) of **6-Phenyl-2-(2,2,2-trifluoroacetyl)-5-oxa-2-azaspiro[3.4]octan-8-one: 4j**

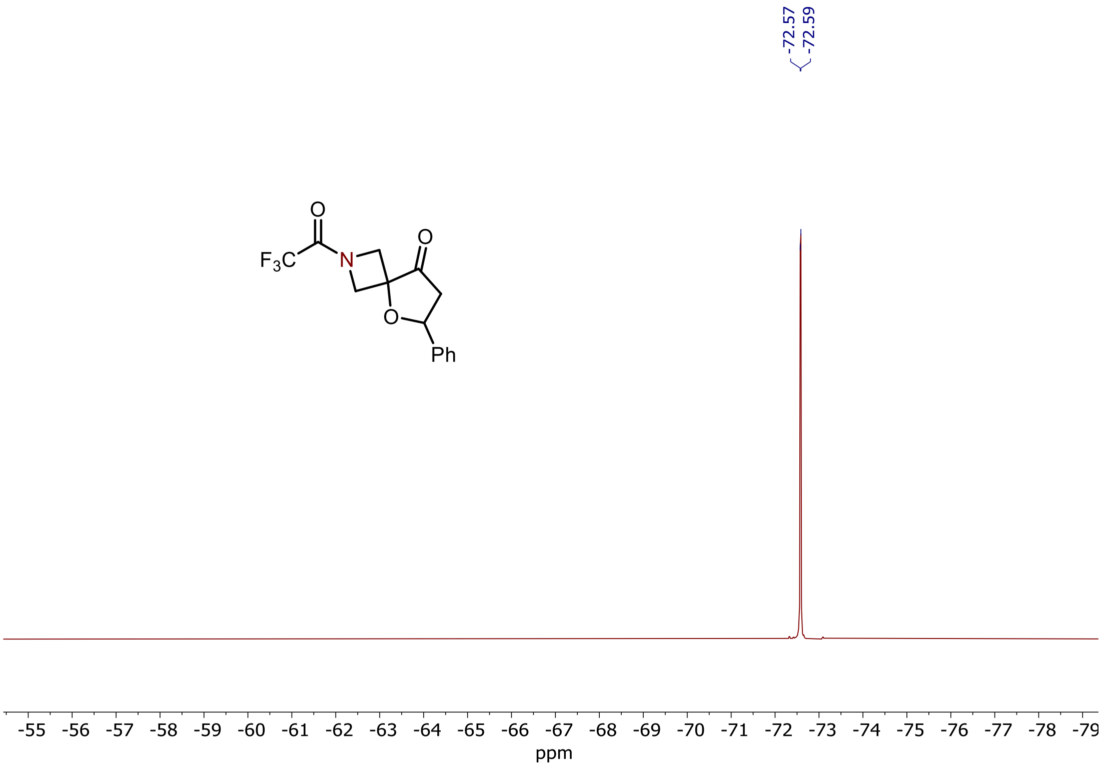

^{1}H NMR (400 MHz, CD_3OD) of **3-(8-Oxo-2-(2,2,2-trifluoroacetyl)-5-oxa-2-azaspiro[3.4]octan-6-yl)pyridin-1-ium 2,2,2-trifluoroacetate: 4k** (*see procedure*)

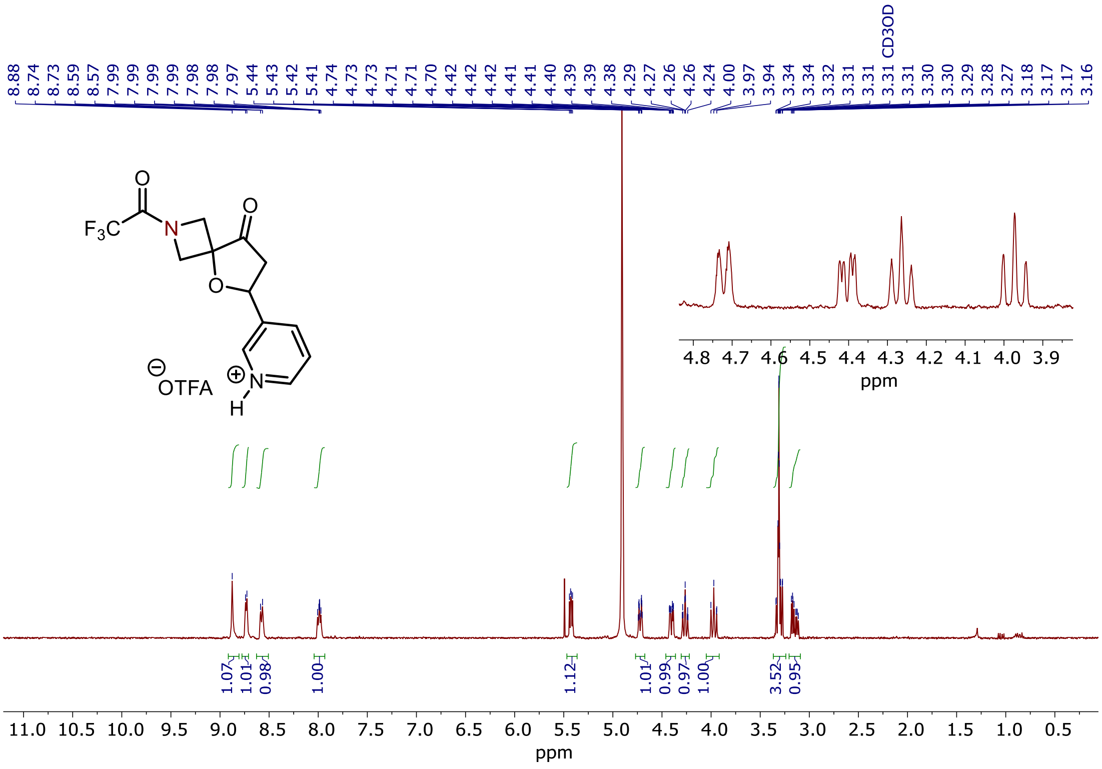

^{13}C NMR (126 MHz, CD_3OD) of **3-(8-Oxo-2-(2,2,2-trifluoroacetyl)-5-oxa-2-azaspiro[3.4]octan-6-yl)pyridin-1-ium 2,2,2-trifluoroacetate: 4k**

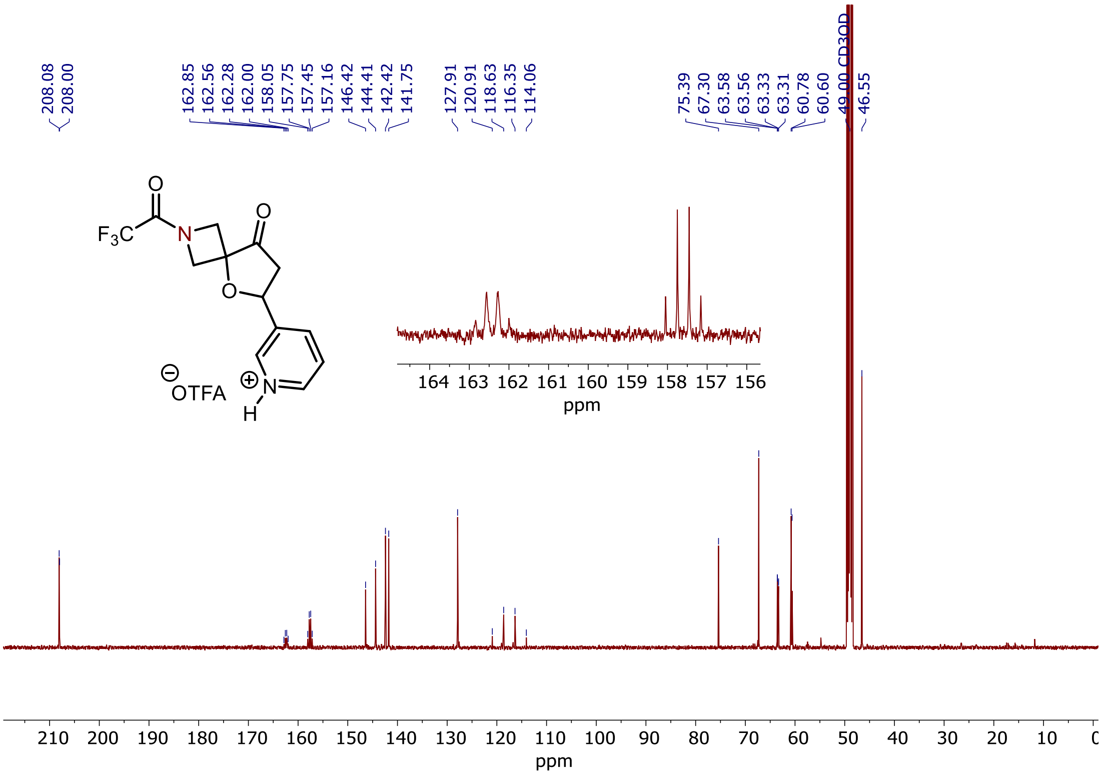

^{19}F NMR (377 MHz, CD_3OD) of **3-(8-Oxo-2-(2,2,2-trifluoroacetyl)-5-oxa-2-azaspiro[3.4]octan-6-yl)pyridin-1-ium 2,2,2-trifluoroacetate: 4k**

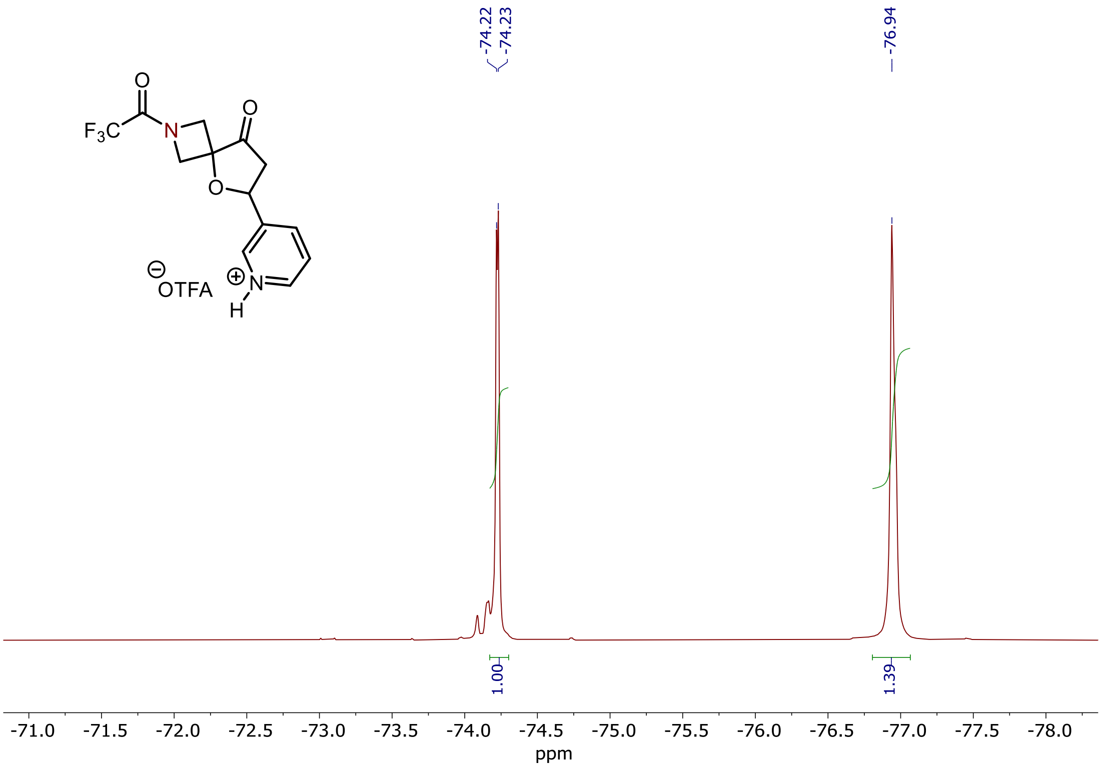

^{1}H NMR (400 MHz, $CDCl_3$) of **2-(2,2,2-Trifluoroacetyl)-6-vinyl-5-oxa-2-azaspiro[3.4]octan-8-one: 4l** (*see procedure*)

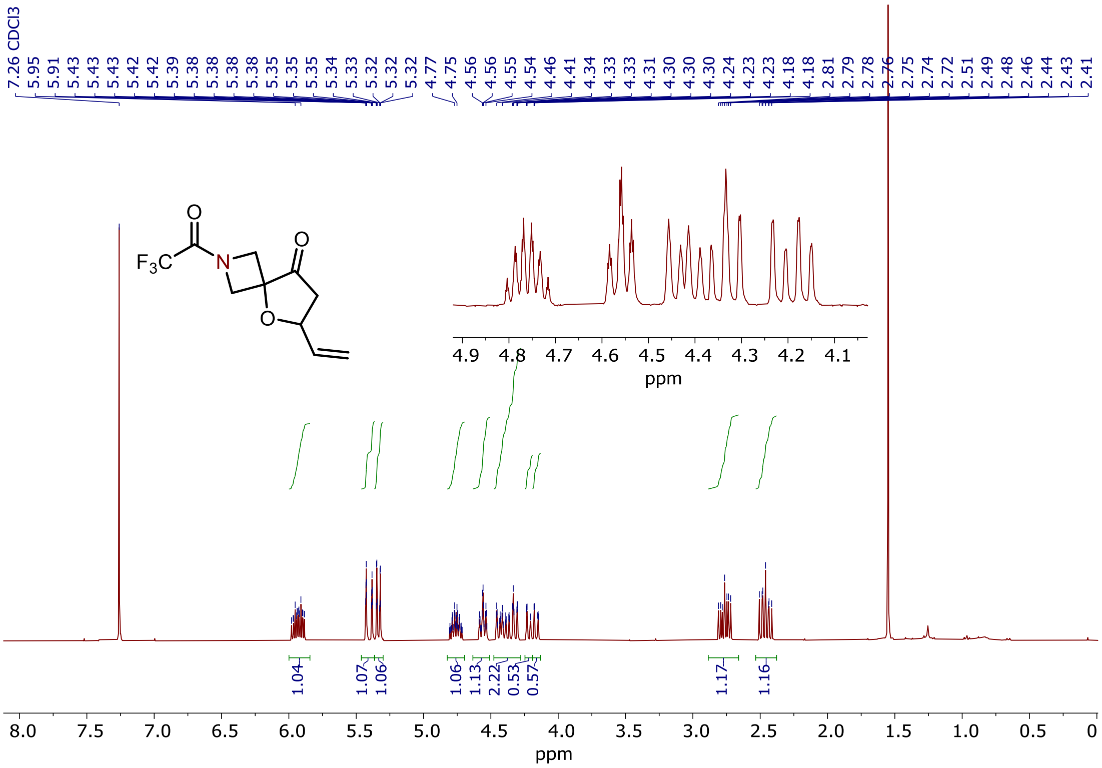

^{13}C NMR (126 MHz, $CDCl_3$) of **2-(2,2,2-Trifluoroacetyl)-6-vinyl-5-oxa-2-azaspiro[3.4]octan-8-one: 4l**

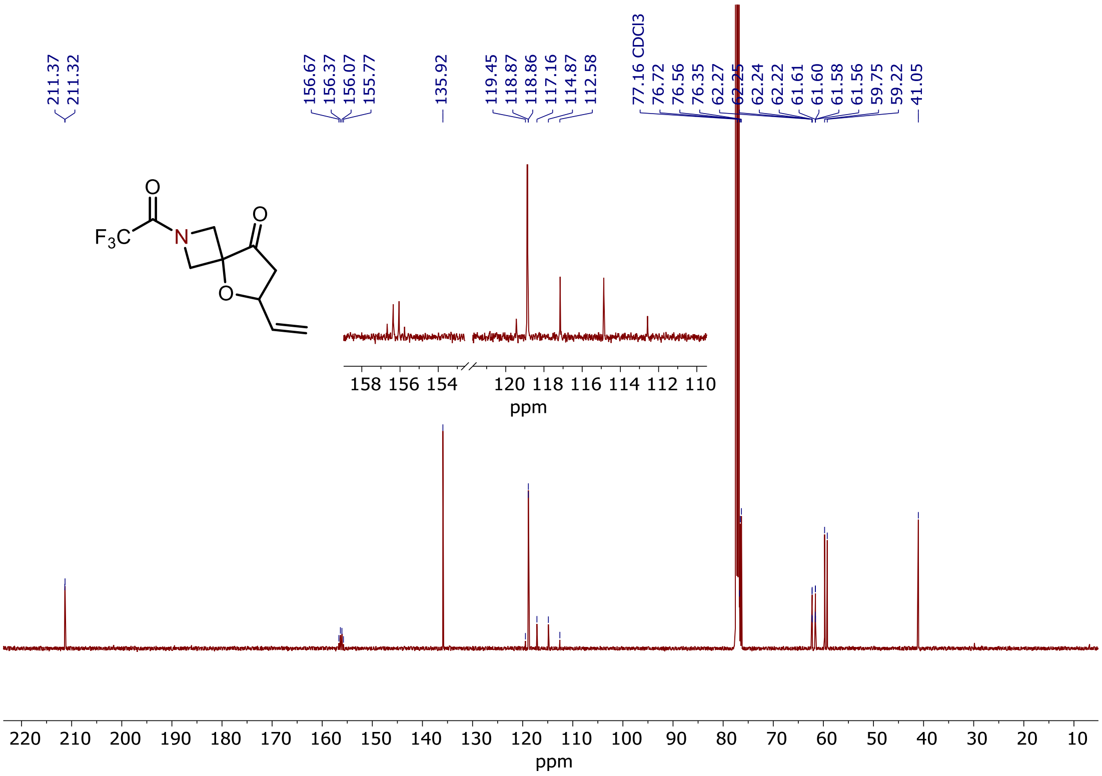

^{19}F NMR (377 MHz, $CDCl_3$) of **2-(2,2,2-Trifluoroacetyl)-6-vinyl-5-oxa-2-azaspiro[3.4]octan-8-one: 4l**

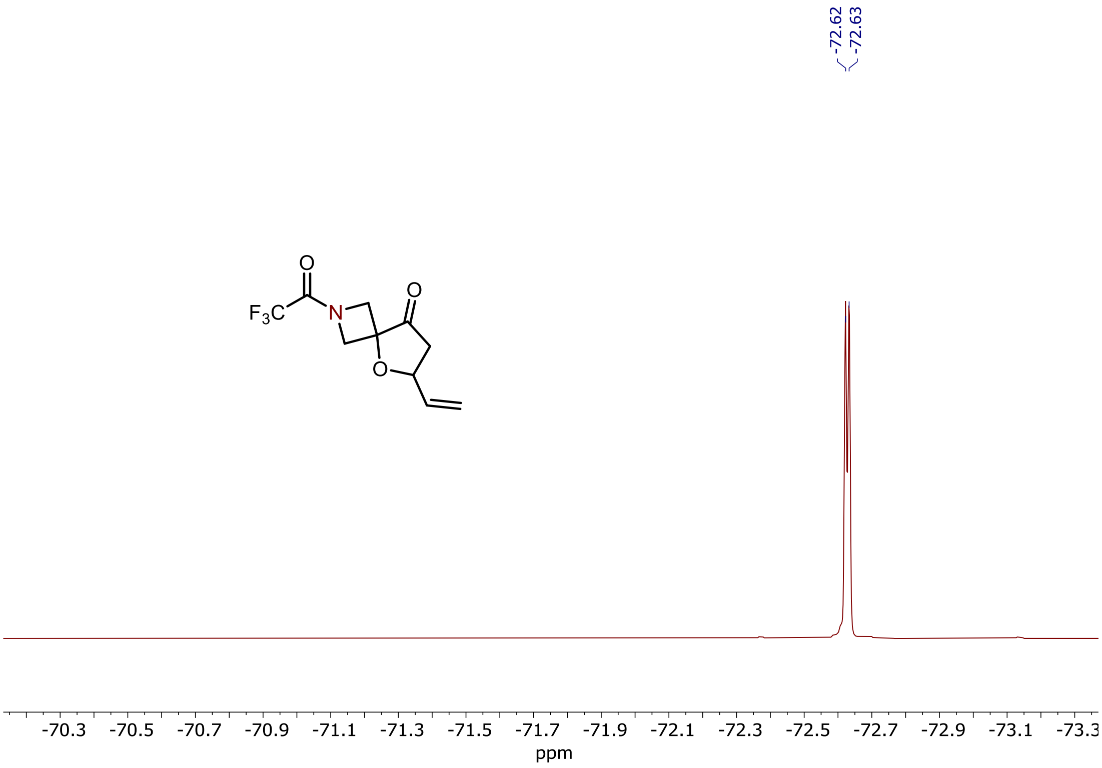

[1]**H NMR** (400 MHz, $CDCl_3$) of **6-(Chloromethyl)-2-(2,2,2-trifluoroacetyl)-5-oxa-2-azaspiro[3.4]octan-8-one: 4m** (*see procedure*)

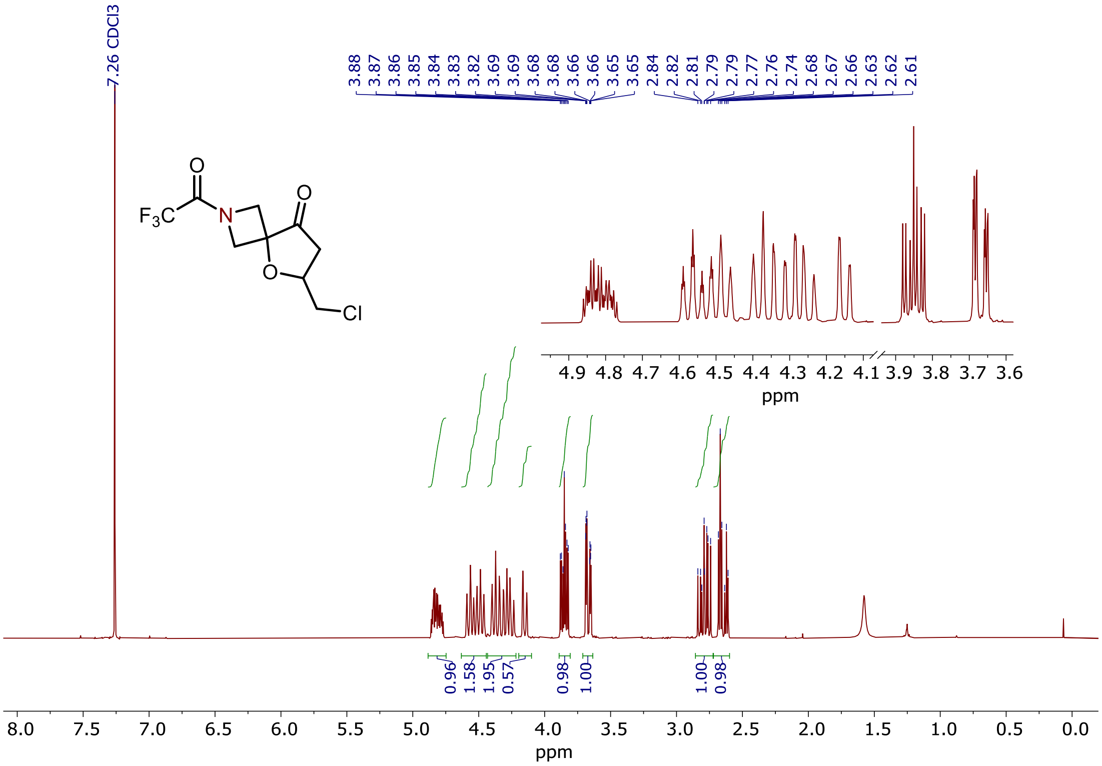

[13]**C NMR** (126 MHz, $CDCl_3$) of **6-(Chloromethyl)-2-(2,2,2-trifluoroacetyl)-5-oxa-2-azaspiro[3.4]octan-8-one: 4m**

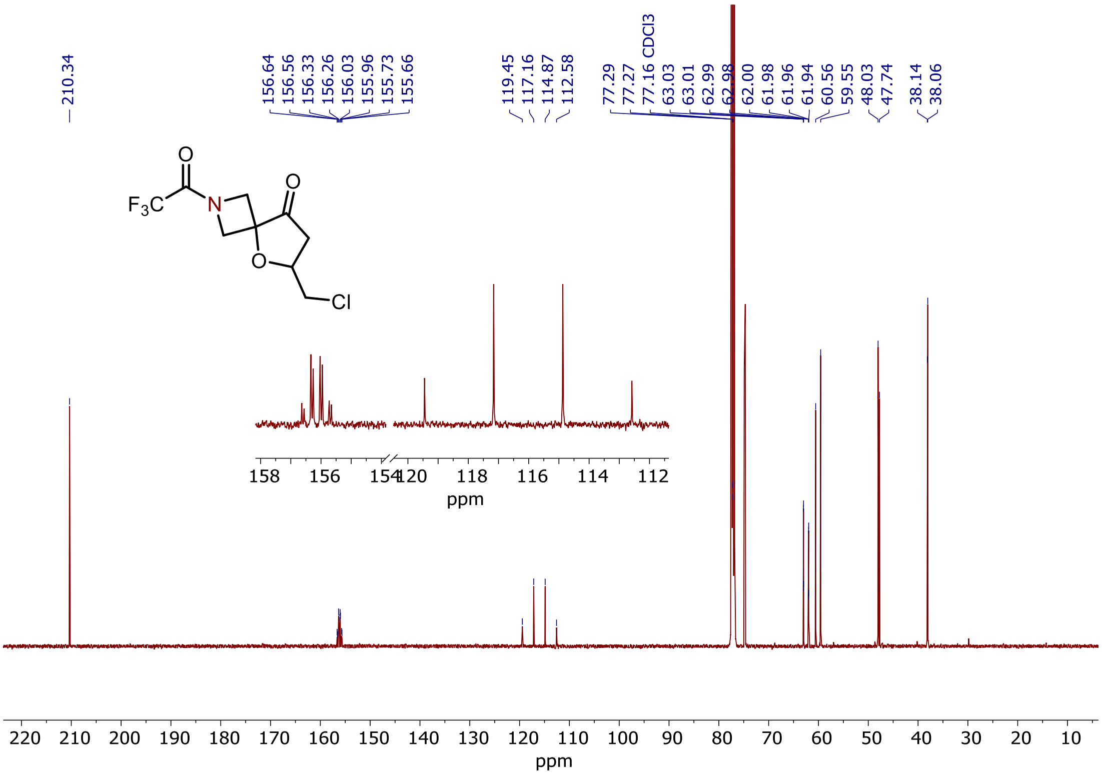

^{19}F NMR (377 MHz, $CDCl_3$) of **6-(Chloromethyl)-2-(2,2,2-trifluoroacetyl)-5-oxa-2-azaspiro[3.4]octan-8-one: 4m**

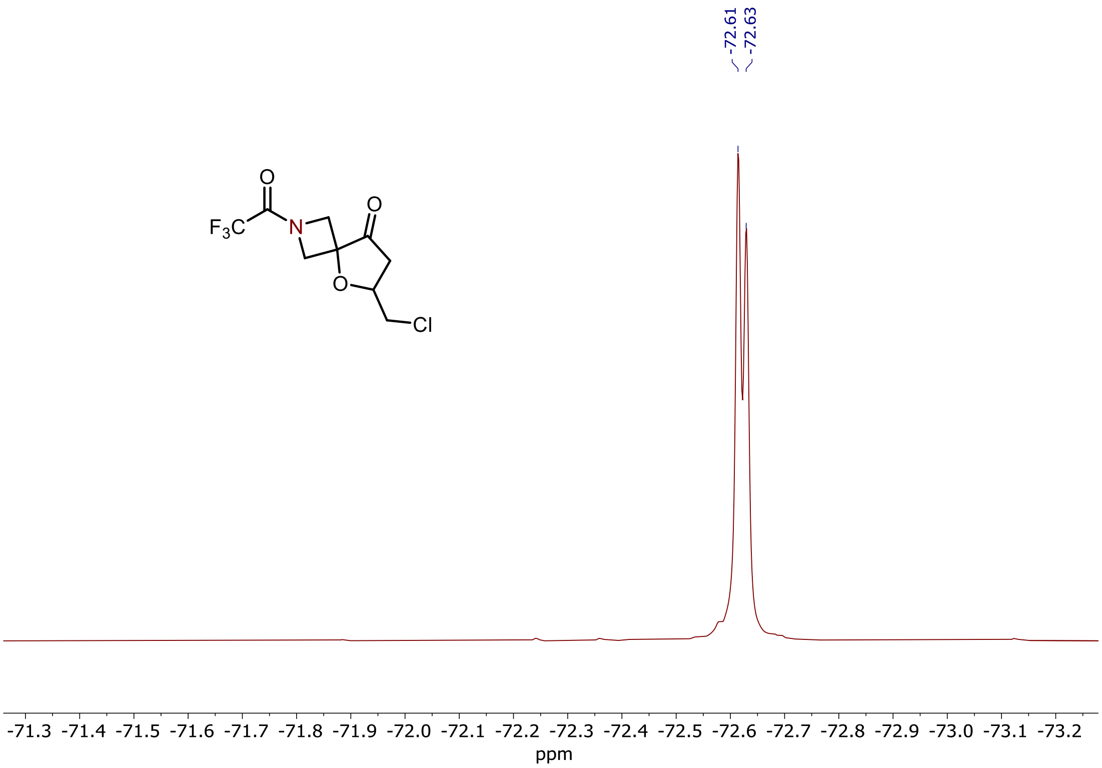

^{1}H NMR (500 MHz, $CDCl_3$) of **6-((Benzyloxy)methyl)-2-(2,2,2-trifluoroacetyl)-5-oxa-2-azaspiro[3.4]octan-8-one: 4n** (*see procedure*)

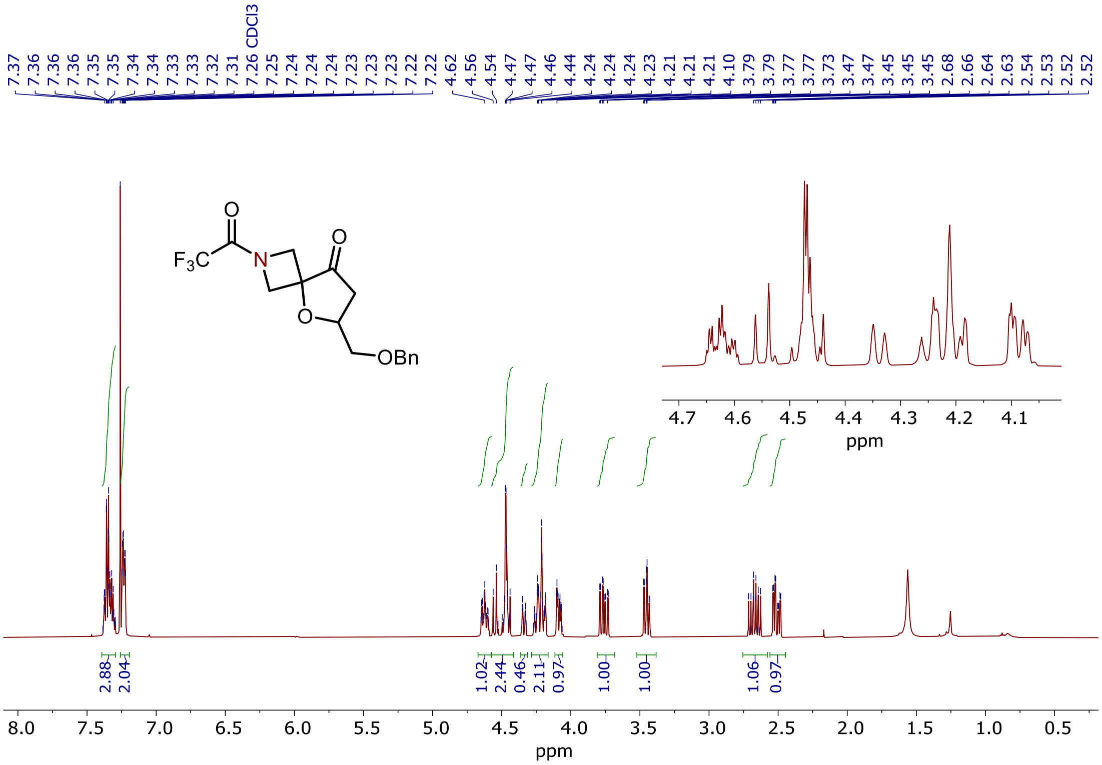

^{13}C NMR (126 MHz, $CDCl_3$) of **6-((Benzyloxy)methyl)-2-(2,2,2-trifluoroacetyl)-5-oxa-2-azaspiro[3.4]octan-8-one: 4n**

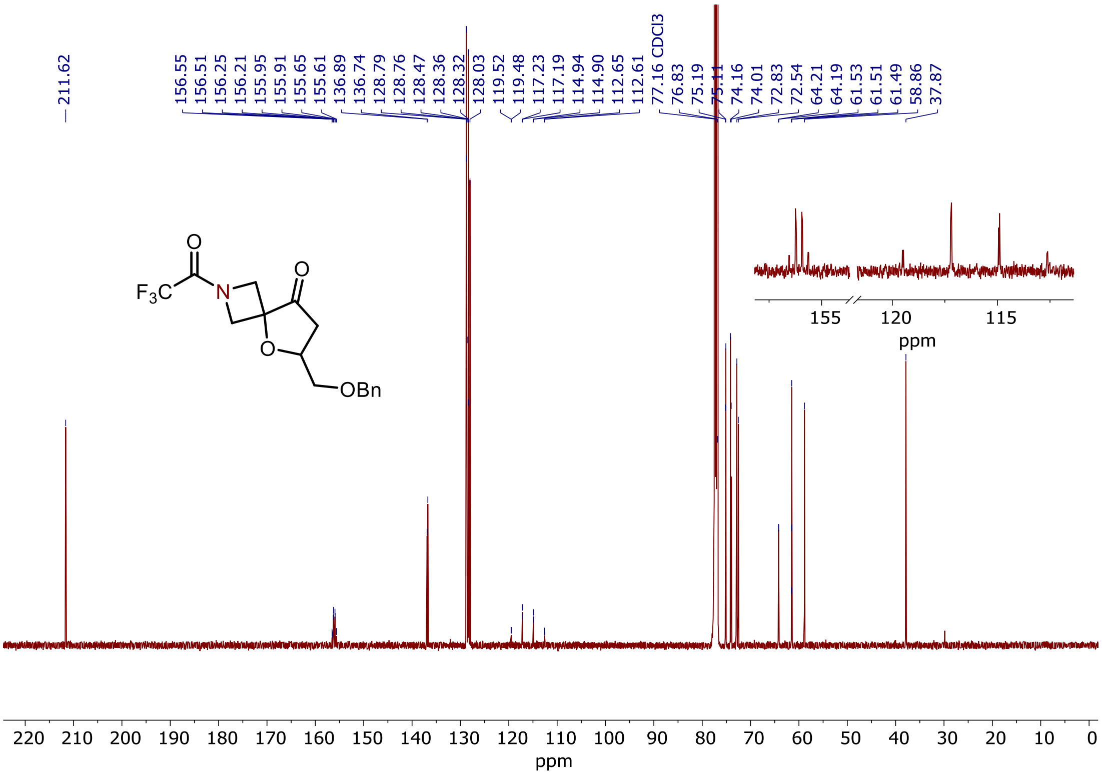

^{19}F NMR (377 MHz, $CDCl_3$) of **6-((Benzyloxy)methyl)-2-(2,2,2-trifluoroacetyl)-5-oxa-2-azaspiro[3.4]octan-8-one: 4n**

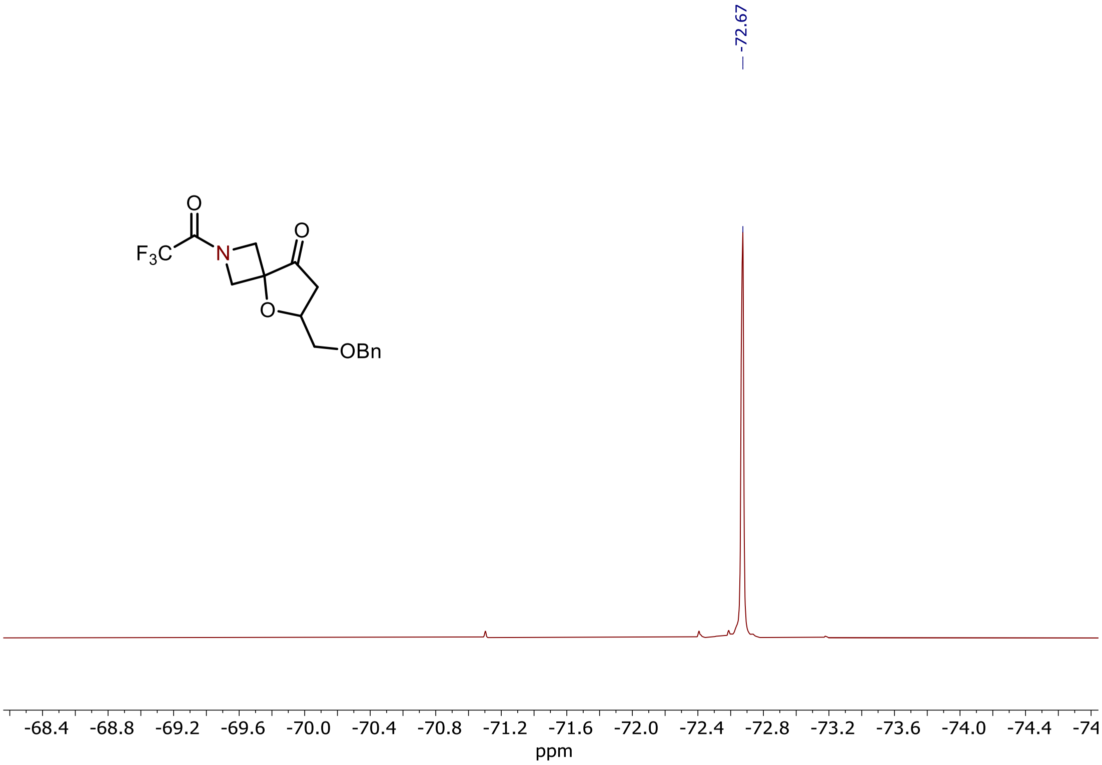

^{1}H NMR (400 MHz, $CDCl_3$) of **6,6-Dimethyl-2-(2,2,2-trifluoroacetyl)-5-oxa-2-azaspiro[3.4]octan-8-one: 4o** (*see procedure*)

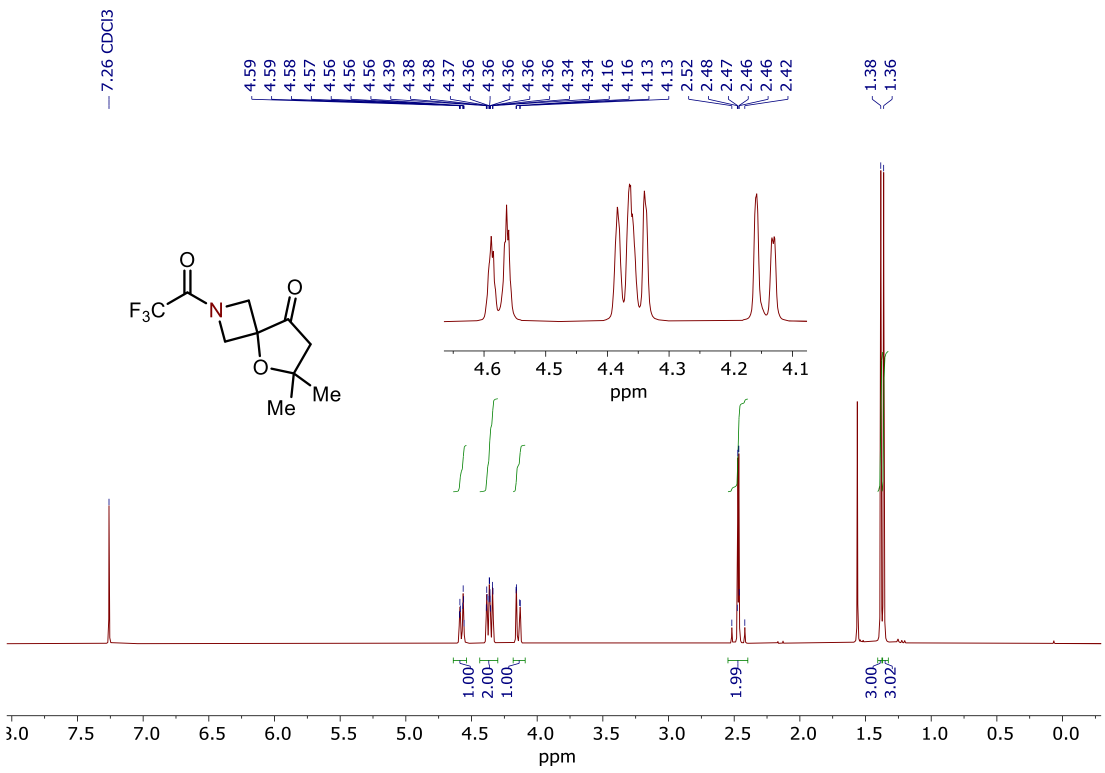

^{13}C NMR (126 MHz, $CDCl_3$) of **6,6-Dimethyl-2-(2,2,2-trifluoroacetyl)-5-oxa-2-azaspiro[3.4]octan-8-one: 4o**

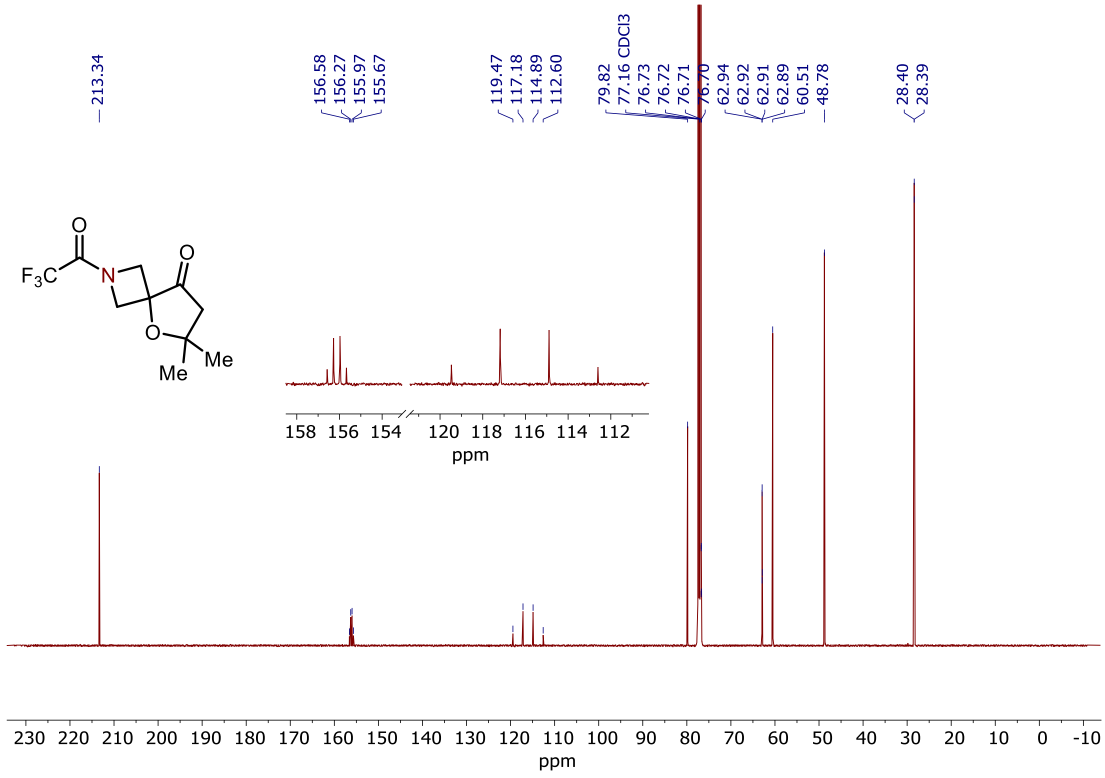

^{19}F NMR (377 MHz, $CDCl_3$) of **6,6-Dimethyl-2-(2,2,2-trifluoroacetyl)-5-oxa-2-azaspiro[3.4]octan-8-one: 4o**

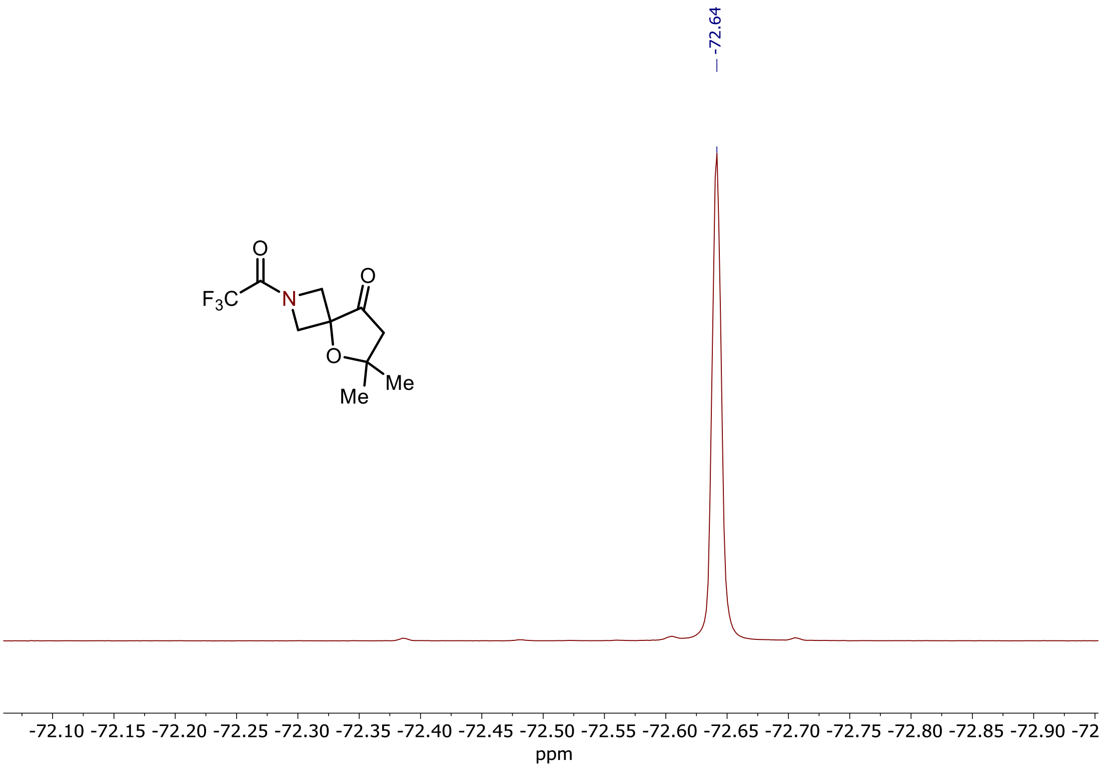

^{1}H NMR (400 MHz, $CDCl_3$) of **2-(2,2,2-Trifluoroacetyl)-5-oxa-2-azadispiro[3.1.5^6.2^4]tridecan-13-one: 4p** (*see procedure*)

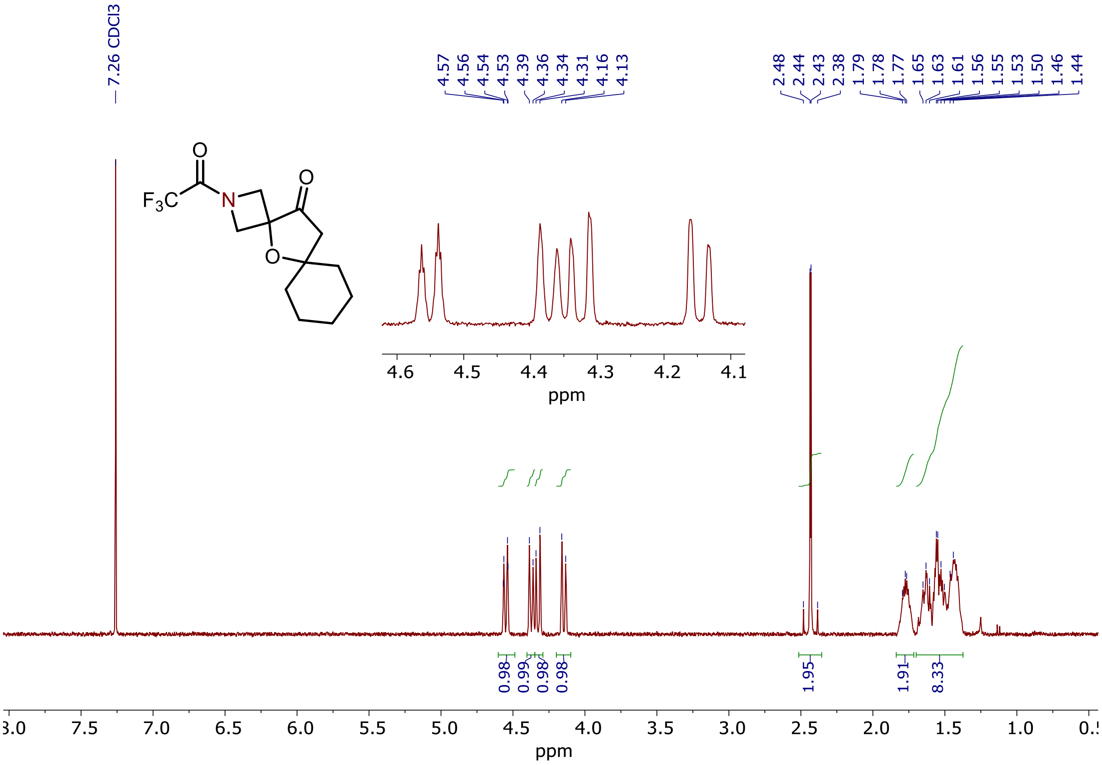

^{13}C NMR (126 MHz, $CDCl_3$) of **2-(2,2,2-Trifluoroacetyl)-5-oxa-2-azadispiro[3.1.5^6.2^4]tridecan-13-one: 4p**

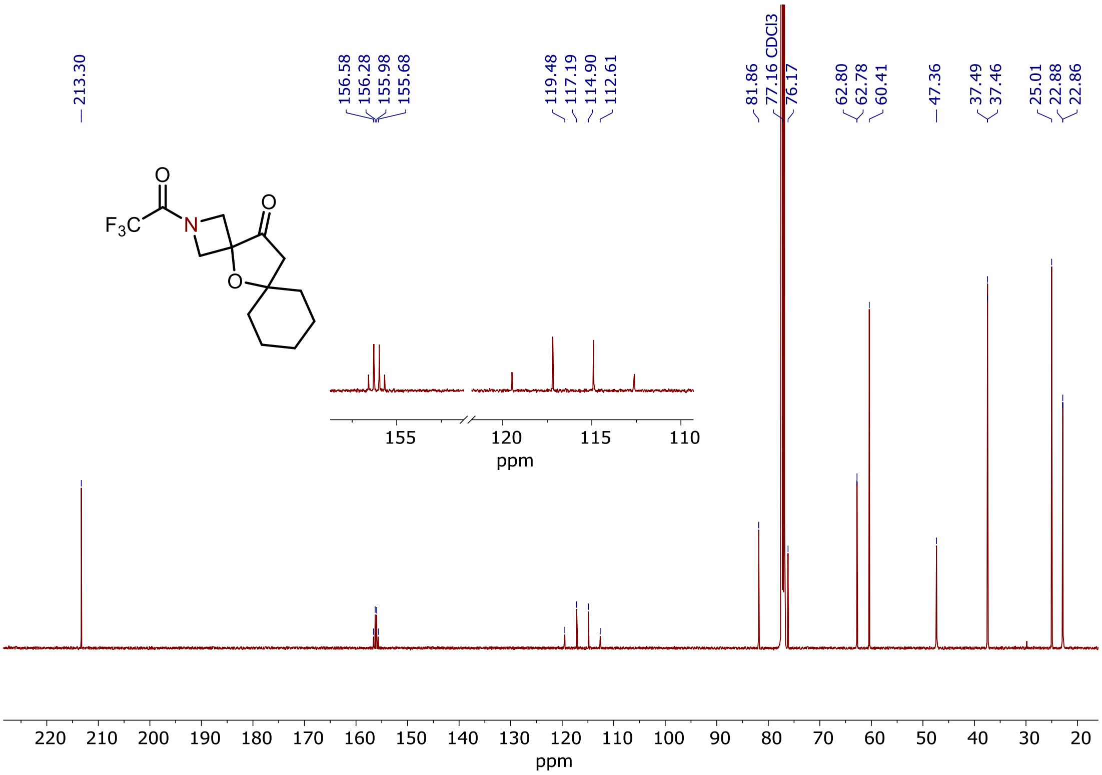

^{19}F NMR (377 MHz, $CDCl_3$) of **2-(2,2,2-Trifluoroacetyl)-5-oxa-2-azadispiro[3.1.5^6.2^4]tridecan-13-one: 4p**

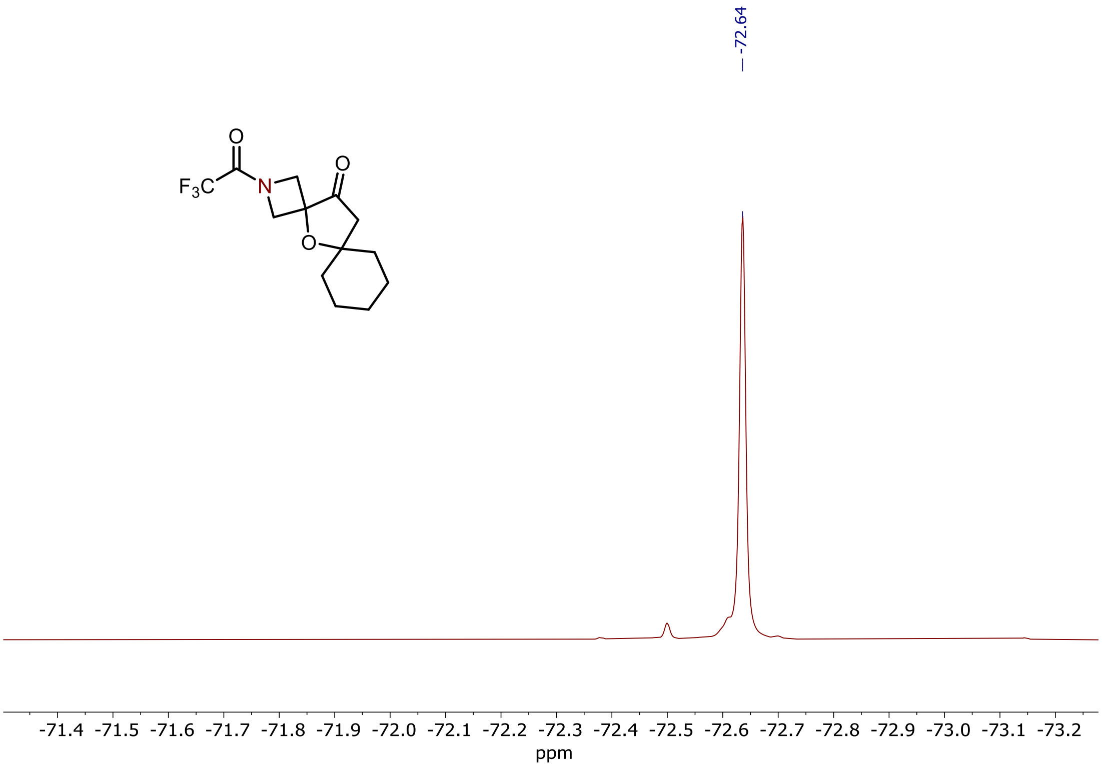

^{1}H NMR (400 MHz, $CDCl_3$) of **7-Methyl-2-(2,2,2-trifluoroacetyl)-6-vinyl-5-oxa-2-azaspiro[3.4]octan-8-one**: 96:4:*anti*:*syn*-**4q** (*anti*-**4q** peaks labelled; *see procedure*)

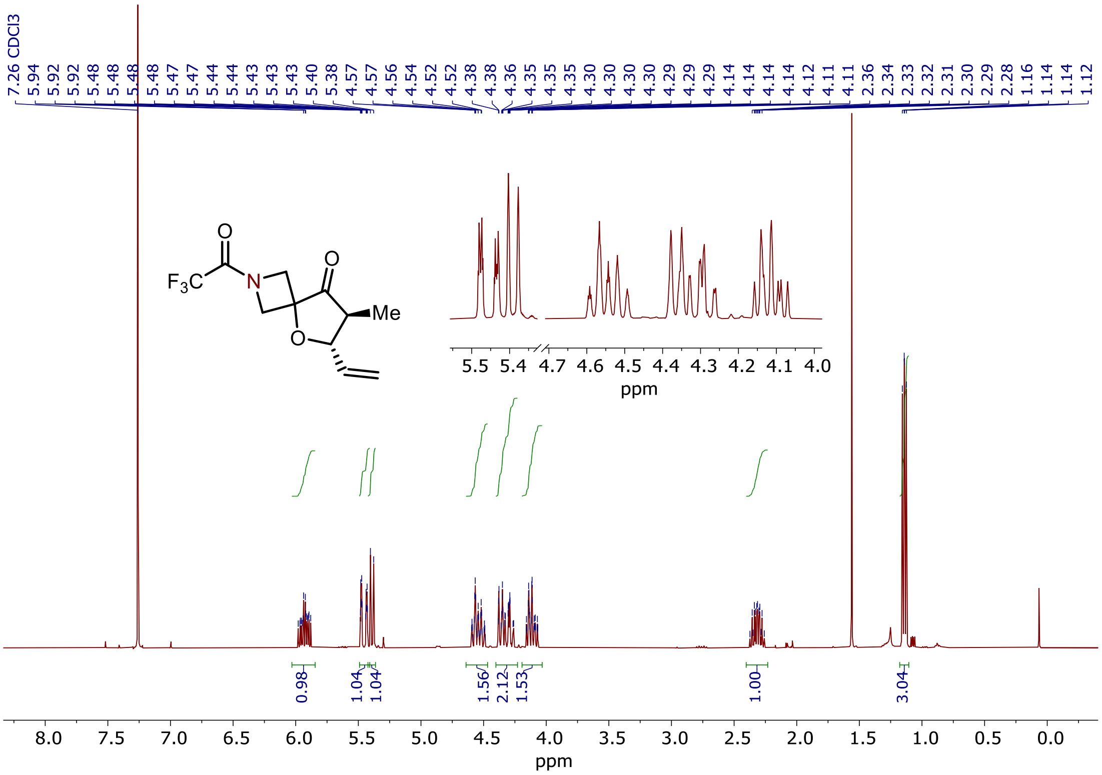

^{13}C NMR (126 MHz, $CDCl_3$) of **7-Methyl-2-(2,2,2-trifluoroacetyl)-6-vinyl-5-oxa-2-azaspiro[3.4]octan-8-one:** 96:4:*anti*:*syn*-**4q** (*anti*-**4q** peaks labelled)

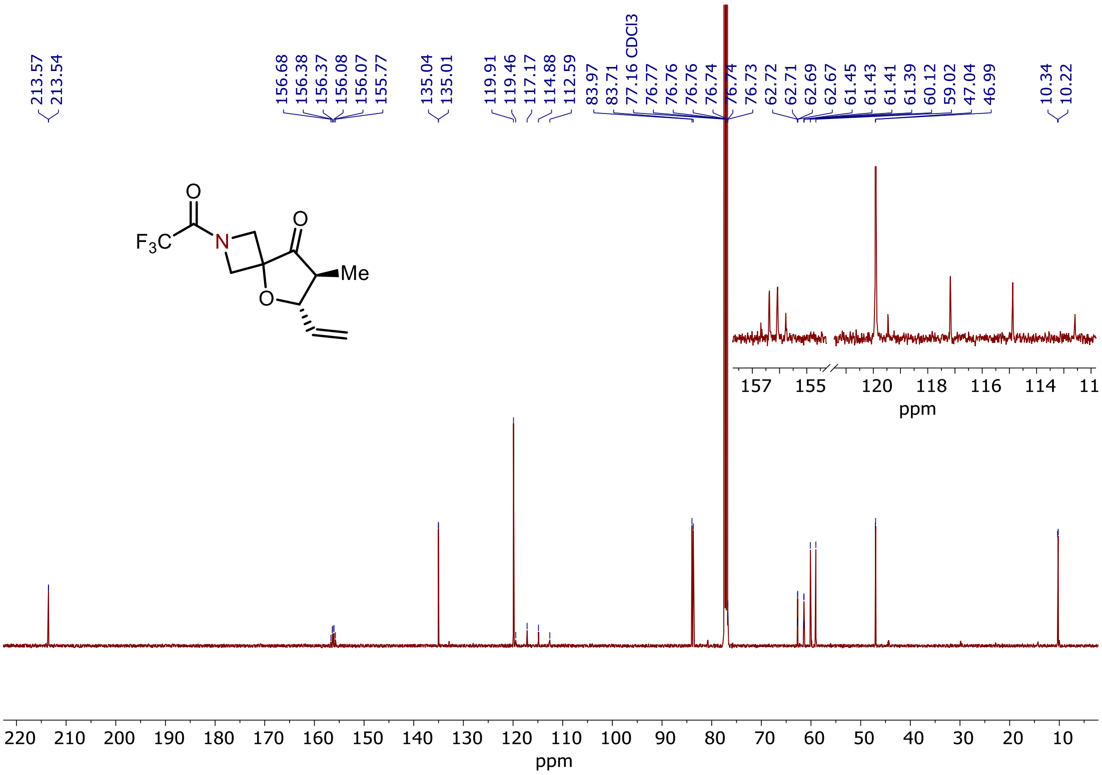

^{19}F NMR (377 MHz, $CDCl_3$) of **7-Methyl-2-(2,2,2-trifluoroacetyl)-6-vinyl-5-oxa-2-azaspiro[3.4]octan-8-one:** 96:4:*anti*:*syn*-**4q** (*anti*-**4q** peaks labelled)

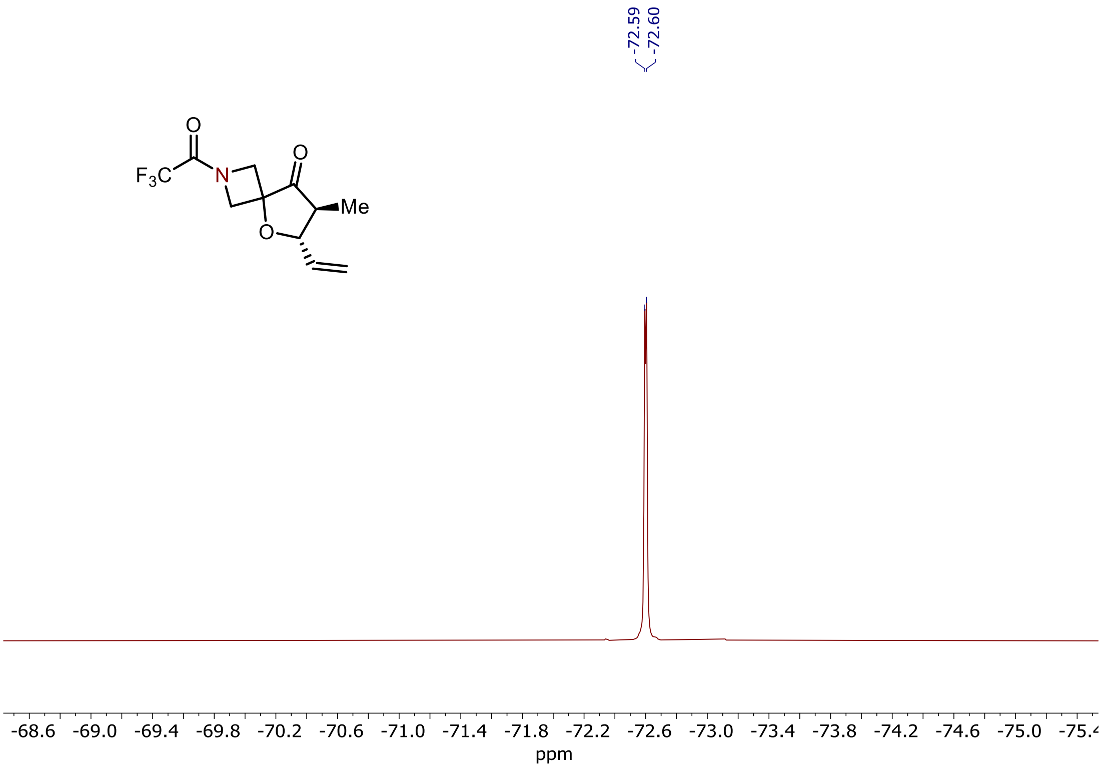

^{1}H NMR (400 MHz, $CDCl_3$) of **7-Methyl-2-(2,2,2-trifluoroacetyl)-6-vinyl-5-oxa-2-azaspiro[3.4]octan-8-one**: 17:83 *syn*:*anti*-**4q** (*syn*-**4q** peaks labelled; see procedure)

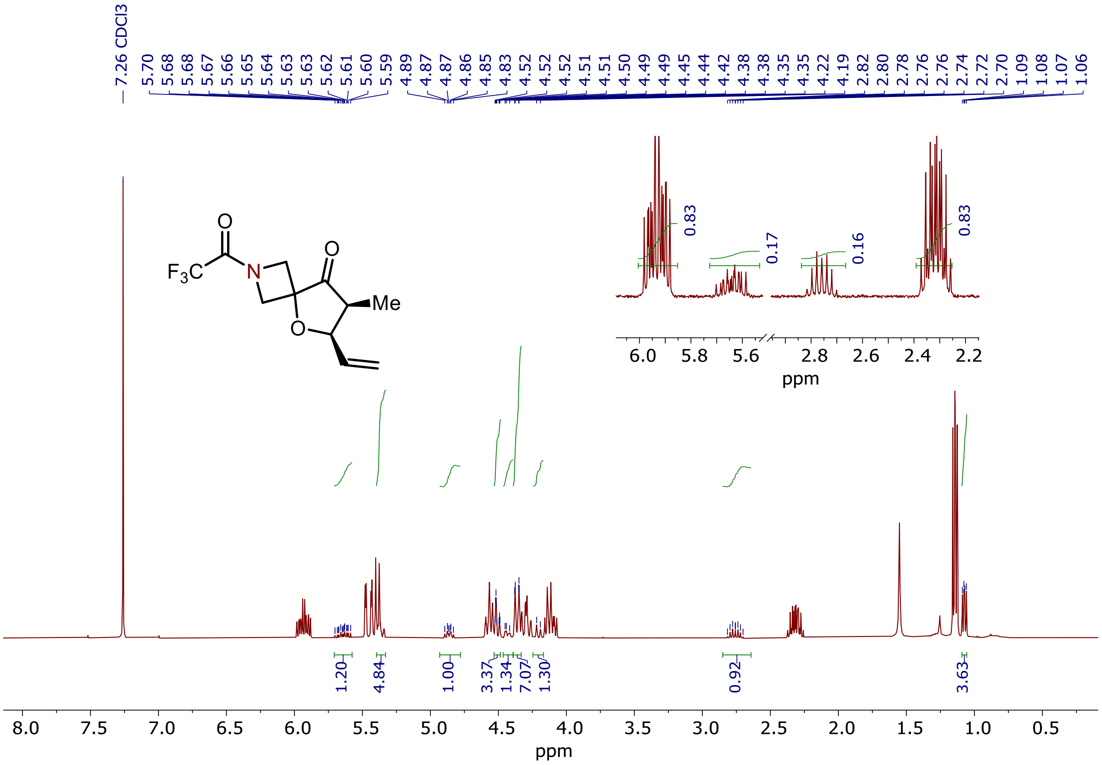

^{13}C NMR (126 MHz, $CDCl_3$) of **7-Methyl-2-(2,2,2-trifluoroacetyl)-6-vinyl-5-oxa-2-azaspiro[3.4]octan-8-one:** 17:83 *syn:anti*-**4q** (*syn*-**4q** peaks labelled)

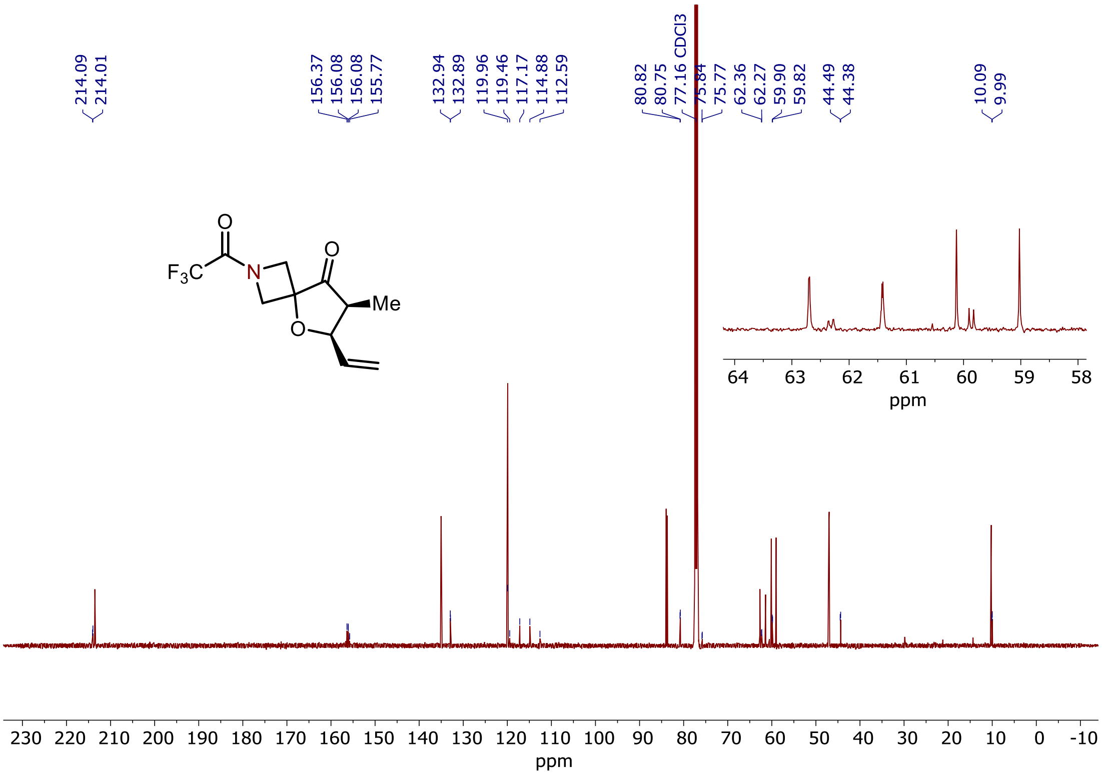

^{19}F NMR (377 MHz, $CDCl_3$) of **7-Methyl-2-(2,2,2-trifluoroacetyl)-6-vinyl-5-oxa-2-azaspiro[3.4]octan-8-one:** 17:83 *syn:anti*-**4q** (*syn*-**4q** peaks labelled)

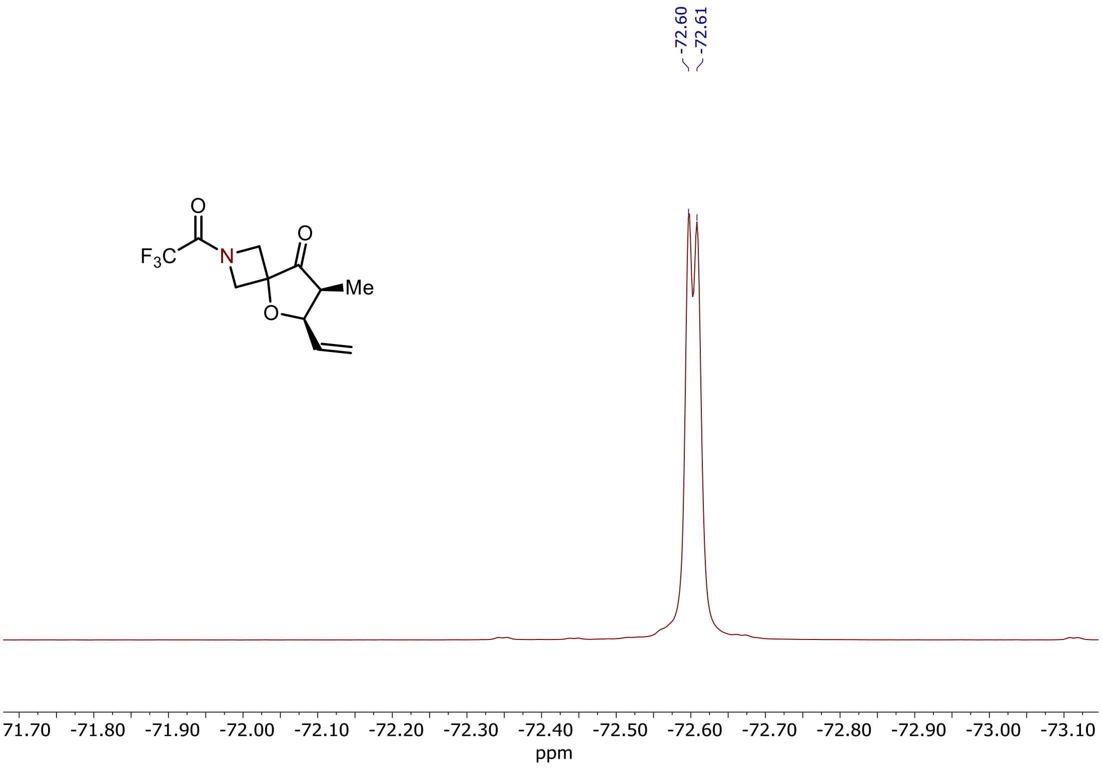

^{1}H NMR (400 MHz, $CDCl_3$) of **6-Methyl-2-(2,2,2-trifluoroacetyl)-5-oxa-2-azaspiro[3.5]nonan-9-one: 4r** (*see procedure*)

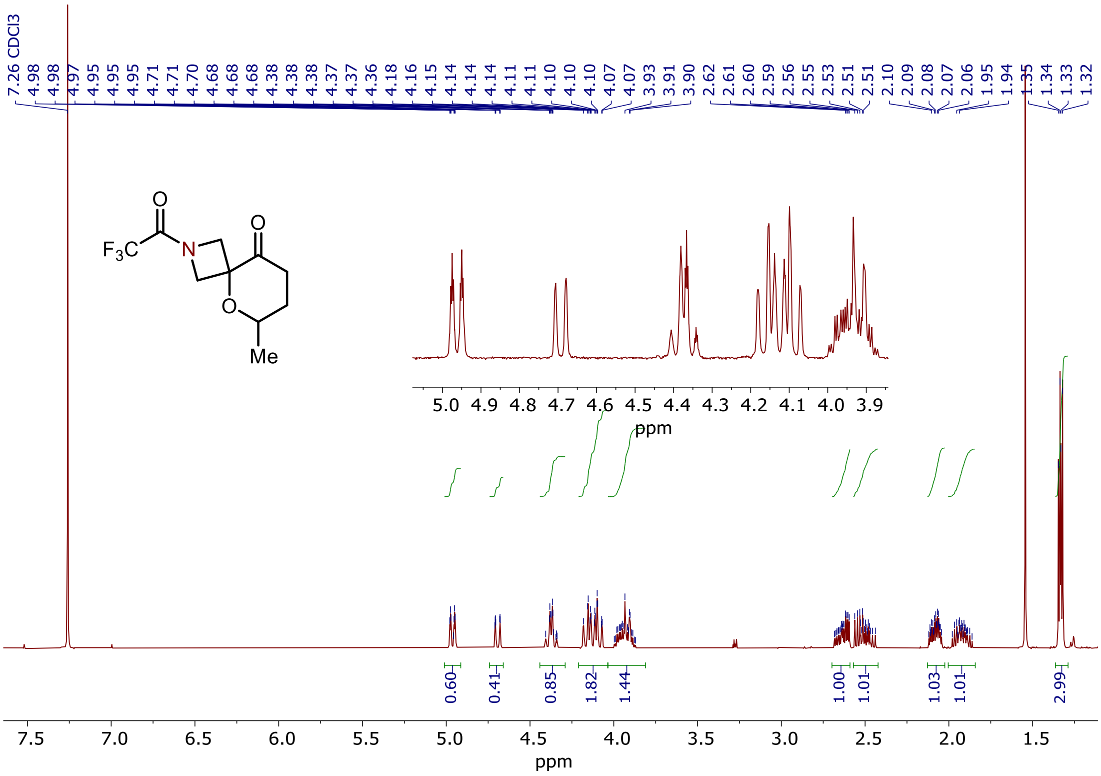

^{13}C NMR (126 MHz, $CDCl_3$) of **6-Methyl-2-(2,2,2-trifluoroacetyl)-5-oxa-2-azaspiro[3.5]nonan-9-one: 4r**

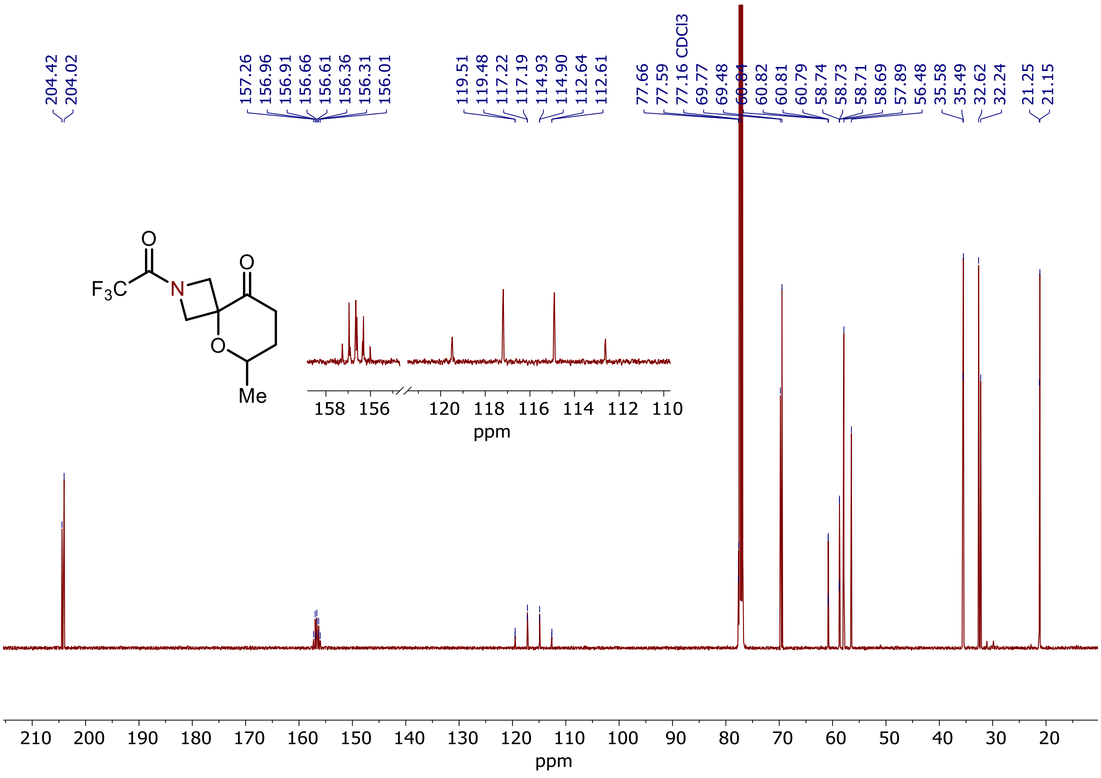

^{19}F NMR (377 MHz, $CDCl_3$) of **6-Methyl-2-(2,2,2-trifluoroacetyl)-5-oxa-2-azaspiro[3.5]nonan-9-one: 4r**

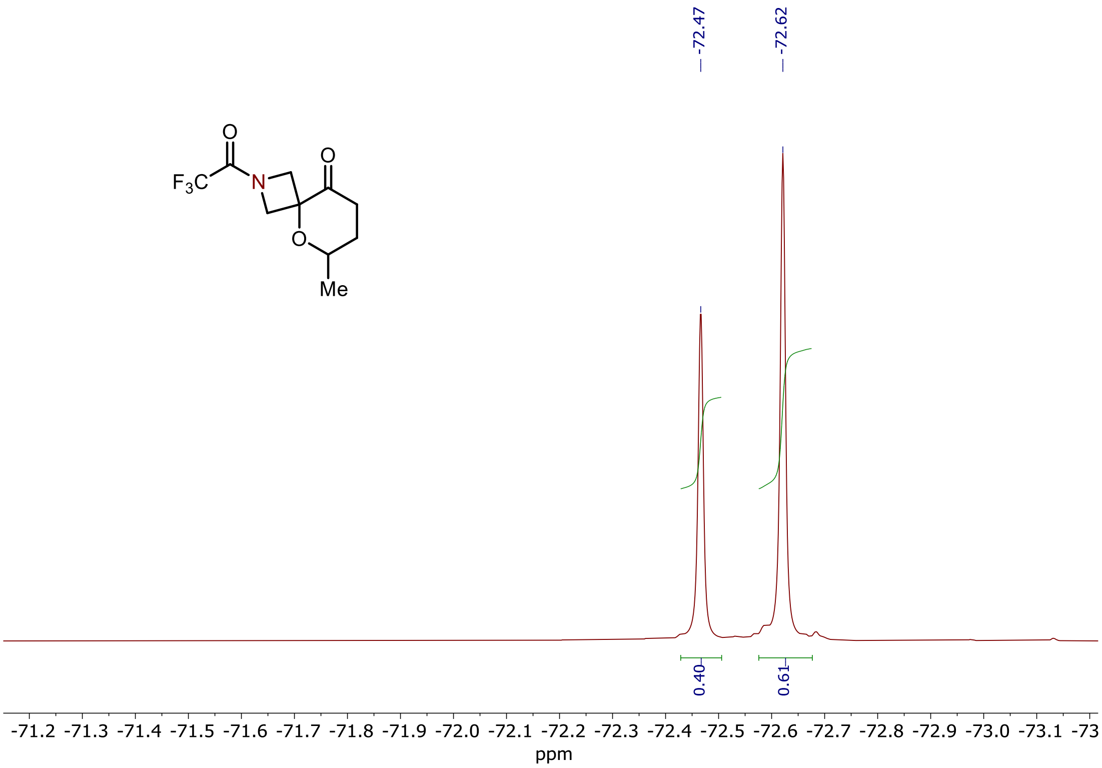

^{1}H NMR (500 MHz, $CDCl_3$) of **1-(2,2,2-Trifluoroacetyl)-3'H-spiro[azetidine-3,2'-benzofuran]-3'-one: 4t** (*see procedure*)

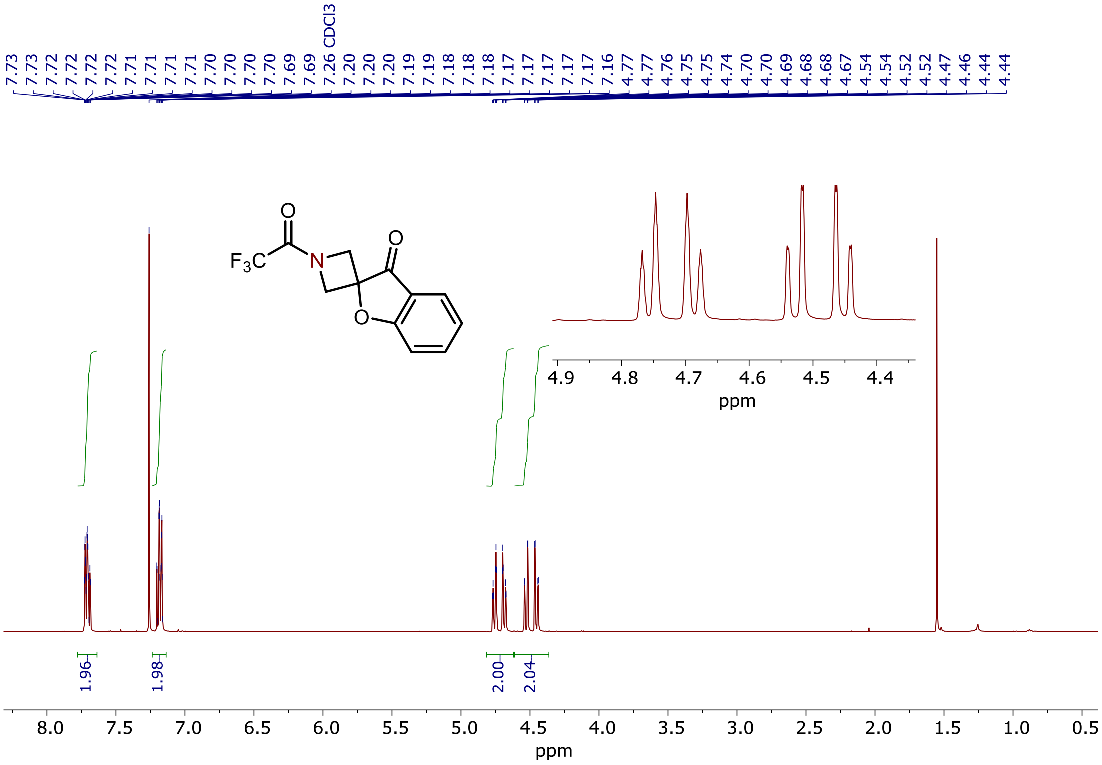

^{13}C NMR (126 MHz, $CDCl_3$) of **1-(2,2,2-Trifluoroacetyl)-3'H-spiro[azetidine-3,2'-benzofuran]-3'-one: 4t**

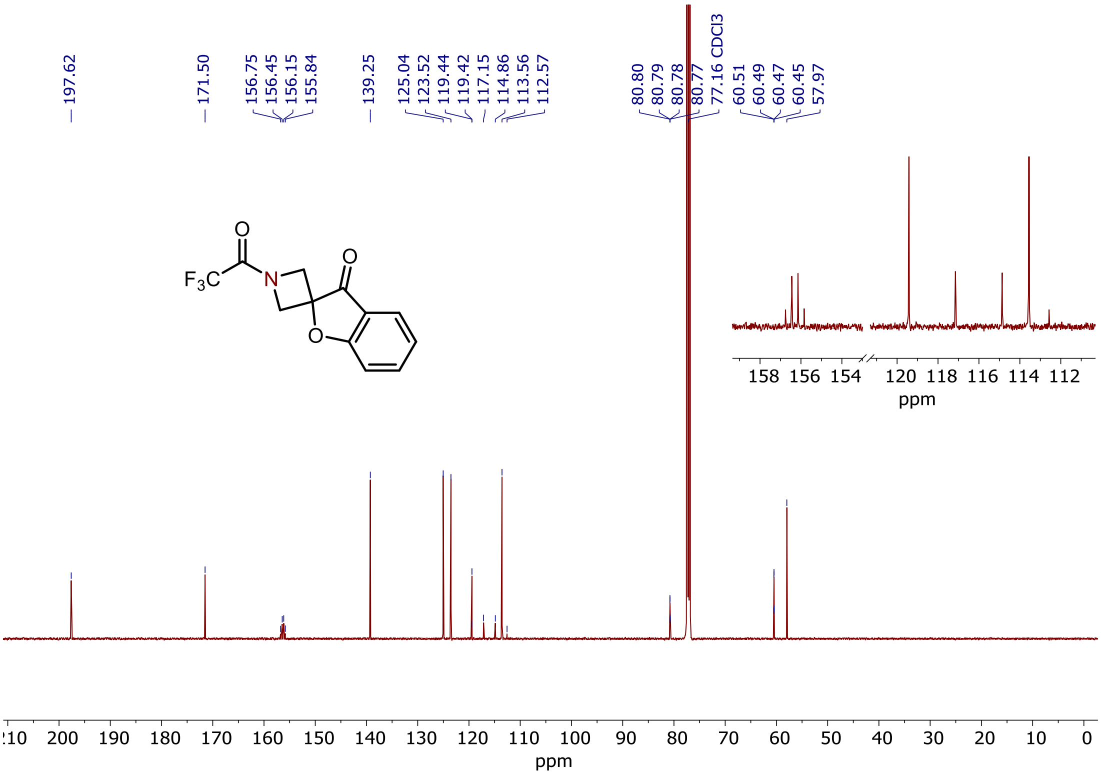

^{19}F NMR (377 MHz, $CDCl_3$) of **1-(2,2,2-Trifluoroacetyl)-3'H-spiro[azetidine-3,2'-benzofuran]-3'-one: 4t**

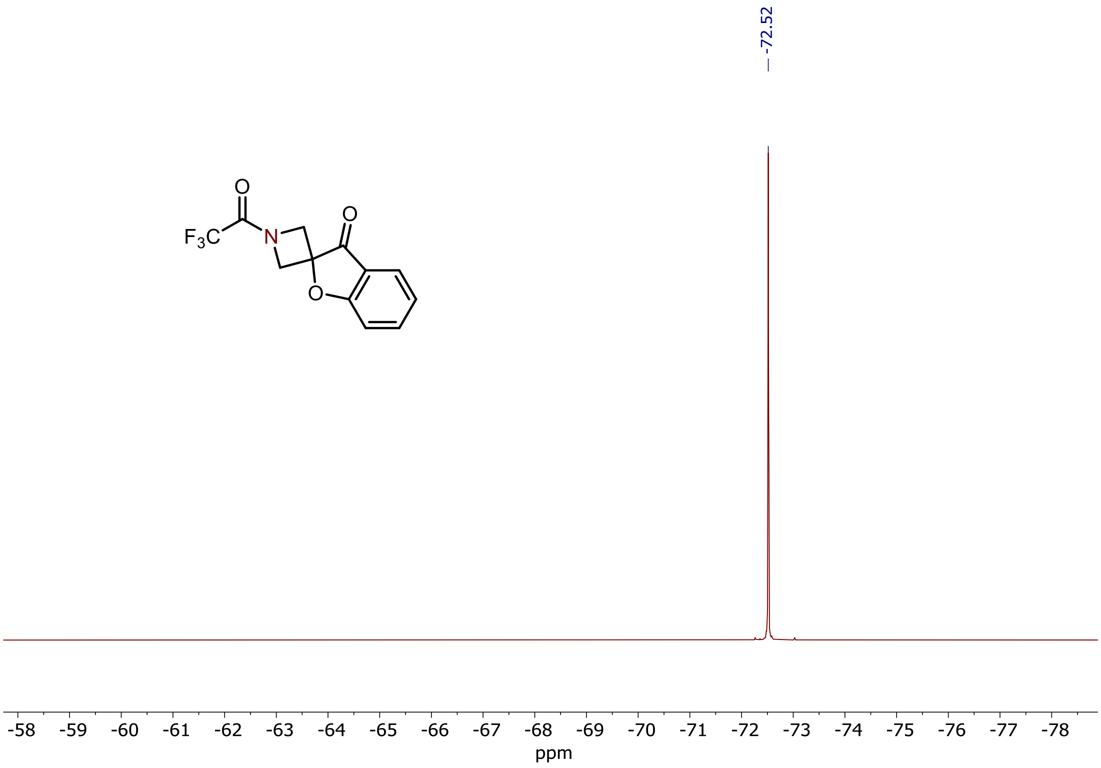

^{1}H NMR (500 MHz, $CDCl_3$) of **2,2,2-Trifluoro-1-(3'-hydroxy-3'H-spiro[azetidine-3,2'-furo[3,2-b]pyridin]-1-yl)ethan-1-one: 4u′** (*see procedure*)

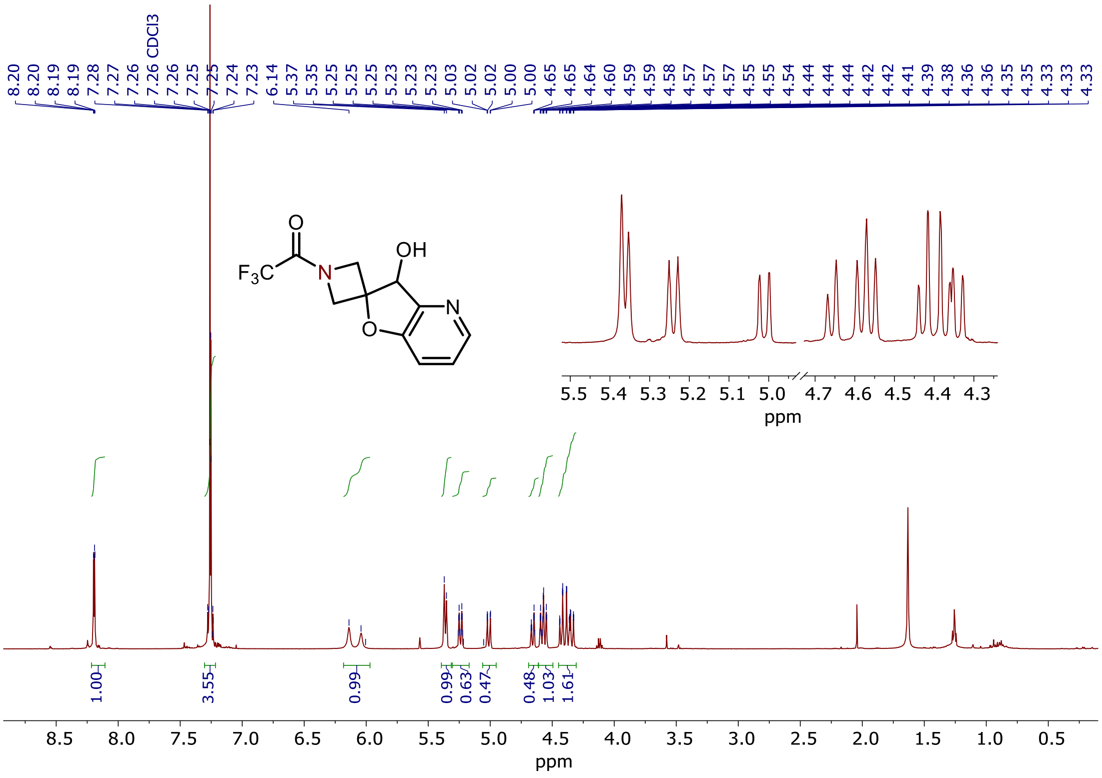

^{13}C NMR (126 MHz, $CDCl_3$) of **2,2,2-Trifluoro-1-(3'-hydroxy-3'H-spiro[azetidine-3,2'-furo[3,2-b]pyridin]-1-yl)ethan-1-one: 4u′**

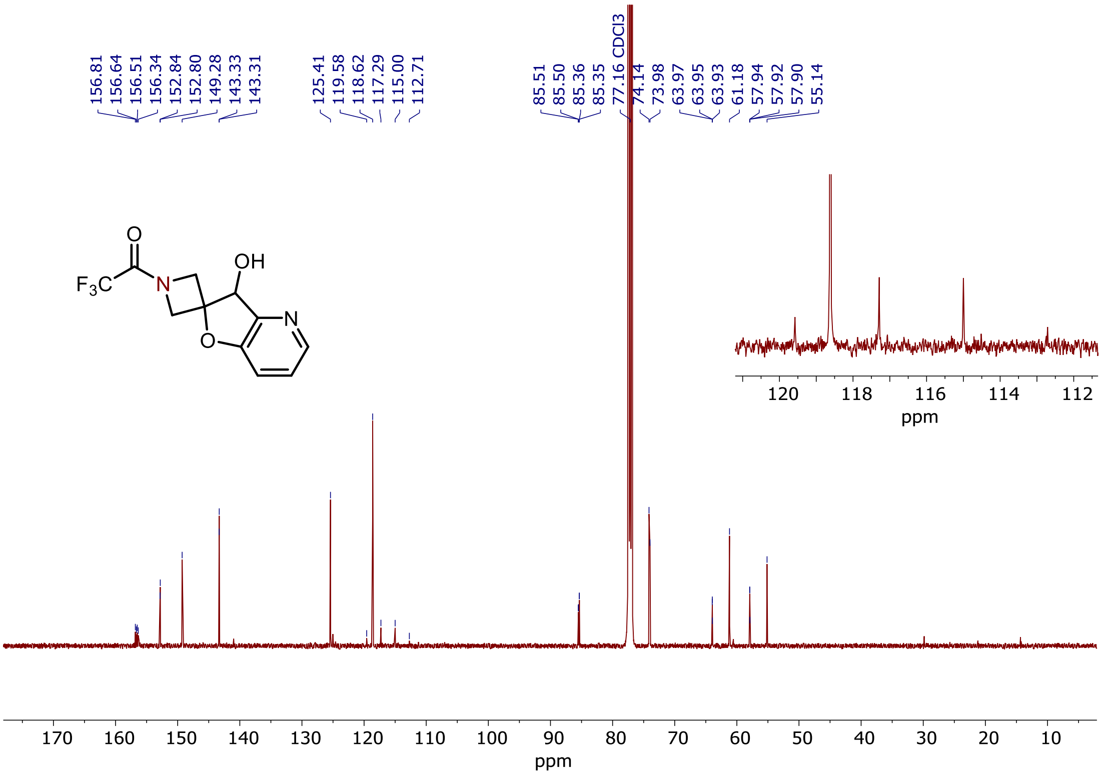

^{19}F NMR (377 MHz, $CDCl_3$) of **2,2,2-Trifluoro-1-(3'-hydroxy-3'H-spiro[azetidine-3,2'-furo[3,2-b]pyridin]-1-yl)ethan-1-one: 4u′**

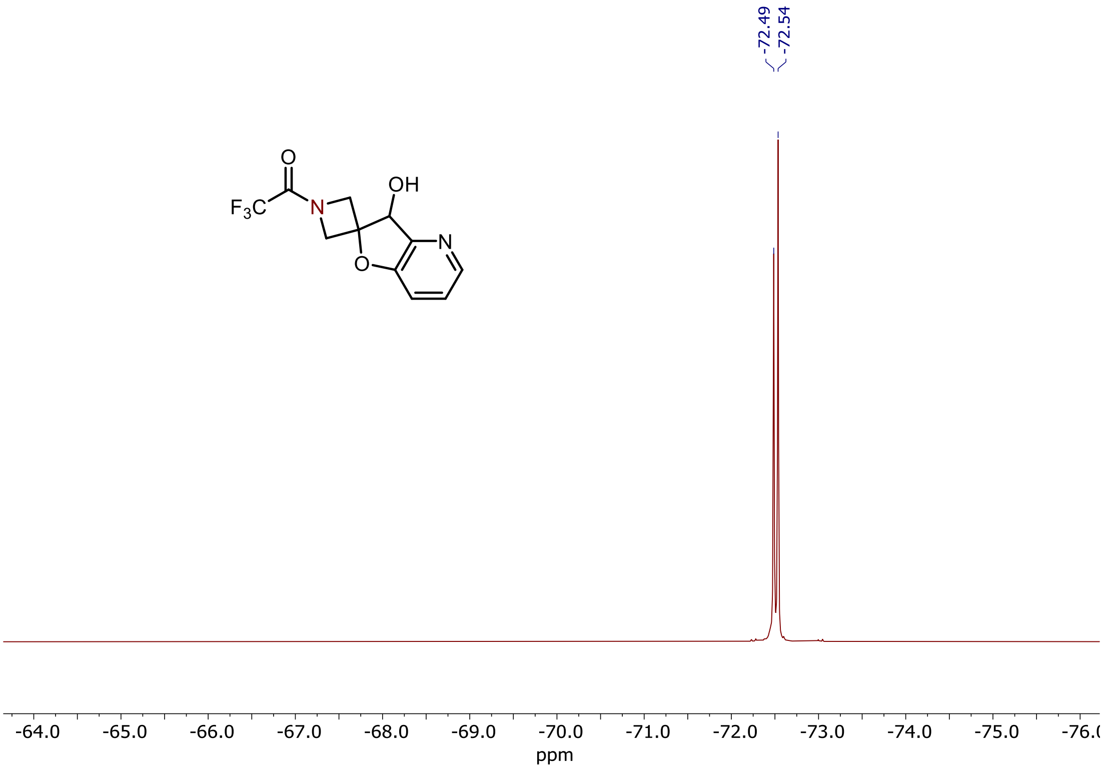

^{1}H NMR (500 MHz, $CDCl_3$) of **1-(2,2,2-Trifluoroacetyl)-3'H-spiro[azetidine-3,2'-naphtho[2,3-b]furan]-3'-one: 4v** (*see procedure*)

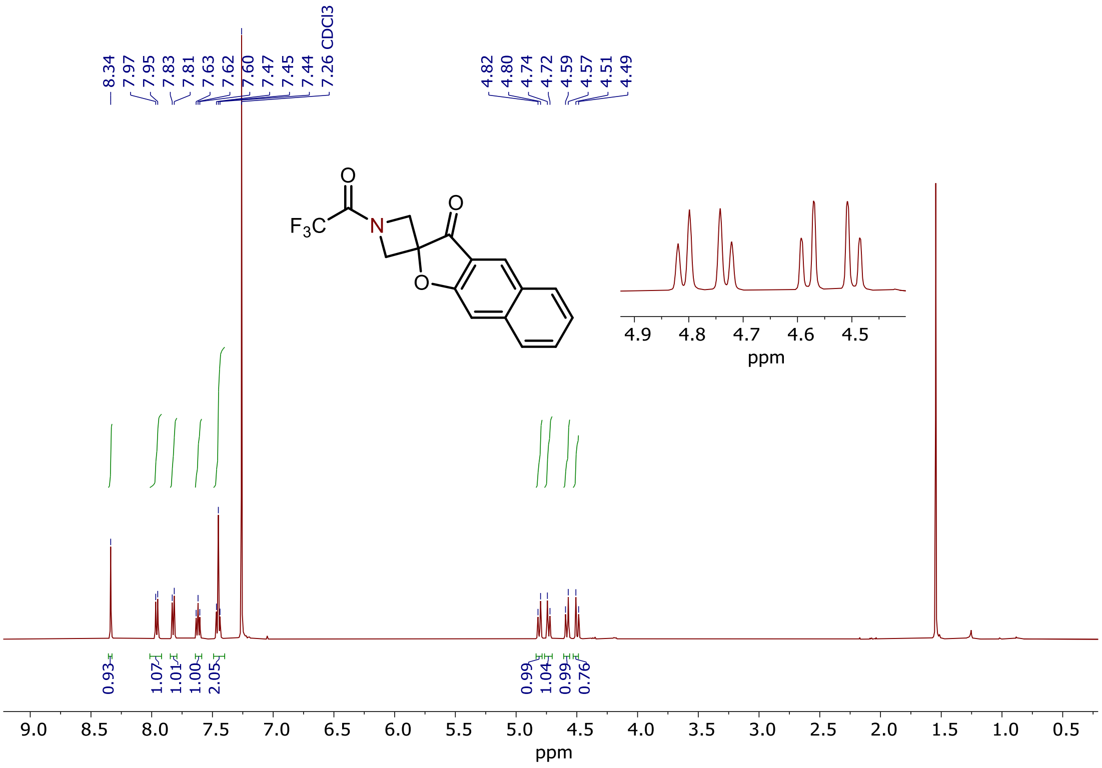

^{13}C NMR (126 MHz, $CDCl_3$) of **1-(2,2,2-Trifluoroacetyl)-3'H-spiro[azetidine-3,2'-naphtho[2,3-b]furan]-3'-one: 4v**

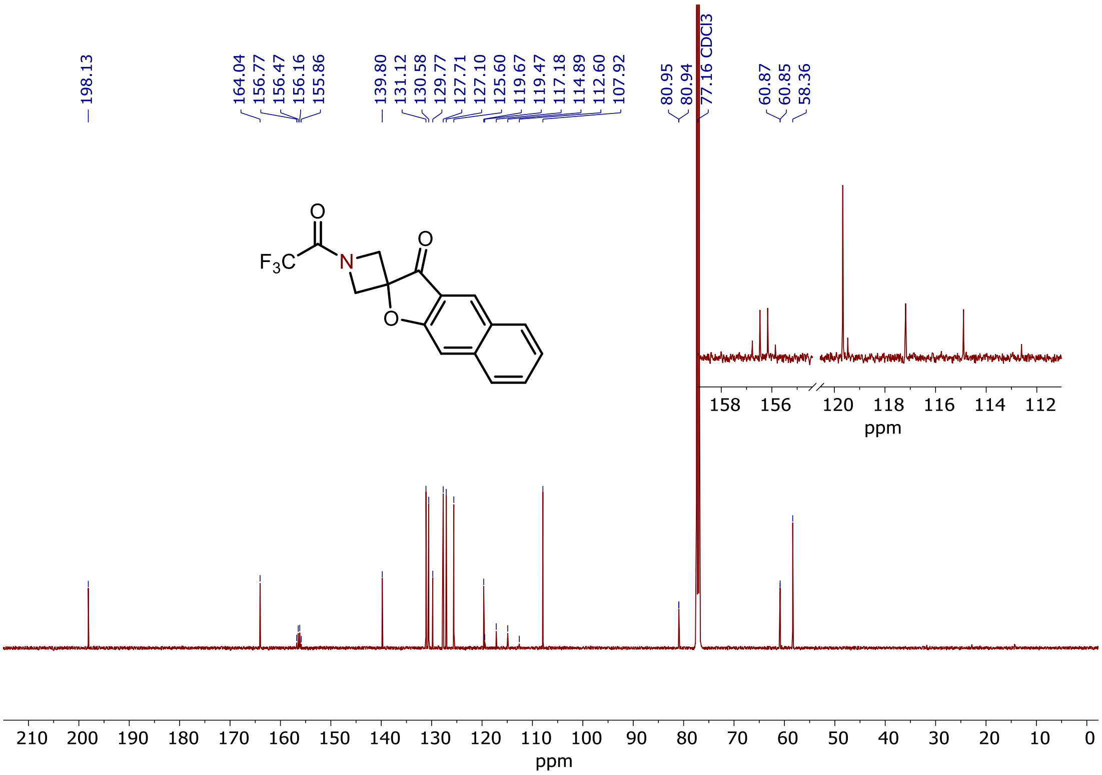

^{19}F NMR (377 MHz, $CDCl_3$) of **1-(2,2,2-Trifluoroacetyl)-3'H-spiro[azetidine-3,2'-naphtho[2,3-b]furan]-3'-one: 4v**

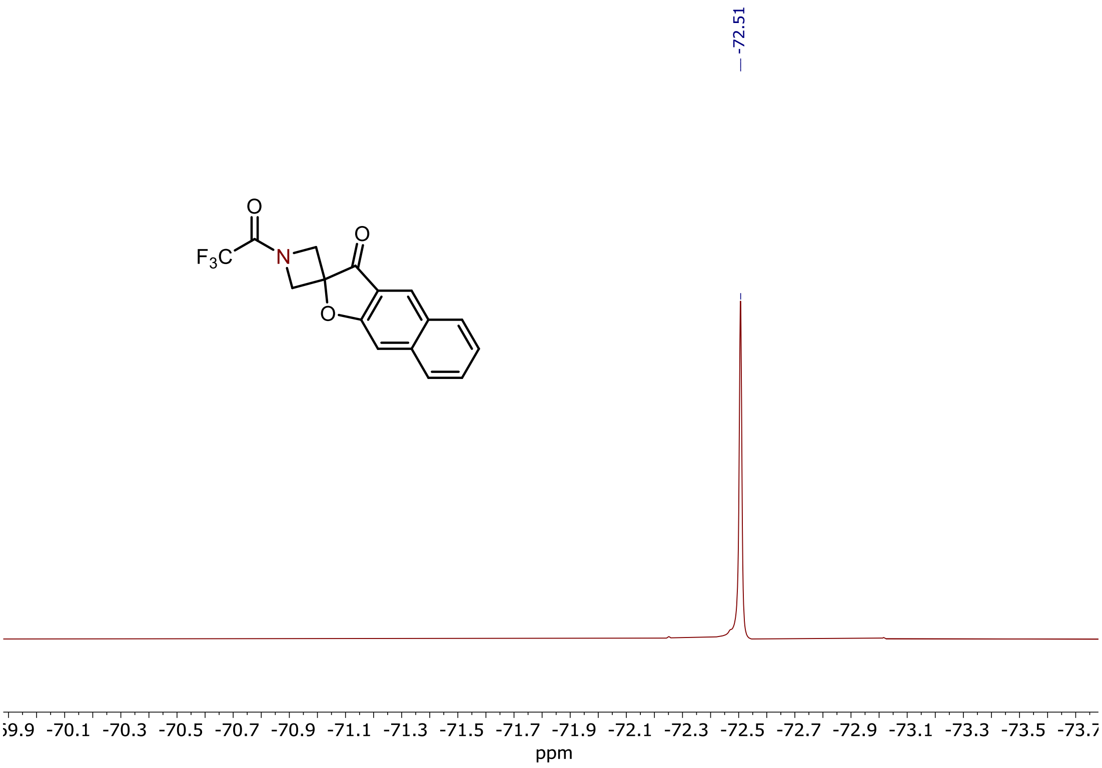

^{1}H NMR (400 MHz, CD_2Cl_2) of **2-Methyl-6-((trifluoromethyl)sulfonyl)-1-oxa-6-azaspiro[3.3]heptan-3-one: 4aa** (*see procedure*)

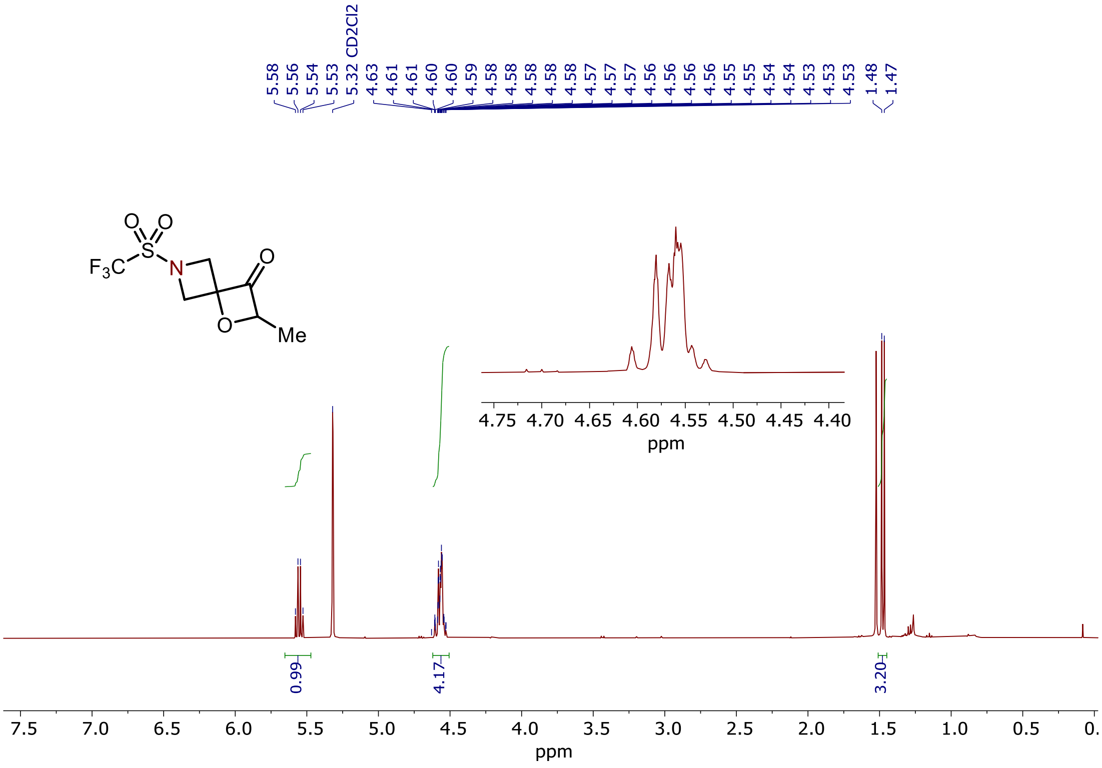

^{13}C NMR (126 MHz, CD_2Cl_2) of **2-Methyl-6-((trifluoromethyl)sulfonyl)-1-oxa-6-azaspiro[3.3]heptan-3-one: 4aa**

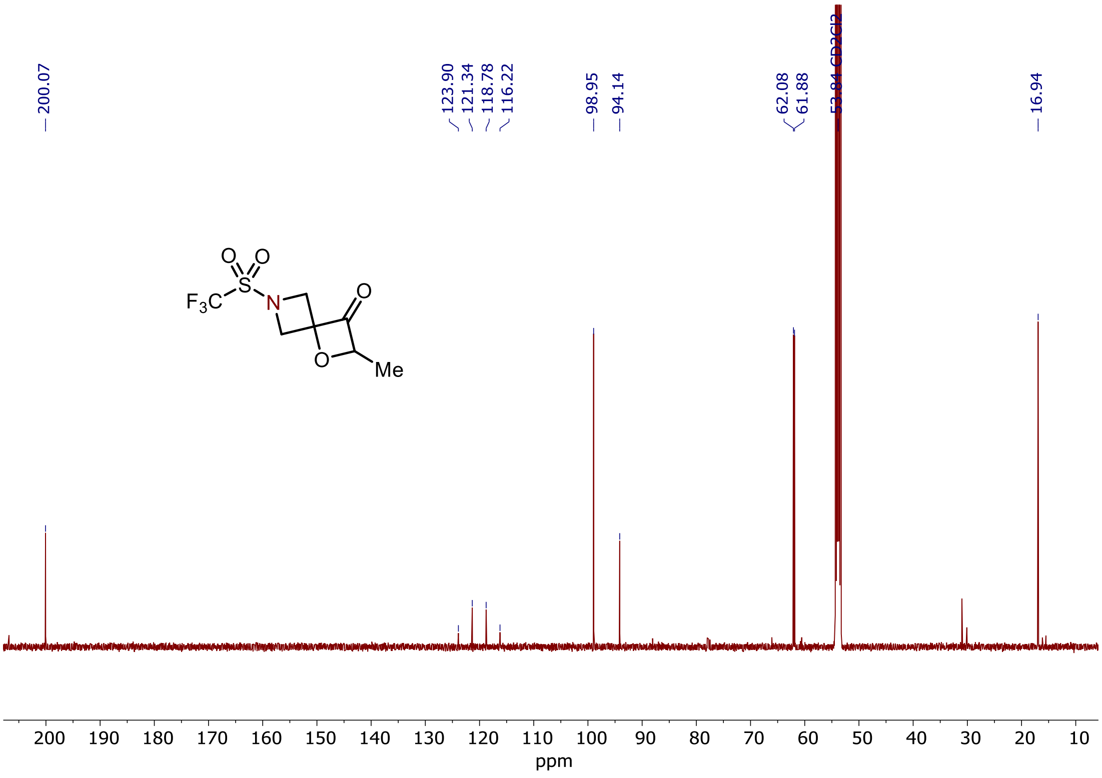

^{19}F NMR (377 MHz, $CDCl_3$) of **2-Methyl-6-((trifluoromethyl)sulfonyl)-1-oxa-6-azaspiro[3.3]heptan-3-one: 4aa**

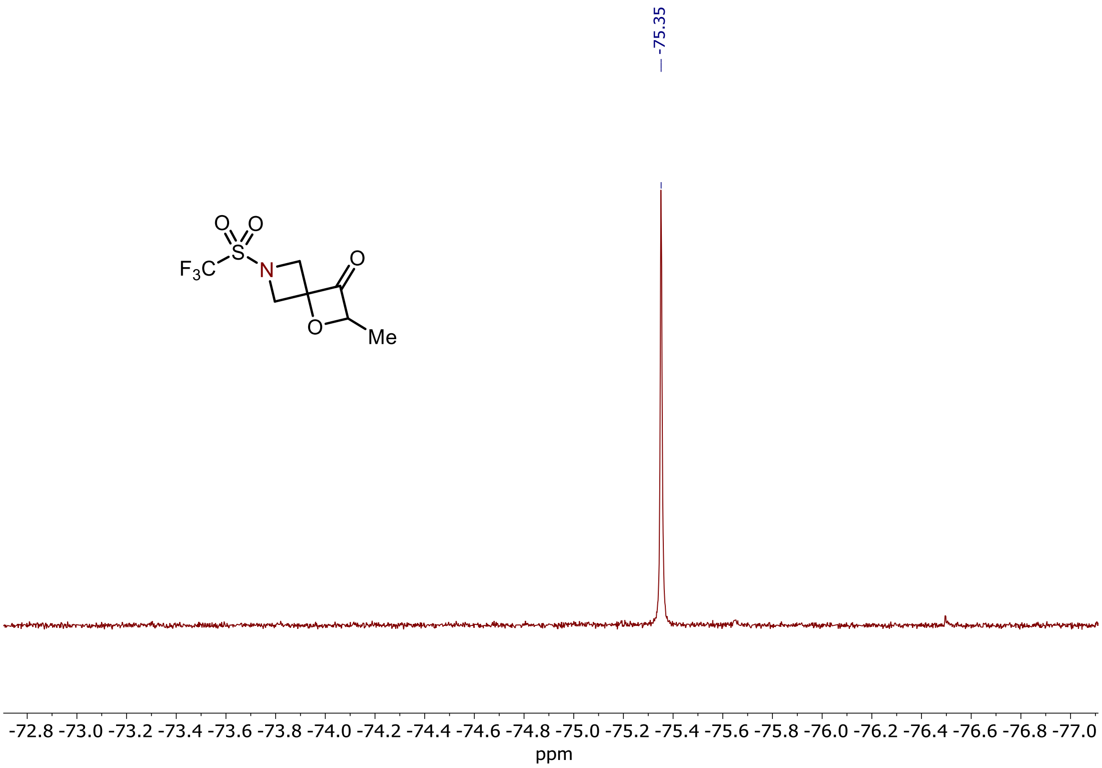

^1H NMR (400 MHz, $CDCl_3$) of **6-Methyl-2-(2,2,2-trifluoroacetyl)-5-oxa-2-azaspiro[3.4]octan-8-one: 4bb** (*see procedure*)

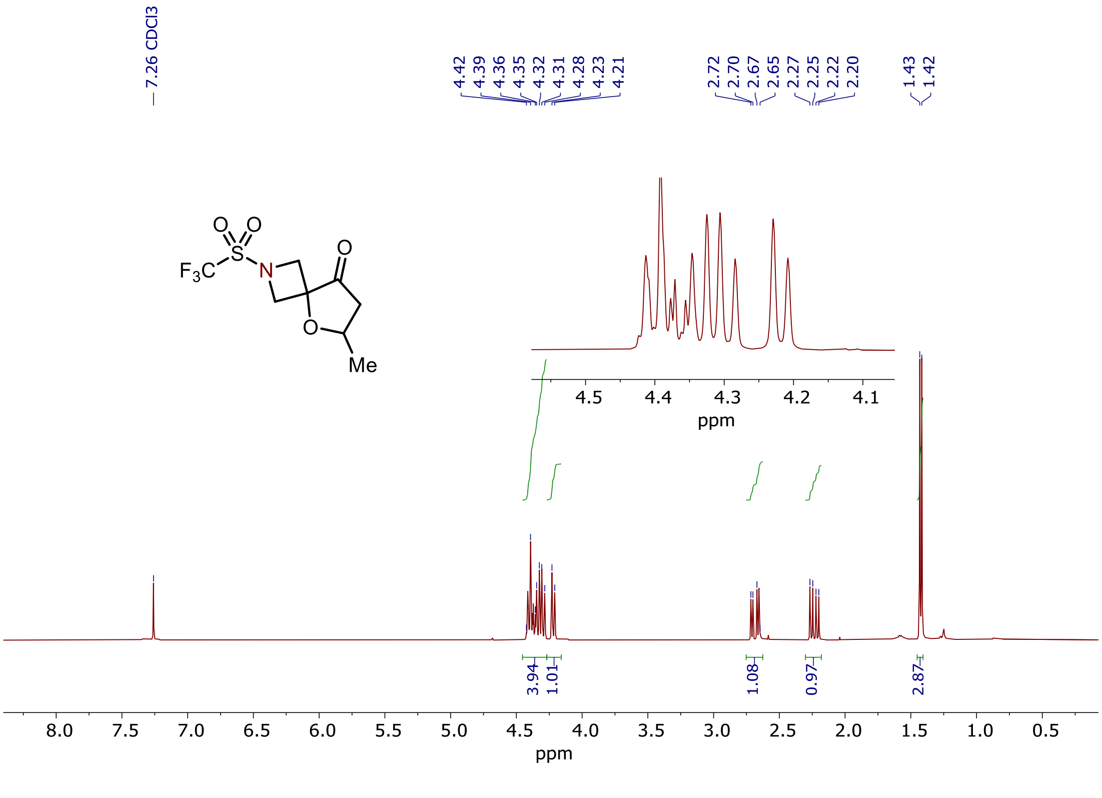

^{13}C NMR (126 MHz, CDCl$_3$) of **6-Methyl-2-(2,2,2-trifluoroacetyl)-5-oxa-2-azaspiro[3.4]octan-8-one: 4bb**

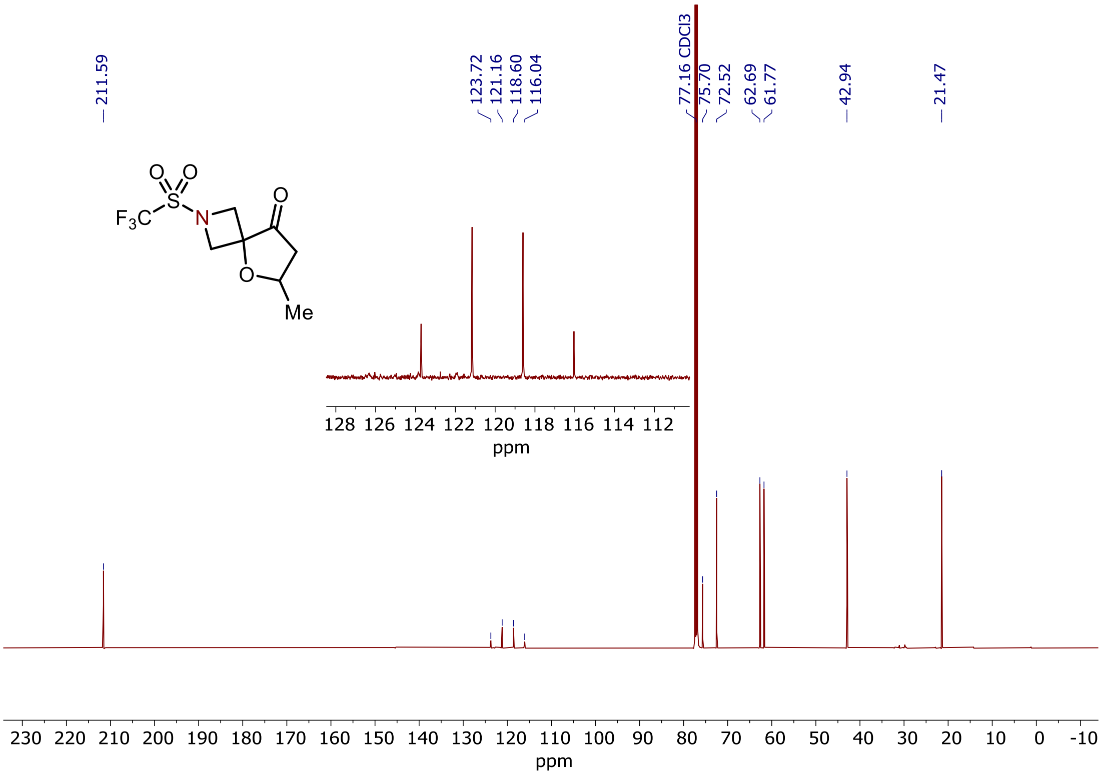

^{19}F NMR (377 MHz, CDCl$_3$) of **6-Methyl-2-(2,2,2-trifluoroacetyl)-5-oxa-2-azaspiro[3.4]octan-8-one: 4bb**

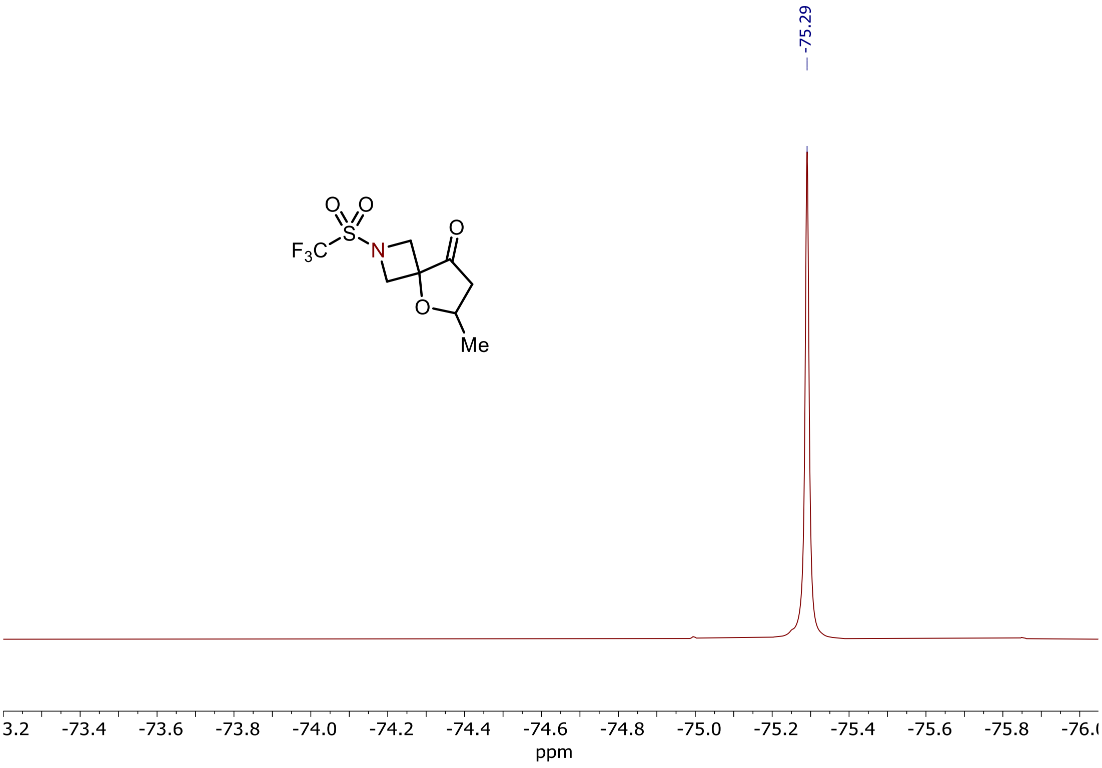

^{1}H NMR (500 MHz, $CDCl_3$) of **2-((Trifluoromethyl)sulfonyl)-5-oxa-2-azadispiro[3.1.56.24]tridecan-13-one: 4cc** (*see procedure*)

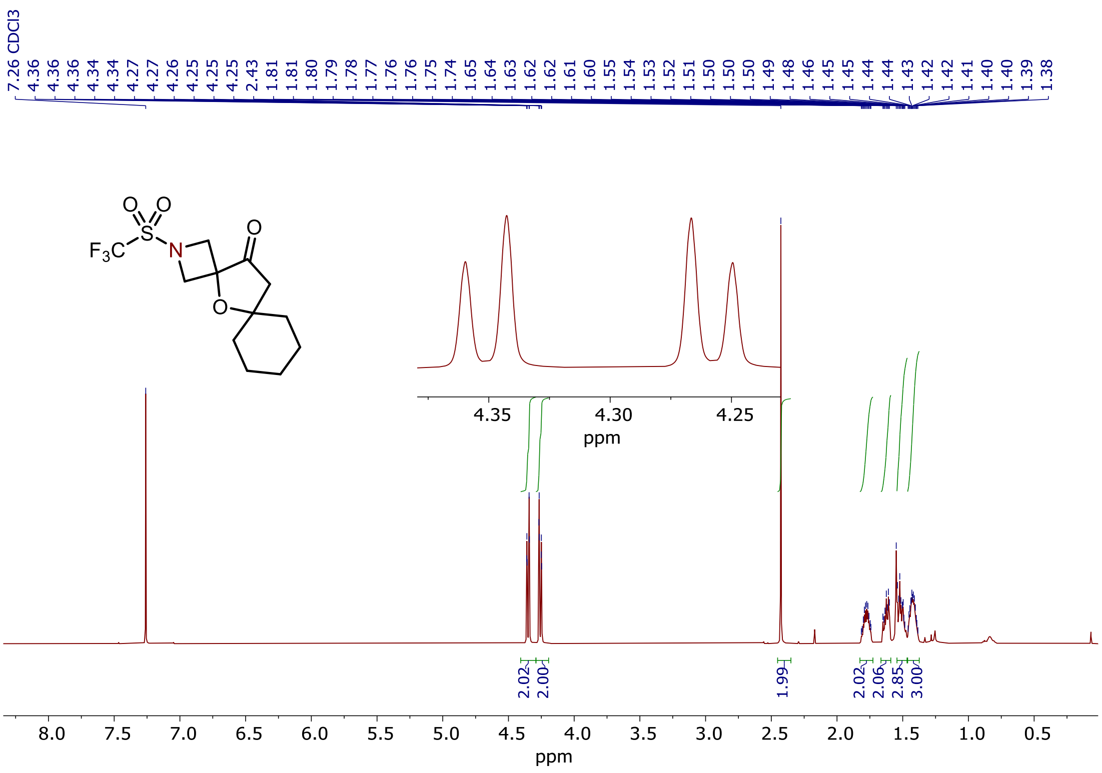

^{13}C NMR (126 MHz, $CDCl_3$) of **2-((Trifluoromethyl)sulfonyl)-5-oxa-2-azadispiro[3.1.56.24]tridecan-13-one: 4cc**

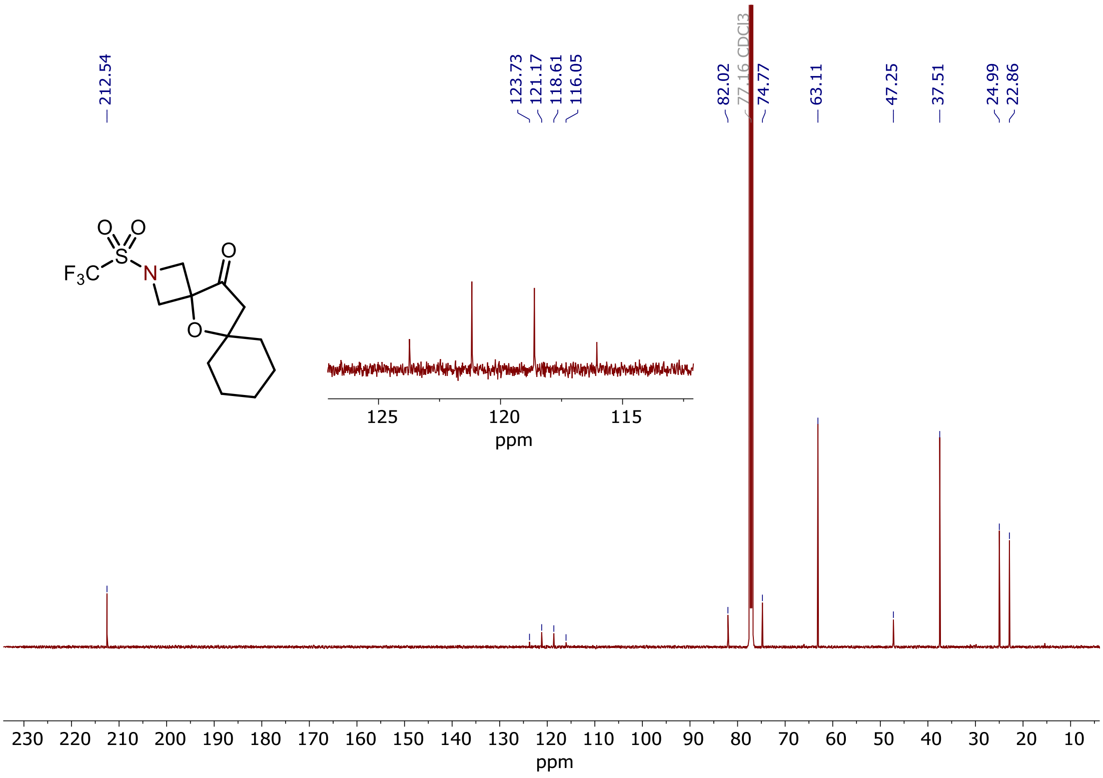

^{19}F NMR (377 MHz, $CDCl_3$) of **2-((Trifluoromethyl)sulfonyl)-5-oxa-2-azadispiro[3.1.56.24]tridecan-13-one: 4cc**

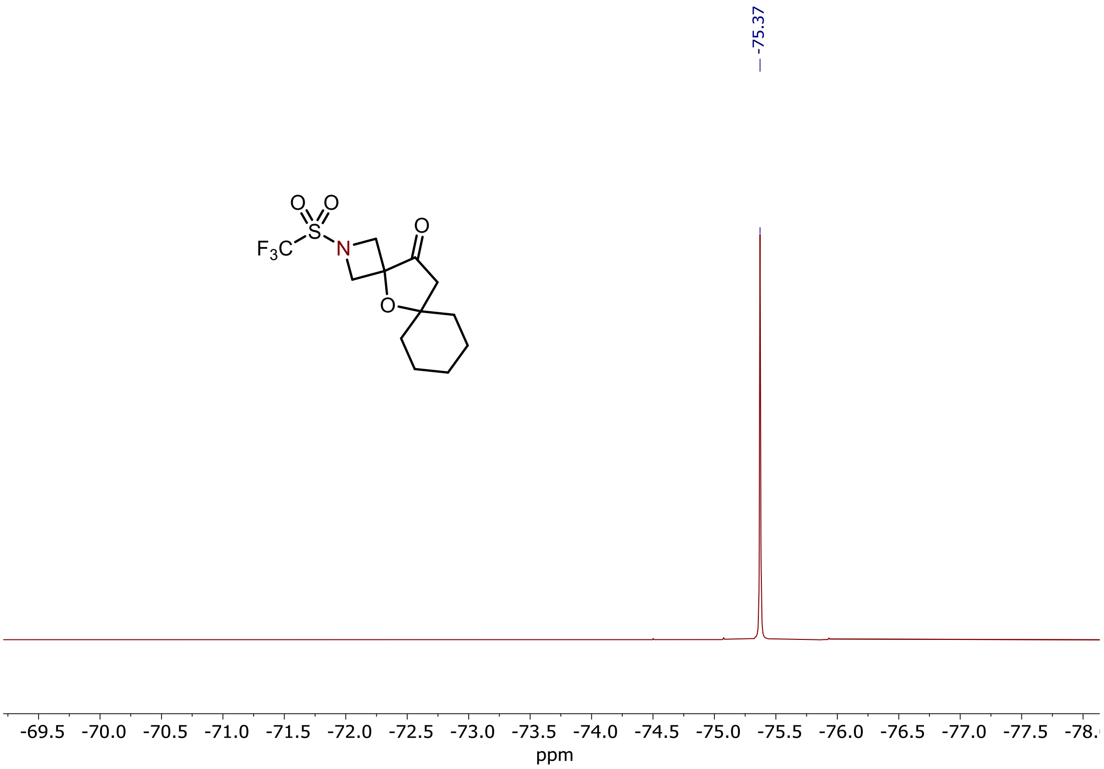

^{1}H NMR (400 MHz, $CDCl_3$) of ***tert*-butyl 2-methyl-3-oxo-1-oxa-6-azaspiro[3.3]heptane-6-carboxylate: 8a** (*see procedure*)

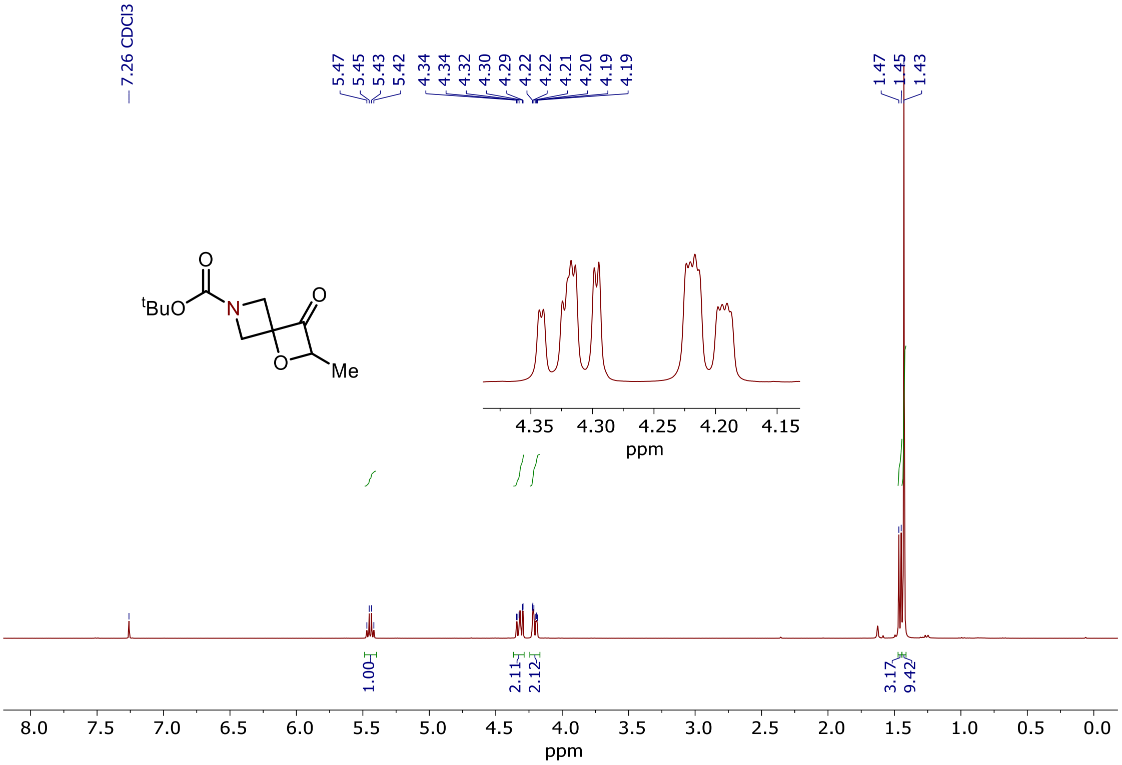

^{13}C NMR (101 MHz, $CDCl_3$) of ***tert*-butyl 2-methyl-3-oxo-1-oxa-6-azaspiro[3.3]heptane-6-carboxylate: 8a**

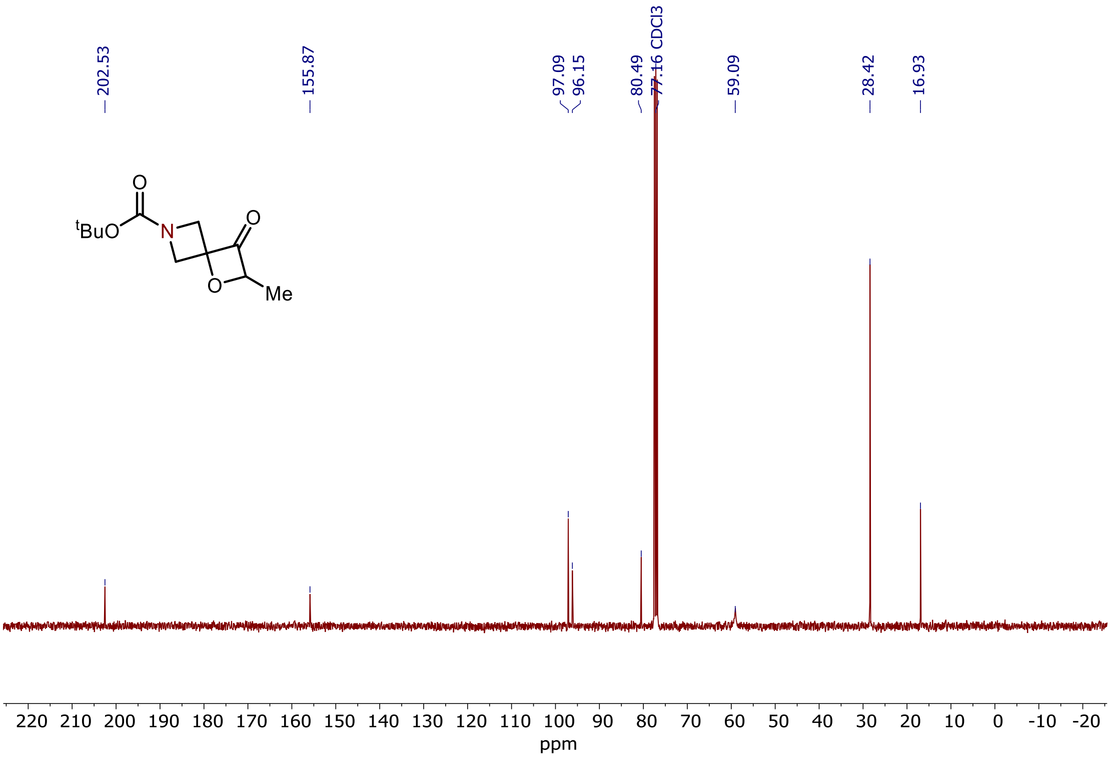

^{1}H NMR (400 MHz, $CDCl_3$) of ***tert*-butyl 13-oxo-5-oxa-2-azadispiro[3.1.5^{6}.2^{4}]tridecane-2-carboxylate: 8p** (*see procedure*)

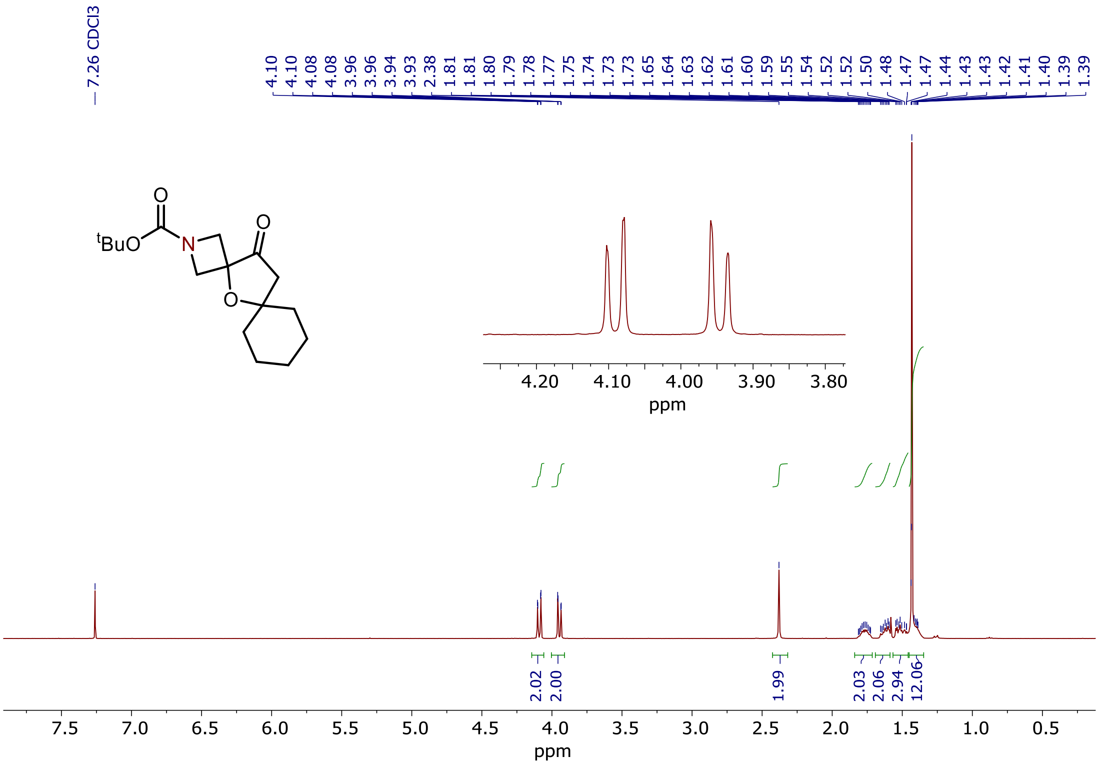

^{13}C NMR (126 MHz, $CDCl_3$) of ***tert*-butyl 13-oxo-5-oxa-2-azadispiro[3.1.5^6.2^4]tridecane-2-carboxylate: 8p**

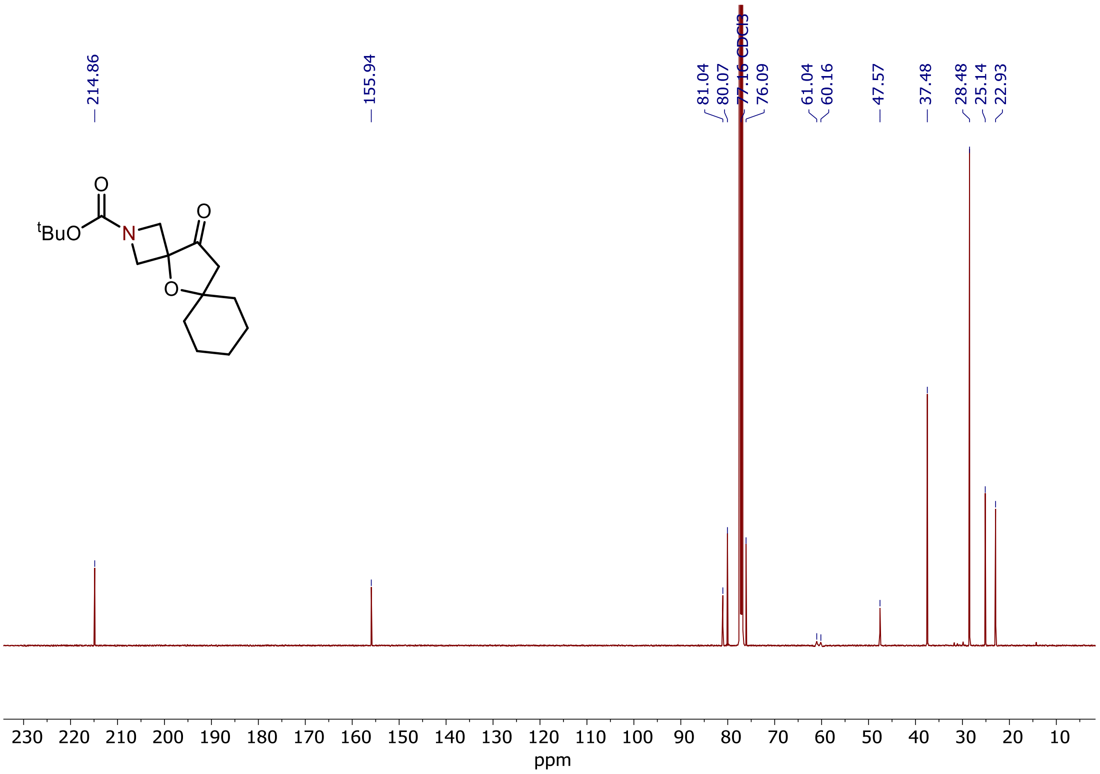

^{1}H NMR (400 MHz, $CDCl_3$) of ***tert*-butyl 3'-oxo-3'H-spiro[azetidine-3,2'-naphtho[2,3-b]furan]-1-carboxylate: 8v** (*see procedure*)

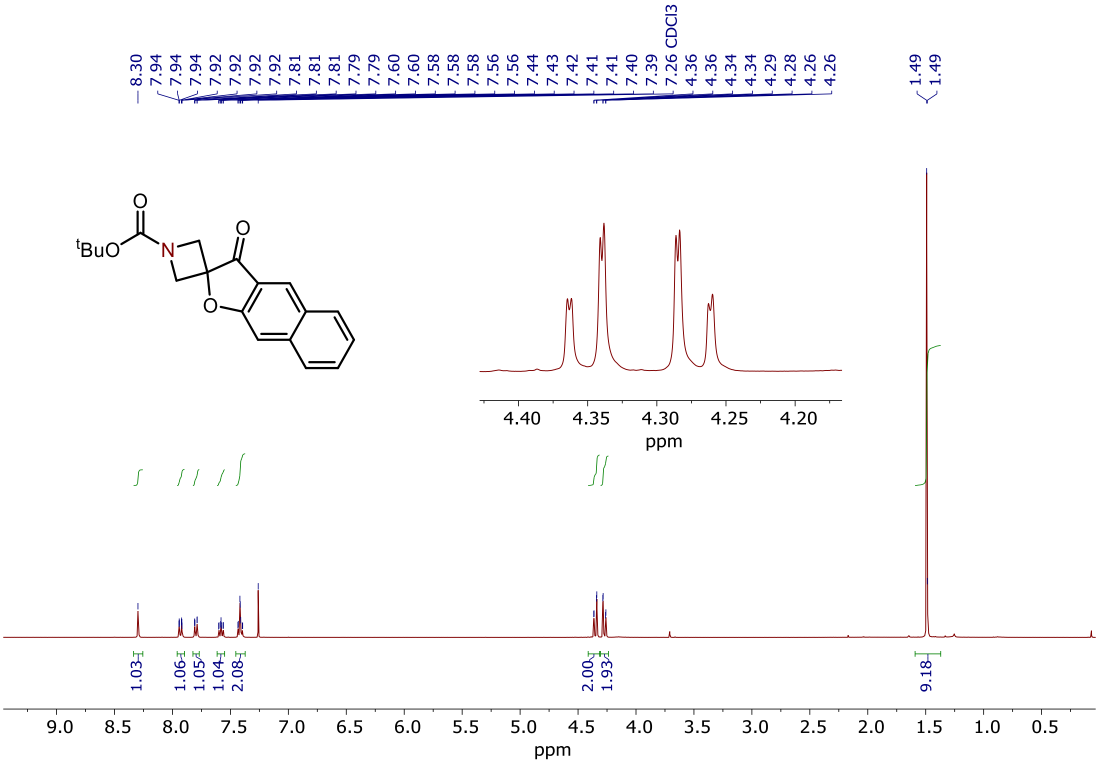

^{13}C NMR (126 MHz, $CDCl_3$) of **6-Methyl-2-(2,2,2-trifluoroacetyl)-5-oxa-2-azaspiro[3.4]octan-8-one: 8v**

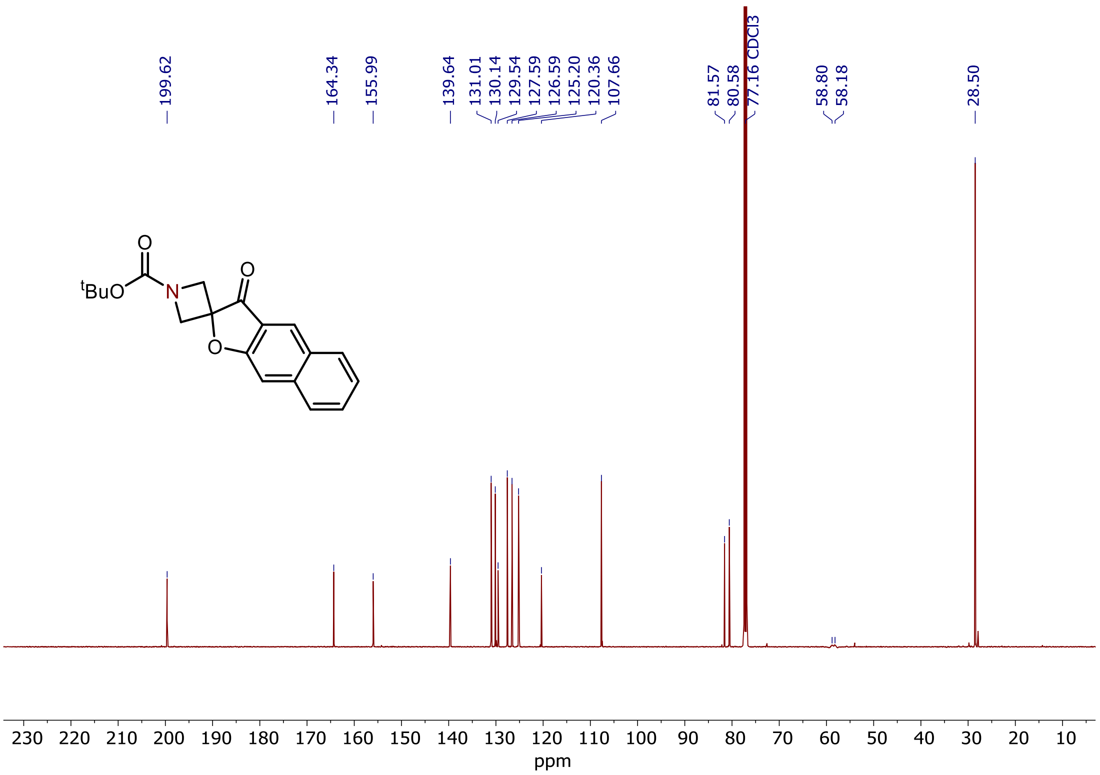

^{1}H NMR (400 MHz, CD_3OD) of **3-((3-(Methoxycarbonyl)-1-(2,2,2-trifluoroacetyl)azetidin-3-yl)oxy)pyridin-1-ium 2,2,2-trifluoroacetate: 4ua** (*see discussion*)

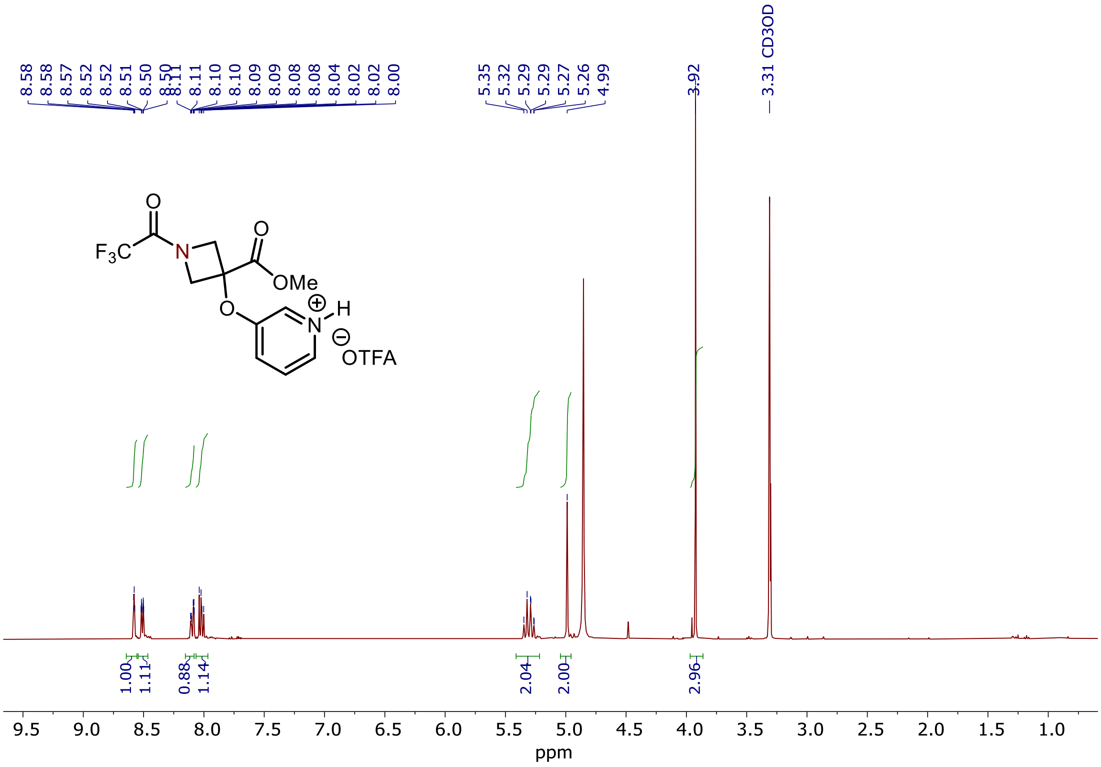

^{13}C NMR (126 MHz, CD_3OD) of **3-((3-(Methoxycarbonyl)-1-(2,2,2-trifluoroacetyl)azetidin-3-yl)oxy)pyridin-1-ium 2,2,2-trifluoroacetate: 4ua**

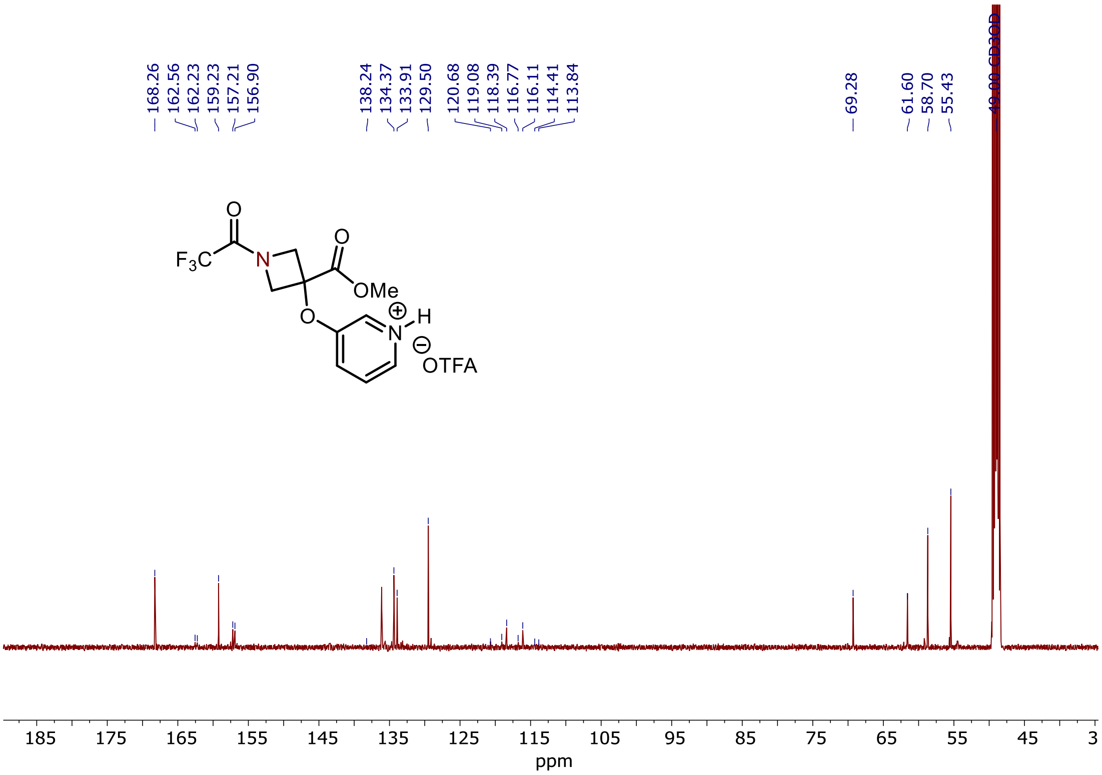

^{19}F NMR (377 MHz, CD_3OD) of **3-((3-(Methoxycarbonyl)-1-(2,2,2-trifluoroacetyl)azetidin-3-yl)oxy)pyridin-1-ium 2,2,2-trifluoroacetate: 4ua**

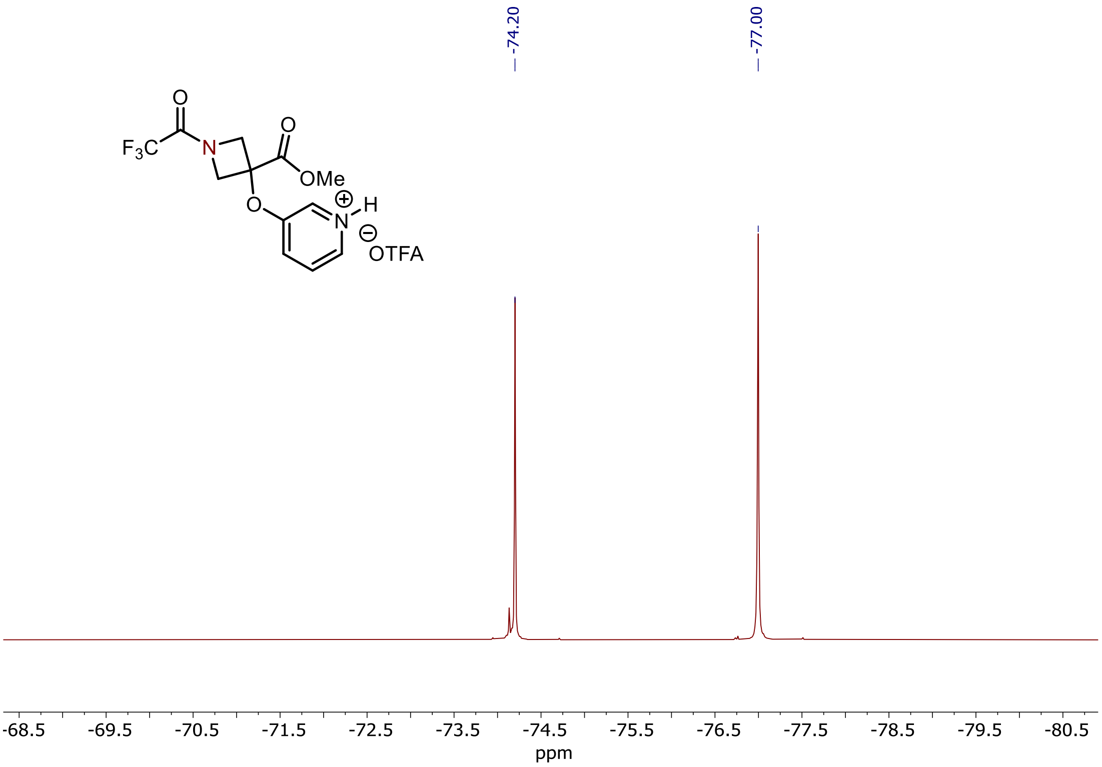

^{1}H NMR (400 MHz, $CDCl_3$) of **(E)-3-phenyl-1-(3-((triethylsilyl)oxy)-1-(2,2,2-trifluoroacetyl)azetidin-3-yl)prop-2-en-1-one: 9j** (*see discussion*)

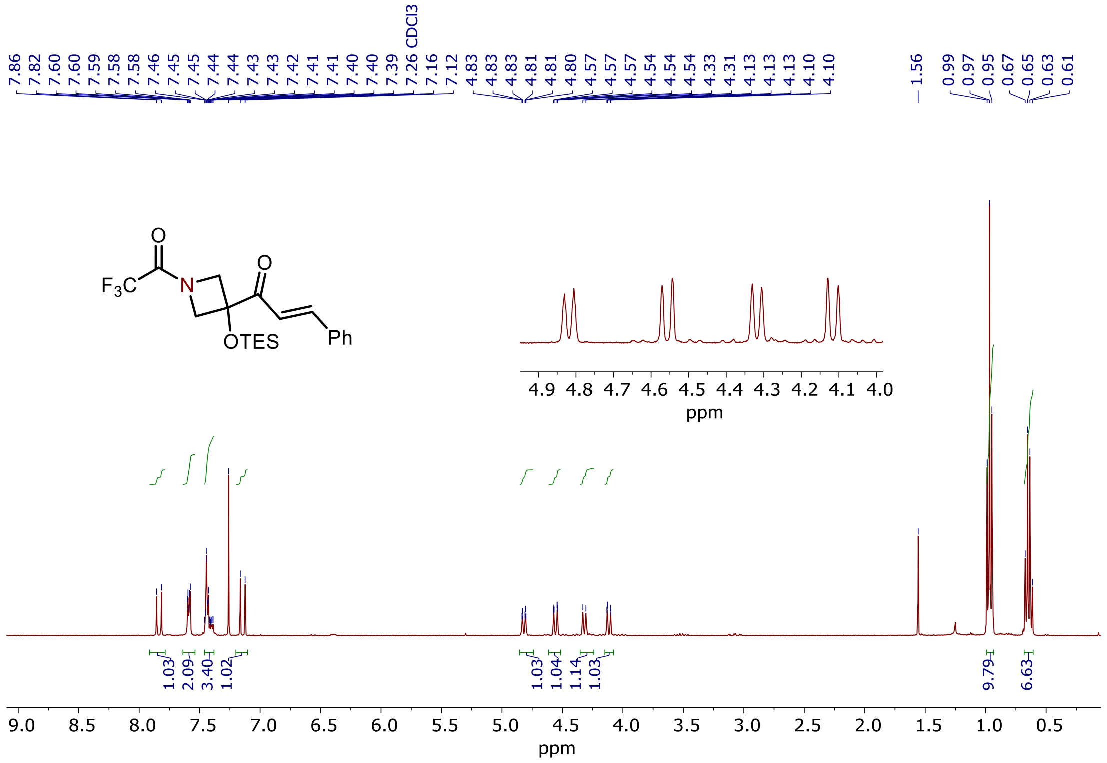

^{13}C NMR (126 MHz, $CDCl_3$) of **(E)-3-phenyl-1-(3-((triethylsilyl)oxy)-1-(2,2,2-trifluoroacetyl)azetidin-3-yl)prop-2-en-1-one: 9j**

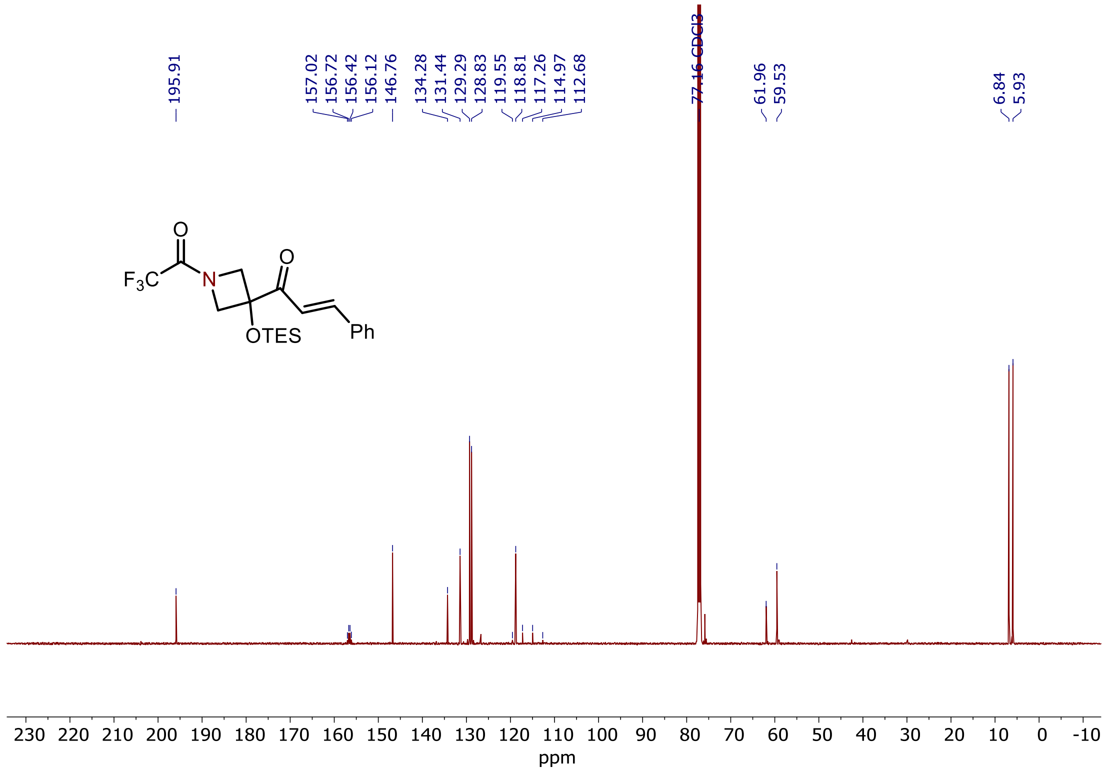

^{19}F NMR (377 MHz, $CDCl_3$) of **(*E*)-1-(3-Hydroxy-1-(2,2,2-trifluoroacetyl)azetidin-3-yl)-3-phenylprop-2-en-1-one: 9j**

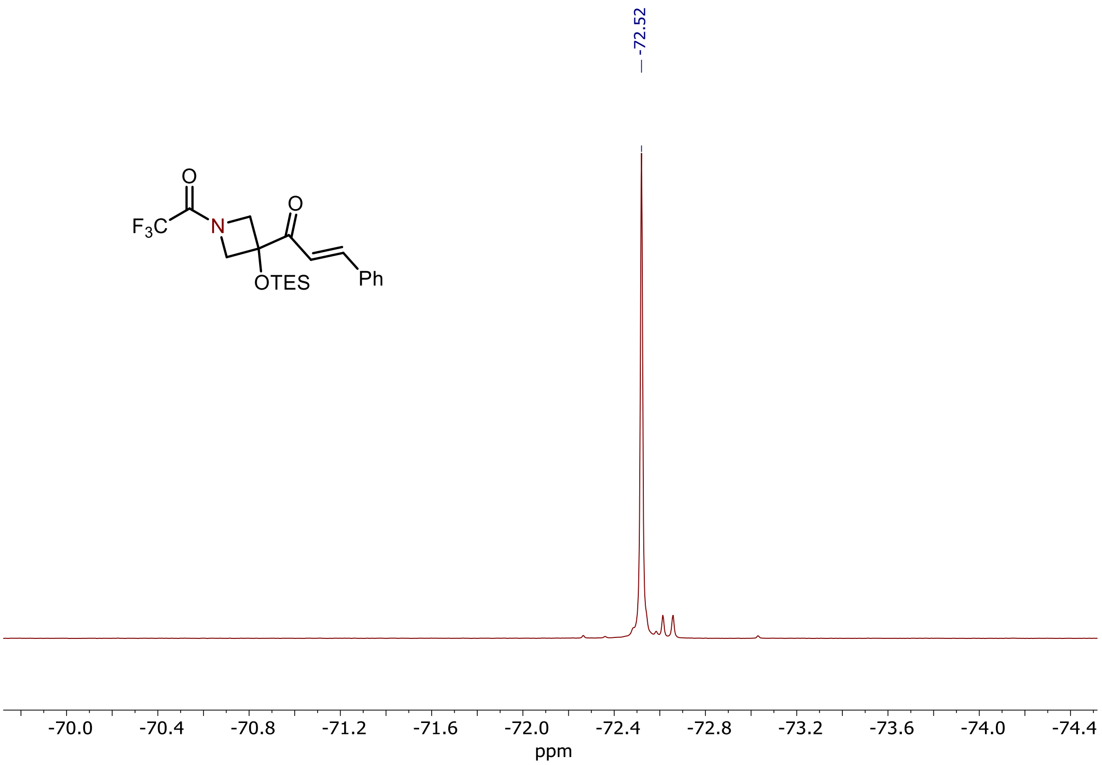

Supplement: Supplementary file 1 — Supplementary [file ANIE-60-11824-s001.pdf]
